# Psychiatric Safety of Tirzepatide in People With Obesity and No Known Major Psychopathology: A Post Hoc Analysis of SURMOUNT

**DOI:** 10.1002/oby.70122

**Published:** 2026-01-15

**Authors:** Thomas A. Wadden, Maria A. Oquendo, Robert F. Kushner, Dachuang Cao, Chrisanthi A. Karanikas, Afton Kechter, Madhumita A. Murphy

**Affiliations:** ^1^ Department of Psychiatry, Perelman School of Medicine University of Pennsylvania Philadelphia Pennsylvania USA; ^2^ Division of Endocrinology, Feinberg School of Medicine Northwestern University Chicago Illinois USA; ^3^ Eli Lilly and Company Indianapolis Indiana USA

**Keywords:** clinical trials, C‐SSRS, obesity, PHQ‐9, psychiatric safety, tirzepatide

## Abstract

**Objective:**

This post hoc analysis assessed psychiatric changes with tirzepatide in adults with obesity, without known major psychopathology, from SURMOUNT‐1, SURMOUNT‐2, and SURMOUNT‐3.

**Methods:**

In participants (*N* = 4056) treated with tirzepatide (5/10/15 mg or maximum tolerated dose 10/15 mg) versus placebo, depressive symptoms and suicidal ideation and behavior (SI/SB) were measured using the Patient Health Questionnaire‐9 (PHQ‐9) and Columbia‐Suicide Severity Rating Scale (C‐SSRS), respectively. Nervous system and psychiatric disorder adverse events (AEs) were collected.

**Results:**

Mean (SD) baseline PHQ‐9 scores were 2.7 (3.0) for tirzepatide and 2.6 (3.1) for placebo, indicating no/minimal symptoms of depression. At week 72, scores were 1.9 (2.7) and 2.4 (3.3), respectively (estimated treatment difference [SE]: −0.6 [0.1]); *p* < 0.001. Tirzepatide‐treated participants were less likely to shift to a more severe PHQ‐9 category (18.2% vs. 24.3%; *p* < 0.001). Using the C‐SSRS, 0.6% of participants in each group reported SI, most of which was considered low risk. SB (nonfatal) occurred in 0.1% of tirzepatide‐treated participants versus none with placebo. AEs were generally similar across groups.

**Conclusions:**

In this post hoc analysis, tirzepatide versus placebo did not appear to be associated with an increased risk of depression in participants with overweight/obesity and without known major psychopathology. Rates of SI/SB observed with tirzepatide were similar to those of other incretin‐based therapies. Further study of tirzepatide's safety in persons with significant psychiatric illness may be warranted.

**Trial Registration:**

ClinicalTrials.gov identifiers: NCT04184622, NCT04657003, and NCT04657016

## Study Importance

**What is already known?:**

- Obesity is associated with an increased risk of depression and other psychiatric disorders which health care professionals have been advised to monitor in patients prescribed medications approved for weight management.
- In the SURMOUNT clinical trial program, robust weight reduction was observed with once weekly tirzepatide treatment, a once weekly dual GIP/GLP‐1 receptor agonist, in people with obesity or overweight, with and without type 2 diabetes, compared to placebo.

**What does this study add?:**

- In this post hoc pooled analysis of 4056 participants in SURMOUNT‐1, SURMOUNT‐2, and SURMOUNT‐3, no clinically meaningful differences were observed in mean changes in Patient Health Questionnaire‐9 scores between the pooled tirzepatide (5/10/15 mg) and placebo groups. Reports of overall suicidal ideation (SI), assessed by the Columbia‐Suicide Severity Rating Scale, were similar between groups, although a numerically greater percentage of tirzepatide‐ than placebo‐treated participants reported moderate‐risk SI. Two participants in the tirzepatide group versus none in placebo reported nonfatal suicidal behavior (SB).

**How might these results change the direction of research or the focus of clinical practice?:**

- In this post hoc analysis, tirzepatide relative to placebo was not associated with an increased risk of symptoms of depression. Occurrences of SI/SB were low and similar to those observed in pooled analyses of other incretin‐based therapies approved for chronic weight management.
- The authors support regulatory authorities' recommendations for health care professionals to monitor mood and behavior in patients prescribed incretin‐based therapies for weight management, combined with continued post‐marketing surveillance.

## 1 Introduction

Semaglutide [1, 2] and tirzepatide [3, 4] both slow gastric emptying and enhance neural satiety signaling, resulting in substantial decreases in energy intake [5, 6] and mean reductions in baseline body weight at 68–72 weeks of approximately 15% and 21%, respectively [1, 3]. These reductions are associated with significantly greater improvements than behavioral weight loss interventions in blood pressure, blood glucose, and other measures of cardiometabolic risk [1, 3, 7, 8, 9]. Additional controlled trials have revealed major improvements in cardiovascular outcomes [10, 11], sleep apnea [12], osteoarthritic knee pain [13], and metabolic‐dysfunction‐associated steatohepatitis [14], as well as decreased risk of developing type 2 diabetes (T2D) [15].

Despite these many health benefits, questions remain regarding whether semaglutide, tirzepatide, and other FDA‐approved medications for weight management increase the risk of depression or suicidal thoughts and behaviors [16, 17, 18, 19]. With the exception of orlistat, these drugs access brain regions that regulate energy intake and may interact with mood‐influencing pathways [20]. Concerns about depression are attributable to findings that persons with obesity, particularly with a body mass index (BMI) ≥ 40 kg/m^2^, are at substantially higher risk of major depressive disorder (MDD) than individuals without obesity [21, 22]. Their heightened risk appears related to factors that include pervasive weight stigmatization [23, 24], emotional and physical burdens of obesity‐related comorbidities [25], and chronic inflammation, common to both obesity and depression [26, 27, 28].

In January 2024, after reviewing clinical trial and pharmacovigilance data for glucagon‐like peptide‐1 receptor agonists (GLP‐1RAs), prescribed for obesity or T2D, the FDA concluded that it had “not found evidence that use of these medications causes suicidal thoughts or actions,” a determination similar to that later issued by the European Medicines Agency [18, 19]. Both agencies, however, recommended continued post‐marketing surveillance and careful monitoring in primary care practice of patients' mood and behavior. These generally favorable conclusions were supported by the results of a subsequent post hoc pooled analysis of 3681 participants with obesity treated with placebo versus the GLP‐1RA medication semaglutide 2.4 mg. No clinically meaningful differences were observed between groups in the incidence of significant symptoms of depression or in the small number of cases of suicidal ideation or behavior [29].

The present study reports the results of a pooled post hoc analysis of the psychiatric safety of tirzepatide, a long‐acting agonist of both glucose‐dependent insulinotropic polypeptide (GIP) and GLP‐1 receptors, which was evaluated in the SURMOUNT trials [3, 8, 30]. This is the first report to focus on the psychiatric events associated with the use of a combined GIP/GLP‐1RA, whose dual mechanism of action likely contributes to its larger weight loss as compared with GLP‐1 receptor agonism alone [31].

## 2 Methods

### 2.1 Study Designs and Participants

Trial designs and results for the 72‐week SURMOUNT‐1, SURMOUNT‐2, and SURMOUNT‐3 studies have been published [3, 8, 30]. Online Supporting Information S1 and S2 in this publication provide additional details about the methods and results of the present analyses, while online Supporting Information S3, S4, and S5 provide the trial protocols for the three studies. Participation was open to adults with BMI ≥ 30 kg/m^2^ or ≥ 27 kg/m^2^ with at least one weight‐related comorbidity (SURMOUNT‐2 included participants with T2D). Psychiatric exclusion criteria included a lifetime history of suicide attempt, as well as active (or unstable) MDD or other severe psychiatric illness (e.g., schizophrenia, bipolar disorder) in the past 2 years, as reported to study investigators at screening (see Supporting Information S1). Participants were also excluded if they endorsed moderately severe or greater symptoms of depression on the Patient Health Questionnaire‐9 (PHQ‐9) [32] or clinically significant symptoms of suicidal ideation or behavior on the Columbia Suicide Severity Rating Scale (C‐SSRS) [33, 34]. Participants were randomized to once weekly subcutaneous tirzepatide 5 mg, 10 mg, or 15 mg (1:1:1:1 [SURMOUNT‐1]) or 10 mg or 15 mg (1:1:1 [SURMOUNT‐2]), or the maximum tolerated dose of 10 mg or 15 mg (1:1 [SURMOUNT‐3]), or placebo, as adjunct to diet and physical activity modification.

Trials were conducted in accordance with the International Conference on Harmonization Guidelines for Good Clinical Practice and the Declaration of Helsinki. All participants signed informed consent. Respective protocols were approved by local ethical review boards. Participant retention was high in all treatment groups across the trials (Figure S1).

We note that a subset of participants in SURMOUNT‐1 were selected to have prediabetes, in addition to overweight/obesity, and remained on medication for 176 weeks to determine the effect of tirzepatide in preventing incident T2D [15]. Data from these participants are included in the 72‐week analysis of the three studies, but the full 176‐week data are provided in online Supporting Information S2 as an exploratory assessment of tirzepatide's longer‐term psychiatric safety. Participants in SURMOUNT‐4 were excluded from the present analyses because all study enrollees completed a 36‐week, open‐label tirzepatide lead‐in period before being randomized to placebo or continued tirzepatide [35].

### 2.2 Psychiatric Safety Assessments

Participants were monitored for depression, suicidal ideation (SI) and suicidal behavior (SB), and other psychiatric events through repeated administration of the PHQ‐9, C‐SSRS, and adverse event (AE) reporting, respectively. The PHQ‐9, a well‐validated, self‐report screening tool, assesses the presence and intensity of symptoms of MDD, as defined by the Diagnostic and Statistical Manual of Mental Disorders IV [32]. Using a scale of “0” (not at all) to “3” (nearly every day), respondents rate their experience of nine symptoms over the past 2 weeks [32]. Total scores range from 0 to 27. Scores of 0–4, 5–9, 10–14, 15–19, and ≥ 20 suggest no/minimal, mild, moderate, moderately severe, and severe symptoms of depression, respectively. Scores of ≥ 15 typically trigger referral to a mental health professional. The PHQ‐9 was administered at visit 1 (screening), weeks 0 (baseline), 12, 24, 36, 48, 60, and 72, and at the 4‐week (off‐treatment) safety follow‐up.

The C‐SSRS is a clinician‐administered questionnaire that identifies the occurrence, severity, and frequency of SI/SB over a specified period. It includes suggested questions to elicit the necessary information to determine if SI and/or SB occurred [33, 34]. For the SURMOUNT studies, the C‐SSRS was adapted to assess SI/SB only; sections on the intensity of ideation and lethality of SB were removed. SI types 1–5 were defined as: (1) wish to be dead; (2) nonspecific active suicidal thoughts; (3) active SI with any method (not plan) without intent to act; (4) active SI with some intent to act, without a specific plan; and (5) active SI with a specific plan and intent, respectively. Five types of SB also were assessed, as described later. Study staff administered the C‐SSRS to participants at visit 1 (screening), week 0 (baseline/randomization), and then every 4 weeks through week 72, followed by the 4‐week off‐treatment safety visit. This yielded a total of 18 assessments while participants received study medication, along with the one follow‐up safety assessment. Participants were referred to a mental health professional if the investigator deemed it necessary for the individual's safety, including if the participant had: (1) a PHQ‐9 score ≥ 15 or (2) a response to the C‐SSRS indicative of either type 4 or type 5 SI or any type of SB. This referral policy remained in effect from weeks 78–176 in the 3‐year SURMOUNT‐1 study in people with obesity and prediabetes.

### 2.3 Reporting of Neuropsychiatric AEs

At every study visit, beginning at randomization and continuing every 4 weeks through the safety follow‐up visit, study staff asked participants whether they had experienced any changes in their health since their last visit. Participants' responses were recorded, reviewed by study investigators, and, as appropriate, rated for severity and possible relatedness to study medication. AEs in the system organ classes of psychiatric disorders and nervous system disorders were identified using predefined Medical Dictionary for Regulatory Activities coding (version 26.0).

### 2.4 Statistical Analyses

Data examined included PHQ‐9 scores, the proportion of participants reporting SI/SB on the C‐SSRS, and neuropsychiatric AEs, using all randomized and treated participants including off‐treatment data (safety analysis set). Change in PHQ‐9 total score from baseline to week 72 was examined using ANCOVA with randomized treatment as a factor and baseline value as a covariate. The focus on week 72 was consistent with the assessment of the primary outcome in all three trials (i.e., percentage change in body weight from baseline to week 72), although changes in PHQ‐9 scores were analyzed at additional time points. Categorical shifts from baseline to maximum post‐baseline PHQ‐9 scores were summarized as counts and proportions. Increases and decreases in PHQ‐9 categories were compared between treatment groups using the Cochran Mantel–Haenszel test. The proportion of participants that reached a PHQ‐9 total score ≥ 15, at any time during treatment, was analyzed using logistic regression. Following conventions used in the analysis and publication of data from the first four SURMOUNT trials, the proportions of participants who reported neuropsychiatric AEs were summarized descriptively, without statistical testing, because of the generally low event rates and the absence of a priori hypotheses that were statistically powered. The proportions of participants with SI/SB were also summarized descriptively, without statistical testing, due to their low numbers and the absence of appropriately powered hypotheses.

All analyses were post hoc and not adjusted for multiplicity, with statistical significance defined as *p* < 0.05. Analyses were performed using SAS version 9.4, SAS Institute Inc., Cary, NC, USA.

## 3 Results

### 3.1 Baseline Characteristics

In total, 4056 participants were included in this analysis (tirzepatide: *N* = 2806; placebo: *N* = 1250). Baseline demographics and clinical characteristics were generally similar between groups, with no statistically significant differences on any variables. Participants were predominantly female (2554 [63.0%]) and White (3000 [74.0%]) with a mean duration of obesity of 15.3 years. Common comorbidities at screening included dyslipidemia (1482 [36.5%]) and hypertension (1638 [40.4%]) (Table 1). Overall, 580 (20.7%) participants assigned to tirzepatide and 267 (21.4%) receiving placebo reported a history of psychiatric disorders, and 567 (20.2%) and 286 (22.9%), respectively, reported a history of nervous system disorders (Table S1). At baseline, 244 (8.7%) and 90 (7.2%) participants in the two groups, respectively, reported using antidepressants, psychostimulants, or symptomatic medications for dementia (Table S2).

**TABLE 1:** Participants' baseline demographics and clinical characteristics.

Parameter	Pooled tirzepatide, *N* = 2806	Pooled placebo, *N* = 1250	Total population, *N* = 4056
Age, years	47.1 ± 12.6	47.3 ± 12.6	47.2 ± 12.6
Sex, *n* (%)			
Female	1776 (63.3)	778 (62.2)	2554 (63.0)
Male	1030 (36.7)	472 (37.8)	1502 (37.0)
Race, *n* (%)			
American Indian or Alaska Native	175 (6.2)	62 (5.0)	237 (5.8)
Asian	293 (10.4)	112 (9.0)	405 (10.0)
Black or African American	232 (8.3)	109 (8.7)	341 (8.4)
Multiple	47 (1.7)	14 (1.1)	61 (1.5)
Native Hawaiian or other Pacific Islander	9 (0.3)	3 (0.2)	12 (0.3)
White	2050 (73.1)	950 (76.0)	3000 (74.0)
Ethnicity, *n* (%)			
Hispanic or Latino	1428 (50.9)	659 (52.7)	2087 (51.5)
Not Hispanic or Latino	1210 (43.1)	532 (42.6)	1742 (42.9)
Not reported	168 (6.0)	59 (4.7)	227 (5.6)
Duration of obesity (years)	15.3 ± 11.2	15.2 ± 11.1	15.3 ± 11.1
Weight (kg)	103.5 ± 22.0	103.2 ± 21.5	103.4 ± 21.9
BMI (kg/m^2^)	37.3 ± 6.7	37.2 ± 7.0	37.3 ± 6.8
Waist circumference (cm)	113.7 ± 15.0	113.5 ± 15.3	113.6 ± 15.1
HbA1c (%)	6.08 ± 1.18	6.13 ± 1.20	6.10 ± 1.19
Fasting serum glucose (mg/dL)	109.4 ± 36.0	110.5 ± 37.3	109.8 ± 36.4
Fasting insulin (mIU/L)	13.9 ± 11.1	13.5 ± 11.3	13.8 ± 11.1
Systolic blood pressure (mmHg)	124.8 ± 13.0	124.4 ± 13.1	124.7 ± 13.0
Diastolic blood pressure (mmHg)	79.6 ± 8.3	79.2 ± 8.4	79.5 ± 8.4
Pulse (bpm)	73.0 ± 10.0	72.8 ± 9.8	72.9 ± 9.9
HDL cholesterol (mg/dL)	48.0 ± 12.9	47.5 ± 12.7	47.9 ± 12.8
LDL cholesterol (mg/dL)	110.2 ± 34.1	109.8 ± 33.9	110.1 ± 34.0
Triglycerides (mg/dL)	150.8 ± 113.0	150.9 ± 90.2	150.8 ± 106.5
ALT (IU/L)	27.7 ± 18.1	27.4 ± 17.8	27.6 ± 18.0
UACR, g/kg, median (min, max)	7.0 (0.0, 3978.7)	7.0 (0.7, 1392.0)	7.0 (0.0, 3978.7)
Macroalbuminuria, *n* (%)	53 (1.9)	21 (1.7)	74 (1.8)
Microalbuminuria, *n* (%)	313 (11.2)	140 (11.2)	453 (11.2)
eGFR, mL/min/1.73 m^2^	97.4 ± 17.8	96.7 ± 18.2	97.2 ± 17.9
< 60 mL/min/m^2^, *n* (%)	59 (2.1)	33 (2.6)	92 (2.3)
≥ 60 mL/min/1.73 m^2^, *n* (%)	2747 (97.9)	1217 (97.4)	3964 (97.7)
Prediabetes, *n* (%)^a^	762 (40.2)	270 (42.0)	1032 (40.6)
Dyslipidemia, *n* (%)	1005 (35.8)	477 (38.2)	1482 (36.5)
Hypertension, *n* (%)	1118 (39.8)	520 (41.6)	1638 (40.4)

*Note*: Data are mean ± SD, unless otherwise noted. Data are pooled from SURMOUNT‐1, SURMOUNT‐2 and SURMOUNT‐3.

Abbreviations: ALT, alanine aminotransferase; eGFR, estimated glomerular filtration rate; HbA1c, glycated hemoglobin; HDL, high‐density lipoprotein; LDL, low‐density lipoprotein; UACR, urine albumin‐creatinine ratio.

^a^ The denominator to calculate prediabetes at baseline was tirzepatide: *N* = 1896, placebo: *N* = 643, and total population: *N* = 2539 from the SURMOUNT‐1 trial.

### 3.2 Psychiatric Safety Assessments

#### 3.2.1 Change in PHQ‐9 Total Scores

**FIGURE 1:**
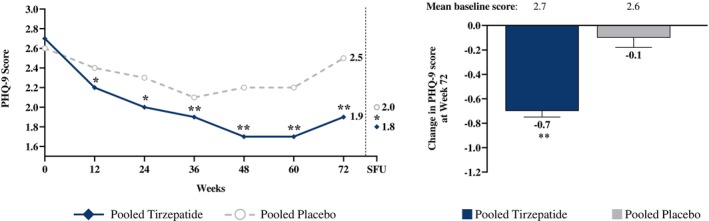
Patient Health Questionnaire‐9 (PHQ‐9) scores over time, including safety follow‐up (SFU) visit, in pooled SURMOUNT‐1, SURMOUNT‐2, and SURMOUNT‐3 trials. Data are LSM (SE) actual values over time through the 4‐week off‐treatment safety follow‐up period and LSM (SE) change from baseline at week 72 from the modified intent‐to‐treat population (safety analysis set). Data are pooled from SURMOUNT‐1, SURMOUNT‐2, and SURMOUNT‐3. The dotted line separates the end of the treatment period from the off‐treatment safety follow‐up. *p < 0.05 and **p < 0.001 versus placebo. SFU = 4‐week off‐treatment safety follow‐up visit. LSM, least squares mean; PHQ‐9, Patient Health Questionnaire 9; SE, standard error.

Mean (SD) PHQ‐9 scores at baseline were 2.7 (3.0) for the tirzepatide group and 2.6 (3.1) for placebo, which at week 72 were 1.9 (2.7) and 2.4 (3.3), respectively (Table 2, Figure 1). Mean estimated treatment difference (SE) between groups for change in PHQ‐9 scores from baseline to week 72 was −0.6 (0.1) (*p* < 0.001) (Table 2). Mean PHQ‐9 scores for both groups remained in the range of no/minimal symptoms of depression at all assessments. However, mean scores declined significantly more from baseline in tirzepatide‐ versus placebo‐treated participants at all time points (Figure 1). Mean values in both groups increased slightly from week 60 to week 72, prior to withdrawal of study medication, but declined in both groups at the off‐treatment safety follow‐up (Figure 1).

**TABLE 2:** Mean PHQ‐9 score by week, change from baseline (Week 0) to Week 72 in PHQ‐9 score, and proportion of participants reaching a PHQ‐9 score ≥ 15 at any time post baseline.

	Pooled tirzepatide, *N* = 2806	Pooled placebo, *N* = 1250
Week 0	2.7 ± 3.04 *n* = 2805	2.6 ± 3.08 *n* = 1249
Week 12	2.2 ± 2.64 *n* = 2733	2.4 ± 2.93 *n* = 1205
Week 24	2.1 ± 2.58 *n* = 2705	2.2 ± 2.80 *n* = 1158
Week 36	1.9 ± 2.51 *n* = 2665	2.1 ± 2.75 *n* = 1111
Week 48	1.8 ± 2.43 *n* = 2610	2.2 ± 2.98 *n* = 1052
Week 60	1.7 ± 2.44 *n* = 2577	2.1 ± 2.95 *n* = 1018
Week 72	1.9 ± 2.71 *n* = 2547	2.4 ± 3.30 *n* = 1012
Safety follow‐up	1.8 ± 2.53 *n* = 1871	2.0 ± 2.64 *n* = 813
Last on‐treatment	1.9 ± 2.62 *n* = 2718	2.4 ± 3.12 *n* = 1197
Last on‐study	1.8 ± 2.62 *n* = 2764	2.1 ± 2.88 *n* = 1219
Change from baseline to week 72, LSM ± SE	−0.7 ± 0.05 *n* = 2546	−0.1 ± 0.08 *n* = 1008
Difference versus placebo, LSM ± SE (*p* value^a^)	−0.6 ± 0.10 (*p* < 0.001)	—
Change from baseline to safety follow‐up, LSM ± SE	−0.8 ± 0.05 *n* = 1870	−0.6 ± 0.08 *n* = 809
Difference versus placebo, LSM ± SE (*p* value^a^)	−0.2 ± 0.10 (*p* = 0.017)	—
Change from baseline to last on‐study, LSM ± SE	−0.8 ± 0.05 *n* = 2763	−0.5 ± 0.07 *n* = 1215
Difference versus placebo, LSM ± SE (*p* value^a^)	−0.3 ± 0.08 (*p* < 0.001)	—
Proportion of participants reaching a score ≥ 15 at any time post baseline^b^, *n* (%)	33 (1.2)	29 (2.3)
Odds ratio versus placebo (*p* value^a^)	0.47 (p = 0.004)	—
Reporting PHQ‐9 item 9 at any time post baseline, *n* (%)	73 (2.6)	42 (3.4)
Odds ratio versus placebo (*p* value^a^)	0.73 (*p* = 0.117)	—

*Note*: Data are mean ± SD PHQ‐9 scores and LSM ± SE change from baseline scores, unless otherwise noted, from the modified intent‐to‐treat population (safety analysis set). Data are pooled from SURMOUNT‐1, SURMOUNT‐2 and SURMOUNT‐3.

Abbreviations: LSM, least squares mean; PHQ‐9, Patient Health Questionnaire 9; SD, standard deviation; SE, standard error.

^a^ *p* value is based on ANCOVA or a logistic model with treatment as a factor and baseline PHQ‐9 total score as a covariate.

^b^ Participants who had both a baseline total score ≥ 15 and a post‐baseline total score ≥ 15 were not included.

In participants who lost < 5% of body weight from baseline to week 72, the mean (SD) end‐of‐treatment change in PHQ‐9 total scores was −0.6 (3.3) in the tirzepatide group versus −0.3 (3.1) for placebo. Data in Figure S2 show the mean changes in PHQ‐9 scores for participants in both treatment groups according to the category of weight reduction achieved (e.g., ≥ 5% to < 10%).

#### 3.2.2 Categorical Shift in PHQ‐9 Scores

Of the 1879 tirzepatide‐treated participants who reported no/minimal symptoms of depression at baseline (score, 0–4), 1492 (79.4%) remained in this category through the safety follow‐up, 319 (17.0%) participants reported mild symptoms (score, 5–9) at one or more assessments, 54 (2.9%) acknowledged moderate symptoms (score, 10–14), 13 (0.7%) reported moderately severe symptoms (score, 15–19), and 1 (0.1%) acknowledged severe symptoms (score, 20–27) (Table 3). A similar but less favorable pattern of scores was observed in the placebo group. Participants treated with tirzepatide versus placebo were less likely to shift to a more severe category of depressive symptoms (18.2% vs. 24.3%; *p* < 0.001; Table 4). By contrast, tirzepatide‐ versus placebo‐treated participants were more likely to shift to a less severe category of depressive symptoms over time (52.4% vs. 41.8%, *p* < 0.001; Table 4).

**TABLE 3:** Categorical shift in PHQ‐9 total score from baseline to safety follow‐up visit.

SURMOUNT‐1, ‐2 and ‐3: Baseline PHQ‐9 category by score	Baseline *n* (%)	PHQ‐9 category at safety follow‐up for pooled tirzepatide (*N* = 2806) or placebo (*N* = 1250)^a^, *n* (%)				
		None (0–4)	Mild (5–9)	Moderate (10–14)	Moderately severe (15–19)	Severe (20–27)
None (0–4)						
Pooled tirzepatide	1879 (67.0)	1492 (79.4)	319 (17.0)	54 (2.9)	13 (0.7)	1 (0.1)
Pooled placebo	880 (70.4)	648 (73.6)	174 (19.8)	47 (5.3)	10 (1.1)	1 (0.1)
Mild (5–9)						
Pooled tirzepatide	667 (23.8)	298 (44.7)	264 (39.6)	96 (14.4)	7 (1.0)	2 (0.3)
Pooled placebo	250 (20.0)	92 (36.8)	108 (43.2)	43 (17.2)	6 (2.4)	1 (0.4)
Moderate (10–14)						
Pooled Tirzepatide	214 (7.6)	73 (34.1)	89 (41.6)	41 (19.2)	8 (3.7)	3 (1.4)
Pooled placebo	82 (6.6)	13 (15.9)	33 (40.2)	24 (29.3)	9 (11.0)	3 (3.7)
Moderately severe (15–19)						
Pooled tirzepatide	2 (0.1)	1 (50.0)	1 (50.0)	0	0	0
Pooled placebo	3 (0.2)	1 (33.3)	1 (33.3)	0	0	1 (33.3)
Severe (20–27)						
Pooled tirzepatide	1 (< 0.1)	0	1 (100.0)	0	0	0
Pooled placebo	0	0	0	0	0	0

*Note*: Data are *n* (%) from the modified intent‐to‐treat population (safety analysis set). Data are pooled from SURMOUNT‐1, SURMOUNT‐2 and SURMOUNT‐3. Categorical shifts in PHQ‐9 total score to “improved,” “stable,” or “not improved” categories are indicated in green, yellow, and red, respectively.

Abbreviation: PHQ‐9, Patient Health Questionnaire 9.

^a^ Missing data for 43 (1.5%) tirzepatide‐treated participants and 35 (2.8%) placebo‐treated participants.

**TABLE 4:** Proportion of participants experiencing an increase or decrease in PHQ‐9 category of depression from baseline to safety follow‐up visit.

Outcome of interest	Pooled tirzepatide, *N* = 2806	Pooled placebo, *N* = 1250
Increase in depression		
Any increase in depression category^a^	503 (18.2)**	295 (24.3)
Increase from no or mild depression to moderate, moderately severe, or severe depression^b^	173 (6.8)*	108 (9.6)
Increase from mild or moderate depression to moderately severe or severe depression^c^	20 (2.3)*	19 (5.7)
Decrease in depression		
Any decrease in depression category^d^	463 (52.4)**	140 (41.8)
Decrease from mild depression to no depression^e^	298 (44.7)*	92 (36.8)
Decrease from moderate depression to mild depression^f^	89 (41.6)	33 (40.2)
Decrease from mild or moderate depression to no depression^g^	371 (42.1)**	105 (31.6)

*Note*: Data are *n* (%) from baseline max to post‐baseline max at safety follow‐up (safety analysis set), from the modified intent‐to‐treat population. The denominator is the number of participants with baseline and ≥ 1 post‐baseline measurement in each category. Data are pooled from SURMOUNT‐1, SURMOUNT‐2 and SURMOUNT‐3. *p* value is based on Cochran Mantel–Haenszel test to adjust for trial.

Abbreviation: PHQ‐9 = Patient Health Questionnaire 9.

^a^ Includes participants in the none, mild, moderate, or moderately severe category during baseline and with ≥ 1 post‐baseline measurement (tirzepatide, *N* = 2762; placebo, *N* = 1215).

^b^ Includes participants in the none or mild depression category during baseline and with ≥ 1 post‐baseline measurement (tirzepatide, *N* = 2546; placebo, *N* = 1130).

^c^ Includes participants in the mild or moderate depression category during baseline and with ≥ 1 post‐baseline measurement (tirzepatide, *N* = 881; placebo, *N* = 332).

^d^ Includes participants in the mild, moderate, moderately severe, or severe category during baseline and with ≥ 1 post‐baseline measurement (tirzepatide, *N* = 884; placebo, *N* = 335).

^e^ Includes participants in the mild depression category during baseline and with ≥ 1 post‐baseline measurement (tirzepatide, *N* = 667; placebo, *N* = 250).

^f^ Includes participants in the moderate depression category during baseline and with ≥ 1 post‐baseline measurement (tirzepatide, *N* = 214; placebo, *N* = 82).

^g^ Includes participants in the mild or moderate depression category during baseline and with ≥ 1 post‐baseline measurement (tirzepatide, *N* = 881; placebo, *N* = 332).

* *p* < 0.05.

** *p* < 0.001 versus placebo.

Examining maximum PHQ‐9 scores occurring at any time during the trial among participants who had a baseline total score < 15, 33 (1.2%) tirzepatide participants and 29 (2.3%) receiving placebo reported a PHQ‐9 score ≥ 15 on one or more occasions, a value suggesting moderately severe or greater symptoms of depression (Table 2). Tirzepatide was associated with a reduced occurrence of this outcome relative to placebo (*p* = 0.004).

#### 3.2.3 SI/SB as Assessed by C‐SSRS

At screening, 70 participants randomized to tirzepatide and 38 assigned to placebo reported having a lifetime history of SI or SB, with 2 participants (tirzepatide 15 mg, 1; placebo, 1) reporting low‐risk SI in the past month. No participants in either pooled group reported SI or SB at baseline (week 0), as assessed by the C‐SSRS.

From randomization through safety follow‐up, 17 of 2793 tirzepatide participants (0.6%, 95% CI [0.1%, 1.0%]) and 7 of 1246 placebo participants (0.6%, 95% CI [0.3%, 0.9%]) reported SI during one or more of the 19 C‐SSRS administrations (Table 5). A majority of these participants in both groups only reported SI judged to be of low risk (i.e., types 1 and 2) because it did not include a method or specific plan of self‐harm or an intention to act (Table 5). Seven of these seventeen tirzepatide participants (equal to 0.3% of the total tirzepatide sample, 95% CI [0.1%, 0.4%]) and one of the seven placebo participants (equal to 0.1% of the total placebo sample, 95% CI [−0.1%, 0.2%]) acknowledged additional SI that was considered of moderate risk (i.e., type 3) or greater. Of these, three tirzepatide participants (i.e., 0.1% of total sample, 95% CI [0.0%, 0.2%]) and one placebo participant (i.e., 0.1% of total sample, 95% CI [−0.1%, 0.2%]) met additional criteria for high‐risk SI (types 4 and 5; Table 5). Table 6 provides detailed information for these 24 participants about the week of SI onset, all types of SI reported, and whether SI resolved at safety follow‐up, which it did in 13 of 17 tirzepatide (data for 3 participants were not available) and 3 of 7 placebo participants.

**TABLE 5:** Proportion of participants with suicidal ideation and behavior as assessed by the Columbia Suicide Severity Rating Scale (C‐SSRS) from baseline to safety follow‐up visit.

Event during treatment	Pooled tirzepatide, *N* = 2793	Pooled placebo, *N* = 1246
Suicidal ideation or behavior (1–10)^a^	17 (0.6)	7 (0.6)
Suicidal ideation (1–5)^b^	17 (0.6)	7 (0.6)
Low risk		
Wish to be dead	16 (0.6)	7 (0.6)
2 Nonspecific active suicidal thoughts	9 (0.3)	3 (0.2)
Moderate risk		
3 Active suicidal ideation with any method (not plan) without intent to act	7 (0.3)	1 (0.1)
High risk		
4 Active suicidal ideation with some intent to act, without specific plan	3 (0.1)	1 (0.1)
5 Active suicidal ideation with specific plan and intent	1 (< 0.1)	0
Suicidal behavior (6–10)^c^	2 (0.1)	0
6 Preparatory acts or behavior	1 (< 0.1)	0
7 Aborted attempt	1 (< 0.1)	0
8 Interrupted attempt	1 (< 0.1)	0
9 Nonfatal suicide attempt	1 (< 0.1)	0
10 Completed suicide	0	0
Self‐injurious behavior without suicidal attempt	0	0

*Note*: Data are number of participants (%) with different types of suicidal ideation and behavior from the modified intent‐to‐treat population (safety analysis set). Data are pooled from SURMOUNT‐1, SURMOUNT‐2 and SURMOUNT‐3.

Abbreviation: C‐SSRS, Columbia‐Suicide Severity Rating Scale.

^a^ Includes participants who experience any one of the ten suicidal ideation events or suicidal behavior events at least once during treatment.

^b^ Includes participants who experience any one of the five suicidal ideation events at least once during treatment.

^c^ Includes participants who experience any one of the five suicidal behavior events at least once during treatment.

**TABLE 6:** Reports of suicidal ideation (SI) and suicidal behavior (SB) obtained with the Columbia Suicide Severity Rating Scale (C‐SSRS) from baseline through safety follow‐up (SFU) visit.

Participant (sex, age)	Trial	Treatment group	SI reported (week)	SI/SB type^a^, ^b^	Worse SI/SB type^a^, ^b^	SI at end of treatment period/SFU (Yes, No)
#1 (F, 45 years)	SURMOUNT‐1	Tirzepatide 5 mg	16	1	1	No/No
#2 (F, 54 years)^c^	SURMOUNT‐1	Tirzepatide 15 mg	32	1	1	No/No
#3 (F, 27 years)^c^	SURMOUNT‐1	Tirzepatide 5 mg	4	1	1	NA/NA
#4 (M, 50 years)	SURMOUNT‐2	Tirzepatide 15 mg	60	1	1	No/No
#5 (F, 65 years)^c^	SURMOUNT‐2	Tirzepatide 10 mg	44	1	1	No/No
#6 (F, 36 years)^c^	SURMOUNT‐3	Tirzepatide MTD	72	1	1	Yes/No
#7 (F, 33 years)	SURMOUNT‐3	Tirzepatide MTD	48	1	1	No/No
#8 (F, 44 years)^c^	SURMOUNT‐1	Tirzepatide 10 mg	60	1	1	No/NA
#9 (F, 50 years)	SURMOUNT‐1	Tirzepatide 10 mg	44	1	1	No/No
			60	2	2	No/No
#10 (M, 31 years)	SURMOUNT‐1	Tirzepatide 15 mg	12	1,2	2	No/No
			16	1,2	2	No/No
#11 (F, 24 years)	SURMOUNT‐3	Tirzepatide MTD	24	2,3	3	No/No
#12 (F, 59 years)	SURMOUNT‐1	Tirzepatide 15 mg	36	1,2,3	3	No/NA
#13 (F, 55 years)	SURMOUNT‐1	Tirzepatide 5 mg	64	1,2,3	3	No/No
#14 (F, 56 years)	SURMOUNT‐1	Tirzepatide 5 mg	68	1,2,3,4	4	No/No
#15 (F, 27 years)^c^	SURMOUNT‐1	Tirzepatide 10 mg	8	1,2,3,4	4	No/Yes
			48	1	1	No/Yes
			SFU	1	1	No/Yes
#16 (F, 30 years)	SURMOUNT‐1	Tirzepatide 15 mg	20	1,2,3,4,5,6,8	5,8	No/No
#17 (F, 22 years)	SURMOUNT‐1	Tirzepatide 10 mg	56	1,2,3,7,9	3,9	No/No
#18 (F, 41 years)^c^	SURMOUNT‐1	Placebo	4	1	1	No/No
#19 (F, 67 years)	SURMOUNT‐1	Placebo	SFU	1	1	No/Yes
#20 (M, 54 years)^c^	SURMOUNT‐1	Placebo	SFU	1	1	NA/Yes
#21 (F, 77 years)^c^	SURMOUNT‐2	Placebo	8	1	1	Yes/Yes
			12	1	1	Yes/Yes
			16	1	1	Yes/Yes
			20	1	1	Yes/Yes
			24	1	1	Yes/Yes
			28	1	1	Yes/Yes
			32	1	1	Yes/Yes
			36	1	1	Yes/Yes
			40	1	1	Yes/Yes
			44	1	1	Yes/Yes
			48	1	1	Yes/Yes
			52	1	1	Yes/Yes
			56	1	1	Yes/Yes
			60	1	1	Yes/Yes
			64	1	1	Yes/Yes
			68	1	1	Yes/Yes
			72	1	1	Yes/Yes
			SFU	1	1	Yes/Yes
#22 (M, 29 years)	SURMOUNT‐1	Placebo	60	1,2	2	No/No
#23 (M, 22 years)^c^	SURMOUNT‐1	Placebo	72	1,2	2	Yes/No
#24 (F, 42 years)	SURMOUNT‐1	Placebo	SFU	1,2,3,4	4	No/Yes

*Note*: No participants reported suicide ideation or behavior at baseline (Week 0).

Abbreviations: MTD, maximum tolerated dose; NA, not available.

^a^ Suicidal ideation types 1–5 using the C‐SSRS were defined as: (1) wish to be dead; (2) nonspecific active suicidal thoughts; (3) active suicidal ideation with any method (not plan) without intent to act; (4) active suicidal ideation with some intent to act, without specific plan; and (5) active suicidal ideation with specific plan and intent. Types 1 and 2 are considered low risk, type 3 is considered moderate risk, and types 4 and 5 are judged high risk.

^b^ Suicidal behavior types 6–10 using the C‐SSRS were defined as (6) preparatory acts or behavior; (7) aborted attempt; (8) interrupted attempt; (9) nonfatal suicide attempt; (10) completed suicide.

^c^ Reported history of suicidal ideation or behavior in participants' lifetime and/or in the month prior to study treatment.

Two participants who received tirzepatide (i.e., 0.1% of total tirzepatide sample, 95% CI [0.0%, 0.2%]) reported SB compared with none assigned to placebo (Table 5). At week 20, one participant (#16 in Table 6), who took sertraline for depression at trial entry, reported an interrupted suicide attempt that resulted in hospitalization, with recovery a week later. She resumed study medication at week 30 and completed the trial. At week 56, a second participant (#17), with a history of anxiety, smoking, and alcohol use, was determined to have experienced an aborted suicide attempt, followed 10 days later by a nonfatal attempt. The site investigator discontinued study medication at the visit, and the participant reported no further SI or SB at week 72 or safety follow‐up.

#### 3.2.4 Suicidal Ideation and Behavior as Assessed by the PHQ‐9

From randomization through the safety follow‐up visit, 73 participants assigned to tirzepatide (2.6%) and 42 assigned to placebo (3.4%) reported on the PHQ‐9 (item 9) “thoughts that you would be better off dead, or hurting yourself in some way.” The difference between groups was not significant (*p* = 0.117) (Table 2).

#### 3.2.5 Neuropsychiatric AE

A total of 444 (15.8%) participants treated with tirzepatide, compared with 163 (13.0%) assigned to placebo, reported at least one treatment‐emergent nervous system disorder AE (Table 7). The greater percentage in the tirzepatide group was due primarily to a numerically higher occurrence of dizziness and dysgeusia in these participants relative to placebo (Table 7; Table S3). The overall reported occurrence of treatment‐emergent psychiatric disorder AEs was similar across groups, except for a numerically lower occurrence of anxiety symptoms with tirzepatide (Table 7; Table S3). The occurrence of depressive disorders and SI was low and numerically comparable across groups (Table S4). AE reporting did not identify any additional cases of SI/SB not captured by the C‐SSRS assessments.

**TABLE 7:** Treatment‐emergent nervous system disorder and psychiatric disorder adverse events.

Adverse events by system organ class and high‐level group term	Pooled tirzepatide, *N* = 2806	Pooled placebo, *N* = 1250
Participants with ≥ 1 treatment‐emergent nervous system disorder adverse event	444 (15.82)	163 (13.04)
Autonomic nervous system disorders	0	1 (0.08)
CNS hemorrhages and cerebrovascular accidents	7 (0.25)	1 (0.08)
CNS vascular disorder NEC	3 (0.11)	0
Cervical spinal cord and nerve root disorders	0	1 (0.08)
Chronic polyneuropathies	2 (0.07)	0
Disturbances in consciousness NEC	33 (1.18)	19 (1.52)
Dyskinesias and movement disorders NEC	1 (0.04)	0
Encephalopathies NEC	1 (0.04)	0
Facial cranial nerve disorders	2 (0.07)	1 (0.08)
Generalized tonic–clonic seizures	1 (0.04)	0
Headaches NEC	193 (6.88)	78 (6.24)
Hydrocephalic conditions	1 (0.04)	0
Increased intracranial pressure disorders	1 (0.04)	0
Lumbar spinal cord and nerve root disorders	25 (0.89)	17 (1.36)
Memory loss (excluding dementia)	3 (0.11)	2 (0.16)
Mental impairment (excluding dementia and memory loss)	3 (0.11)	1 (0.08)
Migraine headaches	27 (0.96)	8 (0.64)
Mononeuropathies	6 (0.21)	4 (0.32)
Multiple sclerosis acute and progressive	1 (0.04)	1 (0.08)
Myelitis (including infective)	0	1 (0.08)
Narcolepsy and hypersomnia	1 (0.04)	0
Neurological signs and symptoms NEC (including dizziness)	148 (5.27)	28 (2.24)
Neuromuscular disorders NEC	3 (0.11)	1 (0.08)
Neuromuscular junction dysfunction	0	1 (0.08)
Olfactory nerve disorders	6 (0.21)	3 (0.24)
Optic nerve disorders NEC	1 (0.04)	0
Paresthesia and dysesthesia	18 (0.64)	10 (0.80)
Peripheral neuropathies NEC	3 (0.11)	1 (0.08)
Seizures and seizure disorders NEC	2 (0.07)	3 (0.24)
Sensory abnormalities NEC (including dysgeusia)	25 (0.89)	5 (0.40)
Sleep disturbances NEC	2 (0.07)	0
Spinal cord and nerve root disorder NEC	1 (0.04)	0
Structural brain disorder NEC	0	1 (0.08)
Transient cerebrovascular events	1 (0.04)	2 (0.16)
Tremor (excluding congenital)	5 (0.18)	1 (0.08)
Trigeminal disorders	1 (0.04)	0
Participants with ≥ 1 treatment‐emergent psychiatric disorder adverse event	191 (6.81)	103 (8.24)
Adjustment disorders	3 (0.11)	1 (0.08)
Anxiety disorders NEC	5 (0.18)	1 (0.08)
Anxiety symptoms	42 (1.50)	44 (3.52)
Attention deficit and disruptive behavior disorders	1 (0.04)	2 (0.16)
Bipolar disorders	0	1 (0.08)
Depressive disorders	41 (1.46)	26 (2.08)
Disturbances in initiating and maintaining sleep	81 (2.89)	29 (2.32)
Eating disorders NEC	1 (0.04)	0
Emotional and mood disturbances NEC	6 (0.21)	1 (0.08)
Fear symptoms and phobic disorders (including social phobia)	1 (0.04)	0
Increased physical activity levels	1 (0.04)	1 (0.08)
Mental disorders NEC	1 (0.04)	0
Mood alterations with depressive symptoms	8 (0.29)	6 (0.48)
Mood alterations with manic symptoms	2 (0.07)	0
Obsessive‐compulsive disorders and symptoms	2 (0.07)	0
Panic attacks and disorders	7 (0.25)	1 (0.08)
Parasomnias	2 (0.07)	0
Psychiatric symptoms NEC	0	1 (0.08)
Sexual desire disorders	12 (0.43)	2 (0.16)
Sleep disorders NEC	4 (0.14)	1 (0.08)
Somatic symptoms disorders	0	1 (0.08)
Stereotypies and automatisms	1 (0.04)	1 (0.08)
Stress disorders	0	1 (0.08)
Substance‐related and addictive disorders	2 (0.07)	0
Suicidal and self‐injurious behavior	3 (0.11)	1 (0.08)

*Note*: Data are *n* (%) from the modified intent‐to‐treat population (safety analysis set). Data are pooled from SURMOUNT‐1, SURMOUNT‐2 and SURMOUNT‐3. Treatment‐emergent adverse events were classed according to Medical Dictionary for Regulatory Activities (version 26.0).

Abbreviations: CNS, central nervous system; NEC, not elsewhere classified.

#### 3.2.6 Longer‐Term Psychiatric Safety in Participants With Prediabetes in SURMOUNT‐1

A total of 1032 participants in the SURMOUNT‐1 trial had prediabetes at baseline, in addition to meeting other study eligibility criteria; 762 were randomized to tirzepatide and 270 to placebo. Following the 72‐week trial, 541 (71.0%) and 136 (50.4%) of these participants, respectively, completed a continuation study (on their original treatment assignment) through week 176, designed to assess the incidence of T2D. Safety outcomes, including depressive symptoms, SI/SB, and psychiatric AEs, continued to be assessed and are reported in online Supporting Information S2. Results of these long‐term assessments generally mirrored findings of the overall 72‐week pooled analysis; however, findings should be considered exploratory.

## 4 Discussion

In this pooled post hoc analysis of 4056 adults in the SURMOUNT‐1, SURMOUNT‐2, and SURMOUNT‐3 studies, tirzepatide was not associated with an increase in significant symptoms of depression relative to placebo. Occurrences of these events were low in both groups. Only 1.2% and 2.3% of participants randomized to tirzepatide and placebo, respectively, reached a score ≥ 15 on the PHQ‐9, a value suggestive of moderately severe or greater symptoms of depression and the need for formal mental health assessment [32]. These percentages are lower than rates of MDD observed in persons with obesity in the general population [22, 36]. At the end of treatment, the mean PHQ‐9 score decreased significantly more with tirzepatide than with placebo, though neither the size of the reduction nor the difference between groups can be considered clinically meaningful, particularly given the low baseline scores in both groups. However, carefully controlled trials of the effects of incretin‐based therapies in persons with overweight/obesity and clinically significant symptoms of depression are currently being conducted [37].

Less than 1% (0.6%) of participants in each group reported incident SI as assessed by the C‐SSRS, a value lower than the 4.3% past‐year prevalence of SI observed in a large representative sample of US adults surveyed in 2015–2019 [38] (our study's psychiatric exclusion criteria likely contribute to the lower rate of SI observed in our participants). Most SI in the present study was transient, absent at safety follow‐up, and considered of low clinical risk because it did not include a method, plan, or intent to engage in self‐harm. A majority of these participants with SI reported wishing to “not wake up,” in association with common life stressors, such as relationship or work problems. Moderate‐ or high‐risk SI was reported by a numerically greater percentage of the tirzepatide group (0.3%) than the placebo participants (0.1%). It was accompanied in one tirzepatide‐treated participant by an interrupted suicide attempt and in a second participant by both an aborted and a nonfatal attempt. Both participants recovered fully. No suicide attempts were reported by placebo‐treated participants, and AE reporting did not identify any cases of SI/SB not detected by the C‐SSRS. Continued surveillance of tirzepatide, as used by increasing numbers of the general population, is warranted because of the very small but numerically greater percentage of tirzepatide‐ versus placebo‐treated participants in the present post hoc analysis who reported moderate‐risk SI or a suicide attempt. The low number of events in this study precludes appropriately powered statistical analyses. Therefore, definitive conclusions cannot be reached about whether the observed imbalance is related to treatment, to patient characteristics (e.g., psychiatric, neurobiological, psychosocial, demographic [39]), their interaction, or to chance alone.

The low occurrence of SI/SB with tirzepatide is similar to that observed in the pooled analysis of 2116 participants treated by semaglutide 2.4 mg in the STEP 1, 2, and 3 trials [29]. As assessed by the C‐SSRS, 0.4% of semaglutide‐treated participants reported SI, and there was one nonfatal suicide attempt; 0.6% of 1260 placebo‐treated participants reported SI, and one nonfatal suicide attempt was recorded. Higher rates of SI/SB could have been expected in the tirzepatide versus semaglutide studies simply because of the greater number of C‐SSRS assessments in the former group, with 19 assessments during study treatment and safety follow‐up in the 72‐week SURMOUNT trials, compared with 5 assessments in the 68‐week STEP investigations. The results for tirzepatide are also consistent with findings from a pooled analysis of four Phase 3a trials of liraglutide 3.0 mg [40], a GLP‐1RA medication approved in 2014 for weight management [41, 42]. The liraglutide trials administered the C‐SSRS on a schedule more comparable to that used with tirzepatide (e.g., 15 assessments over 56 weeks). One percent (1.03%) of participants in both the pooled liraglutide (*N* = 3270) and placebo (*N* = 1832) groups reported SI, which was judged to be of moderate or greater risk in 0.3% and 0.2% of participants in the two groups, respectively [40]. One nonfatal suicide attempt was reported in each group. The past‐year prevalence of suicide attempt in the previously mentioned representative U.S. sample was 0.5% in men and 0.6% in women [38].

SI/SB assessment using item 9 of the PHQ‐9 yielded endorsement rates for both tirzepatide‐ and placebo‐treated participants (2.6% and 3.4%, respectively) that were higher than those obtained with the C‐SSRS. Similarly high endorsement was observed on the PHQ‐9 in the pooled analysis of semaglutide‐ (3.4%) and placebo‐treated (5.0%) participants in the STEP trials [29]. The higher rates with the PHQ‐9 appear to be attributable to patient discomfort in directly disclosing sensitive information to health care professionals, as required with the C‐SSRS. Tirzepatide's generally favorable psychiatric safety, as determined by formal assessment, was supported by the low and balanced distribution between groups of treatment‐emergent AEs including depressive disorders, stress and anxiety symptoms, insomnia, and substance‐use disorders. Similarly, favorable findings were reported from a meta‐analysis of tirzepatide's neuropsychiatric effects, based on 15 randomized trials of participants with T2D, overweight/obesity, and metabolic‐dysfunction‐associated steatohepatitis [43]. The principal imbalance observed was a higher rate of dizziness in tirzepatide‐ versus placebo‐treated participants, as found—along with dysgeusia—in the present pooled analysis. Dizziness and dysgeusia may be linked to orthostatic adaptation from substantial body weight reduction, as well as nutrient deficiencies through loss of appetite. Medical nutritional monitoring and counseling may mitigate these symptoms [44].

Results of the present post hoc analysis, combined with findings of the meta‐analysis [43], suggest that the psychiatric safety of tirzepatide, the only dual GIP/GLP‐1RA approved to date, is comparable to that of GLP‐1RAs. A recent meta‐analysis of 27 randomized controlled trials of GLP‐1RAs, provided for obesity or T2D, concluded that these agents do not appear to increase the low incidence of suicide and self‐harm observed in clinical trial populations [45]. The authors of the analysis, however, consistent with regulatory authorities [18, 19], reiterated the need for continued monitoring to identify particular participants who may be at risk of adverse psychiatric events, as extended use of GLP‐1RAs and other incretin‐based therapies expands in the general population. This is similar to the need for further monitoring and understanding of the low but increased rate of SI and suicide attempts in persons who undergo metabolic and bariatric surgery [45].

The positive assessment of tirzepatide's psychiatric safety is tempered by trial participants having been screened to be free of a lifetime history of suicide attempts and a current PHQ‐9 score > 15, as well as active (or unstable) MDD or other severe psychiatric illnesses in the past 2 years. Thus, the present results cannot establish the psychiatric safety of tirzepatide for weight management when used by individuals with major psychopathology. Additional controlled trials would appear warranted for patients with a variety of appropriately managed psychiatric disorders (e.g., MDD, schizophrenia, bipolar disorder, anxiety) [46], who are assessed using more varied and exhaustive measures than those employed in the present study. This might, for example, include using the investigator‐administered Hamilton Depression Rating Scale [47] in lieu of the PHQ‐9, the latter which is a screening instrument that must be complemented by clinical interview in order to diagnose depression. While awaiting further such studies, large case–control studies (e.g., 50,000 participants), which employed aggregated electronic health records, have provided reassurance about the short‐term psychiatric safety of incretin‐based medications, relative to other drugs, when used for T2D or overweight/obesity by populations likely to have a greater range of psychiatric illnesses than present in controlled clinical trials [48, 49]. This includes the results of a nationwide case‐time‐control study conducted in France. Prior 30‐day use of GLP‐1RAs was not associated with an increased risk of suicide attempts or death by suicide, and the medications presented no specific risk for individuals with obesity or psychiatric disorders [50].

Strengths of this analysis include its large sample of well‐characterized participants in randomized clinical trials who were assessed at frequent intervals using well‐validated measures of depressive symptoms and SI/SB. Limitations include the previously discussed exclusion of people with severe psychiatric illness, as well as the increased attrition rate at week 176 in the 3‐year SURMOUNT‐1 study in people with obesity and prediabetes, which limits the interpretation of the longer‐term psychiatric safety observed with tirzepatide.

Given the high risk of depression and anxiety in people with obesity—particularly with BMI ≥ 40 kg/m^2^—we support ongoing monitoring of mental health in all such individuals, whether or not engaged in weight management, so that they may receive psychiatric care as needed. We similarly support the FDA's current recommendations to avoid the use of tirzepatide (and other obesity medications) for weight management in persons with a history of suicide attempt or active SI [4].

In conclusion, this post hoc analysis found that treatment with once weekly tirzepatide was not associated with an increased risk of developing symptoms of clinically significant depression in people with overweight or obesity, with or without T2D, and without known major psychopathology. Tirzepatide was associated with low rates of SI and SB, similar to those reported in comparable pooled analyses of the GLP‐1RAs liraglutide 3.0 mg and semaglutide 2.4 mg [29, 40].

## Author Contributions

T.A.W. and C.A.K. wrote the manuscript. D.C. was responsible for the statistical analyses. M.A.M. is the guarantor of this work and, as such, takes responsibility for the integrity of the data and the accuracy of the data analysis. All authors participated in the interpretation of data and critical review of the manuscript, had full access to all data in the study, and approved this manuscript to be submitted for publication.

## Funding

Funding for this post hoc analysis was provided by Eli Lilly and Company.

## Conflicts of Interest

T.A.W. reports serving on advisory boards for Novo Nordisk and Weight Watchers and receiving grants, on behalf of the University of Pennsylvania, from Eli Lilly and Company, Epitomee Medical, and Novo Nordisk. M.A.O. reports receiving royalties from the Research Foundation for Mental Hygiene for commercial use of the Columbia Suicide Severity Rating Scale, volunteers as a scientific advisor to Mind Medicine, reviews grants for Alkermes, is a Trustee of Tufts University, and advises St. George's University and Fundacion Jimenez Diaz. R.F.K. reports financial compensation as a consultant or member of the medical advisory board for Novo Nordisk, Weight Watchers, Eli Lilly and Company, Structure, Boehringer Ingelheim, Altimmune, Regeneron, Antag, Currax, and AstraZeneca. D.C., C.A.K., A.K., and M.A.M. are employees and shareholders of Eli Lilly and Company.

## Supporting information


**Supplement S1:** Additional data on psychiatric safety from baseline through the 72‐week treatment period and 4‐week safety follow‐up period for the pooled analysis of the SURMOUNT‐1, SURMOUNT‐2 and SURMOUNT‐3 trials.

**Supplement S2:** Data on long‐term changes (Weeks 0 to 176 and safety follow‐up visit at week 193) in psychiatric safety in a subset of participants in SURMOUNT‐1 who had prediabetes at baseline and received study medication for 176 weeks to assess the effects of tirzepatide, compared with placebo, on the incidence of type 2 diabetes.

**Supplement S3:** Full study protocol for SURMOUNT‐1.

**Supplement S4:** Full study protocol for SURMOUNT‐2.

**Supplement S5:** Full study protocol for SURMOUNT‐3.

## Data Availability

Lilly provides access to all individual participant data collected during the trial, after anonymization, with the exception of pharmacokinetic or genetic data. Data are available to request 6 months after the indication studied has been approved in the US and EU and after primary publication acceptance, whichever is later. No expiration date of data requests is currently set once data are made available. Access is provided after a proposal has been approved by an independent review committee identified for this purpose and after receipt of a signed data sharing agreement. Data and documents, including the study protocol, statistical analysis plan, clinical study report, and blank or annotated case report forms, will be provided in a secure data sharing environment. For details on submitting a request, see the instructions provided at https://vivli.org/.
