# Supplementary material for: Psychiatric Safety of Tirzepatide in People With Obesity and No Known Major Psychopathology: A Post Hoc Analysis of SURMOUNT
Source: Obesity (Silver Spring). 2026 Jan 15;34(3):565–78. doi: 10.1002/oby.70122 (PMC12933222; doi:10.1002/oby.70122)

SUPPLEMENT 1

Psychiatric Safety of Tirzepatide in People with Obesity and No Known Major Psychopathology: A Pooled Post Hoc Analysis of the SURMOUNT-1, SURMOUNT-2, and SURMOUNT-3 Trials

Thomas A. Wadden, PhD, Maria A. Oquendo, MD, PhD, Robert F. Kushner, MD, Dachuang Cao, PhD, MS, Chrisanthi A. Karanikas, MS, Afton Kechter, PhD, Madhumita A. Murphy, MD, MS, MBA

This supplement provides data on: Psychiatric safety data throughout the 72-week treatment period and 4-week safety follow-up period of the SURMOUNT-1, SURMOUNT-2 and SURMOUNT-3 trials.

CONTENTS

eMETHODS

Exclusion Criteria Related to Psychiatric Illness…………………………………………......2

SUPPLEMENTAL TABLES

Table S1. Participants' Medical History of Psychiatric and Nervous System Disorders.………..…3

Table S2. Participants' Use at Baseline of Antidepressant and Psychostimulant Medications, and Symptomatic Medications for Dementia……..………..……………………..……………….4

Table S3. Treatment-emergent Nervous System and Psychiatric Disorder Adverse Events……...5

Table S4. Treatment-emergent Major Depressive Disorder/Suicidal Ideation Events ………..…..9

SUPPLEMENTAL FIGURES

Supplemental Figure Legends………………………………………………………………10

Figure S1. Participant Disposition in SURMOUNT-1, SURMOUNT-2 and SURMOUNT-3…...11

Figure S2. Last On-study PHQ-9 Score by Percent Body Weight Reduction Threshold…………12

eMETHODS

Exclusion Criteria Related to Psychiatric Illness

Exclusion criteria related to psychiatric illness included any lifetime history of a suicide attempt, as well as a history of significant active or unstable major depressive disorder or other severe psychiatric illness (e.g., schizophrenia, bi-polar disorder) within the last 2 years. Severe psychiatric illness was defined as, "a diagnosable mental, behavioral, or emotional disorder of sufficient duration to meet diagnostic criteria in the DSM, which results in functional impairment that substantially interferes with or limits one or more major life activities.[1]" Participants also were excluded, if at Visit 1 (screening) or Visit 3 (prior to randomization), they scored 15 or higher on the Patient Health Questionnaire-9 (PHQ-9), values suggestive of moderately severe or greater symptoms of depression. They similarly were excluded if they acknowledged experiencing: active suicidal ideation with some intent to act, without a specific plan; active suicidal ideation with a specific plan and intent to act: and an actual attempt, interrupted attempt, aborted attempt, or preparatory act or behavior, as determined by the Columbia–Suicide Severity Rating Scale (C-SSRS), or if the ideation or behavior occurred within the past month prior to randomization. Participants with major depressive disorder or generalized anxiety disorder whose disease state, in the opinion of the investigator, was considered stable and expected to remain stable throughout the course of the study were considered for inclusion if they were not on excluded medications (i.e., medications that may cause weight gain including but not limited to tricyclic antidepressants, atypical antipsychotics, and certain mood stabilizers).

1. Insel TR. Assessing the economic costs of serious mental illness. Am J Psychiatry. 2008;165(6):663-665.

Table S1. Participants' Medical History of Psychiatric and Nervous System Disorders

Characteristic	Pooled Tirzepatide N=2806	Pooled Placebo N=1250	Total N=4056
**Psychiatric disorders[a]**	**580 (20.67)**	**267 (21.36)**	**847 (20.88)**
Anxiety	248 (8.84)	108 (8.64)	356 (8.78)
Depression	222 (7.91)	103 (8.24)	325 (8.01)
Insomnia	184 (6.56)	72 (5.76)	256 (6.31)
Attention deficit hyperactivity disorder	37 (1.32)	15 (1.20)	52 (1.28)
**Nervous system disorder[a]**	**567 (20.21)**	**286 (22.88)**	**853 (21.03)**
Migraine	157 (5.60)	75 (6.00)	232 (5.72)
Headache	141 (5.02)	62 (4.96)	203 (5.00)
Carpal tunnel syndrome	63 (2.25)	33 (2.64)	96 (2.37)
Diabetic neuropathy	37 (1.32)	26 (2.08)	63 (1.55)
Neuropathy peripheral	40 (1.43)	12 (0.96)	52 (1.28)
Tension headache	33 (1.18)	14 (1.12)	47 (1.16)
Sciatica	25 (0.89)	16 (1.28)	41 (1.01)

Data are n (%) from the modified intent-to-treat population (safety analysis set). Data are pooled from SURMOUNT-1, SURMOUNT-2 and SURMOUNT-3. Medical history of disorders were classed according to MedDRA (version 26.0) and organized by system organ class and preferred term.

[a] Occurring in at least 1% of participants from the total population.

Abbreviations: MedDRA=Medical Dictionary for Regulatory Activities.

Table S2. Participants' Use at Baseline of Antidepressant and Psychostimulant Medications, and Symptomatic Medications for Dementia Medication

Medication	Pooled Tirzepatide N=2806	Pooled Placebo N=1250	Total N=4056
**Antidepressant, psychostimulant, and anti-dementia medication**	**244 (8.70)**	**90 (7.20)**	**334 (8.23)**
Selective Serotonin Reuptake Inhibitors	120 (4.28)	49 (3.92)	169 (4.17)
Other Antidepressants	125 (4.45)	43 (3.44)	168 (4.14)
Centrally Acting Sympathomimetics	28 (1.00)	14 (1.12)	42 (1.04)
Non-Selective Monoamine Reuptake Inhibitors	3 (0.11)	1 (0.08)	4 (0.10)
Other Psychostimulants And Nootropics	3 (0.11)	1 (0.08)	4 (0.10)
Xanthine Derivatives	2 (0.07)	0	2 (0.05)
Anticholinesterases	0	1 (0.08)	1 (0.02)
Other Symptomatic Medications for Dementia	1 (0.04)	0	1 (0.02)

Data are n (%) from the modified intent-to-treat population (safety analysis set). Data are pooled from SURMOUNT-1, SURMOUNT-2 and SURMOUNT-3.

Table S3. Treatment-emergent Nervous System and Psychiatric Disorder Adverse Events

**High-level group term**<br>Preferred term	**Pooled Tirzepatide N=2806**	**Pooled Placebo N=1250**
**Participants with ≥1 treatment-emergent nervous system disorder event**	**444 (15.82)**	**163 (13.04)**
**Autonomic nervous system disorders**	0	1 (0.08)
Autonomic nervous system imbalance	0	1 (0.08)
**CNS hemorrhages and cerebrovascular accidents**	7 (0.25)	1 (0.08)
Cerebrovascular accident	1 (0.04)	0
Hemorrhagic stroke	1 (0.04)	0
Ischemic stroke	3 (0.11)	1 (0.08)
Thalamus hemorrhage	1 (0.04)	0
Vertebrobasilar stroke	1 (0.04)	0
**CNS vascular disorder NEC**	3 (0.11)	0
Carotid arteriosclerosis	2 (0.07)	0
Carotid artery disease	1 (0.04)	0
**Cervical spinal cord and nerve root disorders**	0	1 (0.08)
Cervical radiculopathy	0	1 (0.08)
**Chronic polyneuropathies**	2 (0.07)	0
Diabetic neuropathy	2 (0.07)	0
**Disturbances in consciousness NEC**	33 (1.18)	19 (1.52)
Lethargy	4 (0.14)	0
Loss of consciousness	1 (0.04)	1 (0.08)
Somnolence	12 (0.43)	6 (0.48)
Syncope	16 (0.57)	13 (1.04)
**Dyskinesias and movement disorders NEC**	1 (0.04)	0
Psychomotor hyperactivity	1 (0.04)	0
**Encephalopathies NEC**	1 (0.04)	0
Encephalopathy	1 (0.04)	0
**Facial cranial nerve disorders**	2 (0.07)	1 (0.08)
Bell's palsy	1 (0.04)	1 (0.08)
Facial paralysis	1 (0.04)	0
**Generalized tonic-clonic seizures**	1 (0.04)	0
Generalized tonic-clonic seizures	1 (0.04)	0
**Headaches NEC**	193 (6.88)	78 (6.24)
Headache	183 (6.52)	73 (5.84)
Post-traumatic headache	0	1 (0.08)
Sinus headache	0	2 (0.16)
Tension headache	10 (0.36)	3 (0.24)
Vascular headache	0	1 (0.08)
**Hydrocephalic conditions**	1 (0.04)	0
External hydrocephalus	1 (0.04)	0
Hydrocephalus	1 (0.04)	0
**Increased intracranial pressure disorders**	1 (0.04)	0
Brain edema	1 (0.04)	0
**Lumbar spinal cord and nerve root disorders**	25 (0.89)	17 (1.36)
Lumbar radiculopathy	5 (0.18)	2 (0.16)

Sciatica	21 (0.75)	15 (1.20)
**Memory loss (excluding dementia)**	3 (0.11)	2 (0.16)
Amnesia	2 (0.07)	1 (0.08)
Memory impairment	1 (0.04)	1 (0.08)
**Mental impairment (excluding dementia and memory loss)**	3 (0.11)	1 (0.08)
Disturbance in attention	3 (0.11)	1 (0.08)
**Migraine headaches**	27 (0.96)	8 (0.64)
Migraine	27 (0.96)	7 (0.56)
Ophthalmic migraine	0	1 (0.08)
**Mononeuropathies**	6 (0.21)	4 (0.32)
Carpel tunnel syndrome	0	3 (0.24)
Nerve compression	3 (0.11)	1 (0.08)
Peroneal nerve palsy	3 (0.11)	0
**Multiple sclerosis acute and progressive**	1 (0.04)	1 (0.08)
Multiple sclerosis	1 (0.04)	1 (0.08)
Multiple sclerosis relapse	1 (0.04)	0
**Myelitis (including infective)**	0	1 (0.08)
Myelitis transverse	0	1 (0.08)
**Narcolepsy and hypersomnia**	1 (0.04)	0
Hypersomnia	1 (0.04)	0
**Neurological signs and symptoms NEC**	148 (5.27)	28 (2.24)
Brain fog	1 (0.04)	0
Dizziness	132 (4.70)	26 (2.08)
Dizziness postural	7 (0.25)	1 (0.08)
Presyncope	9 (0.32)	1 (0.08)
**Neuromuscular disorders NEC**	3 (0.11)	1 (0.08)
Muscle contractions involuntary	3 (0.11)	1 (0.08)
**Neuromuscular junction dysfunction**	0	1 (0.08)
Myasthenia gravis	0	1 (0.08)
**Olfactory nerve disorders**	6 (0.21)	3 (0.24)
Anosmia	4 (0.14)	3 (0.24)
Hyposmia	1 (0.04)	0
Parosmia	1 (0.04)	0
**Optic nerve disorders NEC**	1 (0.04)	0
Optic neuritis	1 (0.04)	0
**Paresthesia and dysesthesia**	18 (0.64)	10 (0.80)
Burning sensation	1 (0.04)	0
Hyperesthesia	1 (0.04)	0
Hypoesthesia	8 (0.29)	6 (0.48)
Paresthesia	8 (0.29)	4 (0.32)
**Peripheral neuropathies NEC**	3 (0.11)	1 (0.08)
Brachial plexopathy	1 (0.04)	0
Neuropathy peripheral	2 (0.07)	1 (0.08)
**Seizures and seizure disorders NEC**	2 (0.07)	3 (0.24)
Epilepsy	1 (0.04)	2 (0.16)
Idiopathic generalized epilepsy	0	1 (0.08)
Seizure	1 (0.04)	1 (0.08)
**Sensory abnormalities NEC**	25 (0.89)	5 (0.4)
Ageusia	1 (0.04)	1 (0.08)

Allodynia	1 (0.04)	0
Dysgeusia	12 (0.43)	0
Neuralgia	6 (0.21)	1 (0.08)
Post herpetic neuralgia	1 (0.04)	1 (0.08)
Restless leg syndrome	0	1 (0.08)
Taste disorder	4 (0.14)	2 (0.16)
**Sleep disturbances NEC**	2 (0.07)	0
Periodic limb movement disorders	1 (0.04)	0
Sleep deficit	1 (0.04)	0
**Spinal cord and nerve root disorder NEC**	1 (0.04)	0
Radiculopathy	1 (0.04)	0
**Structural brain disorder NEC**	0	1 (0.08)
White matter lesion	0	1 (0.08)
**Transient cerebrovascular events**	1 (0.04)	2 (0.16)
Transient ischemic attack	1 (0.04)	2 (0.16)
**Tremor (excluding congenital)**	5 (0.18)	1 (0.08)
Resting tremor	1 (0.04)	0
Tremor	4 (0.14)	1 (0.08)
**Trigeminal disorders**	1 (0.04)	0
Trigeminal neuralgia	1 (0.04)	0
**Participants with ≥1 treatment-emergent psychiatric disorder event**	**191 (6.81)**	**103 (8.24)**
**Adjustment disorders**	3 (0.11)	1 (0.08)
Adjustment disorder with depressed mood	1 (0.04)	0
Adjustment disorder with mixed anxiety and depression mood	1 (0.04)	0
Grief reaction	1 (0.04)	1 (0.08)
**Anxiety disorders NEC**	5 (0.18)	1 (0.08)
Anxiety disorder	4 (0.14)	0
Generalized anxiety disorder	1 (0.04)	1 (0.08)
**Anxiety symptoms**	42 (1.50)	44 (3.52)
Anxiety	40 (1.43)	44 (3.52)
Stress	3 (0.11)	0
**Attention deficit and disruptive behavior disorders**	1 (0.04)	2 (0.16)
Attention deficit hyperactivity disorder	1 (0.04)	2 (0.16)
**Bipolar disorders**	0	1 (0.08)
Bipolar disorder	0	1 (0.08)
**Depressive disorders**	41 (1.46)	26 (2.08)
Depression	37 (1.32)	23 (1.84)
Major depression	4 (0.14)	3 (0.24)
**Disturbances in initiating and maintaining sleep**	81 (2.89)	29 (2.32)
Initial insomnia	4 (0.14)	0
Insomnia	78 (2.78)	29 (2.32)
**Eating disorders NEC**	1 (0.04)	0
Bulimia nervosa	1 (0.04)	0
**Emotional and mood disturbances NEC**	6 (0.21)	1 (0.08)
Discouragement	3 (0.11)	0
Irritability	3 (0.11)	1 (0.08)
**Fear symptoms and phobic disorders (including social phobia)**	1 (0.04)	0
Claustrophobia	1 (0.04)	0

**Increased physical activity levels**	1 (0.04)	1 (0.08)
Restlessness	1 (0.04)	1 (0.08)
**Mental disorders NEC**	1 (0.04)	0
Mental status changes	1 (0.04)	0
**Mood alterations with depressive symptoms**	8 (0.29)	6 (0.48)
Depressed mood	8 (0.29)	6 (0.48)
**Mood alterations with manic symptoms**	2 (0.07)	0
Mania	2 (0.07)	0
**Obsessive-compulsive disorders and symptoms**	2 (0.07)	0
Body dysmorphic disorder	1 (0.04)	0
Dermatillomania	1 (0.04)	0
**Panic attacks and disorders**	7 (0.25)	1 (0.08)
Panic attack	2 (0.07)	1 (0.08)
Panic disorder	5 (0.18)	0
**Parasomnias**	2 (0.07)	0
Nightmare	2 (0.07)	0
**Psychiatric symptoms NEC**	0	1 (0.08)
Abulia	0	1 (0.08)
**Sexual desire disorders**	12 (0.43)	2 (0.16)
Libido decreased	12 (0.43)	2 (0.16)
**Sleep disorders NEC**	4 (0.14)	1 (0.08)
Sleep disorder	4 (0.14)	1 (0.08)
**Somatic symptoms disorders**	0	1 (0.08)
Conversion disorder	0	1 (0.08)
**Stereotypies and automatisms**	1 (0.04)	1 (0.08)
Bruxism	1 (0.04)	1 (0.08)
**Stress disorders**	0	1 (0.08)
Post-traumatic stress disorder	0	1 (0.08)
**Substance related and addictive disorders**	2 (0.07)	0
Nicotine dependence	1 (0.04)	0
Substance abuse	1 (0.04)	0
**Suicidal and self-injurious behavior**	3 (0.11)	1 (0.08)
Suicidal ideation	1 (0.04)	1 (0.08)
Suicidal attempt	2 (0.07)	0

Data are n (%) from the modified intent-to-treat population (safety analysis set). Data are pooled from SURMOUNT-1, SURMOUNT-2 and SURMOUNT-3. Note: Treatment-emergent nervous system disorder and psychiatric disorder adverse events were classed according to MedDRA (version 26.0) and organized by high-level group term and preferred term.

Abbreviations: CNS=central nervous system; MedDRA=Medical Dictionary for Regulatory Activities; NEC=not elsewhere classified.

Table S4. Treatment-emergent Major Depressive Disorder/Suicidal Ideation Events

Event category or term	Pooled Tirzepatide N=2806	Pooled Placebo N=1250
**Participants with TEAE of MDD/SI events**	**55 (2.0)**	**33 (2.6)**
**Depression (excluding suicide and self-injury) (SMQ)**	53 (1.9)	33 (2.6)
Depression	37 (1.3)	23 (1.8)
Depressed mood	8 (0.3)	6 (0.5)
Major depression	4 (0.1)	3 (0.2)
Discouragement	3 (0.1)	0
Adjustment disorder with depressed mood	1 (<0.1)	0
Adjustment disorder with mixed anxiety and depressed mood	1 (<0.1)	0
Depression rating scale score increased	0	1 (0.1)
**Suicide/self-injury (SMQ)**	3 (0.1)	1 (0.1)
Suicide attempt	2 (0.1)	0
Suicidal ideation	1 (<0.1)	1 (0.1)
**Participants with TEAE of severe or serious MDD/SI events**	**6 (0.2)**	**1 (0.1)**
**Depression (excluding suicide and self-injury) (SMQ)**	4 (0.1)	1 (0.1)
Depression	1 (<0.1)	1 (0.1)
Major depression	2 (0.1)	0
Adjustment disorder with mixed anxiety and depressed mood	1 (<0.1)	0
**Suicide/self-injury (SMQ)**	3 (0.1)	0
Suicide attempt	2 (0.1)	0
Suicidal ideation	1 (<0.1)	0

Data are n (%) from the modified intent-to-treat population (safety analysis set). Data are pooled from SURMOUNT-1, SURMOUNT-2 and SURMOUNT-3. Note: Treatment-emergent major depressive/suicidal ideation events were classed according to MedDRA (version 26.0).

Abbreviations: MDD=major depressive disorder; MedDRA=Medical Dictionary for Regulatory Activities; SI=suicidal ideation; SMQ=standardized MedDRA query; TEAE=treatment-emergent adverse event.

Supplemental Figure Legends

Figure S1. Participant Disposition in SURMOUNT-1, SURMOUNT-2 and SURMOUNT-3

Figure S2. Last On-study PHQ-9 Score by Percent Body Weight Reduction Threshold Data are mean change from baseline in PHQ-9 total score at week 72 (primary endpoint) by body weight reduction threshold (safety analysis set). The <5% subgroup included participants who gained weight. Abbreviations: PHQ-9=Patient Health Questionnaire 9.

Figure S1. Participant Disposition in SURMOUNT-1, SURMOUNT-2 and SURMOUNT-3

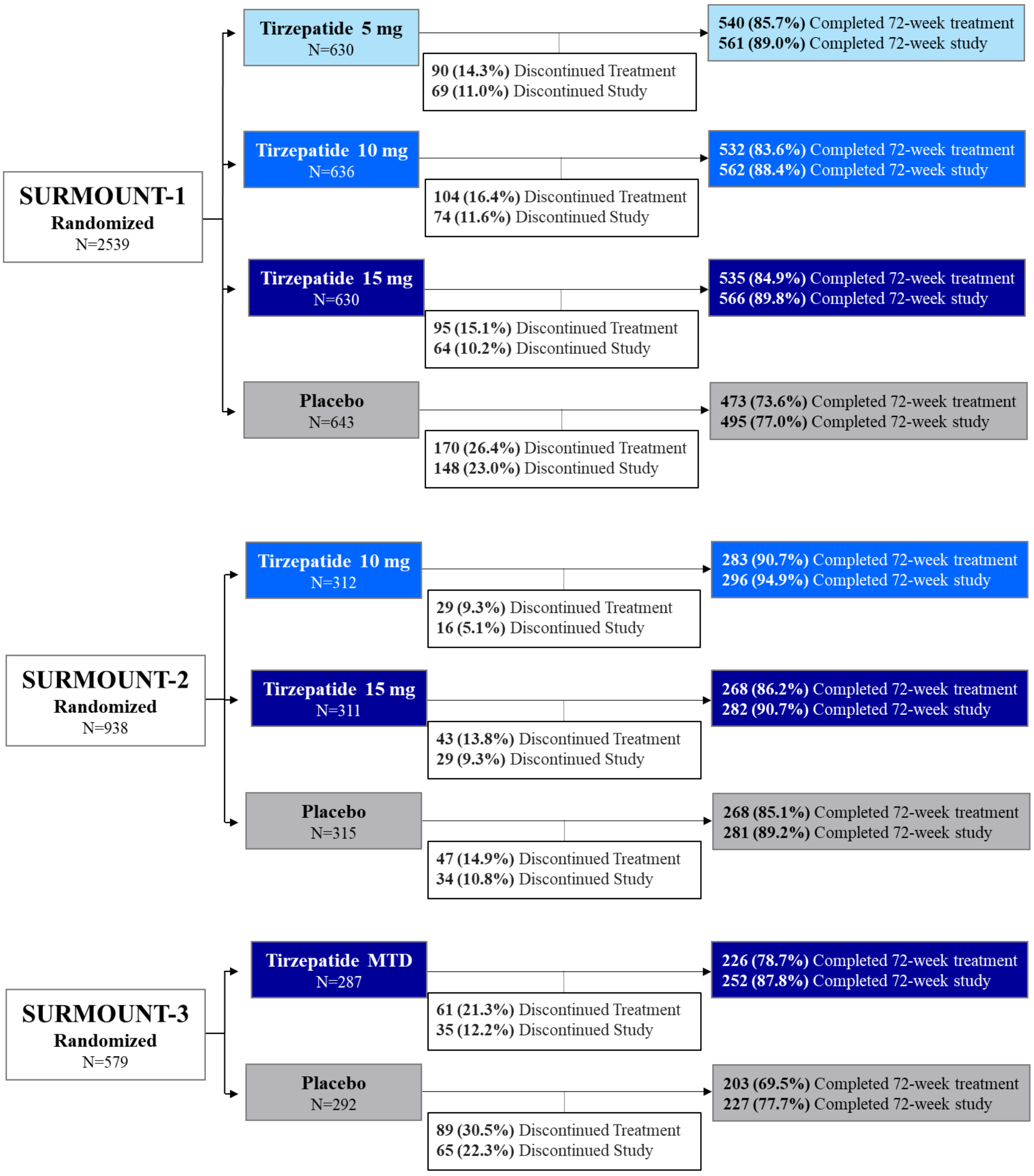

Figure S2. Last On-study PHQ-9 Score by Percent Body Weight Reduction Threshold

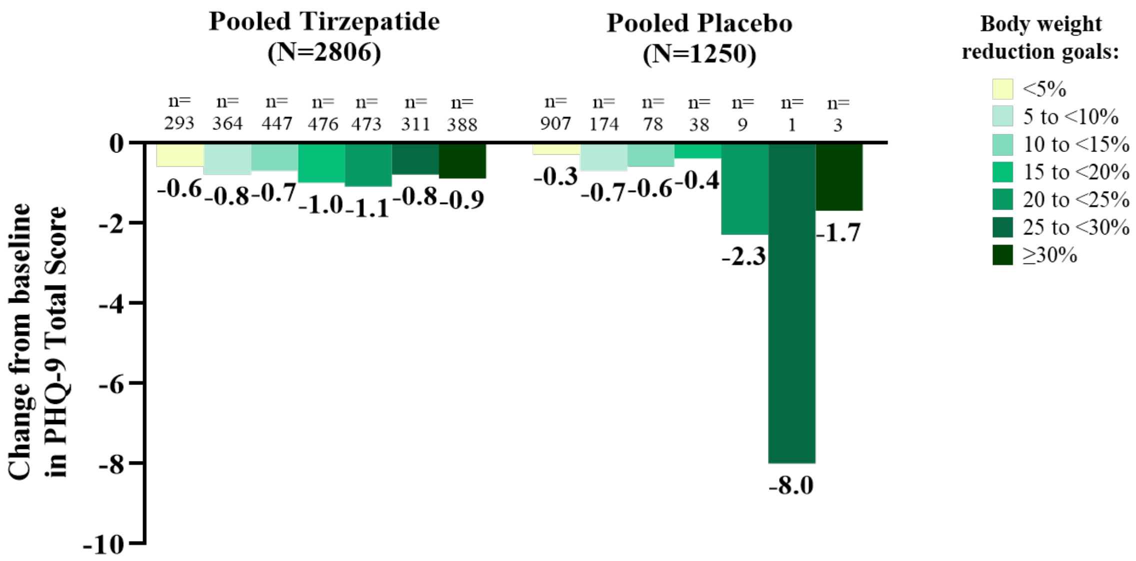

Supplement: Supplementary file 1 — Supplement S1: Additional data on psychiatric safety from baseline through the 72‐week treatment period and 4‐week safety follow‐up period for the pooled analysis of the SURMOUNT‐1, SURMOUNT‐2 and SURMOUNT‐3 trials. [file OBY-34-565-s004.pdf]
