# Supplementary material for: Psychiatric Safety of Tirzepatide in People With Obesity and No Known Major Psychopathology: A Post Hoc Analysis of SURMOUNT
Source: Obesity (Silver Spring). 2026 Jan 15;34(3):565–78. doi: 10.1002/oby.70122 (PMC12933222; doi:10.1002/oby.70122)

SUPPLEMENT 2

Psychiatric Safety of Tirzepatide in People with Obesity and No Known Major Psychopathology: A Pooled Post Hoc Analysis of the SURMOUNT-1, SURMOUNT-2, and SURMOUNT-3 Trials

Thomas A. Wadden, PhD, Maria A. Oquendo, MD, PhD, Robert F. Kushner, MD, Dachuang Cao, PhD, MS, Chrisanthi A. Karanikas, MS, Afton Kechter, PhD, Madhumita A. Murphy, MD, MS, MBA

This supplement provides data on: Long-term changes (Weeks 72-176) on the PHQ-9 and C-SSRS in a subset of participants with obesity and prediabetes at baseline who participated in a 3-year SURMOUNT-1 study (through Week 176 including safety follow-up [Week 193]).

CONTENTS

Overview of the 3-year SURMOUNT-1 Study in People with Obesity and Prediabetes…………..3

SUPPLEMENTAL TABLES

Table S1. Mean PHQ-9 Score by Week, Change from Baseline in PHQ-9 Score, and Proportion of Participants Reaching a PHQ-9 Total Score ≥15 Anytime Postbaseline in the 3-year SURMOUNT-1 Study in People with Obesity and Prediabetes..…………………………………5

Table S2. Categorical Shift in PHQ-9 Total Score from Baseline to Safety Follow-up in the 3-year SURMOUNT-1 Study in People with Obesity and Prediabetes………...………………..……..……7

Table S3. Increase/Decrease in Depression Based on PHQ-9 Score from Baseline to Week 176 and at Safety Follow-up in the 3-year SURMOUNT-1 Study in People with Obesity and Prediabetes……………………………………………………………………………………...8

Table S4. Proportion of Participants with Suicidal Ideation and Behavior as Assessed by the Columbia Suicide Severity Rating Scale (C-SSRS) from Baseline to Safety Follow-up Visit in People with Obesity and Prediabetes……………..……………………………………..…..…….…9

Table S5. Suicidal Ideation and Behavior as Assessed by the Columbia Suicide Severity Rating Scale (C-SSRS) Through Safety Follow-up (SFU) in the 3-year SURMOUNT-1 Study in People with Obesity and Prediabetes…………………….…………………………….………………...…...10

Table S6. Treatment-emergent Nervous System and Psychiatric Disorder Adverse Events in the 3-year SURMOUNT-1 Study in People with Obesity and Prediabetes…………………………..11

Table S7. Treatment-emergent Major Depressive Disorder/Suicidal Ideation Events in the 3-year SURMOUNT-1 Study in People with Obesity and Prediabetes…………………………………14

SUPPLEMENTAL FIGURES

Supplemental Figure Legends……………………………………………………………………15

Figure S1. Patient Health Questionnaire 9 (PHQ-9) Scores Over Time, Including the Safety Follow-up (SFU) Visit, in the 3-year SURMOUNT-1 Study in People with Obesity and Prediabetes………………………………………………………………………………………16

Figure S2. Last On-study PHQ-9 Score by Percent Body Weight Reduction Threshold the 3-year SURMOUNT-1 Study in People with Obesity and Prediabetes…………………………………17

Overview of the 3-year SURMOUNT-1 Study in People with Obesity and Prediabetes

In SURMOUNT-1, a total of 2,539 participants with obesity, of whom 1,032 also had prediabetes, were randomized in a 1:1:1:1 ratio to receive placebo or once weekly doses of tirzepatide at 5 mg, 10 mg, or 15 mg. At the conclusion of the main trial at week 72, treatment was discontinued in participants who had been free of prediabetes at study enrollment. The results of the primary trial were published based on the results for the 2,539 randomized participants (*Jastreboff et al. N Engl J Med. 2022;387(3):205-216*). Treatment continued to be provided for an additional 104 weeks (through week 176) to the 1,032 participants who were selected at baseline to have prediabetes; participants continued on their original treatment assignment. The key secondary endpoints assessed in this 176-week continuation study were the percentage change in body weight from baseline to week 176 (evaluated in the 10 mg and 15 mg tirzepatide groups and the placebo group) and the incidence of type 2 diabetes, as assessed in the pooled tirzepatide groups and the placebo group) through the 176-week treatment period and the 17-week (off-drug) safety follow-up visit (week 193).

Safety assessments continued throughout the 176-week continuation trial.

The Patient Health Questionnaire-9 (PHQ-9), described in the Methods section, was administered at weeks 85, 98, 111, 124, 137, 150, 163, 176, and 188 (for a total of 9 assessments during the continuation trial, including the off-drug safety visit at week 188).

The Columbia-Suicide Severity Rating Scale (S-SSRS) was administered at all study visits during the continuation trial, which occurred at approximately 6 to 7 week intervals (i.e., weeks 78, 85, 91, 98, 104, 111, 117, 124, 130, 137, 143, 150, 156, 163, 169, 176, and 188, for a total of 17 assessments during the continuation trial, including the off-drug safety visit).

Adverse events and severe adverse events were collected at all study visits during continuation trial.

Results at Week 176 for Participant Retention and Primary Endpoints

A total of 136 of 270 participants (50.4%) randomized to placebo completed the 176-week trial, compared with 172 of 247 (69.6%) assigned to tirzepatide 5 mg, 185 of 262 (70.6%) assigned to tirzepatide 10 mg, and 184 of 253 (72.7%) assigned to tirzepatide 15 mg.

The mean percentage change in body weight from baseline to week 176 was -1.3% for placebo, as compared with -12.3%, -18.7%, and -19.7% for the 5, 10, and 15 mg doses of tirzepatide, respectively ($P<0.001$ for all comparisons with placebo).

At week 176, fewer participants in the pooled tirzepatide group received a diagnosis of type 2 diabetes as compared with participants in the placebo group (1.3% vs 13.3%; hazard ratio, 0.07; 95% confidence interval [CI], 0.0 to 0.1; $p<0.001$). At week 193 (17 weeks after treatment discontinuation), 2.4% of participants assigned to tirzepatide and 13.7% of those who received placebo were diagnosed with type 2 diabetes (hazard ratio, 0.12; 95% CI, 0.1 to 0.2; $p<0.001$).

Psychiatric Safety During the 3-year SURMOUNT-1 Study in People with Obesity and Prediabetes

The tables and figures that follow present changes from baseline through week 176 (and through the safety follow-up visit) in the 1,032 participants who had prediabetes when randomized to SURMOUNT-1. These results provide preliminary information about the long-term psychiatric safety of tirzepatide for weight management, relative to placebo. The findings generally mirror the favorable psychiatric safety observed with tirzepatide in the pooled 72-week analysis. However, the findings should be considered exploratory, in part, because of the increased rates of participant attrition at week 176, particularly in the placebo group.

Table S1. Mean PHQ-9 Score by Week, Change from Baseline in PHQ-9 Score, and Proportion of Participants Reaching a PHQ-9 Total Score ≥15 Anytime Postbaseline in the 3-year SURMOUNT-1 Study in People with Obesity and Prediabetes

	**Pooled Tirzepatide N=762**	**Placebo N=270**
**Week 0**	2.7 ± 3.05 *n=761*	2.8 ± 3.24 *n=270*
**Week 12**	2.0 ± 2.51 *n=735*	2.5 ± 3.23 *n=260*
**Week 24**	1.9 ± 2.44 *n=733*	2.2 ± 2.59 *n=256*
**Week 36**	1.7 ± 2.48 *n=724*	2.1 ± 2.81 *n=239*
**Week 48**	1.6 ± 2.22 *n=706*	2.2 ± 2.78 *n=221*
**Week 60**	1.6 ± 2.27 *n=698*	2.3 ± 2.93 *n=214*
**Week 72**	1.8 ± 2.56 *n=688*	2.7 ± 3.62 *n=211*
**Week 85**	1.7 ± 2.68 *n=609*	2.2 ± 3.01 *n=172*
**Week 98**	1.6 ± 2.36 *n=592*	2.1 ± 2.51 *n=157*
**Week 111**	1.4 ± 2.15 *n=586*	2.1 ± 3.05 *n=153*
**Week 124**	1.8 ± 2.55 *n=579*	2.1 ± 2.59 *n=150*
**Week 137**	1.7 ± 2.58 *n=569*	2.0 ± 2.52 *n=144*
**Week 150**	1.5 ± 2.18 *n=560*	2.1 ± 2.80 *n=141*
**Week 163**	1.5 ± 2.28 *n=556*	2.3 ± 3.48 *n=136*
**Week 176**	1.7 ± 2.49 *n=552*	2.6 ± 2.81 *n=142*

**Safety follow-up**	2.1 ± 2.71<br>*n=591*	2.4 ± 2.94<br>*n=166*
**Last on-treatment**	1.8 ± 2.56<br>*n=735*	2.3 ± 2.56<br>*n=258*
**Last on-study**	2.1 ± 2.70<br>*n=748*	2.3 ± 2.74<br>*n=261*
**Change from baseline to week 176, LSM ± SE**	**-0.8 ± 0.10**<br>***n=552***	**0.0 ± 0.20**<br>***n=142***
Difference vs placebo, LSM ± SE (p-value[a])	-0.9 ± 0.22 (p<0.001)	
**Change from baseline to safety follow-up, LSM ± SE**	**-0.6 ± 0.11**<br>***n=591***	**-0.3 ± 0.20**<br>***n=166***
Difference vs placebo, LSM ± SE (p-value[a])	-0.3 ± 0.23 (p=0.149)	
**Change from baseline to last on-study, LSM ± SE**	**-0.6 ± 0.09**<br>***n=748***	**-0.5 ± 0.16**<br>***n=261***
Difference vs placebo, LSM ± SE (p-value[a])	-0.2 ± 0.18 (p=0.303)	
**Proportion of participants reaching a total score ≥15 any time postbaseline[b], n (%)**	**18 (2.4)**	**15 (5.6)**
Odds ratio vs placebo (p-value[a])	0.41 (p=0.015)	
**Reporting PHQ-9 Item 9 anytime postbaseline, n (%)**	**34 (4.5)**	**14 (5.2)**
Odds ratio vs placebo (p-value[a])	0.86 (p=0.637)	

Data are mean ± SD PHQ-9 scores, unless otherwise noted, from the modified intent-to-treat population (safety analysis set). Pooled tirzepatide doses include 5 mg, 10 mg and 15 mg.

[a] p-value is based on ANCOVA or logistic model with treatment as factor and baseline PHQ-9 total score as covariate.

[b] Participants who had both a baseline total score ≥15 and a postbaseline total score ≥15 were not included.

Abbreviations: ANCOVA=analysis of covariance; LSM=least squares mean; N/A=not applicable; PHQ-9=Patient Health Questionnaire 9; SD=standard deviation; SE=standard error.

Table S2. Categorical Shift in PHQ-9 Total Score from Baseline to Safety Follow-up in the 3-year SURMOUNT-1 Study in People with Obesity and Prediabetes

SURMOUNT-1 3-Year Trial: Baseline PHQ-9 Category by Score	n (%)	PHQ-9 category at week 193 for pooled tirzepatide (N=762) or placebo (N=270)[a], n (%)				
		None (0-4)	Mild (5-9)	Moderate (10-14)	Moderately Severe (15-19)	Severe (20-27)
**None (0-4)**						
Pooled Tirzepatide	518 (68.0)	370 (71.4)	116 (22.4)	26 (5.0)	6 (1.2)	0
Placebo	169 (62.6)	122 (72.2)	35 (20.7)	8 (4.7)	3 (1.8)	1 (0.6)
**Mild (5-9)**						
Pooled Tirzepatide	180 (23.6)	73 (40.6)	70 (38.9)	31 (17.2)	4 (2.2)	2 (1.1)
Placebo	70 (25.9)	20 (28.6)	28 (40.0)	15 (21.4)	6 (8.6)	1 (1.4)
**Moderate (10-14)**						
Pooled Tirzepatide	49 (6.4)	14 (28.6)	15 (30.6)	12 (24.5)	7 (14.3)	1 (2.0)
Placebo	22 (8.1)	3 (13.6)	8 (36.4)	7 (31.8)	1 (4.5)	3 (13.6)
**Moderately Severe (15-19)**						
Pooled Tirzepatide	1 (0.1)	0	1 (100.0)	0	0	0
Placebo	0	0	0	0	0	0
**Severe (20-27)**						
Pooled Tirzepatide	0	0	0	0	0	0
Placebo	0	0	0	0	0	0

Data are n (%) from the modified intent-to-treat population. Pooled tirzepatide doses include 5 mg, 10 mg and 15 mg. Note: Categorical shift in PHQ-9 total score to “improved”, “stable” or “not improved” categories are indicated in green, yellow, and red, respectively.
[a] Missing data for 14 (1.8%) tirzepatide-treated participants and 9 (3.3%) placebo-treated participants.
Abbreviations: PHQ-9=Patient Health Questionnaire 9.

Table S3. Increase/Decrease in Depression Based on PHQ-9 Score from Baseline to Week 176 and at Safety Follow-up in the 3-year SURMOUNT-1 Study in People with Obesity and Prediabetes

Outcome of interest	Pooled Tirzepatide N=762	Placebo N=270
**Increase in depression**		
**Any increase in depression category**[a]	193 (25.8)	73 (28.0)
**Increase from no or mild depression to moderate, moderately severe or severe depression**[b]	69 (9.9)	34 (14.2)
**Increase from mild or moderate depression to moderately severe or severe depression**[c]	14 (6.1)	11 (12.0)
**Decrease in depression**		
**Any decrease in depression category**[d]	103 (44.8)	31 (33.7)
**Decrease from mild depression to no depression**[e]	73 (40.6)	20 (28.6)
**Decrease from moderate depression to mild depression**[f]	15 (30.6)	8 (36.4)
**Decrease from mild or moderate depression to no depression**[g]	87 (38.0)*	23 (25.0)

Data are n (%) from baseline max to postbaseline max, from the modified intent-to-treat population (safety analysis set). The denominator is the number of participants with baseline and ≥1 postbaseline measurement in each category. P-value is based on Cochran Mantel-Haenszel test to adjust for trial. Pooled tirzepatide doses include 5 mg, 10 mg and 15 mg. *p<0.05 vs placebo.

[a] Includes participants in the none, mild, moderate, or moderately severe category during baseline and with ≥1 postbaseline measurement (tirzepatide, N=748; placebo, N=261).

[b] Includes participants in the none or mild depression category during baseline and with ≥1 postbaseline measurement (tirzepatide, N=698; placebo, N=239).

[c] Includes participants in the mild or moderate depression category during baseline and with ≥1 postbaseline measurement (tirzepatide, N=229; placebo, N=92).

[d] Includes participants in the mild, moderate, moderately severe or severe category during baseline and with ≥1 postbaseline measurement (tirzepatide, N=230; placebo, N=92).

[e] Includes participants in the mild depression category during baseline and with ≥1 postbaseline measurement (tirzepatide, N=180; placebo, N=70).

[f] Includes participants in the moderate depression category during baseline and with ≥1 postbaseline measurement (tirzepatide, N=49; placebo, N=22).

[g] Includes participants in the mild or moderate depression category during baseline and with ≥1 postbaseline measurement (tirzepatide, N=229; placebo, N=92).

Abbreviations: PHQ-9=Patient Health Questionnaire 9.

Table S4. Proportion of Participants with Suicidal Ideation and Behavior as Assessed by the Columbia Suicide Severity Rating Scale (C-SSRS) from Baseline to Safety Follow-up Visit in the 3-year SURMOUNT-1 Study in People with Obesity and Prediabetes

**Events during treatment**	**Pooled Tirzepatide N=758**	**Placebo N=268**
**Suicidal ideation or behavior (1-10)[a]**	**6 (0.8)**	**3 (1.1)**
**Suicidal ideation (1-5)[b]**	**6 (0.8)**	**3 (1.1)**
**Low risk**		
1) Wish to be dead	6 (0.8)	3 (1.1)
2) Non-specific active suicidal thoughts	2 (0.3)	1 (0.4)
**Moderate risk**		
3) Active suicidal ideation with any method (not plan) without intent to act	3 (0.4)	0
**Severe risk**		
4) Active suicidal ideation with some intent to act, without specific plan	1 (0.1)	0
5) Active suicidal ideation with specific plan and intent	0	0
**Suicidal behavior (6-10)[c]**	**0**	**0**
6) Preparatory acts or behavior	0	0
7) Aborted attempt	0	0
8) Interrupted attempt	0	0
9) Non-fatal suicide attempt	0	0
10) Completed suicide	0	0
**Self-injurious behavior without suicidal attempt**	**0**	**0**

Data are number of participants (%) with different types of suicidal ideation and behavior at safety follow-up (safety analysis set) from the modified intent-to-treat population. Pooled tirzepatide doses include 5 mg, 10 mg and 15 mg.
[a] Includes participants who experience any one of the ten suicidal ideation events or suicidal behavior events at least once during treatment.
[b] Includes participants who experience any one of the five suicidal ideation events at least once during treatment.
[c] Includes participants who experience any one of the five suicidal behavior events at least once during treatment.
Abbreviations: C-SSRS=Columbia-Suicide Severity Rating Scale.

Table S5. Suicidal Ideation and Behavior as Assessed by the Columbia Suicide Severity Rating Scale (C-SSRS) Through Safety Follow-up (SFU) in the 3-year SURMOUNT-1 Study in People with Obesity and Prediabetes

Participant (Sex, Age)	Treatment Group	SI Reported (Week)	SI/SB[a,b]	Worse SI/SB Type[a,b]	SI at End of Treatment Period / SFU (Yes, No)
**#25 (F, 58y)**	**Tirzepatide 5 mg**	143	1	1	No / No
**#26 (F, 61y)**	**Tirzepatide 5 mg**	176	1	1	Yes / No
**#27 [c] (F, 57y)**	**Tirzepatide 15 mg**	91	1	1	No / No
		98	1	1	
**#28 [c] (F, 44y)**	**Tirzepatide 10 mg**	60	1	1	
		98	1,3	3	No / No
**#12 (F, 59y)**	**Tirzepatide 15 mg**	36	1,2,3	3	No / No
**#15 [c] (F, 27y)**	**Tirzepatide 10 mg**	8	1,2,3,4	4	No / Yes
		48	1	1	
		SFU	1	1	
**#29 (M, 72y)**	**Placebo**	150	1	1	No / No
**#19 (F, 67y)**	**Placebo**	SFU	1	1	No / Yes
**#22 (M, 29y)**	**Placebo**	60	1,2	2	No / No

Note: No participants reported suicide ideation or behavior at baseline (Week 0).
[a] Suicidal ideation types 1–5 using the C-SSRS were defined as: (1) wish to be dead; (2) non-specific active suicidal thoughts; (3) active suicidal ideation with any method (not plan) without intent to act; (4) active suicidal ideation with some intent to act, without specific plan; and (5) active suicidal ideation with specific plan and intent.
[b] Suicidal behavior types 6–10 using the C-SSRS were defined as (6) preparatory acts or behavior; (7) aborted attempt; (8) interrupted attempt; (9) non-fatal suicide attempt; (10) completed suicide.
[c] Reported history of suicidal ideation or behavior in participant's lifetime and/or in the month prior to study treatment.
Abbreviations: C-SSRS=Columbia-Suicide Severity Rating Scale; MTD=maximum tolerated dose; SB=suicidal behavior; SFU=safety follow-up; SI=suicidal ideation; y=years.

Table S6. Treatment-emergent Nervous System and Psychiatric Disorder Adverse Events in the 3-year SURMOUNT-1 Study in People with Obesity and Prediabetes

**High-level Group Term**<br>**Preferred Term**	**Pooled Tirzepatide N=762**	**Placebo N=270**
**Participants with ≥1 treatment-emergent nervous system disorder event**	**121 (15.88)**	**48 (17.78)**
**CNS hemorrhages and cerebrovascular accidents**	2 (0.26)	1 (0.37)
Cerebrovascular accident	1 (0.13)	0
Ischemic stroke	0	1 (0.37)
Vertebrobasilar stroke	1 (0.13)	0
**Cervical spinal cord and nerve root disorders**	0	1 (0.37)
Cervical radiculopathy	0	1 (0.37)
**Disturbances in consciousness NEC**	12 (1.57)	4 (1.48)
Lethargy	3 (0.39)	0
Loss of consciousness	1 (0.13)	0
Somnolence	4 (0.52)	1 (0.37)
Syncope	4 (0.52)	3 (1.11)
**Dyskinesias and movement disorders NEC**	1 (0.13)	0
Psychomotor hyperactivity	1 (0.13)	0
**Facial cranial nerve disorders**	0	1 (0.37)
Bell's palsy	0	1 (0.37)
**Generalized tonic-clonic seizures**	1 (0.13)	0
Generalized tonic-clonic seizures	1 (0.13)	0
**Headaches NEC**	52 (6.82)	21 (7.78)
Headache	50 (6.56)	21 (7.78)
Tension headache	2 (0.26)	1 (0.37)
**Increased intracranial pressure disorders**	1 (0.13)	0
Brain edema	1 (0.13)	0
**Lumbar spinal cord and nerve root disorders**	3 (0.39)	3 (1.11)
Lumbar radiculopathy	1 (0.13)	0
Sciatica	3 (0.39)	3 (1.11)
**Memory loss (excluding dementia)**	0	1 (0.37)
Memory impairment	0	1 (0.37)
**Mental impairment (excluding dementia and memory loss)**	0	1 (0.37)
Disturbance in attention	0	1 (0.37)
**Migraine headaches**	9 (1.18)	1 (0.37)
Migraine	9 (1.18)	1 (0.37)
**Mononeuropathies**	3 (0.39)	1 (0.37)
Carpel tunnel syndrome	0	1 (0.37)
Nerve compression	1 (0.13)	0
Peroneal nerve palsy	2 (0.26)	0
**Multiple sclerosis acute and progressive**	1 (0.13)	0
Multiple sclerosis	1 (0.13)	0
Multiple sclerosis relapse	1 (0.13)	0
**Neurological signs and symptoms NEC**	36 (4.72)	8 (2.96)
Brain fog	1 (0.13)	0

Dizziness	32 (4.20)	8 (2.96)
Dizziness postural	2 (0.26)	0
Presyncope	2 (0.26)	0
**Neuromuscular junction dysfunction**	0	1 (0.37)
Myasthenia gravis	0	1 (0.37)
**Olfactory nerve disorders**	3 (0.39)	2 (0.74)
Anosmia	1 (0.13)	2 (0.74)
Hyposmia	1 (0.13)	0
Parosmia	1 (0.13)	0
**Optic nerve disorders NEC**	1 (0.13)	0
Optic neuritis	1 (0.13)	0
**Paresthesia and dysesthesia**	5 (0.66)	2 (2.22)
Hypoesthesia	2 (0.26)	4 (1.48)
Paresthesia	3 (0.39)	2 (0.74)
**Peripheral neuropathies NEC**	1 (0.13)	1 (0.37)
Neuropathy peripheral	1 (0.13)	1 (0.37)
**Seizures and seizure disorders NEC**	1 (0.13)	1 (0.37)
Epilepsy	1 (0.13)	1 (0.37)
Idiopathic generalized epilepsy	0	1 (0.37)
**Sensory abnormalities NEC**	8 (1.05)	2 (0.74)
Dysgeusia	5 (0.66)	0
Neuralgia	0	1 (0.37)
Post herpetic neuralgia	1 (0.13)	0
Taste disorder	2 (0.26)	1 (0.37)
**Structural brain disorder NEC**	0	1 (0.37)
White matter lesion	0	1 (0.37)
**Transient cerebrovascular events**	0	1 (0.37)
Transient ischemic attack	0	1 (0.37)
**Tremor (excluding congenital)**	1 (0.13)	0
Tremor	1 (0.13)	0
**Trigeminal disorders**	1 (0.13)	0
Trigeminal neuralgia	1 (0.13)	0
**Participants with ≥1 treatment-emergent psychiatric disorder event**	**53 (6.96)**	**23 (8.52)**
**Adjustment disorders**	1 (0.13)	0
Adjustment disorder with mixed anxiety and depression mood	1 (0.13)	0
**Anxiety disorders NEC**	1 (0.13)	0
Anxiety disorder	1 (0.13)	0
**Anxiety symptoms**	12 (1.57)	6 (2.22)
Anxiety	10 (1.31)	6 (2.22)
Stress	2 (0.26)	0
**Depressive disorders**	13 (1.71)	7 (2.59)
Depression	13 (1.71)	5 (1.85)
Major depression	0	2 (0.74)
**Disturbances in initiating and maintaining sleep**	21 (2.76)	6 (2.22)
Initial insomnia	1 (0.13)	0
Insomnia	21 (2.76)	6 (2.22)
**Increased physical activity levels**	0	1 (0.37)

Restlessness	0	1 (0.37)
**Mood alterations with depressive symptoms**	4 (0.52)	3 (1.11)
Depressed mood	4 (0.52)	3 (1.11)
**Mood alterations with manic symptoms**	2 (0.26)	0
Mania	2 (0.26)	0
**Obsessive-compulsive disorders and symptoms**	1 (0.13)	0
Dermatillomania	1 (0.13)	0
**Psychiatric symptoms NEC**	0	1 (0.37)
Abulia	0	1 (0.37)
**Sexual desire disorders**	2 (0.26)	1 (0.37)
Libido decreased	2 (0.26)	1 (0.37)
**Sleep disorders NEC**	0	1 (0.37)
Sleep disorder	0	1 (0.37)
**Somatic symptoms disorders**	0	1 (0.37)
Conversion disorder	0	1 (0.37)
**Stereotypies and automatisms**	1 (0.13)	0
Bruxism	1 (0.13)	0
**Stress disorders**	0	1 (0.37)
Post-traumatic stress disorder	0	1 (0.37)
**Substance related and addictive disorders**	1 (0.13)	0
Substance abuse	1 (0.13)	0

Data are n (%) from the modified intent-to-treat population (safety analysis set). Note: Treatment-emergent nervous system disorders adverse events were classed according to MedDRA (version 27.0) and organized by high-level group term and preferred term.

Abbreviations: CNS=central nervous system; MedDRA=Medical Dictionary for Regulatory Activities; NEC=not elsewhere classified.

Table S7. Treatment-emergent Major Depressive Disorder/Suicidal Ideation Events in the 3-year SURMOUNT-1 Study in People with Obesity and Prediabetes

**Event Category or Term**	**Pooled Tirzepatide N=762**	**Placebo N=270**
**Participants with TEAE of MDD/SI events**	**26 (3.4)**	**14 (5.2)**
**Depression (excluding suicide and self-injury) (SMQ)**	26 (3.4)	14 (5.2)
Depression	20 (2.6)	9 (3.3)
Depressed mood	4 (0.5)	3 (1.1)
Adjustment disorder with depressed mood	2 (0.3)	0
Major depression	0	2 (0.7)
Adjustment disorder with mixed anxiety and depressed mood	1 (0.1)	0

Data are n (%) from the modified intent-to-treat population (safety analysis set). Note: Treatment-emergent major depressive/suicidal ideation events were classed according to MedDRA (version 27.0).

Abbreviations: MDD=major depressive disorder; MedDRA=Medical Dictionary for Regulatory Activities; SI=suicidal ideation; SMQ=standardized MedDRA query; TEAE=treatment-emergent adverse event.

Supplemental Figure Legends

Figure S1. Patient Health Questionnaire 9 (PHQ-9) Scores Over Time, Including the Safety Follow-up (SFU) Visit, in the 3-year SURMOUNT-1 Study in People with Obesity and Prediabetes Data are mean actual values over time and LSM (SE) change from baseline at week 176 from the modified intent-to-treat population (safety analysis set). The dotted line separates the end of the treatment period from the off-treatment safety follow-up. **p<0.001 vs placebo. Pooled tirzepatide doses include 5 mg, 10 mg and 15 mg. Abbreviations: LSM=least squares mean; PHQ-9=Patient Health Questionnaire 9; SE=standard error.

Figure S2. Last On-study PHQ-9 Score by Percent Body Weight Reduction Threshold in the 3-year SURMOUNT-1 Study in People with Obesity and Prediabetes Data are mean change from baseline in PHQ-9 total score at week 176 (primary endpoint) by body weight reduction threshold (safety analysis set). The <5% subgroup included participants who gained weight. Abbreviations: PHQ-9=Patient Health Questionnaire 9.

Figure S1. Patient Health Questionnaire 9 (PHQ-9) Scores Over Time, Including the Safety Follow-up (SFU) Visit, in the 3-year SURMOUNT-1 Study in People with Obesity and Prediabetes

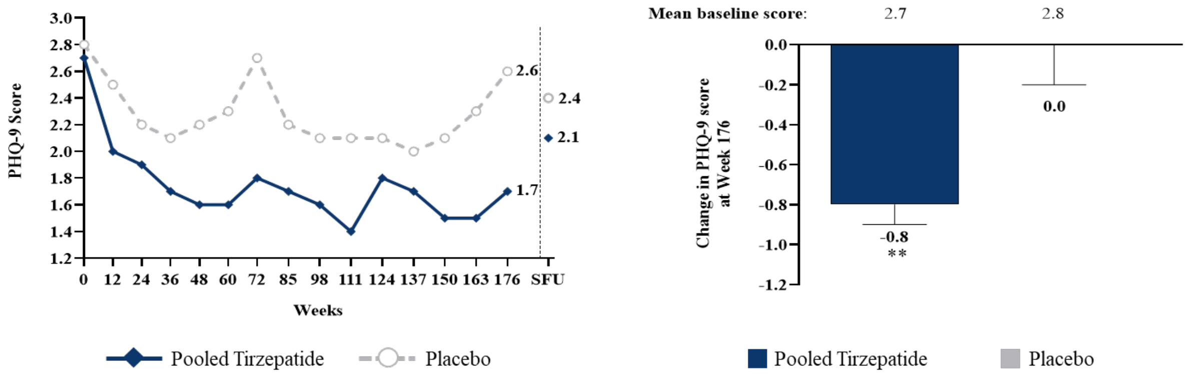

Figure S2. Last On-study PHQ-9 Score by Percent Body Weight Reduction Threshold in the 3-year SURMOUNT-1 Study in People with Obesity and Prediabetes

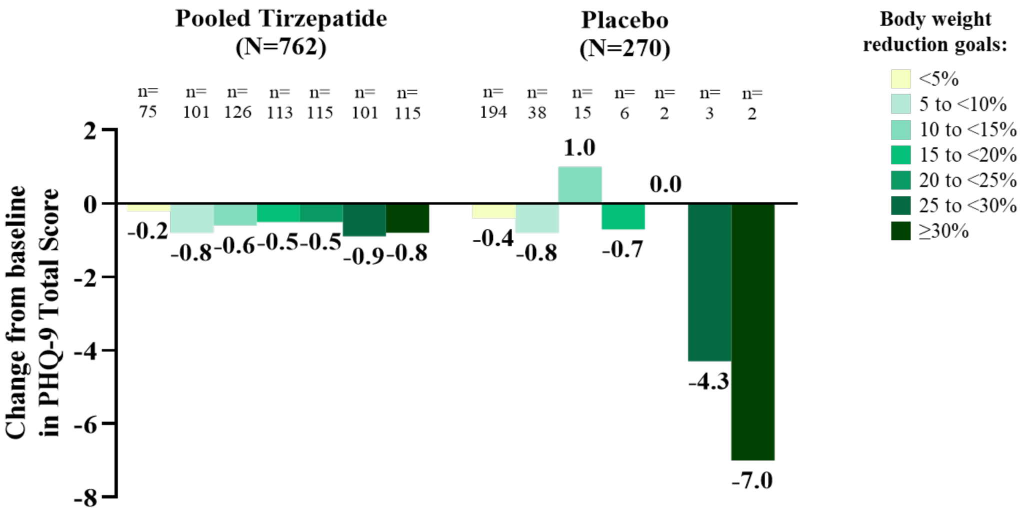

Supplement: Supplementary file 2 — Supplement S2: Data on long‐term changes (Weeks 0 to 176 and safety follow‐up visit at week 193) in psychiatric safety in a subset of participants in SURMOUNT‐1 who had prediabetes at baseline and received study medication for 176 weeks to assess the effects of tirzepatide, compared with placebo, on the incidence of type 2 diabetes. [file OBY-34-565-s005.pdf]
