# Supplementary material for: Psychiatric Safety of Tirzepatide in People With Obesity and No Known Major Psychopathology: A Post Hoc Analysis of SURMOUNT
Source: Obesity (Silver Spring). 2026 Jan 15;34(3):565–78. doi: 10.1002/oby.70122 (PMC12933222; doi:10.1002/oby.70122)
Supplement: Supplementary file 3 — Supplement S3: Full study protocol for SURMOUNT‐1. [file OBY-34-565-s002.pdf]

SUPPLEMENT 3

Psychiatric Safety of Tirzepatide in People with Obesity and No Known Major Psychopathology: A Pooled Post Hoc Analysis of the SURMOUNT-1, SURMOUNT-2, and SURMOUNT-3 Trials

Thomas A. Wadden, PhD, Maria A. Oquendo, MD, PhD, Robert F. Kushner, MD, Dachuang Cao, PhD, MS, Chrisanthi A. Karanikas, MS, Afton Kechter, PhD, Madhumita A. Murphy, MD, MS, MBA

This supplement provides data on: Study protocol for the SURMOUNT-1 trial.

Title Page

Confidential Information

The information contained in this document is confidential and is intended for the use of clinical investigators. It is the property of Eli Lilly and Company or its subsidiaries and should not be copied by or distributed to persons not involved in the clinical investigation of tirzepatide (LY3298176), unless such persons are bound by a confidentiality agreement with Eli Lilly and Company or its subsidiaries.

Note to Regulatory Authorities: This document may contain protected personal data and/or commercially confidential information exempt from public disclosure. Eli Lilly and Company requests consultation regarding release/redaction prior to any public release. In the United States, this document is subject to Freedom of Information Act (FOIA) Exemption 4 and may not be reproduced or otherwise disseminated without the written approval of Eli Lilly and Company or its subsidiaries.

Protocol Title: Efficacy and Safety of Tirzepatide Once Weekly in Participants without Type 2 Diabetes Who Have Obesity or are Overweight with Weight-Related Comorbidities: A Randomized, Double-Blind, Placebo-Controlled Trial (SURMOUNT-1)

Protocol Number: I8F-MC-GPHK(b)

Compound Number: LY3298176

Study Phase: Phase 3

Short Title: Efficacy and Safety of Tirzepatide Once Weekly Versus Placebo in Participants Who are either Obese or Overweight with Weight-Related Comorbidities (SURMOUNT-1)

Acronym: SURMOUNT-1

Sponsor Name: Eli Lilly and Company

Legal Registered Address: Indianapolis, Indiana, USA 46285

Regulatory Agency Identifier Number(s)

IND: 139721

Approval Date: Protocol Amendment (b) Electronically Signed and Approved by Lilly on date provided below.

Approval Date: 04-Oct-2021 GMT

Protocol Amendment Summary of Changes Table

DOCUMENT HISTORY	
**Document**	**Date**
Amendment (a)	24-Jul-2020
Original Protocol	09-Sep-2019

Overall Rationale for Amendment (b):

The overall rationale for the current GPHK protocol amendment is to update the key study objectives and endpoints in Section 3; clarify the definition of incident diabetes, add guidance for retesting, confirmation, recording, and adjudication of incident diabetes in Section 4.1.2.3; add guidance in Sections 6.6.1 and 6.6.3 for study drug dose modification during the additional 2-year treatment period in participants with perceived excessive weight loss; and to update information relevant to statistical analyses in Section 9 to align with recent updates to the SAP. These and other changes are listed in the below table.

Section # and Name	Description of Change	Brief Rationale
1.1. Synopsis<br>3. Objectives and Endpoints	Key secondary objective at 20 weeks was modified, changing heading from "by dose" to "pooled dose" analysis, and editing objective to reflect the pooled 10 mg and 15 mg doses.	Analysis of pooled data from participants on different tirzepatide doses is more appropriate as participants randomized to the 15-mg dose of tirzepatide will not be exposed to that dose until after Week 20.
1.1. Synopsis<br>3. Objectives and Endpoints	Key secondary objective at 72 weeks, by dose analysis, was modified:	
	For the body weight objective, added the endpoint "≥20% body weight reduction from randomization".	To better inform clinical decisions in management of people living with obesity.
	Deleted the objective and associated endpoint for SF-36v2 acute form Physical Functioning domain score for 10 mg and 15 mg separately.	Objective and associated endpoint were moved to Key secondary at 72 weeks, pooled dose analysis.

Section # and Name	Description of Change	Brief Rationale
1.1. Synopsis<br>3. Objectives and Endpoints	Revised key secondary objective at 72 weeks from "by dose" to "pooled dose" analysis to demonstrate that the pooled 10 mg and 15 mg QW tirzepatide is superior to placebo for SF-36v2 acute form Physical Functioning domain score.	To reduce the number of key secondary objectives.
1.1. Synopsis<br>3. Objectives and Endpoints	In the key secondary objective at 72 weeks, pooled dose analysis for tirzepatide (all doses combined) to demonstrate superiority to placebo for lipid parameters, the endpoint was modified to replace "LDL" with "non-HDL cholesterol", and to replace "total" with "HDL" cholesterol.	Emerging science indicates assessing HDL-cholesterol in addition to non-HDL cholesterol should provide more valuable data on CVD risk in this specific population.
1.1. Synopsis<br>3. Objectives and Endpoints	Additional secondary objective at 72 weeks, pooled dose analysis, the endpoint associated with lipid parameters was modified to replace "HDL" with "LDL" cholesterol, and to add "total cholesterol".	For consistency and to reflect the changes to the key secondary objectives.
4.1.1.2.3. Additional 2-Year Treatment Period for Participants with Prediabetes at Randomization (Visits 101 through 116)	Added wording "unless diabetes had been diagnosed prior to the visits".	For clarity.
4.1.2.2. Oral Glucose Tolerance Test	Added wording regarding use of OGTT for confirming diagnosis of diabetes.	For clarity and consistency with new text related to confirmation of diabetes diagnosis in Section 4.1.2.3.
4.1.2.3. Definition and Management of Incident Diabetes	Revised definition of incident diabetes and added subsections containing clarifying information related to "Confirmation of diabetes diagnosis" and "Recording of incident diabetes events".	Definition of incident diabetes was revised to clarify the criteria regarding the requirement for 2 abnormal test results to confirm the diagnosis.

Section # and Name	Description of Change	Brief Rationale
6.6.1. Tirzepatide	Added sentence allowing dose modification for management of participants with perceived excessive weight loss during the additional 2-year treatment period for participants with prediabetes at randomization.	To support patient management and retention in the additional 2-year treatment period.
6.6.3. Management of Participants with Perceived Excessive Weight Loss During the Additional 2-year Treatment Period for Participants with Prediabetes at Randomization	Added this subsection containing information for managing participants with perceived excessive weight loss during the additional 2-year treatment period for participants with prediabetes at randomization.	Provide clear, detailed information for managing and retaining participants in the additional 2-year treatment period.
7.2. Participant Discontinuation/ Withdrawal from the Study	Added bullet point "Female participants will be withdrawn from the study if the participant becomes pregnant."	For clarity in participant management.
9.1. Statistical Hypotheses	Updated to reflect the changes specified in the key secondary objectives and endpoints in Section 3.	For consistency.
9.4.3.1. Primary Analyses	Updated information on estimands and related analyses.	To address and align with FDA feedback.
9.4.3.2. Key Secondary Analyses	Updated to reflect the changes specified in the key objectives and endpoints in Section 3.	For consistency.
9.4.4.4.2. Events Related to Potential Abuse Liability	Updated to replace current information with "To identify AE terms suggestive of potential abuse liability, narrow terms from SMQ of Drug abuse and dependence (20000101) will be used."	Based on mechanism of action of tirzepatide, anticipated gastrointestinal adverse events, and low likelihood for abuse potential/psychoactive effects, it is reasonable to narrow the list of abuse terms used.
10.1.4. Committee Structure	Added wording pertaining to adjudication of incident diabetes events and cross-reference to related details in Section 4.1.2.3.	For clarity and consistency.

Section # and Name	Description of Change	Brief Rationale
10.4. Appendix 4: Contraceptive Guidance and Collection of Pregnancy Information	In subsection for “Female Participants who become pregnant”, modified final sentence to indicate that if a study participant becomes pregnant, the participant will discontinue study intervention “and” will be withdrawn from the study.	For clarity and to align with other changes in protocol Section 7.2.
10.7. Appendix 7: Protocol GPHK Standardized Protocols for the Measurement of Height, Weight, Waist Circumference, Vital Signs, Electrocardiogram, and Oral Glucose Tolerance Test	Added note in the text stating that placement of a venous cannula may not be required when a 2-hour OGTT is repeated to confirm the diagnosis of diabetes during the study, and only samples at 0 and 120 minutes for glucose measurement will be collected.	For clarity and to align with changes in Section 4.1.2.2.
Throughout	Used the term “additional 2-year treatment period for participants with prediabetes at randomization” to replace similar but inconsistently used terms when referring to the study period following primary outcome at Visit 21 (Week 72).	For clarity and consistency.
Throughout	Minor, editorial and document formatting revisions.	Minor, therefore have not been summarized.

Table of Contents

1. Protocol Summary 9
1.1. Synopsis 9
1.2. Schema 17
1.3. Schedule of Activities 18
1.3.1. Schedule of Activities covering visits to primary study endpoint 18
1.3.2. Schedule of Activities for additional 2-year treatment period for participants with prediabetes at randomization 22

2. Introduction 29
2.1. Study Rationale 29
2.2. Background 30
2.3. Benefit/Risk Assessment 31

3. Objectives and Endpoints 32

4. Study Design 37
4.1. Overall Design 37
4.1.1. Overview of Study Periods 37
4.1.2. Study Procedures 42
4.2. Scientific Rationale for Study Design 45
4.3. Justification for Dose 46
4.4. End of Study Definition 47

5. Study Population 48
5.1. Inclusion Criteria 48
5.2. Exclusion Criteria 49
5.3. Lifestyle Considerations 53
5.3.1. Meals and Dietary Restrictions 54
5.3.2. Physical Activity 55
5.4. Screen Failures 55

6. Study Intervention 56
6.1. Study Interventions Administered 56
6.1.1. Medical Devices 56
6.2. Preparation/Handling/Storage/Accountability 57
6.3. Measures to Minimize Bias: Randomization and Blinding 57
6.4. Study Intervention Compliance 58
6.5. Concomitant Therapy 58
6.6. Dose Modification 59
6.6.1. Tirzepatide 59
6.6.2. Management of Participants with Gastrointestinal Symptoms 59
6.6.3. Management of Participants with Perceived Excessive Weight Loss During the Additional 2-year Treatment Period for Participants with Prediabetes at Randomization 60
6.7. Intervention after the End of the Study 60
6.7.1. Treatment after Study Completion 60

7. Discontinuation of Study Intervention and Participant Discontinuation/Withdrawal 61
7.1. Discontinuation of Study Intervention 61
7.1.1. Temporary Study Drug Discontinuation 63
7.2. Participant Discontinuation/Withdrawal from the Study 64
7.2.1. Participant Disposition and Timing of Safety Follow-up for the Primary Endpoint (72 Weeks) 65
7.2.2. Participant Disposition and Timing of Safety Follow-up for those with Prediabetes at Randomization Continuing in Additional 2-year Treatment Period 66
7.2.3. Discontinuation of Inadvertently Enrolled Participants 67
7.3. Lost to Follow-up 67

8. Study Assessments and Procedures 68
8.1. Efficacy Assessments 68
8.1.1. Primary Efficacy Assessments 68
8.1.2. Secondary Efficacy Assessments 68
8.1.3. Patient-Reported Outcomes Assessments 68
8.1.4. Short Form 36 version 2, acute, 1 week recall version 69
8.1.5. Impact of Weight on Quality of Life-Lite Clinical Trials Version 69
8.1.6. EQ-5D-5L 69
8.1.7. Patient Global Impression of Status for Physical Activity 70
8.2. Safety Assessments 70
8.2.1. Physical Examinations 70
8.2.2. Vital Signs 70
8.2.3. Electrocardiograms 70
8.2.4. Clinical Safety Laboratory Assessments 71
8.2.5. Safety Monitoring 72
8.2.6. Depression, Suicidal Ideation, and Behavior Risk Monitoring 72
8.3. Adverse Events and Serious Adverse Events 73
8.3.1. Time Period and Frequency for Collecting AE and SAE Information 75
8.3.2. Follow-up of AEs and SAEs 75
8.3.3. Regulatory Reporting Requirements for SAEs 75
8.3.4. Pregnancy 76
8.3.5. Medical Device Incidents (Including Malfunctions) 76
8.3.6. Complaint Handling 77
8.4. Treatment of Overdose 77
8.5. Pharmacokinetics 78
8.6. Pharmacodynamics 78
8.7. Genetics 78
8.7.1. Whole Blood Sample(s) for Pharmacogenetic Research 78
8.8. Biomarkers 79
8.9. Health Economics 79

9. Statistical Considerations 80
9.1. Statistical Hypotheses 80

9.2. Sample Size Determination....81
9.3. Populations for Analyses....83
9.4. Statistical Analyses....83
9.4.1. General Statistical Considerations....83
9.4.2. Treatment Group Comparability....84
9.4.3. Efficacy Analyses....84
9.4.4. Safety Analyses....86
9.4.5. Pharmacokinetic/Pharmacodynamic Analyses....88
9.4.6. Evaluation of Immunogenicity....88
9.4.7. Other Analyses....88
9.5. Interim Analyses....89

10. Supporting Documentation and Operational Considerations....90
10.1. Appendix 1: Regulatory, Ethical, and Study Oversight Considerations....90
10.1.1. Regulatory and Ethical Considerations....90
10.1.2. Informed Consent Process....90
10.1.3. Data Protection....91
10.1.4. Committees Structure....91
10.1.5. Dissemination of Clinical Study Data....91
10.1.6. Data Quality Assurance....92
10.1.7. Data Capture System....92
10.1.8. Source Documents....93
10.1.9. Study and Site Closure....93
10.1.10. Publication Policy....93
10.2. Appendix 2: Clinical Laboratory Tests....94
10.3. Appendix 3: Adverse Events: Definitions and Procedures for Recording, Evaluating, Follow-Up, and Reporting....98
10.3.1. Special Safety Topics....98
10.4. Appendix 4: Contraceptive Guidance and Collection of Pregnancy Information....104
10.5. Appendix 5: Liver Safety: Suggested Actions and Follow-Up Assessments....108
10.6. Appendix 6: Medical Device Incidents: Definition and Procedures for Recording, Evaluating, Follow-Up, and Reporting....109
10.7. Appendix 7: Protocol GPHK Standardized Protocols for the Measurement of Height, Weight, Waist Circumference, Vital Signs, Electrocardiogram, and Oral Glucose Tolerance Test....111
10.8. Appendix 8: Suggested Visit Structure....114
10.9. Appendix 9: Management of Gastrointestinal Symptoms....115
10.10. Appendix 10: Provisions for Changes in Study Conduct During Exceptional Circumstances....116
10.11. Appendix 11: Abbreviations....120
10.12. Appendix 12: Protocol Amendment History....125

11. References....127

1. Protocol Summary

1.1. Synopsis

Protocol Title:

Efficacy and Safety of Tirzepatide Once Weekly in Participants without Type 2 Diabetes Who Have Obesity or are Overweight with Weight-Related Comorbidities: A Randomized, Double-Blind, Placebo-Controlled Trial (SURMOUNT-1)

Short Title:

Efficacy and Safety of Tirzepatide Once Weekly Versus Placebo in Participants Who are either Obese or Overweight with Weight-Related Comorbidities (SURMOUNT-1)

Rationale:

Obesity is a chronic disease and its increasing prevalence is a public health concern associated with the rising incidence of type 2 diabetes mellitus (T2DM), increased risk for premature death and increased risk for some cancers (Allison et al. 2008; AMA 2013; Council on Science and Public Health 2013). Although loss of 5% to 10% body weight has been shown to reduce complications related to obesity and improve quality of life (Mertens and Van Gaal 2000; Knowler et al. 2002; Jensen et al. 2014; Li et al. 2014; Warkentin et al. 2014), lifestyle therapies fail to achieve sustainable weight loss in the majority of patients with obesity (Dombrowski et al. 2014).

The gut incretin hormones, glucose-dependent insulinotropic polypeptide (GIP) and glucagon-like peptide-1 (GLP-1), are secreted after meal ingestion and mediate the incretin effect. Both hormones have effects on endocrine cells in the pancreas, increasing insulin biosynthesis and secretion, and modifying glucagon secretion (Skow et al. 2016). Based on these properties, several GLP-1 receptor (GLP-1R) agonists have been approved for pharmacological treatment of T2DM (Tomlinson et al. 2016).

In addition to its pancreatic effects, GLP-1R activation decreases gut motility, slows gastric emptying, and promotes satiety (presumably through a combination of GLP-1R activation in the central and peripheral nervous systems), thereby regulating food intake and body weight (Baggio and Drucker 2007). The US Food and Drug Administration and the European Medicines Agency approved the GLP-1R agonist liraglutide for the treatment of overweight and obesity (SAXENDA® package insert, 2014; SAXENDA® SmPc, 2015).

Preclinical data indicate that GIP also exerts effects on appetite regulation and food intake, adipose tissue, and on peripheral energy metabolism. Although studies evaluating effects of GIP on body weight have yielded equivocal results, GIP receptor (GIPR) activation may play a role in body weight regulation and targeting both the GLP-1R and the GIPR simultaneously may result in additive or synergistic effects of the 2 incretins on body weight (Coskun et al. 2018).

Tirzepatide is a 39-amino acid synthetic peptide dual GIP and GLP-1 receptor agonist. Its structure is based on the GIP sequence and includes a C20 fatty diacid moiety. It is administered once-weekly (QW) by subcutaneous (SC) injection (Coskun et al. 2018).

As a dual GIP/GLP-1R agonist, tirzepatide could exceed the efficacy of selective GLP-1R agonists by recruiting metabolically active tissues not targeted by selective GLP-1R agonists (for example, adipose tissue as indicated by the observation of increased energy utilization) (Baggio and Drucker 2007) and has the potential to reach higher efficacy in target tissues, such as insulin-producing pancreatic beta cells that express both GIPR and GLP-1R, before reaching its therapeutic limitation.

Study I8F-MC-GPHK (GPHK; SURMOUNT-1) is a Phase 3, multicenter, randomized, placebo-controlled, double-blinded study of the safety and efficacy of 5-mg, 10-mg and 15-mg tirzepatide QW, compared with placebo, when used in conjunction with a reduced-calorie diet and increased physical activity for weight management, in participants who do not have T2DM, and have obesity (BMI ≥30 kg/m^2) or are overweight (BMI ≥27 kg/m^2) with at least one weight-related comorbid condition (for example, hypertension, dyslipidemia, or cardiovascular disease). All participants will be randomized to at least 72 weeks of treatment to study the effects on body weight reduction. Participants who have prediabetes will be studied for a total of 176 weeks of treatment to provide sufficient follow-up time to detect potential differences in progression to T2DM.

Objectives and Endpoints

Objectives	Endpoints
**Primary**<br>**at 72 weeks, by dose analysis**	
To demonstrate that tirzepatide 10 mg QW is superior to placebo for:<br>• percent change in body weight AND<br>• percentage of participants with ≥5% body weight reduction<br><br>AND/OR<br><br>To demonstrate that tirzepatide 15 mg QW is superior to placebo for:<br>• percent change in body weight AND<br>• percentage of participants with ≥5% body weight reduction	• mean percent change in body weight from randomization<br>• percentage of study participants who achieve ≥5% body weight reduction from randomization
**Key secondary (controlled for Type I error)**<br>**at 20 weeks, pooled dose analysis**	
For pooled tirzepatide 10 mg and 15 mg QW doses, to demonstrate superiority to placebo for:<br>• body weight	• mean change in body weight (kg) from randomization
**Key secondary (controlled for Type I error)**<br>**at 72 weeks, by dose analysis**	
To demonstrate that tirzepatide 5 mg QW is superior to placebo for:<br>• percent change in body weight AND<br>• percentage of participants with ≥5% body weight reduction	• mean percent change in body weight from randomization<br>• percentage of study participants who achieve ≥5% body weight reduction from randomization

Objectives	Endpoints
For tirzepatide 10-mg and/or 15-mg QW doses, to demonstrate superiority to placebo for:<br>• body weight	• percentage of participants who achieve:<br>○ ≥10% body weight reduction from randomization<br>○ ≥15% body weight reduction from randomization<br>○ ≥20% body weight reduction from randomization
• waist circumference	• mean change in waist circumference (cm) from randomization
**Key secondary (controlled for Type I error) at 72 weeks, pooled dose analysis**	
To demonstrate that pooled tirzepatide 10 mg and 15 mg QW is superior to placebo for:<br>• patient-reported outcomes (SF-36v2 acute form Physical Functioning domain score)	• mean change in SF-36v2 acute form Physical Functioning domain score from randomization
**Key secondary (controlled for Type I error) at 72 weeks, pooled dose analysis**	
For tirzepatide QW (all doses combined), to demonstrate superiority to placebo for:<br>• lipid parameters	• mean change from randomization in<br>○ triglycerides (mg/dL)<br>○ non-HDL cholesterol (mg/dL)<br>○ HDL cholesterol (mg/dL)
• SBP	• mean change in SBP (mmHg) from randomization
• insulin	• mean change in fasting insulin (pmol/L) from randomization

Objectives	Endpoints
**Key secondary (controlled for Type I error) at 176 weeks, by dose analysis**	
For tirzepatide 10-mg and/or 15-mg QW doses, to demonstrate superiority to placebo (for participants with prediabetes at randomization) for:<br>• body weight	• mean percent change in body weight from randomization
**Key secondary (controlled for Type I error) at 176 or 193 weeks, pooled dose analysis**	
For tirzepatide QW (all doses combined), to demonstrate superiority to placebo (for participants with prediabetes at randomization) in:<br>• delayed progression to T2DM at 176 weeks<br>• delayed progression to T2DM at 193 weeks	• time to onset of T2DM during 176 weeks treatment period<br>• time to onset of T2DM during entire study including safety follow up period
**Additional secondary at 72 weeks, by dose analysis**	
For tirzepatide 5-mg QW dose, to demonstrate superiority to placebo for:<br>• body weight<br><br>• waist circumference<br><br>• patient-reported outcomes (SF-36v2 Physical Functioning)<br><br>For tirzepatide 5-mg, 10-mg, and/or 15-mg QW doses, to demonstrate superiority to placebo for:<br>• body weight<br><br>• glycemic control	• percentage of participants who achieve:<br>o ≥10% body weight reduction from randomization<br>o ≥15% body weight reduction from randomization<br><br>• mean change in waist circumference (cm) from randomization<br><br>• mean change in SF-36v2 acute form Physical Functioning domain score from randomization<br><br>• mean change in body weight (kg) from randomization<br>• mean change in BMI (kg/m^2) from randomization<br><br>• mean change in HbA1c (%, mmol/mol) from randomization<br>• mean change in fasting glucose (mg/dL) from randomization

Objectives	Endpoints
• patient-reported outcomes	• mean change in IWQOL-Lite-CT Physical Function composite score from randomization
**Additional secondary at 72 weeks, pooled dose analysis**	
For tirzepatide QW (all doses combined), to demonstrate superiority to placebo for:<br><br>• DBP<br><br>• lipid parameters	 <br>• mean change in DBP (mmHg) from randomization<br><br>• mean change from randomization in:<br>o LDL-cholesterol (mg/dL)<br>o total cholesterol (mg/dL)<br>o VLDL-cholesterol (mg/dL)<br>o free fatty acids (mg/dL)
**Additional secondary at 176 weeks, by dose analysis**	
For tirzepatide 5-mg QW dose, to demonstrate superiority to placebo (for participants with prediabetes at randomization) for:<br><br>• body weight<br><br>For tirzepatide 5 mg, 10 mg and/or 15 mg QW doses, to demonstrate superiority to placebo (for participants with prediabetes at randomization) for:<br><br>• body weight	 <br>• mean percent change in body weight from randomization<br><br>• percentage of study participants who achieve ≥5% body weight reduction from randomization
**Additional Secondary at 176 weeks, pooled dose analysis**	
For tirzepatide QW (all doses combined), to demonstrate superiority to placebo (for participants with prediabetes at randomization) for:<br><br>• Patient-reported outcomes	 <br>• mean change in SF-36v2 acute form Physical Functioning domain score from randomization<br>• mean change in IWQOL-Lite-CT Physical Function composite score from randomization

Objectives	Endpoints
**Additional Secondary**	
**Pharmacokinetics/Pharmacodynamics**<br>• To characterize the population PK of tirzepatide and explore the relationships between the tirzepatide concentration and efficacy, safety and tolerability measures	• population PK and PD parameters

Abbreviations: AUC= area under curve: BMI = body mass index; DBP = diastolic blood pressure; HbA1c = hemoglobin A1c; HDL = high-density lipoprotein; IWQOL-Lite-CT = Impact of Weight on Quality of Life-Lite-Clinical Trials Version; LDL = low-density lipoprotein; PK = pharmacokinetics; QW = once-weekly; SF-36v2 = Short Form survey-36 version 2; SBP = systolic blood pressure; T2DM = type 2 diabetes mellitus; VLDL = very low-density lipoprotein.

Overall Design:

Study I8F-MC-GPHK is a Phase 3, multicenter, randomized, placebo-controlled, double-blinded study of the safety and efficacy of 5-mg, 10-mg, and 15-mg tirzepatide QW compared with placebo for weight management when used in conjunction with a reduced-calorie diet and increased physical activity, in participants who have obesity (BMI ≥30 kg/m^2) or are overweight (BMI ≥27 kg/m^2) with weight-related comorbidities (excluding T2DM). Eligible participants will be stratified based on prediabetes status to either 72 (no prediabetes) or 176 weeks (prediabetes), follow-up for endpoints of weight loss, and delay of diabetes onset.

Disclosure Statement: This is a parallel group-treatment study with 4 arms that is participant and investigator blinded.

Number of Participants:

Approximately 2400 participants will be randomized in a 1:1:1:1 ratio to 5-mg tirzepatide (600 participants), 10-mg tirzepatide (600 participants), 15-mg tirzepatide (600 participants), and placebo (600 participants). An upper limit of 70% enrollment of women will be used to ensure a sufficiently large sample of men.

Intervention Groups and Duration:

Study participants will be randomized in a 1:1:1:1 ratio (tirzepatide 5 mg QW, tirzepatide 10 mg QW, tirzepatide 15 mg QW, and placebo QW), stratified by country, sex, and prediabetes status.

All participants will undergo a 2-week screening period and a 72-week treatment period including a 20-week dose escalation. Participants with prediabetes at randomization who remain on study drug during the first 72 weeks will undergo a further 104-week treatment period (total treatment duration of 176 weeks). The safety follow-up period will be 4 weeks (for those completing or discontinuing study drug during the first 72 weeks) or 17 weeks (for participants completing or discontinuing study drug between Weeks 72 through 176).

Data Monitoring Committee:

An independent data monitoring committee (DMC) will review unblinded interim-analysis results.

1.2. Schema

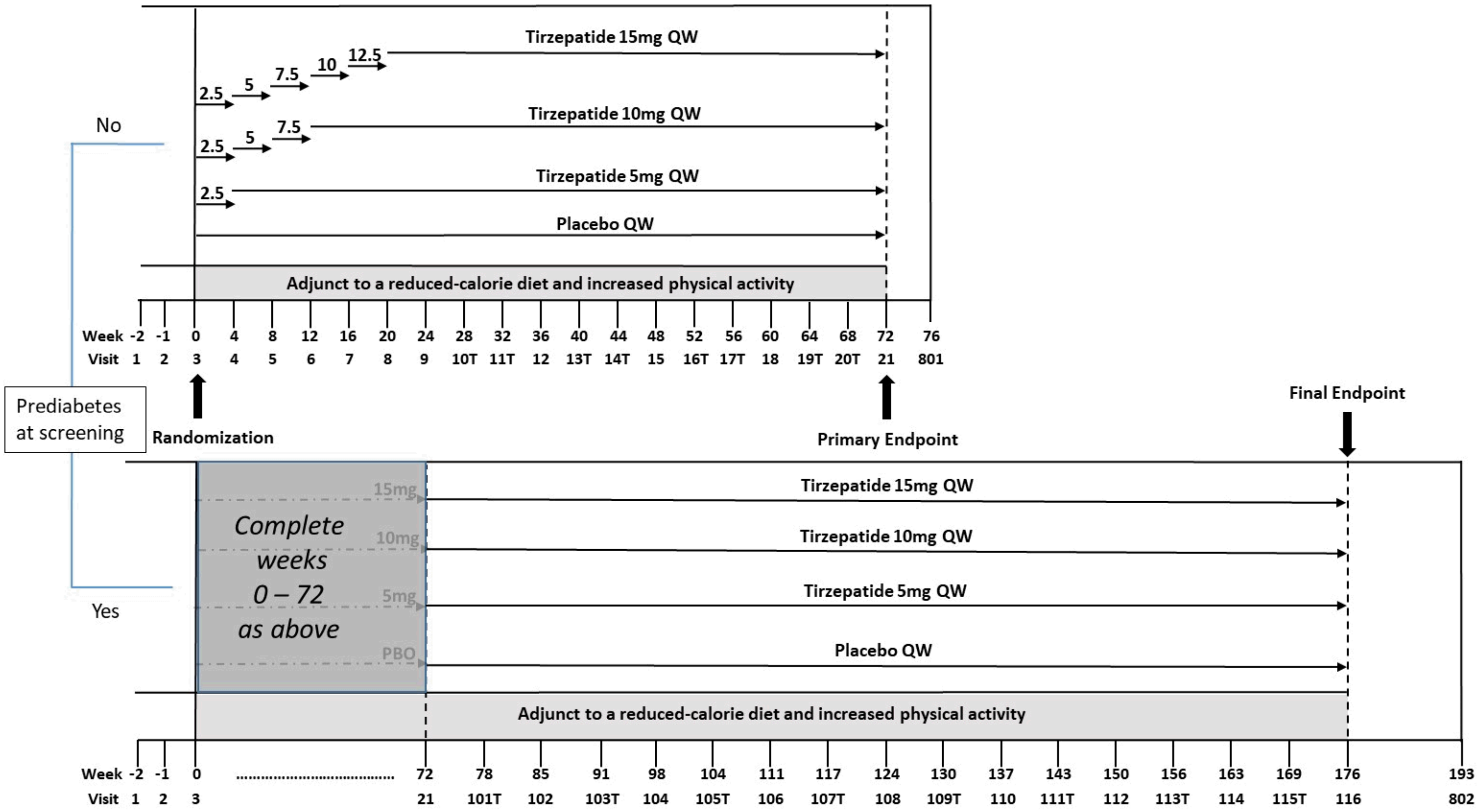

Abbreviations: QW= once weekly; T= telephone visit

All participants will be randomized to at least 72 weeks of treatment to study the effects on body weight reduction. Participants who have prediabetes will be studied for a total of 176 weeks of treatment to provide sufficient follow-up time to detect potential differences in progression to T2DM. The safety follow-up visit will occur after 4 weeks in participants discontinuing (or completing) the study within the first 72 weeks. Those discontinuing (or completing) the study after 72 weeks will undergo safety follow-up after 17 weeks.

1.3. Schedule of Activities

The Schedule of Activities described below should be followed for all participants enrolled in Study GPHK. However, for those participants whose participation in this study is affected by exceptional circumstances (such as pandemics or natural disasters), please refer to Section 10.10 Appendix 10 for additional guidance.

1.3.1. Schedule of Activities covering visits to primary study endpoint

**Visit***	**1**	**2**	**3***	**4**	**5**	**6**	**7**	**8**	**9**	**10**	**11**	**12**	**13**	**14**	**15**	**16**	**17**	**18**	**19**	**20**	**21**	**99***	**ED***	**801***
**Week of Treatment**	**-2**	**-1**	**0**	**4**	**8**	**12**	**16**	**20**	**24**	**28**	**32**	**36**	**40**	**44**	**48**	**52**	**56**	**60**	**64**	**68**	**72**	**72**		**4 wks Post TxP**
**Allowable Deviation (days)***		±3	±3	±3	+7	±3	+7	±3	±3	±3	±3	+7	±3	±3	±3	±3	±3	±3	±3	±3	±7	±7		±3
**Fasting Visit***	X	X	X	X	X	X	X	X	X			X			X			X			X	X	X	X
**Telephone Visit**										X	X		X	X		X	X		X	X				
Informed consent	X																							
Randomization			X																					
Register study visit in IWRS	X	X	X	X	X	X	X	X	X	X	X	X	X	X	X	X	X	X	X	X	X	X	X	X
Clinical Assessments																								
Medical history*	X																							
Physical examination	X																							
Height	X																							
Weight*	X		X	X	X	X	X	X	X			X			X			X			X	X	X	X
Waist circumference			X	X	X	X	X	X	X			X			X			X			X	X	X	X
Electrocardiogram*			X		X		X		X			X									X		X	X
Vital signs (3 sitting BP and HR)*	X		X	X	X	X	X	X	X			X			X			X			X		X	X
Adverse events and product complaints	X	X	X	X	X	X	X	X	X	X	X	X	X	X	X	X	X	X	X	X	X	X	X	X
Concomitant medications	X	X	X	X	X	X	X	X	X	X	X	X	X	X	X	X	X	X	X	X	X	X	X	X
Participant Education and Assessment																								
Hand out diary, instruct in use*			X			X			X			X			X			X			X			

Visit*	1	2	3*	4	5	6	7	8	9	10	11	12	13	14	15	16	17	18	19	20	21	99*	ED*	801*
**Week of Treatment**	**-2**	**-1**	**0**	**4**	**8**	**12**	**16**	**20**	**24**	**28**	**32**	**36**	**40**	**44**	**48**	**52**	**56**	**60**	**64**	**68**	**72**	**72**		**4 wks Post TxP**
**Allowable Deviation (days)***		**±3**	**±3**	**±3**	**+7**	**±3**	**+7**	**±3**	**±3**	**±3**	**±3**	**+7**	**±3**	**±3**	**±3**	**±3**	**±3**	**±3**	**±3**	**±3**	**±7**	**±7**		**±3**
**Fasting Visit***	**X**	**X**	**X**	**X**	**X**	**X**	**X**	**X**	**X**			**X**			**X**			**X**			**X**	**X**	**X**	**X**
**Telephone Visit**										**X**	**X**		**X**	**X**		**X**	**X**		**X**	**X**				
Review study participant diary, including study-drug compliance*				X	X	X	X	X	X	X	X	X	X	X	X	X	X	X	X	X	X		X	
Lifestyle Program instructions*			X	X	X	X			X			X			X			X			X		X	
Review diet and exercise goals*			X	X	X	X	X	X	X	X	X	X	X	X	X	X	X	X	X	X	X		X	
Injection training with autoinjector demonstration device*			X																					
Dispense study drug			X	X	X	X	X	X	X			X			X			X			X*			
Observe participant administer study drug*			X																					
Participant returns study drugs and injection supplies				X	X	X	X	X	X			X			X			X			X		X	
Laboratory Tests																								
Serum pregnancy test*	X																							
Urine pregnancy test*			X			X			X			X			X			X			X	X	X	
Follicle-stimulating hormone test*	X																							

Visit*	1	2	3*	4	5	6	7	8	9	10	11	12	13	14	15	16	17	18	19	20	21	99*	ED*	801*
**Week of Treatment**	-2	-1	0	4	8	12	16	20	24	28	32	36	40	44	48	52	56	60	64	68	72	72		4 wks Post TxP
**Allowable Deviation (days)***		±3	±3	±3	+7	±3	+7	±3	±3	±3	±3	+7	±3	±3	±3	±3	±3	±3	±3	±3	±7	±7		±3
**Fasting Visit***	X	X	X	X	X	X	X	X	X			X			X			X			X	X	X	X
**Telephone Visit**										X	X		X	X		X	X		X	X				
2-hour oral glucose tolerance test (includes glucose, insulin, c-peptide at each timepoint)*		X																			X		X	
Chemistry panel (include Cr for eGFR calculation and glucose)*	X		X			X			X			X			X			X			X		X	X
Lipid panel	X		X						X												X		X	X
Fasting insulin						X			X			X			X			X			X		X	X
C-peptide						X			X			X			X			X			X		X	X
Free fatty acids	X		X						X												X		X	X
Urinary albumin/creatinine	X*								X												X		X	X
Cystatin-c	X*								X												X		X	X
Calcitonin	X*					X			X												X		X	X
Hematology	X*					X			X												X		X	X
HbA1c	X		X			X			X			X			X			X			X		X	X
Pancreatic amylase, lipase	X		X			X			X												X		X	X
Thyroid-stimulating hormone	X*																							
Immunogenicity (includes PK sample)*			X	X		X			X						X						X		X	X
TZP PK*					X		X					X												
Pharmacogenetic stored sample			X																					

**Visit***	**1**	**2**	**3***	**4**	**5**	**6**	**7**	**8**	**9**	**10**	**11**	**12**	**13**	**14**	**15**	**16**	**17**	**18**	**19**	**20**	**21**	**99***	**ED***	**801***
**Week of Treatment**	**-2**	**-1**	**0**	**4**	**8**	**12**	**16**	**20**	**24**	**28**	**32**	**36**	**40**	**44**	**48**	**52**	**56**	**60**	**64**	**68**	**72**	**72**		**4 wks Post TxP**
**Allowable Deviation (days)***		**±3**	**±3**	**±3**	**+7**	**±3**	**+7**	**±3**	**±3**	**±3**	**±3**	**+7**	**±3**	**±3**	**±3**	**±3**	**±3**	**±3**	**±3**	**±3**	**±7**	**±7**		**±3**
**Fasting Visit***	**X**	**X**	**X**	**X**	**X**	**X**	**X**	**X**	**X**			**X**			**X**			**X**			**X**	**X**	**X**	**X**
**Telephone Visit**										**X**	**X**		**X**	**X**		**X**	**X**		**X**	**X**				
Nonpharmacogenetic stored sample			X			X			X						X						X		X	X
Mental Health Questionnaires																								
PHQ-9*	X		X			X			X			X			X			X			X	X	X	X
C-SSRS (Baseline/Screening) Version)*	X																							
C-SSRS (Since Last Visit Version)*		X	X	X	X	X	X	X	X	X	X	X	X	X	X	X	X	X	X	X	X	X	X	X
Self-Harm Supplement Form*	X	X	X	X	X	X	X	X	X	X	X	X	X	X	X	X	X	X	X	X	X	X	X	X
Self-Harm Follow-up Form*	X	X	X	X	X	X	X	X	X	X	X	X	X	X	X	X	X	X	X	X	X	X	X	X
Patient Reported Outcomes																								
PGIS			X																		X		X	
SF-36, version 2, acute form			X																		X		X	
IWQOL-Lite CT			X																		X		X	
EQ-5D-5L			X																		X		X	

1.3.2. Schedule of Activities for additional 2-year treatment period for participants with prediabetes at randomization

Visit*	101	102	103	104	105	106	107	108	109	110	111	112	113	114	115	116	199*	ED*	802*
**Week of Treatment**	**78**	**85**	**91**	**98**	**104**	**111**	**117**	**124**	**130**	**137**	**143**	**150**	**156**	**163**	**169**	**176**	**176**		**17 wks Post TxP**
**Allowable Deviation (days)***	±7	±7	±7	±7	±7	±7	±7	±7	±7	±7	±7	±7	±7	±7	±7	±7	±7		±3
**Fasting Visit***		**X**		**X**		**X**		**X**		**X**		**X**		**X**		**X**	**X**	**X**	**X**
**Telephone Visit**	**X**		**X**		**X**		**X**		**X**		**X**		**X**		**X**				
Register study visit in IWRS	X	X	X	X	X	X	X	X	X	X	X	X	X	X	X	X	X	X	X
Clinical Assessments																			
Weight*		X		X		X		X		X		X		X		X	X	X	X
Waist circumference		X		X		X		X		X		X		X		X	X	X	X
Electrocardiogram*								X								X		X	X
Vital signs (3 sitting BP and HR)*		X		X		X		X		X		X		X		X		X	X
Adverse events/Product Complaints	X	X	X	X	X	X	X	X	X	X	X	X	X	X	X	X	X	X	X
Concomitant medications	X	X	X	X	X	X	X	X	X	X	X	X	X	X	X	X	X	X	X
Participant Education and Assessment																			
Hand out diary, instruct in use*		X		X		X		X		X		X		X					
Review study participant diary, including study drug compliance*	X	X	X	X	X	X	X	X	X	X	X	X	X	X	X	X		X	
Lifestyle Program instructions*		X		X		X		X		X		X		X		X		X	
Review diet and exercise goals*	X	X	X	X	X	X	X	X	X	X	X	X	X	X	X	X		X	
Dispense study drug		X		X		X		X		X		X		X					

**Visit***	**101**	**102**	**103**	**104**	**105**	**106**	**107**	**108**	**109**	**110**	**111**	**112**	**113**	**114**	**115**	**116**	**199***	**ED***	**802***
**Week of Treatment**	**78**	**85**	**91**	**98**	**104**	**111**	**117**	**124**	**130**	**137**	**143**	**150**	**156**	**163**	**169**	**176**	**176**		**17 wks Post TxP**
**Allowable Deviation (days)***	±7	±7	±7	±7	±7	±7	±7	±7	±7	±7	±7	±7	±7	±7	±7	±7	±7		±3
**Fasting Visit***		**X**		**X**		**X**		**X**		**X**		**X**		**X**		**X**	**X**	**X**	**X**
**Telephone Visit**	**X**		**X**		**X**		**X**		**X**		**X**		**X**		**X**				
Participant returns study drugs and injection supplies		X		X		X		X		X		X		X		X		X	
Laboratory Tests																			
Urine pregnancy test*		X		X		X		X		X		X		X		X		X	
2 hour oral glucose tolerance test (includes glucose, insulin, c-peptide)*								X								X	X	X	X
Chemistry panel (includes Cr for eGFR calculation and glucose)*		X		X		X		X		X		X		X		X		X	X
Lipid panel								X								X		X	X
Free fatty acids								X								X		X	X
Urinary albumin/creatinine ratio								X								X		X	X
Cystatin-c								X								X		X	X
Calcitonin								X								X		X	X
Hematology								X								X		X	X
HbA1c		X		X		X		X		X		X		X		X		X	X
Pancreatic amylase, lipase								X								X		X	X
Immunogenicity (includes PK sample)*				X				X				X				X		X	X
Nonpharmacogenetic stored sample								X								X		X	X
Mental Health Questionnaires																			
PHQ-9*		X		X		X		X		X		X		X		X	X	X	X

Visit*	101	102	103	104	105	106	107	108	109	110	111	112	113	114	115	116	199*	ED*	802*
**Week of Treatment**	**78**	**85**	**91**	**98**	**104**	**111**	**117**	**124**	**130**	**137**	**143**	**150**	**156**	**163**	**169**	**176**	**176**		**17 wks Post TxP**
**Allowable Deviation (days)***	±7	±7	±7	±7	±7	±7	±7	±7	±7	±7	±7	±7	±7	±7	±7	±7	±7		±3
**Fasting Visit***		**X**		**X**		**X**		**X**		**X**		**X**		**X**		**X**	**X**	**X**	**X**
**Telephone Visit**	**X**		**X**		**X**		**X**		**X**		**X**		**X**		**X**				
C-SSRS (Since Last Visit Version)*	X	X	X	X	X	X	X	X	X	X	X	X	X	X	X	X	X	X	X
Self-Harm Supplement Form*	X	X	X	X	X	X	X	X	X	X	X	X	X	X	X	X	X	X	X
Self-Harm Follow-up Form*	X	X	X	X	X	X	X	X	X	X	X	X	X	X	X	X	X	X	X
PROs																			
PGIS								X								X		X	
SF-36, version 2 acute								X								X		X	
IWQOL-Lite -CT								X								X		X	
EQ-5D-5L								X								X		X	

*Please see table below for corresponding additional information

Abbreviations: BP = blood pressure; Cr= creatinine; C-SSRS = Columbia-Suicide Severity Rating Scale; ED= early discontinuation of treatment; eGFR = estimated glomerular filtration rate; HbA1c = hemoglobin A1c; HR = heart rate; IWQOL-Lite-CT = Impact of Weight on Quality of Life-Lite Clinical trials; IWRS = interactive web-response system; PGIS = Patient Global Impression of status for physical activity; PHQ-9 = Patient Health Questionnaire-9; PK = pharmacokinetics; SF-36 = Short-Form-36 Health Survey version 2, acute form; TxP = treatment period; TZP = tirzepatide

Additional Information Regarding Activities Described in Schedule of Activities

Activity	Notes
Visit<br>Fasting Visit	On all office visits, study participants should be reminded to report to the site before taking study drug (s) in a fasting condition, after a period of approximately 8 hours without eating, drinking (except water), or any significant physical activity. Since some screening procedures need to be completed in the fasting state, Visit 1 may be conducted over more than 1 day to ensure necessary conditions are met.
Visit 3	Baseline assessments must be completed before processing in the IWRS.
Visit 99<br>Visit 199	Participants wanting to discontinue the study before Week 72 will be asked to return for Visit 99 at 72 weeks ± 7 days after randomization primarily for body weight measurement and assessment of adverse events. If the participant is unwilling to attend Visit 99, it should be documented in the participant medical record that the participant has refused to attend. Participants wanting to discontinue the study after Week 72 but before Week 176 will be asked to return for Visit 199 primarily for the body weight measurement and assessment of adverse events. Refusal to attend should be documented.

ED	Participants who are unable or unwilling to continue the study treatment for any reason will perform an ED visit. If the participant is discontinuing during an unscheduled visit or a scheduled visit, that visit should be performed as the ED visit.
Safety Follow-up Visits<br>Visit 801<br>Visit 802	For participants who discontinue or complete the study within the first 72 weeks, Visit 801 (safety follow-up visit) should be performed 4 weeks after the last visit during the 72-week treatment period. Participants with prediabetes continuing to Week 78 should not perform Visit 801. For participants with prediabetes who complete the 176 weeks or discontinue after Week 72, Visit 802 should be performed 17 weeks after the last visit during the additional 2-year treatment period for participants with prediabetes at randomization.
Allowable Deviation (days)	The visit date is determined in relation to the date of the randomization visit (± the allowed visit window).
Medical History	Medical history includes assessment of preexisting conditions (for example, history of gallbladder disease, cardiovascular disease, and medullary thyroid carcinoma) and substance usage (such as alcohol and tobacco).
Weight	Weight measurements should be obtained per the instructions in Section 10.7. Body weight must be measured in fasting state. If the participant is not fasting, the participant should be called in for a new visit within the visit window to have the fasting body weight measured.
Electrocardiogram	ECG measurements should be obtained per the instructions in Section 10.7. ECGs will be performed in triplicate at Weeks 0 and 36. All other ECGs will be single measurements. ECGs should be collected at least 30 minutes prior to collection of blood samples for laboratory testing, including PK samples.
Vital signs (3 sitting BP and HR)	Vital-sign measurements should be taken before obtaining an ECG tracing and before collection of blood samples for laboratory testing, per the instruction in Section 10.7.
Hand out diary, instruct in use	All training should be repeated as needed to ensure participant compliance.
Review study participant diary and study-drug compliance	Data from diary, as well as study drug compliance, should be reviewed.

Lifestyle Program instructions	Counselling on diet and exercise, to be performed by a dietician or equivalent qualified delegate, to include calculation of individualized energy requirement and methods to change dietary composition and amount of physical activity. The Lifestyle Program Instruction may be delivered on a separate day from the rest of that visit's study procedures but must occur within the visit window. Beginning at Week 8, the Lifestyle Program Instruction may be delivered by phone.
Review diet and exercise goals	All training should be repeated as needed to ensure participant compliance. Study personnel to provide reinforcement and encouragement for lifestyle modifications.
Injection training with Autoinjector demonstration device	All training should be repeated as needed to ensure participant compliance
Dispense study drug Visit 72	This applies only to those participants going into the additional 2-year treatment period for participants with prediabetes at randomization.
Observe participant administer study drug	Participants should administer their first dose of study drug at the end of the Visit 3, after other study procedures are completed
Serum pregnancy test	A serum pregnancy test will be performed at Visit 1 for women of childbearing potential only.
Urine pregnancy test	A urine pregnancy test must be performed at Visit 3 with the result available prior to randomization and first injection of study drug(s) for women of childbearing potential only. Additional pregnancy tests (beyond those required per the Schedule of Activities) should be performed at any time during the trial if a menstrual period is missed, there is clinical suspicion of pregnancy, or as required by local law or regulation.
Follicle-stimulating hormone test	Follicle-stimulating hormone test performed at Visit 1 for postmenopausal women at least 40 years of age with an intact uterus, not on hormone therapy, and who have had spontaneous amenorrhea for more than 1 year without an alternative medical cause.
2-hour oral glucose tolerance test (includes glucose, insulin, c-peptide)	2-hour OGTT testing should be omitted at visits following a protocol-defined diabetes diagnosis.

Urinary albumin/creatinine ratio<br>Cystatin-c<br>Calcitonin<br>Hematology<br>Thyroid-stimulating hormone (TSH)	Screening visit assessment will be used to confirm eligibility. If calcitonin results are not available, a final review of eligibility will occur at Visit 3 before randomization can proceed.
Chemistry panel (include Cr for eGFR calculation)	The CKD-EPI equation will be used by the central lab to estimate and report eGFR.
Immunogenicity (includes PK sample)	PK samples for immunogenicity must be taken prior to drug administration at these visits. In the event of systemic drug hypersensitivity reactions (immediate or nonimmediate), additional blood samples will be collected including ADA, PK, and exploratory biomarker samples.
TZP PK	PK samples will be collected at these visits at time windows of 1 to 24 hours, 24 to 96 hours, or 120 to 168 hours post dose, as assigned by IWRS at randomization. Dependent on the time windows to which a participant is assigned, they may be required to come to site for PK-specific visits.
PHQ-9<br>C-SSRS (Baseline/Screening Version)<br>C-SSRS (Since last Visit Version)<br>Self-Harm Supplement Form	The C-SSRS, Self-Harm Supplement Form and PHQ-9 should be administered *after* assessment of adverse events. For this study, the C-SSRS is adapted for the assessment of the ideation and behavior categories only. The Intensity of Ideation and Lethality of Behavior sections are removed.
Self-Harm Follow-up Form	Self-Harm Follow-up Form is only required if triggered by the Self-Harm Supplement Form, per instructions in the form.

Abbreviations: ADA = antidrug antibody; BP = blood pressure; CKD-EPI = Chronic Kidney Disease-Epidemiology; Cr = creatinine; C-SSRS = Columbia-Suicide Severity Rating Scale; ECG = electrocardiogram; ED = early discontinuation of treatment; eGFR = estimated glomerular filtration rate; HR = heart rate; IWRS = interactive web-response system; OGTT = oral glucose tolerance test; PHQ-9 = Patient Health Questionnaire-9; PK = pharmacokinetic; TSH = thyroid-stimulating hormone; TZP = tirzepatide.

2. Introduction

Obesity is a chronic disease whose increasing prevalence is a public-health concern associated with rising incidence of type 2 diabetes mellitus (T2DM), increased risk for premature death and increased risk for some cancers (Allison et al. 2008; AMA 2013; Council on Science and Public Health 2013). Although loss of 5% to 10% body weight has been shown to reduce complications related to obesity and improve quality of life (Mertens and Van Gaal 2000; Knowler et al. 2002; Jensen et al. 2014; Li et al. 2014; Warkentin et al. 2014), lifestyle therapies fail to achieve sustainable weight loss in the majority of patients with obesity (Dombrowski et al. 2014).

2.1. Study Rationale

The gut incretin hormones, glucose-dependent insulinotropic polypeptide (GIP) and glucagon-like peptide-1 (GLP-1), are secreted after meal ingestion and mediate the incretin effect. Both hormones have effects on endocrine cells in the pancreas, increasing insulin biosynthesis and secretion, stimulating beta-cell neogenesis and proliferation, and protecting beta cells from apoptosis. They also exert actions on alpha cells, modifying glucagon secretion (Skow et al. 2016). Based on these properties, several GLP-1 receptor (GLP-1R) agonists have been approved for pharmacological treatment of T2DM (Tomlinson et al. 2016).

In addition to its pancreatic effects, GLP-1R activation decreases gut motility, slows gastric emptying, and promotes satiety (presumably through a combination of GLP-1R activation in the central and peripheral nervous system), thereby regulating food intake and body weight (Baggio and Drucker 2007). The US Food and Drug Administration and the European Medicines Agency approved the GLP-1R agonist liraglutide for the treatment of overweight and obesity (SAXENDA® package insert, 2014; SAXENDA® SmPc, 2015).

Preclinical data indicate that GIP exerts effects on appetite regulation and food intake, on adipose tissue and on peripheral energy metabolism. Although studies evaluating effects of GIP on body weight have yielded equivocal results, GIP receptor (GIPR) activation may play a role in body weight regulation and targeting both the GLP-1R and the GIPR simultaneously may result in additive or synergistic effects of the 2 incretins on body weight (Coskun et al. 2018).

Tirzepatide is a 39-amino acid synthetic peptide with agonist activity at both the GIPR and GLP-1R. Its structure is based on the GIP sequence and includes a C20 fatty di-acid moiety (Coskun et al. 2018). It is administered once weekly (QW) by subcutaneous (SC) injection. As a dual GIP/GLP-1R agonist, tirzepatide could exceed the efficacy of selective GLP-1R agonists by recruiting metabolically active tissues not targeted by selective GLP-1R agonists (for example, adipose tissue as indicated by the observation of increased energy utilization) (Baggio and Drucker 2007) and has the potential to reach higher efficacy in target tissues that express both GIPR and GLP1R.

Study I8F-MC-GPHK (GPHK, SURMOUNT-1) is a Phase 3, multicenter, randomized, placebo-controlled, double-blinded study, to investigate the safety and efficacy of 5-mg, 10-mg, and 15-mg tirzepatide QW, compared with placebo, when used in conjunction with a reduced-calorie diet and increased physical activity for body weight management in participants who do not have T2DM and have obesity (BMI ≥30 kg/m^2) or are overweight (BMI ≥27 kg/m^2) with at least one

weight-related comorbid condition (for example, hypertension, dyslipidemia, or cardiovascular disease). All participants will be randomized to at least 72 weeks of treatment to study the effects on body weight reduction. Participants who have prediabetes at randomization will be studied for a total of 176 weeks of treatment to provide sufficient follow-up time to assess the effects of tirzepatide on progression to T2D and on long-term body weight changes.

2.2. Background

There remains an unmet need in the pharmacologic treatment of obesity for drugs that are safe, efficacious and well tolerated. With the exception of orlistat, a drug that blocks absorption of dietary fat, many current obesity treatments work primarily by stimulating regions of the brain to promote satiety. Weight loss induced by GLP-1R agonists appears to be mediated through a combination of hormonal inputs to satiety centers (van Bloemendaal et al. 2014) and has not been consistently associated with changes in mental health or with potential for addiction in long-term studies conducted to establish cardiovascular safety in patients with diabetes (Marso et al. 2016a, Marso et al. 2016b, Gerstein et al. 2019). Tirzepatide, which is both a GLP-1R and GIPR agonist, has been associated with significant dose-dependent weight loss at 5-, 10- and 15-mg doses in Phase 2 studies (Frias et al. 2018).

Dose selection for obesity treatment has been informed by 3 clinical trials: a Phase 1 study, Study I8F-MC-GPGA (GPGA), and two Phase 2 studies, Study I8F-MC-GPGB (GPGB) and I8F-MC-GPGF (GPGF).

Phase 1 Study GPGA was a combination of single ascending dose (SAD) and multiple ascending dose (MAD) studies in healthy subjects and a multiple dose study in patients with T2DM. A total of 142 participants (89 healthy subjects and 53 patients with T2DM) received at least 1 dose of treatment. Doses of tirzepatide ranged from:

- 0.25 mg to 8 mg in the SAD (with maximum tolerated dose achieved at 5 mg)
- multiple doses from 0.5 mg to 4.5 mg QW and titrated doses up to 10 mg QW for 4 weeks in healthy subjects
- multiple doses at 0.5 mg and 5 mg QW and titrated up to 15 mg QW for 4 weeks in patients with T2DM in the MAD

The safety and tolerability and pharmacokinetic/pharmacodynamic (PK/PD) profiles of tirzepatide at doses and escalation regimens administered in this Phase 1 study supported further development of tirzepatide for QW dosing in patients with T2DM.

Phase 2 studies GPGB and GPGF studies provided initial safety, tolerability and efficacy data in the tirzepatide 1-to-15-mg dose range when used in treatment of patients with T2DM. In the dose range of 5 to 15 mg, tirzepatide provided significantly greater reductions in hemoglobin A1c (HbA1c) and body weight compared with dulaglutide 1.5 mg QW. The most common AEs, which were also dose dependent, were mild to moderate nausea, vomiting, and diarrhea. Study GPGF showed that adjustments in the tirzepatide dose-escalation algorithm resulted in additional reductions in the frequency of GI AEs and reduced the frequency of treatment discontinuations due to GI AEs.

2.3. Benefit/Risk Assessment

More information about the known and expected benefits, risks, serious AEs (SAEs) and reasonably anticipated AEs of tirzepatide are to be found in the Investigator's Brochure (IB).

3. Objectives and Endpoints

Objectives	Endpoints
**Primary**<br>**at 72 weeks, by dose analysis**	
To demonstrate that tirzepatide 10 mg QW is superior to placebo for:<br>• percent change in body weight AND<br>• percentage of participants with ≥5% body weight reduction<br>AND/OR<br>To demonstrate that tirzepatide 15 mg QW is superior to placebo for:<br>• percent change in body weight AND<br>• percentage of participants with ≥5% body weight reduction	• mean percent change in body weight from randomization<br>• percentage of study participants who achieve ≥5% body weight reduction from randomization
**Key Secondary (controlled for Type I error)**<br>**at 20 weeks, pooled dose analysis**	
For pooled tirzepatide 10 mg and 15 mg QW doses, to demonstrate superiority to placebo for:<br>• body weight	• mean change in body weight (kg) from randomization
**Key Secondary (controlled for Type I error)**<br>**at 72 weeks, by dose analysis**	
To demonstrate that tirzepatide 5 mg QW is superior to placebo for:<br>• percent change in body weight AND<br>• percentage of participants with ≥5% body weight reduction	• mean percent change in body weight from randomization<br>• percentage of study participants who achieve ≥5% body weight reduction from randomization

Objectives	Endpoints
For tirzepatide 10 mg and/or 15 mg QW doses, to demonstrate superiority to placebo for:<br>• body weight<br>• waist circumference	• percentage of participants who achieve:<br>o ≥10% body weight reduction from randomization<br>o ≥15% body weight reduction from randomization<br>o ≥20% body weight reduction from randomization<br>• mean change in waist circumference (cm) from randomization
**Key Secondary (controlled for Type I error)**<br>**at 72 weeks, pooled dose analysis**	
To demonstrate that pooled tirzepatide 10 mg and 15 mg QW is superior to placebo for:<br>• patient-reported outcomes (SF-36v2 acute form Physical Functioning domain score)	• mean change in SF-36v2 acute form Physical Functioning domain score from randomization
**Key Secondary (controlled for Type I error)**<br>**at 72 weeks, pooled dose analysis**	
For tirzepatide QW (all doses combined), to demonstrate superiority to placebo for:<br>• lipid parameters<br>• SBP<br>• insulin	• mean change from randomization in<br>o triglycerides (mg/dL)<br>o non-HDL cholesterol (mg/dL)<br>o HDL cholesterol (mg/dL)<br>• mean change in SBP (mmHg) from randomization<br>• mean change in fasting insulin (pmol/L) from randomization
**Key Secondary (controlled for Type I error)**<br>**at 176 weeks, by dose analysis**	
For tirzepatide 10 mg and/or 15 mg QW doses, to demonstrate superiority to placebo (for participants with prediabetes at randomization) for:<br>• body weight	• mean percent change in body weight from randomization

**Objectives**	**Endpoints**
**Key Secondary (controlled for Type I error)**<br>**at 176 or 193 weeks, pooled dose analysis**	
For tirzepatide QW (all doses combined), to demonstrate superiority to placebo (for participants with prediabetes at randomization) in:<br>• delayed progression to T2DM at 176 weeks<br>• delayed progression to T2DM at 193 weeks	• time to onset of T2DM during 176 weeks treatment period<br>• time to onset of T2DM during entire study including safety follow up period
**Additional secondary at 72 weeks, by dose analysis**	
**Efficacy**<br>For tirzepatide 5 mg QW dose, to demonstrate superiority to placebo at 72 weeks for:<br>• body weight	• percentage of participants who achieve<br>o ≥10% body weight reduction from randomization<br>o ≥15% body weight reduction from randomization
• waist circumference	• mean change in waist circumference (cm) from randomization
• patient reported outcomes (SF-36v2 Physical Functioning)	• mean change in SF-36 v2 acute form Physical Functioning domain score from randomization
For tirzepatide 5 mg, 10 mg, and/or 15 mg QW doses, to demonstrate superiority to placebo for:	
• body weight	• mean change in body weight (kg) from randomization<br>• mean change in BMI (kg/m^2) from randomization
• glycemic control	• mean change in HbA1c (%, mmol/mol) from randomization<br>• mean change in fasting glucose (mg/dL) from randomization
• Patient-reported outcomes	• Mean change in IWQOL-Lite-CT Physical Function composite score from randomization

Objectives	Endpoints
**Additional secondary at 72 weeks, pooled dose analysis**	
For tirzepatide QW (all doses combined), to demonstrate superiority to placebo at 72 weeks for	
• DBP	• mean change in DBP (mmHg) from randomization
• lipid parameters	• mean change from randomization in:<br>o LDL-cholesterol (mg/dL)<br>o total cholesterol (mg/dL)<br>o VLDL-cholesterol (mg/dL)<br>o Free Fatty acids (mg/dL)
**Additional secondary at 176 weeks, by dose analysis**	
For tirzepatide 5 mg QW dose, to demonstrate superiority to placebo (for participants with prediabetes at randomization) for:	
• body weight	• mean percent change in body weight from randomization
For tirzepatide 5 mg, 10 mg, and/or 15 mg QW doses, to demonstrate superiority to placebo (for participants with prediabetes at randomization) for:	
• body weight	• percentage of study participants who achieve ≥5% body weight reduction from randomization
**Additional Secondary at 176 weeks, pooled dose analysis**	
For tirzepatide QW (all doses combined), to demonstrate superiority to placebo (for participants with prediabetes at randomization) for:	
• Patient-reported outcomes	• mean change in SF-36v2 acute form Physical Functioning domain score from randomization<br>• mean change in IWQOL-Lite-CT Physical Function composite score from randomization

Objectives	Endpoints
**Additional Secondary**	
**Pharmacokinetics/Pharmacodynamics**<br>• To characterize the population PK of tirzepatide and explore the relationships between the tirzepatide concentration and efficacy, safety and tolerability measures	• population PK and PD parameters

Abbreviations: AUC = area under the curve; BMI = body mass index; DBP = diastolic blood pressure; HbA1c = hemoglobin A1c; HDL = high-density lipoprotein; IWQOL-Lite-CT = Impact of Weight on Quality of Life-Lite-Clinical Trials Version; LDL = low-density lipoprotein; PD = pharmacodynamics; PK = pharmacokinetics; QW = once-weekly; SAP = statistical analysis plan; SBP = systolic blood pressure; SF-36v2 = Short Form survey-36 version 2, T2DM = type 2 diabetes mellitus; VLDL = very low-density lipoprotein.

4. Study Design

4.1. Overall Design

Study GPHK (SURMOUNT-1) is a Phase 3, multicenter, randomized, double-blinded, placebo-controlled study that will investigate the effects of treatment with tirzepatide 5 mg, 10 mg, and 15 mg QW compared with placebo on weight loss in study participants without T2DM who are either obese (BMI ≥30 kg/m^2) or overweight (≥27 kg/m^2) with at least one weight-related comorbid condition (for example, hypertension, dyslipidemia, or cardiovascular disease). The coprimary endpoints will be mean percent change in weight and percentage of participants reaching ≥5% total body weight loss, measured from randomization to Week 72.

Study participants will be randomized in a 1:1:1:1 ratio (tirzepatide 5 mg QW, tirzepatide 10 mg QW, tirzepatide 15 mg QW, and placebo). An upper limit of 70% enrollment of women will be used to ensure a sufficiently large sample of men.

All participants will undergo a 2-week screening period and a 72-week treatment period including a 20-week dose escalation. Participants who have prediabetes will be studied for a total of 176 weeks of treatment to provide sufficient follow-up time to detect potential differences in progression to T2DM.

The safety follow-up period will be 4 weeks (for participants completing or discontinuing study drug during the first 72 weeks) or 17 weeks (for participants completing or discontinuing study drug between Weeks 72 through 176).

4.1.1. Overview of Study Periods

4.1.1.1. Visit Structure for all Office Visits

On all designated fasting office visits, study participants are required to report to the site in a fasting condition, after approximately 8 hours without eating, drinking (except water), or performing any significant physical activity. If a participant is adversely affected by the fasting condition, they are allowed to eat, however, specific study procedures need to be completed while fasting. See Section 10.8 for a suggested order of activities that occurs at office visits.

4.1.1.2. Main Study Period

4.1.1.2.1. Screening Period

Visit 1

The purpose of screening procedures at Visit 1 is to establish initial eligibility, and to obtain blood samples for laboratory assessments needed to confirm eligibility and classify glycemic status (Section 4.1.2.1). The participant must sign the informed consent form (ICF) before the study procedures are performed, as outlined in the Schedule of Activities, Section 1.3. Since some screening procedures need to be completed in the fasting state (approximately 8 hours without eating, drinking [except water], or any significant physical activity), Visit 1 may be conducted over more than 1 day to ensure necessary conditions are met. Participants who have no glycemic assessments suggestive of T2DM (Section 4.1.2.1) and meet all other applicable

inclusion criteria and none of the applicable exclusion criteria (Sections 5.1 and 5.2) at Visit 1 will continue to Visit 2.

Visit 2

Participants should arrive to the clinic in the fasting state (approximately 8 hours without eating, drinking [except water], or any significant physical activity). At Visit 2, the screening laboratory results will be reviewed. For those participants without glycemic evidence of T2DM (Section 4.1.2.1), and meeting all other eligibility requirements, a 2-hour oral glucose tolerance test (OGTT) will be performed. The 2-hour OGTT consists of assessment of glucose, insulin, and c-peptide at time 0, 30, 60, 90, and 120 minutes (Sections 4.1.2.2 and 10.7). If calcitonin results are not available, Visit 2 can proceed. A final review of eligibility will occur at Visit 3 before randomization can proceed.

The mental health questionnaires (Columbia-Suicide Severity Rating Scale [C-SSRS] and Self-Harm Form) should be completed after the assessment for AEs.

4.1.1.2.2. Treatment Period

Randomization (Visit 3)

At Visit 3, eligible participants will perform all required baseline study procedures (including the collection of all baseline laboratory measures and questionnaires) prior to randomization and prior to taking the first dose of study drug. Participants should arrive to the clinic in the fasting state (after a period of approximately 8 hours without eating, drinking [except water], or any significant physical activity). Patient-reported outcomes (PROs) questionnaires should be administered as early as possible, as per Suggested Visit Structure (Section 10.8). Preferred administration order is: Patient Global Impression of status for physical activity (PGIS), Short Form survey-36 version 2 (SF-36v2) acute form, Impact of Weight on Quality of Life-Lite-Clinical Trials Version (IWQOL-Lite-CT), and EQ-5D-5L. The mental health questionnaires (Patient Health Questionnaire-9 [PHQ-9], C-SSRS, and Self-Harm Form) should be completed after the assessment for AEs.

Participants will receive an initial consultation with a qualified dietician, according to local standards, to set lifestyle goals for caloric intake and physical activity (Sections 5.3.1 and 5.3.2).

Following randomization, study site personnel will demonstrate use of the autoinjector (also referred to as single-dose pen) using the provided demonstration device and observe the study participant inject the first dose of tirzepatide or placebo. The date, time, and location of the first dose of study drug will be recorded on the electronic case report form (eCRF). Beginning at randomization, all participants will receive study drug according to the randomized treatment arm for the duration of the 72-week treatment period including a 20-week dose escalation.

Participants will be provided diaries and be trained to record key study information, as appropriate.

Treatment Period (End of Visit 3 to Visit 21):

During the treatment period, office visits will occur monthly through 24 weeks and then every 12 weeks thereafter, to 72 weeks. The participant should be fasting for all office visits. Telephone visits will occur at 4-week intervals between the office visits starting at Week 28.

Office visit procedures should be conducted according to the Schedule of Activities (Section 1.3), and will include:

- weight, waist circumference, and vital signs measurements
- review of participant diary information (to include reinforcement and compliance assessments for study drug administration and lifestyle goals)
- administration of questionnaires
- laboratory testing
- drug dispensing
- collect AEs, product complaints and concomitant medications

Mental Health Questionnaires should be completed after the assessment of AEs.

Dietician consultations continue at Weeks 4, 8, 12, and then every 12 weeks thereafter, to Week 72. Study drug and injection supplies will be returned per the Schedule of Activities (Section 1.3) and according to local requirements. New supplies will be dispensed as needed.

Study drug dose escalation is double-blinded and will be managed via the interactive web-response system (IWRS). The starting dose is 2.5 mg QW (or matching placebo) for 4 weeks, then the dose is increased by 2.5 mg (or matching placebo) every 4 weeks: 2.5 to 5 mg for those randomized to 5 mg, 2.5 to 5 to 7.5 to 10 mg for those randomized to 10 mg, and continuing to 12.5 to 15 mg for those randomized to 15 mg. The dose is then maintained up to 72 weeks.

At each of the 8 scheduled telephone visits, procedures will include:

- reinforcement and compliance assessments for study drug administration and lifestyle goals,
- listing of concomitant medications
- AE and Product Complaints (PCs) assessment (see Schedule of Activities Section 1.3.1)
- administration of questionnaires

Participants should be instructed to contact the investigative site for assistance as soon as possible if they experience any difficulties administering their study medication. Participants should also be advised about the appropriate course of action in the event that study drug is not taken at the required time (late/missing doses).

Study participants will be permitted to use concomitant medications that they require during the study, except certain excluded medications (see Section 5.2) that may interfere with the assessment of efficacy and safety characteristics of the study treatments.

For those participants without glycemic evidence of T2DM (Section 4.1.2.1), a 2-hour oral OGTT will be performed at Visit 21.

Visit 99

Visit 99 is only applicable to participants who discontinue the study treatment prematurely (before Week 72). Participants will be asked to return for Visit 99 at 72 weeks ± 7 days after

randomization. This visit is critical to ensure complete data collection for the primary weight-loss endpoint.

Participants should attend this visit in the fasting state. Procedures to be completed are

- measurement of weight
- listing of concomitant medications
- assessment of AEs
- completion of the mental health questionnaires (after the AE assessment)

For participants unwilling to attend this visit, their refusal to attend should be documented in the participant medical record.

4.1.1.2.3. Additional 2-Year Treatment Period for Participants with Prediabetes at Randomization (Visits 101 through 116)

Participants classified as having prediabetes at randomization and who remain on study drug through Week 72, will continue follow-up for an additional 104 weeks (total study duration 176 weeks). Subsequent visits will occur approximately every 1.5 months, alternating between telephone and office visits.

Office visit procedures should be conducted according to the Schedule of Activities (Section 1.3), and will include:

- weight, waist circumference, and vital signs measurements
- review of participant diary information (to include reinforcement and compliance assessments for drug administration and lifestyle goals)
- administration of questionnaires
- laboratory testing
- drug dispensing
- collection of AEs, product complaints, and concomitant medications

Patient-reported outcomes questionnaires should be administered as early as possible, as per Suggested Visit Structure (Section 10.8). Mental Health Questionnaires should be completed after the assessment of AEs.

Dietician consultations continue approximately every 3 months after Visit 21 to Visit 116. At Visits 108 and 116, participants will undergo 2 hour OGTTs, unless diabetes had been diagnosed prior to the visits (Sections 4.1.2.2 and 10.7). Study drug and injection supplies will be returned per the Schedule of Activities (Section 1.3) and according to local requirements. New supplies will be dispensed as needed.

At each of the 8 scheduled telephone visits, procedures will include:

- reinforcement and compliance assessments for study drug administration and lifestyle goals
- listing of concomitant medications

- AE and PC assessment (see Schedule of Activities [Section 1.3])
- administration of questionnaires

Participants should be instructed to contact the investigative site for assistance as soon as possible if they experience any difficulties administering their study medication. Participants should also be advised about the appropriate course of action in the event that study drug is not taken at the required time (late/missing doses).

Visit 199

Visit 199 is only applicable to participants who discontinue the study treatment prematurely (between Week 72 and Week 176). Participants will be asked to return for Visit 199 at 176 weeks ± 7 days after randomization. This visit is critical to ensure complete data collection for the weight-loss and progression-to-diabetes endpoints.

Participants should attend this visit in the fasting state. Procedures to be completed are:

- measurement of weight
- measurement of waist circumference
- listing of concomitant medications
- assessment of AEs
- completion of the mental health questionnaires (after the AE assessment)
- 2-hour OGTT

For participants unwilling to attend this visit, their refusal to attend should be documented in the participant medical record.

4.1.1.2.4. Early Discontinuation of Treatment Visit

Participants unable or unwilling to continue the study treatment for any reason will perform an early discontinuation of treatment (ED) visit. If the participant is discontinuing during an unscheduled visit, that visit should be performed as the ED visit. If the participant is discontinuing during a scheduled visit, that visit should be performed as an ED visit. Procedures should be completed according to the Schedule of Activities (Section 1.3). Patient-reported outcomes questionnaires should be administered as early as possible, as per Suggested Visit Structure (Section 10.8). Administration of mental health questionnaires should follow assessment for AEs (see Section 7.2).

4.1.1.2.5. Safety Follow-up Period

Visit 801 or Visit 802

All participants are required to complete a safety follow-up visit (Visit 801 or Visit 802), according to the Schedule of Activities (Section 1.3). Participants discontinuing the study early and performing an ED visit will also be asked to perform the safety follow-up visit. During the safety follow-up period, participants will not receive study drug. Participants are also required to return any remaining study diaries to the study site at the end of this period.

For participants who discontinue or complete the study within the first 72 weeks, Visit 801 (safety follow-up visit) should be performed 4 weeks after the last treatment visit (Visit 21 or 99) or 4 weeks after the ED visit for those who decline to return for Visit 99. Participants with prediabetes continuing to Week 78 should not perform Visit 801.

For those participants without glycemic evidence of T2DM (Section 4.1.2.1), a 2-hour oral OGTT will be performed at Visit 802.

4.1.2. Study Procedures

Participants will perform study procedures listed in the Schedule of Activities (Section 1.3).

Participants will be permitted to use concomitant medications that they require during the study, except certain medications that may interfere with the assessment of efficacy and safety characteristics of the study treatments (Section 5.2).

Study governance considerations are described in detail in Section 10.1 (Appendix 1).

4.1.2.1. Definition of Prediabetes

The duration of treatment in Study GPHK (SURMOUNT-1) is determined by glycemic status at randomization, categorized into those with prediabetes or normoglycemia (participants with T2DM are excluded), as defined by the 2019 American Diabetes Association Standards of Medical Care in Diabetes (American Diabetes Association 2019b). The following populations are defined:

	**Normoglycemia**	**Prediabetes**	**Diabetes**
**Fasting glucose**<br>Obtained alone or at time = 0 during an OGTT	<100 mg/dL<br>(<5.6 mmol/L)	100-125 mg/dL<br>(5.6-6.9 mmol/L)	≥126 mg/dL<br>(≥7.0 mmol/L)
**2H glucose**<br>Obtained at time = 120 min during an OGTT	<140 mg/dL<br>(<7.8 mmol/L)	140-mg-199 mg/dL<br>(7.8-11.0 mmol/L)	≥200 mg/dL<br>(≥11.1 mmol/L)
**HbA1c**	<5.7%<br>(<39 mmol/mol)	5.7%-6.4%<br>(39-47 mmol/mol)	≥6.5%<br>(≥48 mmol/mol)

Abbreviations: H = hour; HbA1c = hemoglobin A1c; OGTT = 2-hour oral glucose tolerance test.

Screening to determine randomization glycemic status will occur in 2 visits:

Screening Visit 1

Participants will have a fasting glucose (FG) and HbA1c test. Results of these tests determine eligibility to proceed to Screening Visit 2.

- FG ≥126 mg/dL (7.0 mmol/L) OR HbA1c ≥6.5% (48 mmol/mol) suggests DM. These participants are ***excluded*** from the study and should be referred to their primary care physician for further work-up to confirm the diagnosis.

- All participants otherwise eligible and without FG or HbA1c values suggestive of diabetes will proceed to Screening Visit 2

Screening Visit 2

All participants attending Screening Visit 2 will undergo a 2-hour OGTT test. These results, in combination with those obtained from Screening Visit 1, will be used to determine study eligibility and randomization glycemic status.

- 2-hour OGTT results: FG ≥126 mg/dL (≥7.0 mmol/L) OR 2-hour OGTT ≥200 mg/dL (≥11.1 mmol/L) suggests diabetes mellitus. These participants are ***excluded*** from the study and should be referred to their primary care physician for further work-up to confirm the diagnosis.

Glycemic Classification

All participants without laboratory tests suggestive of diabetes will be classified as having either normoglycemia or prediabetes. In keeping with American Diabetes Association guidelines (American Diabetes Association 2019b), at least 2 abnormal tests are required to diagnose prediabetes. For example:

- Both 0 AND 2-hour values during the 2-hour OGTT values are in the prediabetes range.
- FG at Screening Visit 1 AND Visit 2 are in the prediabetes range.
- FG at Screening Visit 1 AND 2-hour values during 2-hour OGTT are in the prediabetes range.
- HbA1c AND 1 of either the FG or 2-hour OGTT values are in the prediabetes range.

4.1.2.2. Oral Glucose Tolerance Test

Participants should attend visits requiring a 2-hour OGTT in the fasting state. Samples will be collected at 0, 30, 60, 90, and 120 minutes during the 2-hour OGTT, unless the OGTT is repeated to confirm the diagnosis of diabetes. For glycemic classification, the values at time = 0 min (FG) and 120 minutes (2-hour OGTT) will be used. If a 2-hour OGTT is repeated to confirm the diagnosis of diabetes (see Section 4.1.2.3), only samples at 0 and 120 minutes for glucose measurement will be collected.

Two-hour OGTTs should be performed per the Schedule of Activities (Section 1.3) and as described in Section 10.7, until such time as a protocol-defined diagnosis of diabetes is confirmed (see Section 4.1.2.3). Two-hour OGTT testing should be omitted at visits following a protocol-defined diabetes diagnosis.

4.1.2.3. Definition and Management of Incident Diabetes

Definition of incident diabetes

Incident diabetes is defined when any 1 of the following occur after randomization (American Diabetes Association 2019b):

- unequivocal hyperglycemia (random glucose ≥200 mg/dL) with signs or symptoms of hyperglycemia

- any 2 of the following criteria are observed at the same visit, or 1 abnormal value is observed and subsequently confirmed:
  - HbA1c ≥6.5% (48 mmol/mol)
  - FG or 0-hour serum glucose from 2-hour OGTT ≥126 mg/dL (7.0 mmol/L)
  - 2-hour glucose ≥200 mg/dL (11.1 mmol/L) by a 2-hour OGTT
- initiation of any medication for the treatment of diabetes

Confirmation of diabetes diagnosis

In the event 1 abnormal value is observed (HbA1c ≥6.5% [48 mmol/mol]) OR fasting glucose (or 0-hour serum glucose from 2-hour OGTT) ≥126 mg/dL (7.0 mmol/L) OR 2-hour glucose from 2-hour OGTT ≥200 mg/dL (11.1 mmol/L)* after randomization, the abnormal test should be repeated within 4 weeks to confirm diagnosis of diabetes and to ensure that diabetes management is initiated without delay.

*If the abnormal value observed is 2-hour glucose from the OGTT ≥200 mg/dL (11.1 mmol/L), the 2-hour OGTT should be repeated within 4 weeks (only samples at 0 and 120 minutes for glucose measurement will be collected).

The diagnosis of diabetes is confirmed if any of the following occur:

- HbA1c ≥6.5% (48 mmol/mol) is observed at 2 measurements any time during the study.
- 2-hour glucose ≥200 mg/dL (11.1 mmol/L) during an OGTT is observed at 2 measurements any time during the study.
- FG value ≥126 mg/dL (7.0 mmol/L) is observed at a consecutive FG measurement (either at a scheduled or an unscheduled visit) following an isolated FG (or 0-hour serum glucose from 2-hour OGTT) value ≥126 mg/dL (7.0 mmol/L).
  - If diabetes diagnosis has not been confirmed at the consecutive FG measurement, another FG (or 0-hour serum glucose result from 2-hour OGTT) result ≥126 mg/dL (7.0 mmol/L) observed during the study will be considered as a new finding requiring confirmation.

Note*: Once diabetes has been confirmed, repeating any future abnormal test within 4 weeks is no longer required.*

Recording of incident diabetes events

- Diabetes diagnosis and the onset date as assessed by investigator will be recorded in the AE eCRF and the dedicated endpoint eCRF.
- If the diagnosis of diabetes is based on laboratory results, the date of the first abnormal HbA1c or glucose value within the diabetes range should be indicated as the date of diagnosis, unless, in the investigator's opinion, a different date is more appropriate. If the diagnosis is based on initiation of any medication for the treatment of diabetes, the investigator should indicate the most probable date of diagnosis in the eCRFs.

- All reported or suspected cases of incident diabetes will be adjudicated by an independent clinical endpoint committee (adjudication committee).
- Decisions of the adjudication committee regarding diabetes diagnosis and the onset date will be recorded in the dedicated adjudication eCRF.

Management of incident diabetes

Participants who develop diabetes during the study will be

- provided and trained to use a glucometer
- educated on the signs and symptoms of hypoglycemia and its treatment, and
- provided a diary to record hypoglycemic episodes per Section 10.3.1.1.

Participants will be referred to their usual care provider and provided with a letter showing the study results indicative of diabetes. The decision to further evaluate, to initiate antihyperglycemic therapy, and the choice of antihyperglycemic medication will be at the discretion of the participant's usual care provider, with the exception of use of dipeptidyl-peptidase-4 (DPP-4) inhibitors and open-label GLP-1R agonists, which are prohibited in the study. Monitoring for hypoglycemia includes capture of events as defined in Section 10.3 (Appendix 3).

4.2. Scientific Rationale for Study Design

Tirzepatide is a 39-amino acid synthetic peptide with agonist activity at both the GIPR and GLP-1R. Its structure is based on the GIP sequence and includes a C20 fatty di-acid moiety (Coskun et al. 2018). It is administered QW by SC injection.

As a dual GIP/GLP-1R agonist, tirzepatide could exceed the efficacy of selective GLP-1R agonists by recruiting metabolically active tissues not targeted by selective GLP-1R agonists (for example, adipose tissue as indicated by the observation of increased energy utilization) (Baggio and Drucker 2007) and has the potential to reach higher efficacy in target tissues, such as insulin-producing pancreatic beta cells that express both GIPR and GLP-1R, before reaching its therapeutic limitation. Results from a Phase 2 study (GPGB) demonstrated that tirzepatide use in participants with T2DM was associated with a substantial, dose-dependent weight loss, greater than the weight change observed with dulaglutide, a specific GLP-1R agonist. General safety characteristics of all studied doses of tirzepatide were similar to that of the GLP-1R agonist class, consisting mainly of nausea, vomiting, and diarrhea. In general, these events were transient and mild or moderate in severity, with few severe episodes. Although GI AEs were more common at the 15-mg arm of tirzepatide, this dose demonstrated the highest efficacy in terms of weight loss.

This suggests that tirzepatide has the potential to become a medication for chronic weight management. An optimized dose escalation regimen, proposed in the Study GPHK (SURMOUNT-1) to improve tolerability (and supported by a dose-escalation algorithm from study GPGF), should enable a use of higher doses to maximize effects on body weight. Study GPHK (SURMOUNT-1) is designed to determine the comparative benefits and risks of

tirzepatide 5 mg, 10 mg or 15 mg QW versus placebo in participants who have obesity or are overweight. A double-blind design was selected to minimize participant and investigator bias in assessments for efficacy, safety, and study drug tolerability.

A placebo comparator was selected for this trial in accordance with regulatory guidance (FDA 2007; EMA 2016). In addition, all participants, regardless of treatment assignment, will receive lifestyle modification counselling consistent with current guidelines for weight management (Jensen et al. 2013). Specifically, participants will consult with a dietician, or equivalent qualified delegate, throughout the study to achieve at least a 500 kcal/day energy deficit through a combination of caloric restriction and increased physical activity (Section 5.3.1). The planned duration of treatment for the primary endpoint at 72 weeks allows for at least a 52-week treatment period at the dose achieved following dose escalation to either 5, 10, or 15 mg. This duration is considered appropriate to assess the full effects and benefit/risk of each maintenance dose of tirzepatide compared with placebo on body weight and is consistent with regulatory guidelines (FDA 2007; EMA 2016).

The effects of drug cessation will be assessed in the 4-week and in the 12-week safety follow-up/observational periods

Another objective of the study is to evaluate the effect of tirzepatide on the risk of new-onset diabetes. Obesity is associated with an increased risk of developing T2DM and diabetes prevention is of a great importance in obese subjects to protect them from complications of the disease. Several studies have shown that interventions leading to weight loss may help prevent diabetes (Adams et al. 2017, le Roux et al. 2017).

To obtain additional information regarding the rate of conversion to diabetes on tirzepatide, participants with prediabetes diagnosed at the beginning of the study (being at increased risk of diabetes), will be treated and observed for an additional 2 years. Based on available literature, the 3 years of treatment with tirzepatide should be sufficient to demonstrate the risk reduction of developing T2DM compared to placebo. This additional treatment period would also permit collecting data on the durability of weight changes with long-term therapy and safety of long-term tirzepatide treatment.

To minimize the potential confounding effect of changes to concomitant medications, participants will be permitted to use concomitant medications that do not interfere with the assessment of efficacy or safety characteristics of the study treatments.

4.3. Justification for Dose

Tirzepatide doses of 5 mg, 10 mg, and 15 mg QW will be evaluated in this study. These doses and associated escalation schemes were selected based on assessment of safety, efficacy (weight loss), and GI tolerability data, followed by exposure-response modeling of data in patients in Phase 1 and 2 studies that predicted weight loss in obese and overweight patients without T2DM.

Dosing algorithms starting at a low dose of 2.5 mg accompanied by dose escalation of 2.5-mg increments every 4 weeks should permit time for development of tolerance to GI events and are predicted to minimize GI tolerability concerns. The maximum proposed dose of 15 mg maintains an exposure multiple of 1.6 to 2.4 to the no-observed adverse-effect level doses in 6-month monkey and rat toxicology studies.

4.4. End of Study Definition

Section 7.2 describes the criteria used to determine if a participant has completed the study.

The end of the study is defined as the date of the last visit of the last participant in the study or the last scheduled procedure shown in the Schedule of Activities (Section 1.3.1 or 1.3.2 based on randomization glycemic status), for the last participant in the trial globally.

5. Study Population

Prospective approval of protocol deviations to recruitment and enrollment criteria, also known as protocol waivers or exemptions, is not permitted.

5.1. Inclusion Criteria

Participants are eligible to be included in the study only if all of the following criteria apply:

Type of Participant and Disease Characteristics

1. Have a BMI

- $\geq$30 kg/m^2 or
- $\geq$27 kg/m^2 and previously diagnosed with at least one of the following weight related comorbidities:
    - hypertension: treated or with systolic blood pressure (SBP) $\geq$130 mmHg or diastolic blood pressure $\geq$80 mmHg
    - dyslipidemia: treated or with low-density lipoprotein (LDL) $\geq$160 mg/dL (4.1 mmol/L) or triglycerides $\geq$150 mg/dL (1.7 mmol/L), or high-density lipoprotein (HDL) <40 mg/dL (1.0 mmol/L) for men or HDL<50 mg/dL (1.3 mmol/L) for women
    - obstructive sleep apnea
    - cardiovascular disease (for example, ischemic cardiovascular disease, New York Heart Association (NYHA) Functional Class I-III heart failure)

2. Have a history of at least one self-reported unsuccessful dietary effort to lose body weight

3. In the investigator's opinion, are well-motivated, capable, and willing to
    - learn how to self-inject study drug, as required for this protocol (visually impaired persons who are not able to perform the injections must have the assistance of a sighted individual trained to inject study drug; persons with physical limitations who are not able to perform the injections must have the assistance of an individual trained to inject study drug)
    - inject study drug (or receive an injection from a trained individual if visually impaired or with physical limitations)
    - follow study procedures for the duration of the study, including, but not limited to: follow lifestyle advice (for example, dietary restrictions and exercise plan), maintain a study diary, and complete required questionnaires

Participant Characteristics

4. Are 18 years or older
    a. male participants:
        - Male participants with partners of childbearing potential should be willing to use reliable contraceptive methods throughout the study and for 5 half-lives of study drug plus 90 days, corresponding to 4 months after the last injection.
    b. female participants:
        - Female participants not of childbearing potential may participate and include those who are:
            - infertile due to surgical sterilization (hysterectomy, bilateral oophorectomy or tubal ligation), congenital anomaly such as Mullerian agenesis
            - postmenopausal, defined as either
                - a woman at least 40 years of age with an intact uterus, not on hormone therapy, who has cessation of menses for at least 1 year without an alternative medical cause, AND a follicle-stimulating hormone ≥40 mIU/mL; women in this category must test negative in pregnancy test prior to study entry

                or

                - a woman 55 or older not on hormone therapy, who has had at least 12 months of spontaneous amenorrhea

                or

                - a woman at least 55 years of age with a diagnosis of menopause prior to starting hormone replacement therapy
        - Female participants of child-bearing potential (not surgically sterilized and between menarche and 1-year postmenopausal) must:
            - test negative for pregnancy at Visit 1 based on a serum pregnancy test

            and

            - if sexually active, agree to use 2 forms of effective contraception, where at least 1 form is highly effective, for the duration of the trial and for 30 days thereafter
            - not be breastfeeding

Informed Consent

5. Have given written informed consent to participate in this study in accordance with local regulations and the ethical review board (ERB) governing the study site

5.2. Exclusion Criteria

Participants are excluded from the study if any of the following criteria apply:

Medical Conditions

Diabetes-related

6. Have type 1 diabetes mellitus (T1DM) or T2DM, history of ketoacidosis, or hyperosmolar state/coma
7. Have laboratory evidence diagnostic of diabetes mellitus during screening, including 1 or more of: HbA1c ≥6.5% (≥48 mmol/mol), FG ≥126 mg/dL (≥7.0 mmol/L), random glucose or 2-hour glucose measurement from a 2-hour OGTT ≥200 mg/dL (≥11.1 mmol/L)

Obesity-related

8. Have a self-reported change in body weight >5 kg within 3 months prior to screening
9. Have a prior or planned surgical treatment for obesity (excluding liposuction or abdominoplasty if performed >1 year prior to screening)
10. Have or plan to have endoscopic and/or device-based therapy for obesity or have had device removal within the last 6 months (for example, mucosal ablation, gastric artery embolization, intragastric balloon and duodenal-jejunal bypass sleeve)

Other medical

11. Have renal impairment measured as estimated glomerular filtration rate (eGFR) <30 mL/min/1.73 m^2, calculated by Chronic Kidney Disease-Epidemiology as determined by central laboratory during screening
12. Have a known clinically significant gastric emptying abnormality (for example, severe diabetic gastroparesis or gastric outlet obstruction) or chronically take drugs that directly affect GI motility
13. Have had a history of chronic or acute pancreatitis
14. Have thyroid-stimulating hormone (TSH) outside of the range of 0.4 to 6.0 mIU/L at screening visit

    Note: Patients receiving treatment for hypothyroidism may be included, provided their thyroid hormone replacement dose has been stable for at least 3 months.

    Note: TSH values above the normal range can, in some patients, suggest subclinical hypothyroidism. If, in the investigator's opinion, the participant has subclinical hypothyroidism and may require initiation of thyroid hormone replacement during the course of the study, the patient should be excluded from the study.
15. Have obesity induced by other endocrinologic disorders (for example, Cushing Syndrome) or diagnosed monogenetic or syndromic forms of obesity (for example, Melanocortin 4 Receptor deficiency or Prader Willi Syndrome)
16. Have a history of significant active or unstable Major Depressive Disorder (MDD) or other severe psychiatric disorder (for example, schizophrenia, bipolar disorder, or other serious mood or anxiety disorder) within the last 2 years

Note: Patients with MDD or generalized anxiety disorder whose disease state is considered stable and expected to remain stable throughout the course of the study, in the opinion of the investigator, may be considered for inclusion if they are not on excluded medications

17. Have any lifetime history of a suicide attempt

18. Have a Patient Health Questionnaire-9 (PHQ-9) score of 15 or more at Visit 1 or 3, prior to randomization

19. On the C-SSRS at Visits 1, 2, or 3, prior to randomization:

    - a "yes" answer to either Question 4 (Active Suicidal Ideation with Some Intent to Act, Without Specific Plan)
      or
    - a "yes" answer to Question 5 (Active Suicidal Ideation with Specific Plan and Intent) on the "Suicidal Ideation" portion of the C-SSRS
      or
    - a "yes" answer to any of the suicide-related behaviors (Actual Attempt, Interrupted Attempt, Aborted Attempt, Preparatory Act or Behavior) on the "Suicidal Behavior" portion of the C-SSRS
      and
    - the ideation or behavior occurred within the past month

20. Have uncontrolled hypertension (SBP above or equal to 160 mmHg and/or diastolic blood pressure above or equal to 100 mmHg)

21. Have any of the following cardiovascular conditions within 3 months prior to randomization: acute myocardial infarction (MI), cerebrovascular accident (stroke), unstable angina, or hospitalization due to congestive heart failure (CHF)

22. Have NYHA Functional Classification IV CHF

23. Have acute or chronic hepatitis, signs and symptoms of any other liver disease other than nonalcoholic fatty liver disease, or any of the following, as determined by the central laboratory during screening:

    - alanine aminotransferase (ALT) level >3.0X the upper limit of normal (ULN) for the reference range
      or
    - alkaline phosphatase (ALP) level >1.5X the ULN for the reference range
      or
    - total bilirubin (TBL) level >1.2X the ULN for the reference range (except for cases of known Gilbert's Syndrome)

    ***Note**:* Participants with nonalcoholic fatty liver disease <u>are eligible</u> to participate in this trial if their ALT level is ≤3.0X the ULN for the reference range

24. Have a calcitonin level (at Visit 1) of:

  - ≥20 ng/L at Visit 1, if eGFR ≥60 mL/min/1.73 m^2
  - ≥35 ng/L at Visit 1, if eGFR <60 mL/min/1.73 m^2

25. Have a family or personal history of medullary thyroid carcinoma (MTC) or Multiple Endocrine Neoplasia (MEN) Syndrome type 2

26. Have a history of an active or untreated malignancy or are in remission from a clinically significant malignancy (other than basal- or squamous-cell skin cancer, in situ carcinomas of the cervix, or in situ prostate cancer) for less than 5 years

27. Have any other condition not listed in this section (for example, hypersensitivity or intolerance) that is a contraindication to GLP-1R agonists

28. Have a history of any other condition (such as known drug or alcohol abuse, diagnosed eating disorder, or other psychiatric disorder) that, in the opinion of the investigator, may preclude the participant from following and completing the protocol

29. Have history of use of marijuana within 3 months of enrollment and unwillingness to abstain from marijuana use during the trial. Participants should also refrain from use of cannabidiol oil for the duration of the study

30. Have had a transplanted organ (corneal transplants [keratoplasty] allowed) or awaiting an organ transplant

31. Have any hematological condition that may interfere with HbA1c measurement (for example, hemolytic anemias, sickle cell disease)

Prior/Concomitant Therapy

32. Are receiving or have received within 3 months prior to screening chronic (>2 weeks or 14 days) systemic glucocorticoid therapy (excluding topical, intraocular, intranasal, intra-articular, or inhaled preparations) or have evidence of a significant, active autoimmune abnormality (for example, lupus or rheumatoid arthritis) that has required (within the last 3 months) or is likely to require, in the opinion of the investigator, concurrent treatment with systemic glucocorticoids (excluding topical, intraocular, intranasal, intra-articular or inhaled preparations) in the next 12 months

33. Have current or history of (within 3 months prior to randomization) treatment with medications that may cause significant weight gain, including but not limited to: tricyclic antidepressants, atypical antipsychotic and mood stabilizers for example:

    - imipramine
    - amitriptyline
    - mirtazapine
    - paroxetine
    - phenelzine
    - chlorpromazine
    - thioridazine

- clozapine
- olanzapine
- valproic acid and its derivatives
- lithium

Note: Selective serotonin reuptake inhibitors other than paroxetine are permitted.

34. Have taken within 3 months prior to randomization, medications (prescribed or over-the-counter) or alternative remedies intended to promote weight loss. Examples include, but are not limited to
    - Saxenda® (liraglutide 3.0 mg)
    - Xenical®/Alli® (orlistat)
    - Meridia® (sibutramine)
    - Acutrim® (phenylpropanolamine)
    - Sanorex® (mazindol)
    - Adipex® (phentermine)
    - BELVIQ® (lorcaserin)
    - Qsymia® (phentermine/topiramate combination)
    - Contrave® (naltrexone/bupropion)

    Note: Use of metformin or any other glucose-lowering medication, whether prescribed for polycystic ovary syndrome or diabetes prevention is not permitted.

35. Have started implantable or injectable contraceptives (such as Depo-Provera®) within 18 months prior to screening

Prior/Concurrent Clinical Study Experience

36. Are currently enrolled in any other clinical study involving an investigational product (IP) or any other type of medical research judged not to be scientifically or medically compatible with this study
37. Within the last 30 days, have participated in a clinical study and received treatment, whether active or placebo. If the study involved an IP, 5 half-lives or 30 days, whichever is longer, should have passed

Other Exclusions

38. Are investigator site personnel directly affiliated with this study and/or their immediate family. Immediate family is defined as a spouse, legal partner, parent, child, or sibling, whether biological or legally adopted.
39. Are Eli Lilly and Company (Lilly) employees

5.3. Lifestyle Considerations

Per the Schedule of Activities (Section 1.3), participants will consult with a dietician, or equivalent qualified delegate, according to local standards, to receive lifestyle management counseling at Weeks 0, 4, 8 and 12 during dose escalation and then at Week 24 and every 12

weeks thereafter through 72 weeks. Participants in the additional 2-year treatment period for participants with prediabetes at randomization will continue to receive lifestyle management counseling at 3-month intervals as defined in the Schedule of Activities (Section 1.3).

Diet and exercise goals established during the lifestyle consultation and the importance of adherence to the lifestyle component of the trial will be reinforced at each trial contact by study staff.

5.3.1. Meals and Dietary Restrictions

At Visit 3 and subsequent visits, study participants will receive diet counselling by a dietician/nutritionist, or equivalent qualified delegate, according to local standard. Dietary counseling will consist of advice on healthy food choices and focus on calorie restriction using a hypocaloric diet with macronutrient composition of:

- maximum 30% of energy from fat
- approximately 20% of energy from protein
- approximately 50% of energy from carbohydrates
- an energy deficit of approximately 500 kcal/day compared to the participant's estimated total energy expenditure (TEE)

To encourage adherence, it is recommended that a 3-day diet and exercise diary be completed prior to each counseling visit. During each visit, the participant's diet is reviewed and advice to maximize adherence is provided if needed.

The hypocaloric diet is continued after randomization and throughout the treatment period. If a BMI $\leq$22 kg/m^2 is reached, the recommended energy intake should be recalculated with no kcal deficit for the remainder of the trial.

Total energy expenditure (TEE) is calculated by multiplying the estimated Basal Metabolic Rate (BMR) (see table below) with a Physical Activity Level value of 1.3 (FAO/WHO/UNU 2004), which reflects an inactive lifestyle. This calculation provides a conservative estimate of caloric requirements:

TEE (kcal/day) = BMR X 1.3

Equations for estimating BMR in kcal/day*

Sex	Age	BMR (kcal/day)
Men	18-30 years	15.057 X actual weight in kg + 692.2
	31-60 years	11.472 X actual weight in kg + 873.1
	>60 years	11.711 X actual weight in kg + 587.7
Women	18-30 years	14.818 X actual weight in kg + 486.6
	31-60 years	8.126 X actual weight in kg + 845.6

>60 years	9.082 X actual weight in kg + 658.5

Abbreviations: BMR = basal metabolic rate; WHO = World Health Organization.
*Revised WHO equations (adapted from: FAO/WHO/UNU 2004).

5.3.2. Physical Activity

At Visit 3 and all subsequent visits, participants will be advised to increase their physical activity to at least 150 minutes per week.

5.4. Screen Failures

Screen failures are defined as participants who consent to participate in the clinical study but are not subsequently randomly assigned to the study intervention. A minimal set of screen-failure information should be collected to ensure transparent reporting of screen-failure participants to meet the Consolidated Standards of Reporting Trials (CONSORT) publishing requirements and to respond to queries from regulatory authorities. Minimal information includes demography, screen failure details, eligibility criteria, and any SAE.

Individuals who do not meet the criteria for participation in this study (screen failure) may not be rescreened.

6. Study Intervention

Study intervention is defined as any investigational intervention(s), marketed product(s), placebo, or medical device(s) intended to be administered to a study participant according to the study protocol.

6.1. Study Interventions Administered

**ARM Name**	Tirzepatide 5 mg	Tirzepatide 10 mg	Tirzepatide 15 mg	Placebo
**Dose**	5 mg QW	10 mg QW	15 mg QW	N/A
**Route of Administration**	SC	SC	SC	SC
**Sourcing**	Provided centrally by the Sponsor and dispensed via IWRS			
**Packaging and Labeling**	Study Intervention will be provided in autoinjectors (single-dose pens), packaged in cartons to be dispensed. Clinical study materials will be labeled according to country regulatory requirements.			

Abbreviations: IWRS = interactive web response service; N/A = not available; QW = once-weekly; SC = subcutaneous.

There are no restrictions on the time of day each weekly dose of study drug is given, but it is advisable to administer the SC injections on the same day and same time each week. The actual date, time and injection site location of all dose administrations will be recorded in the diary by the participant. If a dose of study drug is missed, the participant should take it as soon as possible unless it is within 72 hours of the next dose, in which case that dose should be skipped, and the next dose should be taken at the appropriate time. The day of weekly administration can be changed if necessary, as long as the last dose was administered 72 or more hours before.

All participants will inject study drug subcutaneously in the abdomen or thigh using the injection supplies provided; a caregiver may administer the injection in the participant's upper arm. The injection site location of all dose administrations will be recorded by the participant. A new autoinjector will be used for each injection. If study drug is to always be injected in the same body region, participants should be advised to rotate injection sites each week.

6.1.1. Medical Devices

The combination products provided for use in the study are tirzepatide investigational autoinjector (or matching placebo). Any medical-device incidents, including those resulting from malfunctions of the device, must be detected, documented, and reported by the investigator throughout the study (see Section 10.6).

6.2. Preparation/Handling/Storage/Accountability

- The investigator or designee must confirm appropriate temperature conditions have been maintained during transit for all study intervention received and any discrepancies are reported and resolved before use of the study intervention.
- Only participants enrolled in the study may receive study intervention and only authorized site staff may supply study intervention.
- All study intervention must be stored in a secure, environmentally controlled, and monitored (manual or automated) area in accordance with the labeled storage conditions with access limited to the investigator and authorized site staff.
- The investigator, institution, or the head of the medical institution (where applicable) is responsible for study intervention (includes study drug and autoinjector or single-dose pen) accountability, reconciliation, and record maintenance (for example, receipt, reconciliation, and final disposition of records).
- Study site staff must regularly assess whether the participant is correctly administering the assigned study drug and storing study drug according to the provided instructions.

6.3. Measures to Minimize Bias: Randomization and Blinding

This is a double-blind study.

Participants who meet all criteria for enrollment will be randomized to one of the study treatment groups at Visit 3. Assignment to treatment groups will be determined by a computer-generated, random sequence using an IWRS. Participants will be randomized in a 1:1:1:1 ratio to receive tirzepatide 5 mg, tirzepatide 10 mg, tirzepatide 15 mg, or placebo.

The randomization will be stratified by prediabetes status, country, and sex (female, male).

Investigators, site staff, clinical monitors and participants will remain blinded to the treatment assignments until the study is complete. To preserve the blinding of the study, a limited number of individuals from the Sponsor will be unblinded to treatment assignments as needed for the interim analysis and for the primary endpoint (Week 72) database lock (see Section 9.5).

Emergency unblinding may be performed through the IWRS. This option may be used ONLY if the participant's wellbeing requires knowledge of the participant's treatment assignment. All emergency unblinding events are recorded and reported by the IWRS.

If an investigator, site personnel performing assessments, or participant is unblinded, the participant must be discontinued from the study. In cases where there are ethical reasons to have the participant remain in the study, the investigator must obtain specific approval from a Lilly clinical research physician (CRP) for the participant to continue in the study.

In case of an emergency, the investigator has the sole responsibility for determining if unblinding of a participant's treatment assignment is warranted for medical management of the event. The participant safety must always be the first consideration in making such a determination. If a participant's treatment assignment is unblinded, Lilly must be notified immediately. If the investigator decides that unblinding is warranted, it is the responsibility of the investigator to promptly document the decision and rationale and notify Lilly as soon as possible.

6.4. Study Intervention Compliance

Participant compliance with study intervention will be assessed at each visit. Compliance will be assessed by direct questioning and counting of unused study drug and/or empty cartons returned. Study drug compliance will be determined by the following:

- Study drug administration data will be recorded by the participant and reviewed by the investigator at each study visit.
- The participants will be instructed to return any unused study drug and/or empty cartons at the next visit to the study site for the purpose of performing drug accountability.

Treatment compliance for each visit interval is defined as taking at least 75% of the required doses of study drug. Similarly, a participant will be considered significantly noncompliant if he or she is judged by the investigator to have intentionally or repeatedly taken more than the prescribed amount of medication (more than 125%).

In addition to the assessment of a participant's compliance with study drug administration, other aspects of compliance with the study treatments will be assessed at each visit based on the participant's adherence to the visit schedule, completion of study diaries, and any other parameters the investigator considers necessary.

Participants considered to be poorly compliant with their medication and/or the study procedures will receive additional training and instruction, as required, and will be reminded of the importance of complying with the protocol.

6.5. Concomitant Therapy

Participants will be permitted to use concomitant medications that they require during the study, except certain medications (for example; other medications for weight management) that may interfere with the assessment of efficacy and safety characteristics of the study treatments.

Participants who develop diabetes during the study may initiate medication for glucose control, with the exception of DPP-4 inhibitors or GLP-1R agonists (Section 4.1.2.3). Initiation of metformin for the treatment of diabetes is permitted, but metformin should not be initiated during the study for the treatment of other metabolic conditions (for example, polycystic ovary syndrome, diabetes prevention).

Investigative-site staff will inform participants that they must consult with the investigator or a designated site staff member upon being prescribed any new medications during the study. This may not be possible when initiated for treatment of medical emergencies, in which case, the participant will inform the investigator or a designated site staff member as soon as possible.

Nonstudy medications taken by participants who are screened but not randomized will not be reported to Lilly unless an SAE or AE occurs that the investigator believes may have been caused by a study procedure.

6.6. Dose Modification

6.6.1. Tirzepatide

Tirzepatide is given once weekly by SC injection. There are no restrictions on the time of day each weekly dose of tirzepatide is given, but it is advisable to administer the SC injections on the same day of the week and similar time each week. If a dose of tirzepatide is missed, the participant should take it as soon as possible unless it is within 72 hours of the next dose, in which case, that dose should be skipped, and the next dose should be taken at the appropriate time.

Study drug dose modification is not permitted, except for:

- management of intolerable GI symptoms during the entire course of the study (see Section 6.6.2) and,
- management of participants with perceived excessive weight loss during the additional 2-year treatment period for participants with prediabetes at randomization only (see Section 6.6.3).

Only 1 dose reduction per participant will be permitted during the entire course of the study. All dose adjustments will be managed through IWRS.

6.6.2. Management of Participants with Gastrointestinal Symptoms

Participants who experience intolerable GI symptoms (for example, nausea, vomiting, or diarrhea) at any time during the study, should first be counselled on dietary behaviors that may help mitigate nausea and vomiting, (for example, eating smaller meals, splitting 3 daily meals into 4 or more smaller ones, and stopping eating when they feel full). If symptoms persist, the participant should be prescribed, at the investigator's discretion, symptomatic medication (for example, antiemetic or antidiarrheal medication). A temporary interruption of study drug for 1 dose is permitted, provided the participant has taken the last 3 weekly doses. Study treatment should be resumed at the assigned dose immediately, either alone or in combination with symptomatic medication, which can also be utilized to manage symptoms (Section 10.9). Management of study drug after interruptions >1 dose is discussed in Section 7.1.1.

If intolerable GI symptoms (for example, nausea, vomiting, or diarrhea) persist despite the above measures, the investigator should contact Lilly to consider reinitiating study drug at the next-lowest maintenance dose in a blinded fashion (for example, 15 mg reduced to 10 mg, 10 mg reduced to 5 mg, or 5 mg reduced to placebo) (Section 10.9).

If intolerable GI symptoms persist despite symptomatic treatment, temporary drug interruption, and resumption at a lower dose of study drug, the participant should be discontinued from study drug. All participants who discontinue study drug should continue to attend scheduled study visits.

6.6.3. Management of Participants with Perceived Excessive Weight Loss During the Additional 2-year Treatment Period for Participants with Prediabetes at Randomization

As indicated in Section 5.3.1, if BMI ≤22 kg/m^2 is reached any time during the study, the recommended energy intake should be recalculated. From Visit 101 onwards, in the participants who have reached BMI ≤22 kg/m^2, if weight loss continues despite above measures and/or there are concerns related to degree of weight loss that may lead to discontinuation from study drug, the investigator should contact the Sponsor CRP to consider a study drug dose reduction in a blinded fashion (see Section 6.6.2 for details regarding study drug dose reduction).

6.7. Intervention after the End of the Study

6.7.1. Treatment after Study Completion

Tirzepatide will not be made available to participants after conclusion of the study.

7. Discontinuation of Study Intervention and Participant Discontinuation/Withdrawal

7.1. Discontinuation of Study Intervention

Possible reasons leading to permanent discontinuation of IP:

- **participant decision**
  - the participant requests to discontinue IP.
- **clinical considerations**
  - initiation of open-label GLP-1R agonist or DPP-4 inhibitor, if participants will not or cannot discontinue them
  - intolerable GI symptoms despite management as described in Section 6.6.2
  - BMI ≤18.5 kg/m^2 is reached at any time during the treatment period

    Note: The investigator should contact the Sponsor CRP to discuss whether it is medically appropriate for the participant to continue study treatment.
  - diagnosis of T1DM
  - diagnosis of MTC after randomization
  - significant elevation of calcitonin (Section 10.3.1.4)
  - diagnosis of acute or chronic pancreatitis
  - diagnosis of an active or untreated malignancy (other than basal or squamous cell skin cancer, in situ carcinomas of the cervix, or in situ prostate cancer) after randomization
  - development of any significant study drug-related hypersensitivity reaction
  - onset of pregnancy in a female participant
  - occurrence of any other treatment-emergent AE (TEAE), SAE, or clinically significant finding for which the investigator believes that permanent study drug discontinuation is the appropriate measure to be taken
  - inadvertent enrollment if continued treatment with study drug would not be medically appropriate (Section 7.2.3)
  - PHQ-9 score ≥15

        - Participants should be referred to a Mental Health Professional (MHP) to assist in deciding whether the subject should be discontinued from study drug. If a participant's psychiatric disorder can be adequately treated with psycho- and/or pharmacotherapy, then the subject, at the discretion of the Investigator (in agreement with the MHP), may be continued in the trial on randomized therapy.
    - in addition, study drug may be discontinued if participants:
        - answered "yes" to either Question 4 (Active Suicidal Ideation with Some Intent to Act, Without Specific Plan) on the "Suicidal Ideation" portion of the C-SSRS

        or

        - answered "yes" to Question 5 (Active Suicidal Ideation with Specific Plan and Intent) on the "Suicidal Ideation" portion of the C-SSRS

        or

        - answered "yes" to any of the suicide-related behaviors (Actual attempt, Interrupted attempt, Aborted attempt, Preparatory act or behavior) on the "Suicidal Behavior" portion of the C-SSRS.

Note: A psychiatrist or appropriately trained professional may assist in the decision to discontinue the participant.

- **discontinuation due to a hepatic event or liver test abnormality**

- Participants who are discontinued from IP due to a hepatic event or liver test abnormality should have additional hepatic safety data collected via eCRF.
- Discontinuation of the IP for abnormal liver tests **should be** considered by the investigator when a participant meets one of the following conditions after consultation with the Lilly designated medical monitor:
    - ALT or aspartate aminotransferase (AST) >8X ULN
    - ALT>2X baseline value OR ≥300U/L, whichever occurs first, if baseline ALT≥2X ULN
    - ALT or AST >5X ULN for more than 2 weeks
    - ALT or AST >3X ULN and total bilirubin level (TBL) >2X ULN or international normalized ratio (INR) >1.5
    - ALT or AST >3X ULN with the appearance of fatigue, nausea, vomiting, right upper-quadrant pain or tenderness, fever, rash, and/or eosinophilia (>5%)
    - alkaline phosphatase (ALP) >3X ULN
    - ALP >2.5X ULN and TBL >2X ULN

- ALP >2.5X ULN with the appearance of fatigue, nausea, vomiting, right-quadrant pain or tenderness, fever, rash, and/or eosinophilia (>5%)

Participants who stop study drug permanently should continue to attend all scheduled study visits to collect all planned efficacy and safety measurements. Participants who are unwilling to attend all scheduled visits and stop the study prior to 72 weeks, should return for a final weight measurement (Visit 99). If participants are unwilling to attend Visit 99, their refusal to attend should be documented in the patient medical record. Participants with prediabetes who stop study drug after 72 weeks should return for a final assessment of weight and glycemic status (Visit 199). Refusal to attend should be documented in the patient medical record.

Participants discontinuing from the IP prematurely for any reason should complete AE and other follow-up procedures per Section 1.3 (Schedule of Activities), Section 8.3 (Adverse Events and Serious Adverse Events), and Section 8.2 (Safety Assessments) of the protocol.

7.1.1. Temporary Study Drug Discontinuation

In certain situations, after randomization, the investigator may need to temporarily interrupt study drug. Every effort should be made by the investigator to maintain participants on study drug and to restart study drug after any temporary interruption, as soon as it is safe to do so. Distribution of study medication at the correct dose will be per IWRS instructions.

If study drug interruption is…	then…
2 consecutive doses or less	participant restarts study drug at last administered dose, as per escalation schedule.
3 consecutive doses or more	participant restarts study drug (at 5 mg, managed by IWRS) and repeats dose escalation scheme.
due to an AE	the event is to be documented and followed according to the procedures in Section 8.3 of this protocol.
due to intolerable persistent GI AE	participants should be treated as suggested in Section 6.6.2.

Abbreviations: AE = adverse event; GI = gastrointestinal; IWRS = interactive web response service.

Investigators should inform Lilly that study drug has been temporarily interrupted. The data related to temporary interruption of study treatment will be documented in source documents and entered on the eCRF.

7.2. Participant Discontinuation/Withdrawal from the Study

To minimize the amount of missing data and to enable assessment of study objectives as planned in the study protocol, every attempt will be made to keep participants in the study irrespective of the following:

- adherence to or discontinuation from study drug
- adherence to visit schedule
- missing assessments
- study drug discontinuation due to AE
- development of comorbidities
- development of clinical outcomes

The circumstances listed above are ***not*** valid reasons for discontinuation from the study.

Participants will be withdrawn from the study in the following circumstances:

- enrollment in any other clinical study involving an IP or enrollment in any other type of medical research judged not to be scientifically or medically compatible with this study
- participation in the study needs to be stopped for medical, safety, regulatory, or other reasons consistent with applicable laws, regulations, and good clinical practice (GCP)
- participant requests to be withdrawn from the study and clearly indicates that there will be no further contact of any kind with the site
- Female participants will be withdrawn from the study if the participant becomes pregnant.

Participants who agree to provide information relevant to any trial endpoint at the end of the study are not considered to have discontinued from the study.

Participants discontinuing from the study prematurely for any reason should complete AE and other safety follow-up per Section 1.3 (Schedule of Activities), Section 8.3 (Adverse Events and Serious Adverse Events), and Section 8.2 (Safety Assessments) of this protocol.

7.2.1. Participant Disposition and Timing of Safety Follow-up for the Primary Endpoint (72 Weeks)

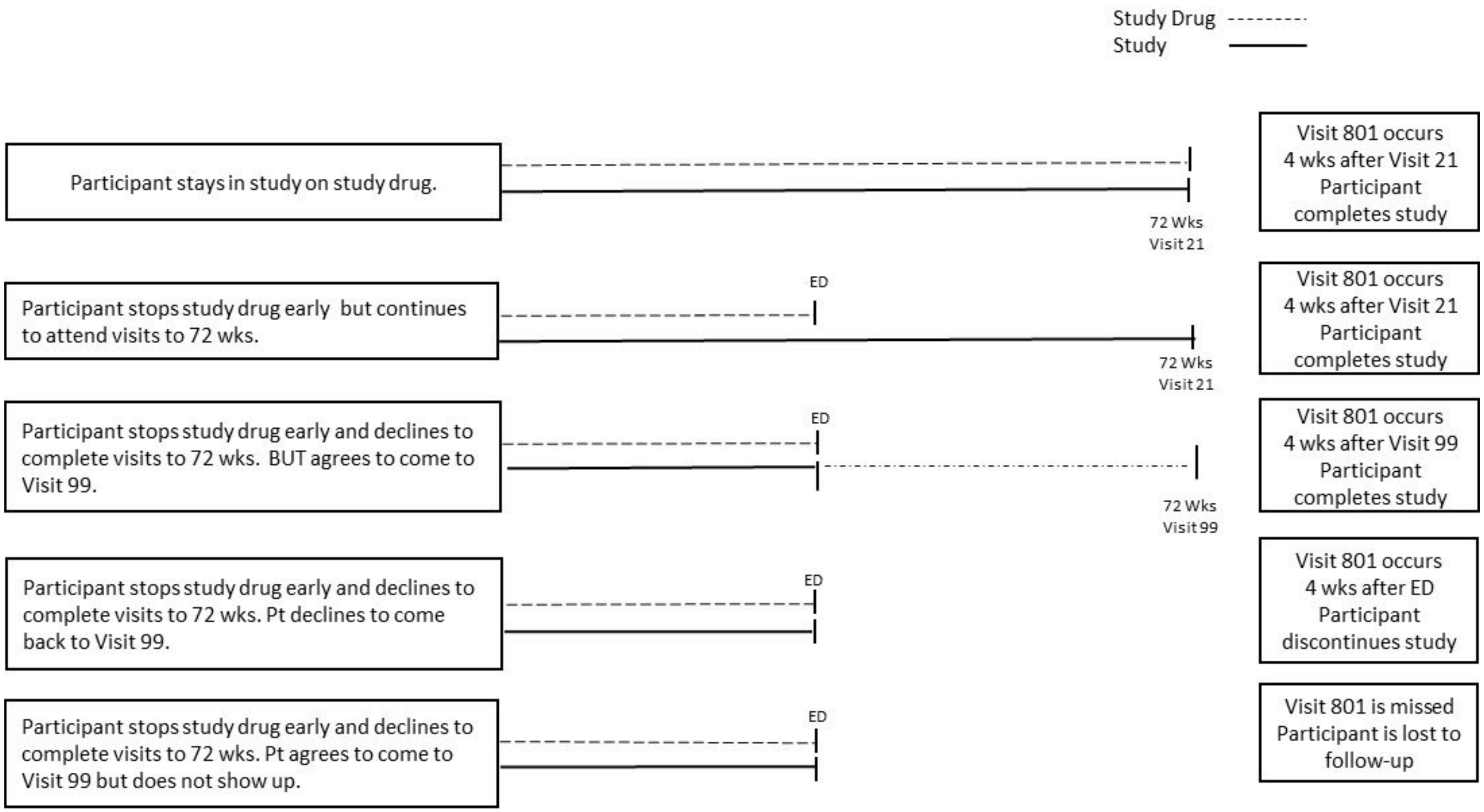

Abbreviations: ED = early discontinuation of treatment; Pt = participant; wks = weeks.

7.2.2. Participant Disposition and Timing of Safety Follow-up for those with Prediabetes at Randomization Continuing in Additional 2-year Treatment Period

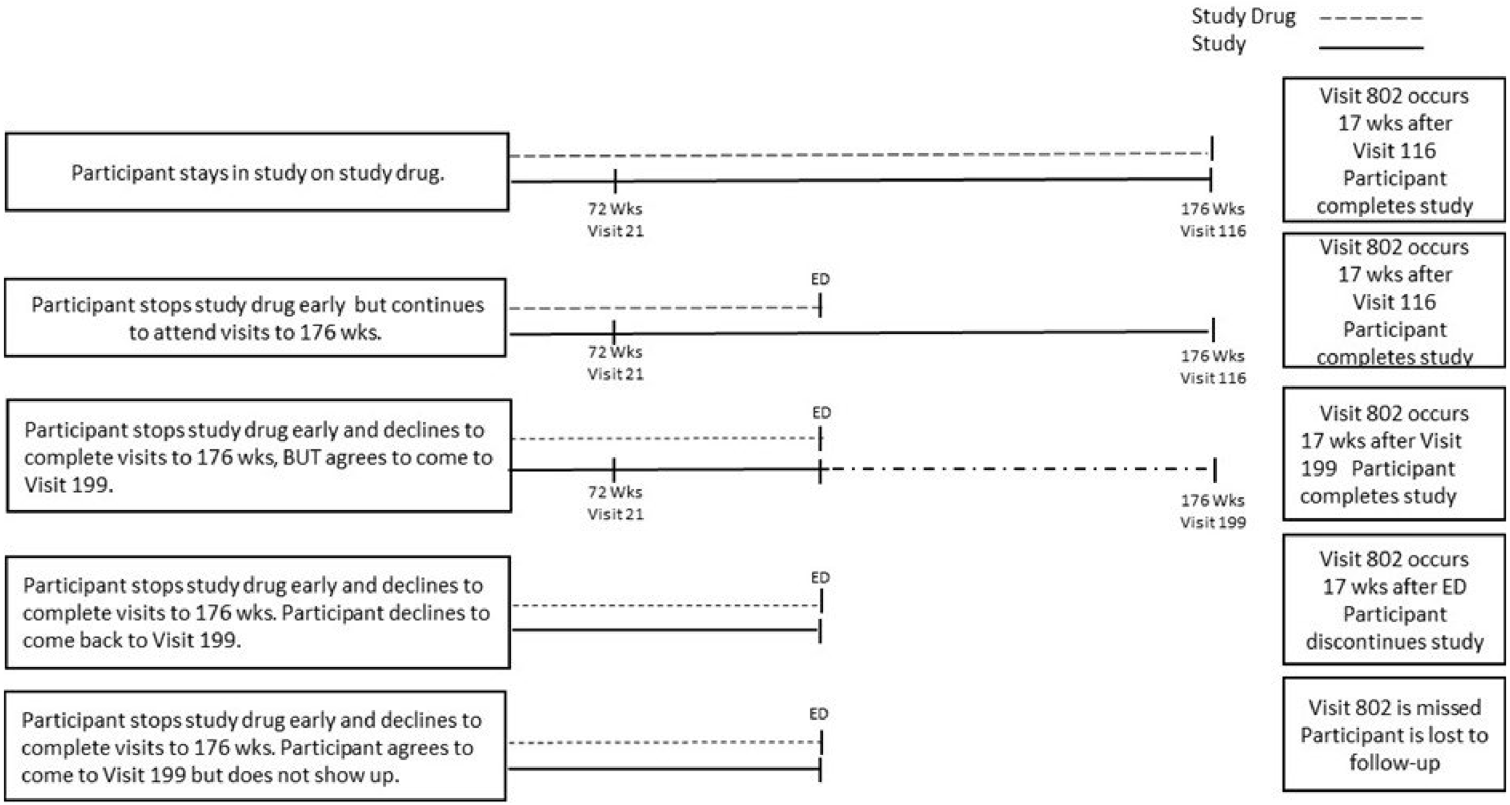

Abbreviations: ED = early discontinuation of treatment; wks = weeks.

Note: Participants with prediabetes at randomization who discontinue study drug on or before Week 72 are not eligible to continue in additional 2-year treatment period after Visit 21 (Week 72) and should proceed to V801.

7.2.3. Discontinuation of Inadvertently Enrolled Participants

If the Sponsor or investigator identify a participant who did not meet enrollment criteria and was inadvertently enrolled, the investigator and the Sponsor CRP must agree whether continuing the study treatment is medically appropriate. Continuation of inadvertently enrolled participants in the study, with or without study drug treatment, requires documented approval from the Sponsor CRP. Safety follow up is as outlined in Section 1.3 (Schedule of Activities), Section 8.3 (Adverse Events and Serious Adverse Events), and Section 8.2 (Safety Assessments) of the protocol.

7.3. Lost to Follow-up

A participant will be considered lost to follow-up if he or she repeatedly fails to return for scheduled visits and is unable to be contacted by the study site. Site personnel are expected to make diligent attempts to contact participants who fail to return for a scheduled visit or were otherwise unable to be followed up by the site.

Site personnel, or an independent third party, will attempt to collect the vital status of the participant within legal and ethical boundaries for all participants randomized, including those who did not get IP. Public sources may be searched for vital status information. If vital status is determined, this will be documented, and the participant will not be considered lost to follow-up.

Eli Lilly and Company personnel will not be involved in any attempts to collect vital status information.

Discontinuation of specific sites or of the study as a whole are handled as part of Section 10.1.9.

8. Study Assessments and Procedures

- Section 10.2 (Appendix 2) lists the laboratory tests that will be performed for this study. Repeat or unscheduled samples may be taken for safety reasons or for technical issues with the samples.
- Unless otherwise stated in the subsections below, all samples collected for specified laboratory tests will be destroyed within 60 days of receipt of confirmed test results. Certain samples may be retained for a longer period, if necessary, to comply with applicable laws, regulations, or laboratory certification standards.

8.1. Efficacy Assessments

8.1.1. Primary Efficacy Assessments

The primary efficacy assessment in this study is the body weight. Body weight measurements will be collected at specific clinic visits as summarized in the Schedule of Activities (Section 1.3). Methods for measuring body weight are described in Section 10.7 (Appendix 7).

8.1.2. Secondary Efficacy Assessments

The following secondary efficacy assessments will also be collected according to the Schedule of Activities:

- waist circumference (measuring method is described in Section 10.7 [Appendix 7]).
- fasting insulin (measured through central lab)
- onset of T2DM (definition is described Section 4.1.2.3)
- HbA1c (%, mmol/mol) (measured through central lab)
- fasting glucose (measured through central lab)
- BMI (derived using body weight in kilograms divided by the square of height in meters)
- blood pressure
- lipids

8.1.3. Patient-Reported Outcomes Assessments

The self-reported questionnaires will be translated into the native language of the region, linguistically validated and administered according to the Schedule of Activities (Section 1.3). At these visits, the questionnaires should be completed before the participant has discussed their medical condition or progress in the study with the investigator and/or site staff, if the participant is not adversely affected by their fasting condition.

8.1.4. Short Form 36 version 2, acute, 1 week recall version

The SF-36v2 acute, 1-week recall version is a 36-item, generic, patient-administered measure designed to assess the following 8 domains:

- Physical Functioning
- Role-Physical
- Bodily Pain
- General Health
- Vitality
- Social Functioning
- Role-Emotional
- Mental Health

The Physical-Functioning domain assesses limitations due to health "now" while the remaining domains assess functioning "in the past week." Each domain is scored individually and information from these 8 domains are further aggregated into 2 health-component summary scores: Physical-Component Summary and Mental-Component Summary. Items are answered on Likert scales of varying lengths (3-, 5-, or 6- point scales). Scoring of each domain and both summary scores are norm-based and presented in the form of T-scores, with a mean of 50 and standard deviation (SD) of 10; higher scores indicate better levels of function and/or better health (Maruish 2011).

8.1.5. Impact of Weight on Quality of Life-Lite Clinical Trials Version

The IWQOL-Lite-CT is a 20-item, obesity-specific PRO instrument developed for use in obesity clinical trials. It assesses 2 primary domains of obesity-related health-related quality of life (HRQoL): physical (7 items), and psychosocial (13 items). A 5-item subset of the physical domain, the physical-function composite is also supported. Items in the physical-function composite describe physical impacts related to general and specific physical activities. All items are rated on either a 5-point frequency ("never" to "always") scale or a 5-point truth ("not at all true" to "completely true") scale (Kolotkin et al. 2017, 2018).

8.1.6. EQ-5D-5L

Generic health-related quality of life will be assessed using the EQ-5D-5L (EQ-5D; EuroQoL Group 2015). The EQ-5D-5L is a standardized 5-item instrument for use as a measure of health outcome. It provides a simple descriptive profile and a single index value for health status that can be used in the clinical and economic evaluation of health care as well as population health surveys. The EQ-5D-5L comprises 5 dimensions of health (mobility, self-care, usual activities, pain/discomfort, and anxiety/depression). The 5L version, introduced in 2005, scores each dimension at 5 levels (no problems, slight problems, moderate problems, severe problems, unable to perform/extreme problems), for a total of 3125 possible health states. In addition to the health profile, a single health-state index value can be derived based on a formula that attaches weights to each of the levels in each dimension. This index value ranges between <0 (where 0 is

a health-state equivalent to death; negative values are valued as worse than dead) to 1 (perfect health) (Dolan 1997). In addition, the EQ Visual Analog Scale records the respondent's self-rated health status on a vertical graduated (0 to 100) scale. In conjunction with the health-state data, it provides a composite picture of the respondent's health status.

The EQ-5D-5L is used worldwide and is available in more than 170 different languages. Details on the instrument, and scoring, organizing, and presenting the data collected can be found in the EQ-5D-5L User Guide (EuroQoL Group 2015).

8.1.7. Patient Global Impression of Status for Physical Activity

Study participants will be asked to complete a PGIS item specifically developed for this study. This is a patient-rated assessment of current health limitation and is rated on a 5-point scale ranging from "1- not at all limited" to "5- extremely limited."

8.2. Safety Assessments

Planned time points for all safety assessments are provided in the Schedule of Activities (Section 1.3).

8.2.1. Physical Examinations

- A complete physical examination will include, at a minimum, assessments of the cardiovascular, respiratory, GI and neurological systems, as well as a thyroid exam. Height, weight, and waist circumference will also be measured and recorded, per Section 10.7.
- Investigators should pay special attention to clinical signs related to previous serious illnesses.

8.2.2. Vital Signs

For each participant, vital signs should be measured according to the Schedule of Activities (Section 1.3) and Section 10.7.

Any clinically significant findings from vital-signs measurement that result in a diagnosis and that occur after the participant receives the first dose of study treatment should be reported to Lilly or its designee as an AE via the eCRF.

8.2.3. Electrocardiograms

For each participant, 12-Lead electrocardiograms (ECGs) should be collected according to the Schedule of Activities (Section 1.3) and Section 10.7.

Electrocardiograms will initially be interpreted by a qualified physician (the investigator or qualified designee) at the site as soon after the time of ECG collection as possible, and ideally while the participant is still present, for immediate subject management, should any clinically relevant findings be identified. Any clinically significant findings from ECGs that result in a diagnosis and that occur after the participant receives the first dose of the investigational treatment should be reported to Lilly or its designee as an AE via the eCRF.

All digital ECGs will be obtained using centrally provided ECG machines and will be electronically transmitted to a designated central ECG laboratory. ECGs will be performed in triplicate at Weeks 0 and 36. The central ECG laboratory will perform a basic quality control check (for example, demographics and study details) and then store the ECGs in a database. At a future time, the stored ECG data may be overread by a cardiologist at the central ECG laboratory for further evaluation of machine-read measurements or to meet regulatory requirements. The machine-read ECG intervals and heart rate (HR) may be used for data analysis and report-writing purposes, unless a cardiologist overreading of the ECGs is conducted prior to completion of the final study report (in which case, the overread data would be used).

8.2.4. Clinical Safety Laboratory Assessments

- For each participant, laboratory tests detailed in (Section 10.2) should be conducted according to the Schedule of Activities (Section 1.3).
- With the exception of laboratory test results that may unblind the study, Lilly or its designee will provide the investigator with the results of laboratory tests analyzed by a central vendor, if a central vendor is used for the clinical trial.
- Any clinically significant findings from laboratory tests that result in a diagnosis and that occur after the participant receives the first dose of IP should be reported to Lilly or its designee as an AE via eCRF.

8.2.4.1. Immunogenicity Assessment

Where local regulations and ERBs allow, blood samples for immunogenicity testing will be collected to determine antibody production against tirzepatide as specified in the Schedule of Activities (Section 1.3).

At the visits and times specified in the Schedule of Activities (Section 1.3), venous blood samples will be collected to determine antibody production against tirzepatide. To interpret the results of immunogenicity, a PK sample will be collected at the same time points as the immunogenicity sample. All samples for immunogenicity should be taken predose when applicable and possible. In the event of systemic drug-hypersensitivity reactions (immediate or nonimmediate), additional samples will be collected (including antidrug antibodies [ADAs], PK, and exploratory biomarker samples) as close to the onset of the event as possible, at the resolution of the event, and 30 days following the event. Instructions for the collection and handling of blood samples will be provided by the Sponsor. The actual date and time (24-hour clock time) of each sampling will be recorded. Immunogenicity will be assessed by a validated assay designed to detect ADAs in the presence of tirzepatide at a laboratory approved by the Sponsor. Antibodies may be further characterized and/or evaluated for their ability to neutralize the activity of tirzepatide. Sample collected at Visit 801 or Visit 802 will assess immunogenicity at washout of tirzepatide (5 half-lives post end of treatment).

Samples with tirzepatide ADA detected will be titered and evaluated for their ability to neutralize the activity of assigned treatment (tirzepatide-neutralizing antibodies). Samples with tirzepatide ADA detected will also be tested for cross-reactive binding to native GIP and GLP-1, and if such is detected, then for neutralizing antibodies against native GIP and GLP-1, respectively.

Treatment-emergent ADAs are defined in Section 9.4.6.

Samples will be retained for a maximum of 15 years after the last participant visit, or for a shorter period if local regulations and ERBs allow, at a facility selected by the Sponsor. The duration allows the Sponsor to respond to future regulatory requests related to tirzepatide. Any samples remaining after 15 years will be destroyed.

8.2.5. Safety Monitoring

Lilly will periodically review evolving aggregate safety data within the study by appropriate methods. The study team will review safety reports in a blinded fashion according to the schedule provided in the Trial-Level Safety Review plan. Lilly will also review SAEs within time frames mandated by company procedures. The Lilly CRP will, as appropriate, consult with the functionally independent Global Patient Safety therapeutic area physician or clinical scientist.

8.2.5.1. Hepatic Safety Monitoring

If a study participant experiences elevated ALT ≥3X ULN, ALP ≥2X ULN, or elevated TBL ≥2X ULN, liver testing (Section 10.5) should be repeated within 3 to 5 days including ALT, AST, ALP, TBL, direct bilirubin, gamma-glutamyl transferase, and creatine kinase to confirm the abnormality and to determine if it is increasing or decreasing. If the abnormality persists or worsens, clinical and laboratory monitoring should be initiated by the investigator and in consultation with the study medical monitor. Monitoring of ALT, AST, TBL, and ALP should continue until levels normalize or return to approximate baseline levels.

Additional safety data should be collected via the eCRF if 1 or more of the following conditions occur:

- elevation of serum ALT to ≥5X ULN on 2 or more consecutive blood tests
- elevated serum TBL to ≥2X ULN (except for cases of known Gilbert's syndrome)
- elevation of serum ALP to ≥2X ULN on 2 or more consecutive blood tests
- participant discontinued from treatment due to a hepatic event or abnormality of liver tests
- hepatic event considered to be an SAE

8.2.6. Depression, Suicidal Ideation, and Behavior Risk Monitoring

Overweight and obese patients are at increased risk for depression (Luppino et al. 2010). Depression can increase the risk for suicidal ideation and behavior. Therefore, study participants will be screened at trial entry and monitored during the study for depression, and suicidal ideation and behavior.

Participants should be monitored appropriately and observed closely for suicidal ideation and behavior or any other unusual changes in behavior, especially at the beginning and end of the course of treatment, or at the time of dose changes, either increases or decreases. Consideration should be given to discontinuing study medication in subjects who experience signs of suicidal ideation or behavior, following a risk assessment (Section 7.1).

Baseline and treatment emergent assessment of depression, suicidal ideation and behavior will be monitored during the study using the C-SSRS and PHQ-9 (Section 8.3.1.1).

8.3. Adverse Events and Serious Adverse Events

Please refer to Section 10.3 Appendix 3 Adverse Events: Definitions and Procedures for Recording, Evaluating, Follow-up, and Reporting.

Investigators are responsible for monitoring the safety of participants who have entered this study and for alerting Lilly or its designee to any event that seems unusual, even if this event may be considered an unanticipated benefit to the participant.

The investigator is responsible for the appropriate medical care of participants during the study.

Investigators must document their review of each laboratory safety report.

The investigator remains responsible for following, through an appropriate health care option, AEs that are serious or otherwise medically important, considered related to the IP or the study, or that caused the participant to discontinue the IP before completing the study. The participant should be followed until the event resolves, stabilizes with appropriate diagnostic evaluation, or is reasonably explained. The frequency of follow-up evaluations of the AE is left to the discretion of the investigator.

Lack of drug effect is not an AE in clinical studies, because the purpose of the clinical study is to establish treatment effect.

After the ICF is signed, study site personnel will record via eCRF the occurrence and nature of each participant's preexisting conditions, including clinically significant signs and symptoms of the disease under treatment in the study. In addition, site personnel will record any change in the condition(s) and any new conditions as AEs. Investigators should record their assessment of the potential relatedness of each AE to protocol procedure and IP via eCRF.

The investigator will interpret and document whether or not an AE has a reasonable possibility of being related to study treatment, study device, or a study procedure, taking into account the disease, concomitant treatment, or pathologies.

A "reasonable possibility" means that there is a cause and effect relationship between the IP, study device, and/or study procedure and the AE.

The investigator answers yes/no when making this assessment.

Planned surgeries and nonsurgical interventions should not be reported as AEs unless the underlying medical condition has worsened during the course of the study.

If a participant's IP is discontinued as a result of an AE, study-site personnel must report this to Lilly or its designee via eCRF, clarifying if possible, the circumstances leading to any dosage modifications or discontinuations of treatment.

Serious Adverse Events

All AEs occurring after signing the ICF are recorded in the eCRF and assessed for serious criteria. An SAE is any AE from this study that results in one of the following outcomes:

- death
- initial or prolonged inpatient hospitalization
- a life-threatening experience (that is, immediate risk of dying)

- persistent or significant disability/incapacity
- congenital anomaly/birth defect
- important medical events that may not be immediately life-threatening or result in death or hospitalization but may jeopardize the participant or may require intervention to prevent 1 of the other outcomes listed in the definition above. Examples of such medical events include allergic bronchospasm requiring intensive treatment in an emergency room or at home, blood dyscrasias or convulsions that do not result in inpatient hospitalization, or the development of drug dependency or drug abuse.
- when a condition related to the drug delivery system necessitates medical or surgical intervention to preclude either permanent impairment of a body function or permanent damage to a body structure, the serious outcome of “required intervention” will be assigned.

All AEs occurring after signing the ICF are recorded in the eCRF and assessed for serious criteria. The SAE reporting to the Sponsor begins after the participant has signed the ICF and has received IP. However, if an SAE occurs after signing the ICF, but prior to receiving IP, the SAE should be reported to the Sponsor per SAE-reporting requirements and timelines (see Section 8.3.1) if it is considered reasonably possibly related to study procedure.

Study-site personnel must alert Lilly or its designee of any SAE within 24 hours of investigator awareness of the event via a sponsor-approved method. If alerts are issued via telephone, they are to be immediately followed with official notification on study-specific SAE forms. This 24-hour notification requirement refers to the initial SAE information and all follow-up SAE information. Participants with a serious hepatic AE should have additional data collected using the eCRF.

Pregnancy (during maternal or paternal exposure to IP) does not meet the definition of an AE. However, to fulfill regulatory requirements any pregnancy should be reported following the SAE process to collect data on the outcome for both mother and fetus.

Investigators are not obligated to actively seek AEs or SAEs in subjects once they have discontinued and/or completed the study (the participant disposition case report form [CRF] has been completed). However, if the investigator learns of any SAE, including a death, at any time after a subject has been discharged from the study, and he or she considers the event reasonably possibly related to the study treatment or study participation, the investigator must promptly notify Lilly.

Suspected Unexpected Serious Adverse Reactions

Suspected unexpected serious adverse reactions (SUSARs) are serious events that are not listed in the IB and that the investigator identifies as related to IP or procedure. United States 21 CFR 312.32, European Union Clinical Trial Directive 2001/20/EC, and the associated detailed guidance or national regulatory requirements in participating countries require the reporting of SUSARs. Lilly has procedures that will be followed for the identification, recording, and expedited reporting of SUSARs that are consistent with global regulations and the associated detailed guidance.

8.3.1. Time Period and Frequency for Collecting AE and SAE Information

All AE data will be collected from the signing of the ICF until the time points specified in the Schedule of Activities (Section 1.3).

Care will be taken not to introduce bias when detecting AEs and/or SAEs. Open-ended and nonleading verbal questioning of the participant is the preferred method to inquire about AE occurrences.

8.3.1.1. Adverse Event Monitoring with a Systematic Questionnaire

Before administering the C-SSRS or the PHQ-9, study site personnel will question the participant about any change in the preexisting condition(s) and the occurrence and nature of any AEs.

Nonserious AEs obtained through the questionnaire are recorded and analyzed separately. Only *serious* AEs elicited through the C-SSRS or PHQ-9 are to be recorded as AEs via the eCRF and reported to Lilly or its designee within 24 hours as SAEs. For this study, the C-SSRS is adapted for the assessment of the ideation and behavior categories only. The Intensity of Ideation and Lethality of Behavior sections are removed.

The C-SSRS is a scale that captures the occurrence, severity, and frequency of suicidal ideation and/or behavior during the assessment period. The scale includes suggested questions to solicit the type of information needed to determine if suicidal ideation and/or behavior occurred. The tool was developed by the National Institute of Mental Health trial group for the purpose of being a counterpart to the Columbia Classification Algorithm of Suicide Assessment categorization of suicidal events.

The PHQ-9 is a validated self-report screening tool that assesses the presence and intensity of depressive symptoms. The PHQ-9, which incorporates the 9 Diagnostic and Statistical Manual-IV depression criteria as “0” (not at all) to “3” (nearly every day), was developed for use in primary care settings (Kroenke et al. 2001).

8.3.2. Follow-up of AEs and SAEs

After the initial AE or SAE report, the investigator is required to proactively follow each participant at subsequent visits/contacts. All SAEs will be followed until resolution, stabilization, the event is otherwise explained, or the participant is lost to follow-up (as defined in Section 1.3).

8.3.3. Regulatory Reporting Requirements for SAEs

- Prompt notification by the investigator to the Sponsor of a SAE is essential so that legal obligations and ethical responsibilities towards the safety of participants and the safety of a study intervention under clinical investigation are met.
- The Sponsor has a legal responsibility to notify both the local regulatory authority and other regulatory agencies about the safety of a study intervention under clinical investigation. The Sponsor will comply with country-specific regulatory requirements relating to safety reporting to the regulatory authority, Institutional Review Boards (IRB)/Independent Ethics Committees (IEC), and investigators.

- Investigator safety reports must be prepared for SUSARs according to local regulatory requirements and sponsor policy and forwarded to investigators as necessary.
- An investigator who receives an investigator safety report describing an SAE or other specific safety information (for example, summary or listing of SAEs) from the Sponsor will review and then file it along with the IB and will notify the IRB/IEC, if appropriate according to local requirements.

8.3.4. Pregnancy

- Details of all pregnancies in female participants and female partners of male participants will be collected after the start of study intervention and the participant will be followed to determine the outcome of the pregnancy for mother and neonate.
- If a pregnancy is reported, the investigator should inform the Sponsor within 24 hours of learning of the pregnancy and should follow the procedures outlined in Section 10.4 (Appendix 4).
- Abnormal pregnancy outcomes (for example, spontaneous abortion, fetal death, stillbirth, congenital anomalies, and ectopic pregnancy) are considered SAEs.

8.3.5. Medical Device Incidents (Including Malfunctions)

Medical devices are being used in this study for the purposes of administering tirzepatide. In order to fulfill regulatory reporting obligations worldwide, the investigator is responsible for the detection and documentation of events meeting the definitions of incident or malfunction that occur during the study with such devices.

The definition of a medical device incident can be found in Section 10.6 (Appendix 6).

Note: Incidents fulfilling the definition of an AE/SAE will also follow the processes outlined in Section 8.3.2.

8.3.5.1. Time Period for Detecting Medical Device Incidents

- Medical device incidents, or malfunctions of the device that result in an incident, will be detected, documented, and reported to the Sponsor during all periods of the study in which the medical device is used.
- If the investigator learns of any incident at any time after a participant has been discharged from the study, and such incident is considered reasonably related to a medical device used during the study, the investigator will promptly notify the Sponsor through the complaints process.

The method of documenting medical device incidents is provided in Section 10.6 (Appendix 6).

8.3.5.2. Follow-up of Medical Device Incidents

- All medical device incidents involving an AE will be followed and reported in the same manner as other AEs (see Section 8.3.2). This applies to all participants, including those who discontinue study intervention.

- The investigator is responsible for ensuring that follow-up includes any supplemental investigations as indicated to elucidate the nature and/or causality of the incident.
- New or updated information will be recorded on the originally completed form with all changes signed and dated by the investigator.

8.3.5.3. Prompt Reporting of Medical Device Incidents and Malfunctions to Sponsor

- Medical device incidents or malfunctions will be reported to the Sponsor within a business day or as determined for the study, after the investigator determines that the event or issue meets the protocol definition of a medical device incident or malfunction.
- Medical device incidents or malfunctions that result in an SAE will be reported to the Sponsor within 24 hours, in the same manner as other (S)AEs.
- Malfunctions will be reported via the Product Complaint Form and sent to the Sponsor per training and per instructions provided on the Product Complaint Form.
- The same individual at the site will be the contact for the receipt of medical device reports and SAE.

8.3.5.4. Regulatory Reporting Requirements for Medical Device Incidents

- The investigator will promptly report to the Sponsor all incidents occurring with any medical device provided for use in the study in order for the Sponsor to fulfill the legal responsibility to notify appropriate regulatory authorities and other entities about certain safety information relating to medical devices being used in clinical studies.
- The investigator, or responsible person according to local requirements (for example; the head of the medical institution), will comply with the applicable local regulatory requirements relating to the reporting of incidents to the IRB/IEC.

8.3.6. Complaint Handling

Lilly collects product complaints on all IPs (drugs/medical devices, inclusive of packaging, labeling, instructions for use, or other component) used in clinical studies, inclusive of comparators, in order to ensure the safety of study participants, monitor product quality, and facilitate process and product improvements.

Participants will be instructed to contact the investigator as soon as possible if he or she has a complaint or problem with the investigational drug or medical device, so that the situation can be assessed.

Investigators will provide complete and timely reports to the Sponsor as instructed for the study, and per the Product Complaint Form.

8.4. Treatment of Overdose

Study drug overdose (more than the specified number of injections) will be reported as an AE. In the event of overdose, refer to the IB for tirzepatide.

8.5. Pharmacokinetics

Pharmacokinetic samples will be collected from all randomized participants.

Plasma tirzepatide concentrations will only be determined from blood samples obtained from participants receiving tirzepatide treatment. Blood samples for PK assessment will be collected at Weeks 8, 16, and 36 following tirzepatide treatment per the Schedule of Activities or at ED (Section 1.3). At each visit prior to a PK assessment, each participant will be assigned via IWRS to one of the sampling PK time windows of 1 to 24 hours, 24 to 96 hours, or 120 to 168 hours postdose. In addition, plasma tirzepatide concentration will be determined on immunogenicity visits up to and including Week 176.

The date and time of the most recent tirzepatide SC injection administered prior to collecting the PK sample must be recorded on the eCRF from the study diaries.

The date and time at which each sample was drawn must be recorded on the laboratory accession page.

Concentrations of tirzepatide will be assayed using a validated liquid chromatography mass spectrometry method.

Bioanalytical samples collected to measure tirzepatide concentration will be retained for a maximum of 1 year following last participant visit for the study. During this time, samples remaining after the bioanalyses may be used for exploratory analyses such as metabolism work, protein binding, and/or bioanalytical method cross-validation.

8.6. Pharmacodynamics

Samples to assess the PD properties of tirzepatide are included in the efficacy measures and not applicable in this section.

8.7. Genetics

8.7.1. Whole Blood Sample(s) for Pharmacogenetic Research

A whole blood sample will be collected for pharmacogenetic analysis as specified in the Schedule of Activities (Section 1.3) where local regulations allow.

Samples will not be used to conduct unspecified disease or population genetic research either now or in the future. Samples will be used to investigate variable response to tirzepatide and to investigate genetic variants thought to play a role in obesity. Assessment of variable response may include evaluation of AEs or differences in efficacy.

All samples will be coded with the participant number. These samples and any data generated can be linked back to the patient only by the investigator site personnel.

Samples will be retained at a facility selected by Lilly or its designee for a maximum of 15 years after the last participant visit for the study, or for a shorter period if local regulations and/or ERBs/ IRBs impose shorter time limits. This retention period enables use of new technologies, response to regulatory questions, and investigation of variable response that may not be observed until later in the development of tirzepatide or after tirzepatide become(s) commercially available.

Molecular technologies are expected to improve during the 15-year storage period and therefore cannot be specifically named. However, existing approaches include whole genome or exome sequencing, genome wide association studies, and candidate gene studies. Regardless of technology utilized, genotyping data generated will be used only for the specific research scope described in this section.

8.8. Biomarkers

Biomarker research is performed to address questions of relevance to drug disposition, target engagement, PD, mechanism of action, variability of participant response (including safety), and clinical outcome. Sample collection is incorporated into clinical studies to enable examination of these questions through measurement of biomolecules including deoxyribonucleic acid (DNA), proteins, lipids, and other cellular elements.

Serum, and plasma samples for biomarker research will be collected at the times specified in the Schedule of Activities (Section 1.3) where local regulations allow.

Samples will be used for research on the drug target, disease process, variable response to tirzepatide, pathways associated with obesity, mechanism of action of tirzepatide, and/or research method or in validating diagnostic tools or assay(s) related to obesity.

All samples will be coded with the participant number. These samples and any data generated can be linked back to the participant only by the investigator site personnel.

Samples will be retained at a facility selected by Lilly or its designee for a maximum 15 years after the last participant visit for the study, or for a shorter period if local regulations and ERBs/IRBs impose shorter time limits. This retention period enables use of new technologies, response to regulatory questions, and investigation of variable response that may not be observed until later in the development of tirzepatide or after tirzepatide become(s) commercially available.

8.9. Health Economics

Health Economics and Medical Resource Utilization Parameters are not evaluated in this study.

9. Statistical Considerations

9.1. Statistical Hypotheses

The alternative hypotheses for the primary objective are the following:

- $H_{10,1}$: QW tirzepatide 10 mg is superior to placebo for percent change in body weight from randomization AND percentage of participants who achieve ≥5% body weight reduction at 72 weeks.
- $H_{15,1}$: QW tirzepatide 15 mg is superior to placebo for percent change in body weight from randomization AND percentage of participants who achieve ≥5% body weight reduction at 72 weeks

The above two hypotheses will be tested in parallel, each at a 2-sided significance level of 0.025.

The alternative hypotheses for the key secondary objective controlling for type 1 error rate are the following:

- $H_{15,2}$ and $H_{10,2}$: superiority of tirzepatide 10 mg and/or 15 mg versus placebo for percentage of participants who achieve ≥10% body weight reduction from randomization at 72 weeks
- $H_{15,3}$ and $H_{10,3}$: superiority of tirzepatide 10 mg and/or 15 mg versus placebo for percentage of participants who achieve ≥15% body weight reduction from randomization at 72 weeks
- $H_{15,4}$ and $H_{10,4}$: superiority of tirzepatide 10 mg and/or 15 mg versus placebo for percentage of participants who achieve ≥20% body weight reduction from randomization at 72 weeks
- $H_{15,5}$ and $H_{10,5}$: superiority of tirzepatide 10 mg and/or 15 mg versus placebo for change from randomization in waist circumference (cm) at 72 weeks
- $H_{1015,6}$: superiority of pooled tirzepatide 10 mg and 15 mg versus placebo for change in body weight (kg) from randomization at 20 weeks
- $H_{5,1}$: superiority of tirzepatide 5 mg versus placebo for percent change in body weight from randomization **and** percentage of participants who achieve ≥5% body weight reduction at 72 weeks
- $H_{p,7}$: superiority of tirzepatide (all doses combined) versus placebo for change in systolic blood pressure (SBP) (mmHg) from randomization at 72 weeks
- $H_{p,8}$: superiority of tirzepatide (all doses combined) versus placebo for change in triglycerides (mg/dL) from randomization at 72 weeks
- $H_{p,9}$: superiority of tirzepatide (all doses combined) versus placebo for change in non-high-density lipoprotein cholesterol (non-HDL-cholesterol) (mg/dL) from randomization at 72 weeks

- $H_{p,10}$: superiority of tirzepatide (all doses combined) versus placebo for change in high-density lipoprotein cholesterol (HDL-cholesterol) (mg/dL) from randomization at 72 weeks
- $H_{p,11}$: superiority of tirzepatide (all doses combined) versus placebo for change in fasting insulin (pmol/L) from randomization at 72 weeks
- $H_{1015,12}$: superiority of pooled tirzepatide 10 mg and 15 mg versus placebo for change in SF-36v2 acute form Physical Functioning domain score from randomization at 72 weeks.
- $H_{15,13}$ and $H_{10,13}$: superiority of tirzepatide 10 mg and/or 15 mg versus placebo for change in percent body weight from randomization at 176 weeks (for participants with prediabetes at randomization).
- $H_{p,14}$ and $H_{p,15}$: superiority of tirzepatide (all doses combined) versus placebo for delaying the onset of type 2 diabetes mellitus (T2DM) during 176 weeks or 193 weeks (for participants with prediabetes at randomization).

The details of family-wise type I error rate control strategy and methods for the aforementioned hypotheses will be provided in the statistical analysis plan (SAP).

9.2. Sample Size Determination

Approximately 3429 participants will be screened to achieve 2400 randomly assigned to study intervention (600 participants per intervention group).

The sample size determination assumes that evaluation of superiority of 10-mg tirzepatide and tirzepatide 15 mg to placebo will be conducted in parallel, each at a 2-sided significance level of 0.025 using a 2-sample t-test. Additionally, a difference of at least 11% mean body weight percentage reduction from randomization at 72 weeks for 10-mg tirzepatide and/or tirzepatide 15 mg compared with placebo, a common SD of 10%, and a dropout rate of 25% are assumed for statistical power calculations. Under the assumptions above, randomizing 2400 participants in a 1:1:1:1 ratio to tirzepatide 5 mg (600), tirzepatide 10 mg (600), tirzepatide 15 mg (600), and placebo (600) provides more than 90% power to demonstrate superiority of tirzepatide 10 mg and/or 15 mg to placebo.

The chosen sample size and randomization ratio also provides >90% power to establish superiority of 10-mg tirzepatide and 15-mg tirzepatide doses to placebo in term of percentage of participants achieving at least 5% body weight reduction at 72 weeks, conducted in parallel using a Fisher's exact test, each at a 2-sided significance level of 0.025, assuming 25% placebo-treated participants and 90% tirzepatide-treated participants achieving the goal and a dropout rate of 25%.

In addition, assuming that approximately 60% of the randomized population will have prediabetes, the study sample size will also provide more than 90% power to demonstrate superiority of tirzepatide (all doses combined) over placebo in terms of delaying the onset of diabetes for participants with prediabetes at study entry. It is based on the following assumptions: 1.6% (corresponding to annual hazard rate of 0.54%) of participants randomized to tirzepatide and 6% of participants randomized to placebo (corresponding to annual hazard rate of

2.1%) will progress to diabetes during the 3-year period; 49% of participants will drop out (corresponding to annual drop-out rate of 22%) during the same period; and the test will be conducted at a 2-sided significance level of 0.05.

9.3. Populations for Analyses

For purposes of analysis, the following analysis sets are defined:

Analysis Set	Description
Entered Participants	all participants who sign informed consent
Randomized Participants	all participants who are randomly assigned a study treatment
Modified Intent-to-Treat (mITT)	all randomly assigned participants who are exposed to at least 1 dose of study drug; participants will be included in the treatment group they were randomized to
Efficacy Analysis Set (EAS)	data obtained during treatment period from mITT, excluding data after discontinuation of study drug (last dose date + 7 days)
Full analysis set (FAS)	data obtained during treatment period from mITT, regardless of adherence to study drug.
Safety Analysis Set (SS)	data obtained during the treatment period plus safety follow-up period from mITT, regardless of adherence to study drug

9.4. Statistical Analyses

9.4.1. General Statistical Considerations

Statistical analysis of this study will be the responsibility of Lilly or its designee. Any change to the data analysis methods described in the protocol will require an amendment ONLY if it changes a principal feature of the protocol. Any other change to the data analysis methods described in the protocol, and the justification for making the change, will be described in the SAP or the Clinical Study Report (CSR). Additional exploratory data analyses may be conducted as deemed appropriate.

Unless otherwise noted, all tests of treatment effects will be conducted at a 2-sided alpha level of 0.05, and the confidence interval will be calculated at 95%, 2-sided. In statistical summaries and analyses, data will be analyzed as randomized.

Unless specified otherwise, efficacy and safety will be assessed using the modified intention-to-treat (mITT) population. Baseline will be defined as the last available non-missing measurement during Visit 1 to Visit 3. Unless specified otherwise, safety assessments will be guided by an estimand comparing safety of tirzepatide doses with placebo irrespective of adherence to study drug. Thus, safety analysis will be conducted using safety analysis set (SS).

Summary statistics for continuous measures will include sample size, mean, SD, median, minimum, and maximum. The analysis model to make comparisons among treatment groups relative to continuous measurements assessed over time will include a mixed model for repeated measures (MMRM), with terms of treatment, visit, and treatment-by-visit interaction, stratification factors, and baseline measurement as a covariate.

Kaplan-Meier method will be used for estimation of cumulative event-free survival rates over time, and Cox proportional hazards regression analysis will be used to compare hazard rates among treatments.

Summary statistics for categorical measures (including categorized continuous measures) will include sample size, frequency, and percentages. Logistic regression will be used to examine the treatment difference in binary efficacy outcomes if there is a need to adjust for covariates.

Otherwise, Fisher's exact test will be used to examine the treatment difference in categorical outcomes.

Summary statistics for discrete count measures will include sample size, mean, SD, median, minimum, and maximum. The negative binomial regression model will be used for the treatment comparison of discrete count measures.

Other statistical methods may be used, as appropriate, and details will be described in the SAP.

9.4.2. Treatment Group Comparability

9.4.2.1. Participant Disposition

A detailed description of participant disposition will be provided at the end of the study.

Frequency counts and percentages of all participants screened, randomized, and receiving at least 1 dose of study drug will be presented by treatment groups. Of the participants in the mITT population, frequency counts and percentages of participants who completed the study, prematurely discontinued the study (and/or study drug), including reason for premature discontinuation, will be presented by treatment groups.

A Kaplan-Meier analysis of time from randomization to premature discontinuation from study by treatment group will be provided.

9.4.2.2. Participant Characteristics

Demographics and other baseline characteristics (including years of education) will be summarized by treatment group for all randomized participants.

9.4.2.3. Concomitant Therapy

Concomitant medications, including previous therapy, will be summarized by treatment arm for SS.

9.4.2.4. Treatment Compliance

Frequency counts and percentages of participants compliant to study drug will be summarized by treatment groups and visits for full analysis set (FAS).

9.4.3. Efficacy Analyses

9.4.3.1. Primary Analyses

There will be 2 estimands of interest in comparing efficacy of tirzepatide doses with placebo.

- For objectives controlled for Type 1 error at 72 weeks:
    1) The "efficacy" estimand, defined as the average treatment effect of tirzepatide relative to placebo at 72 weeks, as an adjunct to a reduced-calorie diet and increased physical activity, in the randomized participants had they remained on their randomized treatment for the entire planned 72 weeks treatment duration.
    2) The "hybrid" estimand, defined as the average treatment effect of tirzepatide relative to placebo at 72 weeks, as an adjunct to a reduced-calorie diet and increased physical activity, for the randomized participants regardless of the adherence to study drug.

- For objectives controlled for Type 1 error assessed after 72 weeks:
  1) The "efficacy" estimand, defined as the treatment effect of tirzepatide relative to placebo at 176/193 weeks, as an adjunct to a reduced-calorie diet and increased physical activity, in the randomized participants with prediabetes at randomization had they remained on their randomized treatment for the entire planned 176 weeks treatment duration.
  2) The "hybrid" estimand, defined as the average treatment effect of tirzepatide relative to placebo at 176/193 weeks, as an adjunct to a reduced-calorie diet and increased physical activity, for the randomized participants with prediabetes at randomization regardless of the adherence to study drug.

For the Food and Drug Administration, the primary efficacy analysis will be guided by the hybrid estimand and conducted using the FAS. This assessment will analyze percent change in body weight obtained at the 72-week visit using an analysis of covariance (ANCOVA) and the percentage of participants achieving at least 5% body weight reduction obtained at the 72-week visit using a logistic regression model. Both models will include terms of treatment, stratification factors, and baseline body weight as a covariate. Missing data at the 72-week visit will be imputed and analysis will be conducted with multiple imputations (see details in the SAP). Since the mean percent change in body weight and percentage of participants with ≥5% body weight reduction objectives need to be achieved at the same time, no multiplicity adjustment is planned for these 2 tests.

For all other purposes, the primary efficacy analysis will be guided by the "efficacy" estimand and conducted using the efficacy analysis set (EAS). The primary analysis model will be an MMRM for body weight percent change over time and logistic regression for percentage of participants achieving at least 5% body weight reduction over time. Details of the statistical modeling will be provided in the SAP.

9.4.3.2. Key Secondary Analyses

- superiority of pooled tirzepatide 10 mg and 15 mg to placebo for change in body weight (kg) from randomization at 20 weeks visit
- superiority of QW tirzepatide 5 mg to placebo for percent change in body weight from randomization AND percentage of participants who achieve ≥5% body weight reduction at 72 weeks visit
- superiority of each tirzepatide dose (10 mg and 15 mg) to placebo for the percentage of study participants who achieve ≥10% body weight reduction at 72 weeks visit
- superiority of each tirzepatide dose (10 mg and 15 mg) to placebo for the percentage of study participants who achieve ≥15% body weight reduction at 72 weeks visit
- superiority of each tirzepatide dose (10 mg and 15 mg) to placebo for the percentage of study participants who achieve ≥20% body weight reduction at 72 weeks visit
- superiority of each tirzepatide dose (10 mg and 15 mg) to placebo for change from randomization in waist circumference (cm) at 72 weeks visit

- superiority of pooled tirzepatide 10 mg and 15 mg to placebo for change from randomization in SF-36v2 acute form physical functioning domain score at 72 weeks visit
- superiority of tirzepatide (all doses combined) to placebo for change from randomization in triglycerides (mg/dL) at 72 weeks visit
- superiority of tirzepatide (all doses combined) to placebo for change from randomization in non-HDL-cholesterol (mg/dL) at 72 weeks visit
- superiority of tirzepatide (all doses combined) to placebo for change from randomization in HDL-cholesterol (mg/dL) at 72 weeks visit
- superiority of tirzepatide (all doses combined) to placebo for change from randomization in SBP (mmHg) at 72 weeks visit
- superiority of tirzepatide (all doses combined) to placebo for change from randomization in fasting insulin (pmol/L) at 72 weeks visit
- superiority of each tirzepatide dose (10 mg and 15 mg) to placebo for change from randomization in percent change of body weight at 176 weeks visit (for participants with prediabetes at randomization)
- superiority of tirzepatide (all doses combined) to placebo in delaying the onset of diabetes during 176 weeks treatment period (for participants with prediabetes at randomization)
- superiority of tirzepatide (all doses combined) to placebo in delaying the onset of diabetes during 193 weeks study period (for participants with prediabetes at randomization)

Additional details, including analysis methods for key secondary endpoints and the strategy for controlling overall type 1 error rate at a 2-sided alpha of 0.05 of primary and key secondary endpoint evaluation, will be provided in the SAP.

9.4.4. Safety Analyses

Unless specified otherwise, safety assessments will be guided by an estimand comparing safety of tirzepatide doses with placebo irrespective of adherence to study drug. Thus, safety analysis will be conducted using SS.

9.4.4.1. Study Drug Exposure

Exposure to each study treatment will be calculated for each participant and summarized by treatment group.

9.4.4.2. Adverse Events

Adverse events will be coded from the actual term using the Medical Dictionary for Regulatory Activities (MedDRA) and reported with preferred terms and system organ class. Counts and percentages of participants experiencing events will be reported for each treatment group, and Fisher's exact test will be used to compare the treatment groups.

The percentage of participants experiencing TEAE, SAE, discontinuation due to AE will be summarized by treatment group.

9.4.4.3. Adverse Event of Special Interest

This section includes areas of interest whether due to observed safety findings, potential findings based on drug class, and agreed upon consultation with regulatory agencies for the reasons previously mentioned.

The following AEs are AEs of special interest (AESI) for this study:

- severe hypoglycemia
- major adverse cardiovascular events (adjudicated); includes, but not limited to cardiovascular death, nonfatal MI, nonfatal stroke, and hospitalization for heart failure
- treatment-emergent supraventricular arrhythmias and cardiac conduction disorders
- hepatobiliary disorders; includes biliary colic, cholecystitis, and other gallbladder disease
- severe GI events
- acute renal events
- MDD/suicidal behavior and ideation
- pancreatitis (adjudicated)
- c-cell hyperplasia and thyroid malignancies
- allergic/hypersensitivity reactions; includes injection site reactions and ADA formation.

Summaries and analyses for incidence of AESIs will be provided by treatment. The details of analysis of AESI will be provided in the SAP.

9.4.4.4. Other Adverse Event Assessments

9.4.4.4.1. Gastrointestinal Events

Summaries and analyses for incidence and severity of nausea, vomiting, and diarrhea will be provided by each treatment.

9.4.4.4.2. Events Related to Potential Abuse Liability

To identify AE terms suggestive of potential abuse liability, narrow terms from SMQ of Drug abuse and dependence (20000101) will be used.

Summaries and analyses for incidence of potential abuse liability terms will be provided by treatment. The details will be provided in the SAP.

9.4.4.4.3. Depression, Suicidal Ideation, and Behavior

In addition to the summary of TEAEs, suicidal ideation and behavior will be assessed by C-SSRS, and depression-related symptoms will be assessed using PHQ-9.

The analysis details will be provided in the SAP.

9.4.4.4.4. Central Laboratory Measures, Vital Signs, and Electrocardiograms

Actual values and change from randomization to postrandomization values of central laboratory measures, vital signs, and selected ECG parameters will be summarized at each scheduled visit. Change from randomization to postrandomization value will be summarized for participants who have both a randomization and at least 1 postrandomization result.

The percentages of participants with treatment-emergent (TE) abnormal, high, or low measures (including laboratory, vital, and ECG parameters) will be summarized and compared between treatment groups using Fisher's exact test.

The analysis details will be provided in the SAP.

9.4.5. Pharmacokinetic/Pharmacodynamic Analyses

Tirzepatide concentration data will be analyzed using a population PK approach using nonlinear mixed-effects modeling techniques implemented on the NONMEM® software. The relationships between tirzepatide dose and/or concentration and selected efficacy, tolerability, and safety endpoints will be characterized. Additionally, the impact of intrinsic and extrinsic participant factors such as age, weight, gender, and renal function on tirzepatide PK and/or PD parameters may be examined. If ADA titers are detected from immunogenicity testing, then the impact of immunogenicity titers on tirzepatide PK or any relevant PD parameters may also be examined.

9.4.6. Evaluation of Immunogenicity

The frequency and percentage of participants with preexisting ADA and with TE ADA+ to tirzepatide will be tabulated. Treatment-emergent ADAs are defined as those with a titer 2-fold (1 dilution) greater than the minimum required dilution (1:10) of the ADA assay if no ADAs were detected at baseline (treatment-induced ADA) or those with a 4-fold (2 dilutions) increase in titer compared with baseline if ADAs were detected at baseline (treatment-boosted ADA). Details of the analyses will be provided in the SAP.

9.4.7. Other Analyses

9.4.7.1. Subgroup Analyses for Primary Outcome

Details of the subgroup analyses will be shown in the SAP.

The following subgroup variables will be considered (but not limited to):

- age (<65 years and ≥65 years)
- sex (female and male)
- baseline BMI (<30, ≥30 and <35, ≥35 and <40, ≥40 kg/m^2)

- race
- ethnicity
- glycemic status at randomization (normoglycemia vs prediabetes)

The outcome measures for the subgroup analyses will include:

- percent change in body weight from randomization at 72 weeks
- percentage of participants achieving at least 5% body weight reduction at 72 weeks

9.5. Interim Analyses

Based on the projected enrollment, 3 interim analyses are planned. Changes in the number of interim analyses and the timing of conducting the interim analyses due to the change of enrollment rate will not require an amendment.

The first unblinded safety interim analysis will occur approximately 6 months after approximately 400 participants are randomized in the study to monitor the safety of the participants in the study.

The second interim analysis is the 36-week interim analysis to monitor the safety of the participants in the study with a special focus to assess the effect of 5-, 10-, and 15-mg doses of tirzepatide compared to placebo on HR. The timing of the 36-week interim analysis will be triggered by the completion of a 24-hour ambulatory monitoring sub-study; specifically, after approximately 576 randomized participants in GPHK who agree to perform ambulatory monitoring have either completed 36 weeks of treatment or discontinued earlier. An independent data monitoring committee (DMC) will have the responsibility to review unblinded interim analysis results. A statistical analysis center independent from the Sponsor will perform the data analysis for the DMC.

The third unblinded safety interim analysis will occur approximately 6 months after the 36-week interim analysis to monitor the safety of the participants in the study.

Following the third interim analysis specified above, the primary database lock will occur after all randomized participants complete the 72-week treatment period or discontinue the study early. The primary objective of the study will be assessed following the primary database lock.

The final database lock will occur after participants classified with prediabetes have completed a 176-week treatment period and the safety follow-up visit or discontinued the study early.

A limited number of individuals will have access to the unblinded data from the interim and/or primary database lock prior to the final database lock (after all randomized participants complete the entire study). Information that may unblind the study after the interim or primary database lock will not be communicated to study sites or blinded study team prior to final database lock, unless it is deemed necessary for participant safety. Details will be specified in the blinding/unblinding plan and in the DMC charter.

10. Supporting Documentation and Operational Considerations

10.1. Appendix 1: Regulatory, Ethical, and Study Oversight Considerations

10.1.1. Regulatory and Ethical Considerations

- This study will be conducted in accordance with the protocol and with the following:
    - o Consensus ethical principles derived from international guidelines including the Declaration of Helsinki and Council for International Organizations of Medical Sciences (CIOMS) International Ethical Guidelines
    - o Applicable The International Council for Harmonization of Technical Requirements for Pharmaceuticals for Human Use (ICH) Good Clinical Practice (GCP) Guidelines
    - o Applicable laws and regulations
- The protocol, protocol amendments, ICF, investigator’s brochure, and other relevant documents (for example, advertisements) must be submitted to an IRB/IEC by the investigator and reviewed and approved by the IRB/IEC before the study is initiated.
- Any amendments to the protocol will require IRB/IEC approval before implementation of changes made to the study design, except for changes necessary to eliminate an immediate hazard to study participants.
- The investigator will be responsible for the following:
    - o Providing written summaries of the status of the study to the ERB annually or more frequently in accordance with the requirements, policies, and procedures established by the IRB/IEC
    - o Notifying the IRB/IEC of SAEs or other significant safety findings as required by ERB procedures
    - o Providing oversight of the conduct of the study at the site and adherence to requirements of 21 CFR, ICH guidelines, the IRB/IEC, European regulation 536/2014 for clinical studies (if applicable), and all other applicable local regulations

10.1.2. Informed Consent Process

- The investigator or his or her representative will explain the nature of the study, including risks and benefits, to the study participant or his/her legally authorized representative and answer all questions regarding the study.

- Study participants must be informed that their participation is voluntary. Study participants or their legally authorized representative will be required to sign a statement of informed consent that meets the requirements of 21 CFR 50, local regulations, ICH guidelines, Health Insurance Portability and Accountability Act (HIPAA) requirements, where applicable, and the IRB/IEC or study center.
- The medical record must include a statement that written informed consent was obtained before the study participant was entered into the trial and the date the written consent was obtained. The authorized person obtaining the informed consent must also sign the ICF.
- Study participants must be reconsented to the most current version of the ICF(s) during their participation in the trial.
- A copy of the ICF(s) must be provided to the study participant or the study participant's legally authorized representative.

10.1.3. Data Protection

- Study participants will be assigned a unique identifier by the investigator. Any study participant records, datasets, or tissue samples that are transferred to the Sponsor will contain the identifier only; study participant names or any information which would make the study participant identifiable will not be transferred.
- The study participant must be informed that his/her personal trial-related data will be used by the Sponsor in accordance with local data protection law. The level of disclosure must also be explained to the study participant.
- The study participant must be informed that his/her medical records may be examined by Clinical Quality Assurance auditors or other authorized personnel appointed by the Sponsor, by appropriate IRB/IEC members, and by inspectors from regulatory authorities.

10.1.4. Committees Structure

Prospective adjudication of incident diabetes events as well as major adverse cardiovascular events and pancreatic AEs will be performed by the independent adjudication committees for this study.

More details on adjudication of incident diabetes events can be found in Section 4.1.2.3.

Section 10.3 Appendix 3 outlines additional information on pancreatic and cardiovascular adjudication committees.

An independent DMC for the interim analysis (Section 9.5) will include members with no conflict of financial interest.

10.1.5. Dissemination of Clinical Study Data

Required clinical trial registries (for example, ClinicalTrials.gov) will be updated with the results from registered clinical trials regardless of the research outcome in accordance with local laws and regulations.

All CSRs, amendments, and addenda will be submitted to external regulatory authorities, external partners (as applicable), and sites.

The publication policy for Study I8F-MC-GPHK (SURMOUNT-1) is outlined in Section 10.1.10 and further described in the Clinical Trial Agreement.

10.1.6. Data Quality Assurance

To ensure accurate, complete, and reliable data, Lilly or its representatives will

- provide instructional material to the study sites, as appropriate
- provide sponsor start-up training to instruct the investigators and study coordinators. This training will provide instruction on the protocol, the completion of the eCRFs, and study procedures
- make periodic visits to the study site
- be available for consultation and stay in contact with the study site personnel by mail, telephone, and/or fax, and
- review and verify data reported to detect potential errors.

In addition, Lilly or its representatives will periodically check a sample of the participant data recorded against source documents at the study site. The study may be audited by Lilly or its representatives and/or regulatory agencies at any time. Investigators will be given notice before an audit occurs.

The investigator will keep records of all original source data. This might include laboratory tests, medical records, and clinical notes. If requested, the investigator will provide the Sponsor, applicable regulatory agencies, and applicable ERBs with direct access to original source documents.

10.1.7. Data Capture System

The investigator is responsible for ensuring the accuracy, completeness, legibility, and timeliness of the data reported to the Sponsor.

An electronic data capture (EDC) system will be used in this study for the collection of CRF data. The investigator maintains a separate source for the data entered by the investigator or designee into the sponsor-provided EDC system. The investigator is responsible for the identification of any data to be considered source and for the confirmation that data reported are accurate and complete by signing the eCRF.

Additionally, clinical outcome assessment data (scales, self-reported diary data) will be collected by the subject/investigator site personnel via a paper source document and will be transcribed by the investigator site personnel into the EDC system.

Data collected via the sponsor-provided data capture systems will be stored at third parties. The investigator will have continuous access to the data during the study and until decommissioning of the data capture systems. Prior to decommissioning, the investigator will receive an archival copy of pertinent data for retention.

Data managed by a central vendor, such as laboratory test data, will be stored electronically in the central vendor's database system and electronic transfers will be provided to the investigator for review and retention. Data will subsequently be transferred from the central vendor to the Lilly data warehouse.

Data from complaint forms submitted to Lilly will be encoded and stored in the global product complaint management system.

10.1.8. Source Documents

- Source documents provide evidence for the existence of the study participant and substantiate the integrity of the data collected. Source documents are filed at the investigator's site.
- Data reported on the CRF or entered in the eCRF that are transcribed from source documents must be consistent with the source documents or the discrepancies must be explained. The investigator may need to request previous medical records or transfer records, depending on the trial. Also, current medical records must be available.

10.1.9. Study and Site Closure

10.1.9.1. Discontinuation of Study

The study will be discontinued if Lilly or its designee judges it necessary for medical, safety, regulatory, or other reasons consistent with applicable laws, regulations, and GCP.

10.1.9.2. Discontinuation of Study Sites

Study site participation may be discontinued if Lilly or its designee, the investigator, or the ERB of the study site judges it necessary for medical, safety, regulatory, or other reasons consistent with applicable laws, regulations, and GCP.

10.1.10. Publication Policy

- The Sponsor will comply with the requirements for publication of study results.
- Authorship will be determined by mutual agreement and in line with International Committee of Medical Journal Editors authorship requirements.
- The publication policy for Study I8F-MC-GPHK (SURMOUNT-1) is described in the Clinical Trial Agreement.

10.2. Appendix 2: Clinical Laboratory Tests

- The tests detailed below will be performed by the central laboratory unless otherwise stated.
- Local laboratory results are only required in the event that the central laboratory results are not available in time for response evaluation. If a local sample is required, it is important that the sample for central analysis is obtained at the same time. Additionally, if the local laboratory results are used to make either a study intervention decision or response evaluation, the results must be entered into the eCRF.
- Protocol-specific requirements for inclusion or exclusion of participants are detailed in Section 5 of the protocol.
- Additional tests may be performed at any time during the study as determined necessary by the investigator or required by local regulations.
- Refer to Section 5.1 (Inclusion Criteria) for screening pregnancy criteria.

Investigators must document their review of each laboratory safety report.

Laboratory/analyte results that could unblind the study will not be reported to investigative sites or other blinded personnel until the study has been unblinded.

Clinical Laboratory Tests

Hematology[a]
Hemoglobin
Hematocrit
Erythrocyte count (RBC)

Mean cell volume
Mean cell hemoglobin concentration
Leukocytes (WBC)
Neutrophils, segmented
Lymphocytes
Monocytes
Eosinophils
Basophils
Platelets

Urine Chemistries[a]
Albumin
Creatinine

Nonpharmacogenetic Stored Samples
Serum
EDTA plasma
P800 plasma

Pharmacokinetics samples[a,e,f,g]

Pharmacogenetics Sample
Whole blood (EDTA)

Oral Glucose Tolerance Test (glucose, insulin[e], c-peptide[e])
Cystatin-C[a]
HbA1c[a]

Endocrine[a]
Calcitonin
Thyroid-stimulating hormone (TSH)

Insulin[e]

C-peptide[e]

Clinical Chemistry[a]

Sodium
Potassium
Bicarbonate
Total bilirubin
Direct bilirubin
Alkaline phosphatase
Alanine aminotransferase (ALT)
Aspartate aminotransferase (AST)
Blood urea nitrogen (BUN)
Creatinine
Uric acid
Calcium
Glucose, fasting
Albumin
Total Cholesterol
Triglycerides
Creatine kinase (CK)

Hormones (females)
Pregnancy Test [a], serum and/or urine[b]
Follicle-stimulating hormone (FSH)[d]
Calculations
eGFR (calculated by CKD-EPI equation)[a,c]
UACR (urine albumin mg/dL/creatinine g/dL=UACR mg/g)

Pancreas (exocrine) [a]
Pancreatic amylase
Lipase

Immunogenicity[a,e,g]
Anti-tirzepatide antibodies
Anti-tirzepatide antibody neutralization

Lipid Panel[a]
LDL-C
HDL-C
VLDL-C

Free Fatty Acids[e]

Abbreviations: ADA = antidrug antibody; ALT = alanine aminotransferase; ALP = alkaline phosphatase; AST = aspartate aminotransferase; BUN = blood urea nitrogen; CK = creatinine kinase, CKD-EPI = Chronic Kidney Disease-Epidemiology; eGFR = estimated glomerular filtration rate; EDTA = ethylenediaminetetraacetic acid; FSH = follicle-stimulating hormone; HbA1c = hemoglobin A1c; HDL = high-density lipoprotein; IWRS = interactive web-response system; LDL = low-density lipoprotein; PK = pharmacokinetics; RBC = red blood cells; TSH = thyroid-stimulating hormone; UACR = urine albumin/creatinine ration; VLDL = very low-density lipoprotein; WBC = white blood cells.

[a] All tests will be performed by a Lilly-designated central laboratory, unless otherwise noted.

[b] A serum pregnancy test will be performed at Visit 1 for women of childbearing potential only.

A urine pregnancy test must be performed at Visit 3 with the result available prior to randomization and first injection of study drug(s) for women of childbearing potential only. Additional pregnancy tests (beyond those required per the Schedule of Activities [Section 1.3]) should be performed at any time during the trial if a menstrual period is missed, there is clinical suspicion of pregnancy, or as required by local law or regulation.

[c] Estimated glomerular filtration rate will be calculated by the central laboratory at all visits and included in lab result reports.

[d] Follicle-stimulating hormone test performed at Visit 1 for postmenopausal women at least 40 years of age with an intact uterus, not on hormone therapy, and who have had spontaneous amenorrhea for more than 1 year without an alternative medical cause.

[e] Results will not be provided to the investigative sites.

[f] PK samples will be collected at these visits at time windows of 1 to 24 hours, 24 to 96 hours, or 120 to 168 hours postdose, as assigned by IWRS. Dependent on the time-windows to which a participant gets assigned, they may be required to come to site for PK-specific visits.

[g] In the event of systemic drug hypersensitivity reactions (immediate or nonimmediate), additional blood samples will be collected including ADA, PK, and exploratory biomarker samples. PK samples for immunogenicity must be taken prior to drug administration.

Selected tests may be obtained in the event of anaphylaxis or systemic allergic/hypersensitivity reactions.

**Hypersensitivity Tests**[a]	
Anti-LY antibodies (immunogenicity)	Tryptase[b]
LY concentration (PK)	N-methylhistamine[b]
	Drug Specific IgE[b]
	Basophil Activation Test[b]
	Complements
	Cytokine Panel

Abbreviations: LY = LY3298176; IgE = immunoglobulin E; PK = pharmacokinetics.

[a] Assayed by Lilly-designated laboratory.

[b] Will be performed if a validated assay is available.

10.3. Appendix 3: Adverse Events: Definitions and Procedures for Recording, Evaluating, Follow-Up, and Reporting

Please refer to Section 8.3 Adverse Events and Serious Adverse Events.

10.3.1. Special Safety Topics

10.3.1.1. Hypoglycemia

Upon ICF signing, all participants will be educated about signs and symptoms of hypoglycemia, how to treat hypoglycemia, and how to collect appropriate information for each episode of hypoglycemia.

Hypoglycemia may be identified by spontaneous reporting of symptoms from participants (whether confirmed or unconfirmed by simultaneous glucose values) or by blood glucose (BG) samples collected during study visits.

All participants who develop diabetes during the study will be provided with glucometers. Participants without diabetes may, at the investigator's discretion, be given glucometers to assist in the evaluation of reported symptoms consistent with hypoglycemia. Participants receiving glucometers will be provided a diary to record relevant information (for example, glucose values, symptoms).

Hypoglycemic episodes will be recorded on a specific eCRF and should not be recorded as AEs unless the event meets serious criteria. If a hypoglycemic event meets severe criteria (see definition below), it should be recorded as serious on the AE and SAE eCRFs, and reported to Lilly as an SAE.

Investigators should use the following definitions and criteria when diagnosing and categorizing an episode considered to be related to hypoglycemia (the plasma Glucose values in this section refer to values determined by a laboratory or International Federation of Clinical Chemistry and Laboratory Medicine blood-equivalent glucose meters and strips) in accordance with the 2019 American Diabetes Association position statement on glycemic targets (American Diabetes Association 2019a):

Glucose Alert Value (Level 1):

- **Documented symptomatic hypoglycemia** is defined as any time a patient feels that he or she is experiencing symptoms and/or signs associated with hypoglycemia and has a BG level of <70 mg/dL (≤3.9 mmol/L).
- **Documented asymptomatic hypoglycemia** is defined as any event not accompanied by typical symptoms of hypoglycemia, but with a measured BG <70 mg/dL (≤3.9 mmol/L).
- **Documented unspecified hypoglycemia** is defined as any event with no information about symptoms of hypoglycemia available, but with a measured BG <70 mg/dL (≤3.9 mmol/L).

Clinically Significant Hypoglycemia (Level 2):

- **Documented symptomatic hypoglycemia** is defined as any time a participant feels that he or she is experiencing symptoms and/or signs associated with hypoglycemia and has a BG level of <54 mg/dL (<3.0 mmol/L).
- **Documented asymptomatic hypoglycemia** is defined as any event not accompanied by typical symptoms of hypoglycemia but with a measured BG <54 mg/dL (<3.0 mmol/L).
- **Documented unspecified hypoglycemia** is defined as any event with no information about symptoms of hypoglycemia available but with a measured BG <54 mg/dL (<3.0 mmol/L).

Severe hypoglycemia (Level 3):

- **Severe hypoglycemia** is defined as an episode with severe cognitive impairment requiring the assistance of another person to actively administer carbohydrate, glucagon, or other resuscitative actions. These episodes may be associated with sufficient neuroglycopenia to induce seizure or coma. Blood glucose measurements may not be available during such an event, but neurological recovery attributable to the restoration of BG to normal is considered sufficient evidence that the event was induced by a low BG concentration.

To avoid duplicate reporting, all consecutive BG values ≤70 mg/dL (3.9 mmol/L) occurring within a 1-hour period may be considered to be a single hypoglycemic event (Weinberg et al. 2010; Danne et al. 2013).

10.3.1.2. Pancreatitis

Acute pancreatitis is defined as an AE of interest in all trials with tirzepatide, including this trial. Acute pancreatitis is an acute inflammatory process of the pancreas that may also involve peripancreatic tissues and/or remote organ systems (Banks and Freeman 2006). The diagnosis of acute pancreatitis requires 2 of the following 3 features:

- abdominal pain, characteristic of acute pancreatitis (generally located in the epigastrium and radiates to the back in approximately half the cases) (Banks and Freeman 2006; Koizumi et al. 2006); the pain is often associated with nausea and vomiting);
- serum amylase (total and/or pancreatic) and/or lipase ≥3X ULN
- characteristic findings of acute pancreatitis on computed tomography (CT) scan or magnetic resonance imaging (MRI).

If acute pancreatitis is suspected, appropriate laboratory tests (including levels of pancreatic amylase and lipase) should be obtained via the central laboratory (and locally, if needed). Imaging studies, such as abdominal CT scan with or without contrast, MRI, or gallbladder ultrasound, should be performed. Abdominal ultrasound may be used as an alternative method only if CT and MRI cannot be performed. If laboratory values and/or abdominal imaging support the diagnosis of acute pancreatitis, the participant must discontinue therapy with

tirzepatide, but will continue in the study. A review of the participant's concomitant medications should be conducted to assess any potential causal relationship with pancreatitis.

Each AE of pancreatitis must be reported. If typical signs and/or symptoms of pancreatitis are present and confirmed by laboratory values (lipase or amylase [total and/or pancreatic]) and imaging studies, the event must be reported as an SAE. For a potential case that does not meet all of these criteria, it is up to the investigator to determine the seriousness of the case (AE or SAE) and the relatedness of the event to study drug(s).

Each participant will have measurements of p-amylase and lipase (assessed at the central laboratory) as shown on the Schedule of Activities (Section 1.3) to assess the effects of the investigational doses of tirzepatide on pancreatic enzyme levels. Serial measurements of pancreatic enzymes have limited clinical value for predicting episodes of acute pancreatitis in asymptomatic participants (Nauck et al. 2017; Steinberg et al. 2017a, 2017b). Thus, further diagnostic follow-up of cases of asymptomatic pancreatic hyperenzymemia (lipase and/or pancreatic amylase ≥3X ULN) is not mandated but may be performed based on the investigator's clinical judgment and assessment of the participant's overall clinical condition. Only cases of pancreatic hyperenzymemia that undergo additional diagnostic follow-up and/or are accompanied by symptoms suggestive of pancreatitis will be submitted for adjudication.

All suspected cases of acute or chronic pancreatitis will be adjudicated by an independent clinical endpoint committee. In addition, AEs of severe or serious abdominal pain of unknown etiology will also be submitted to the adjudication committee to assess for possible pancreatitis or other pancreatic disease. Relevant data from participants with acute or chronic pancreatitis and those with severe or serious abdominal pain will be entered into a specifically designed eCRF page. The adjudication committee representative will enter the results of adjudication in a corresponding eCRF page.

10.3.1.3. Thyroid Malignancies and C-Cell Hyperplasia

Individuals with personal or family history of MTC and/or MEN-2 will be excluded from the study. Participants who are diagnosed with MTC and/or MEN-2 during the study will have study drug stopped and should continue follow-up with an endocrinologist.

The assessment of thyroid safety during the trial will include reporting of any case of thyroid malignancy (including MTC and papillary carcinoma) and measurements of calcitonin. This data will be captured in specific eCRFs. The purpose of calcitonin measurements is to assess the potential of tirzepatide to affect thyroid C-cell function, which may indicate development of C-cell hyperplasia and neoplasms.

10.3.1.4. Calcitonin Measurements

If an increased calcitonin value (see definitions below) is observed in a participant who has been administered a medication that is known to increase serum calcitonin, then this medication should be stopped and calcitonin levels should be measured after an appropriate washout period.

For participants who require additional endocrine assessment because of increased calcitonin concentration as defined in this section, data from the follow-up assessment will be collected in the specific section of the eCRF.

Calcitonin Measurements in Participants with eGFR ≥60 mL/min/1.73m²

A significant increase in calcitonin for participants with eGFR ≥60 mL/min is defined below. If a participant's laboratory results meet these criteria, these clinically significant laboratory results should be recorded as an AE.

- *calcitonin value ≥20 ng/L and <35 ng/L AND ≥50% increase from the screening value.* These participants will be asked to repeat the measurement within 1 month. If this repeat value is increasing (≥10% increase), study drug should be stopped, and the participant encouraged to undergo additional endocrine assessment and longer-term follow-up by an endocrinologist to exclude any serious adverse effect on the thyroid.
- *calcitonin value ≥35 ng/L AND ≥50% over the screening value*. In these participants, study drug should be stopped, and the participant recommended to immediately undergo additional endocrine assessments and longer-term follow-up by an endocrinologist.

Calcitonin Measurement in Participants with eGFR <60 mL/min/1.73m^2

A significant increase in calcitonin for participants with eGFR <60 mL/min/1.73m^2 is defined as a *calcitonin value ≥35 ng/L AND ≥50% over the screening value.* If a participant's labs meet these criteria, these clinically significant labs should be recorded as an AE.

In these participants, study drug should be discontinued (after first confirming the value) and the participant recommended to immediately undergo additional endocrine assessments and longer-term follow-up by an endocrinologist to exclude any serious adverse effect on the thyroid.

10.3.1.5. Major Adverse Cardiovascular Events

Deaths and nonfatal cardiovascular AEs will be adjudicated by a committee of physicians external to Lilly with cardiology expertise. This committee will be blinded to treatment assignment. The nonfatal cardiovascular AEs to be adjudicated include

- myocardial infarction
- hospitalization for unstable angina
- hospitalization for heart failure
- coronary interventions (such as coronary artery bypass graft or percutaneous coronary intervention), and
- cerebrovascular events, including cerebrovascular accident (stroke) and transient ischemic attack.

10.3.1.6. Supraventricular Arrhythmias and Cardiac Conduction Disorders

Treatment-emergent cardiac conduction disorders will be further evaluated. Participants who develop any event from these groups of disorders should undergo an ECG, which should be submitted to the central reading center. The specific diagnosis will be recorded as an AE. Events that meet criteria for serious conditions as described in Section 8.3 must be reported as SAEs.

10.3.1.7. Hypersensitivity Events

All allergic or hypersensitivity reactions will be reported by the investigator as either AEs, or if any serious criterion is met, as SAEs.

In the event of suspected drug hypersensitivity reactions (immediate or nonimmediate) in subjects who experience moderate-to-severe injection reactions as assessed by the investigator, unscheduled blood samples will be collected for PK and ADA analyses at the following time points:

- as close as possible to the onset of the event
- at the resolution of the event
- 30 (±3) days following the event.

Additionally, unscheduled serum samples for immune safety laboratory testing (including, but not limited to β tryptase, total IgE, complement, and cytokine panel testing) should also be collected at approximately 60 to 120 minutes and 4 to 6 weeks after the onset of the event in these participants.

Additional data, such as type of reaction and treatment received, will be collected on any AEs or SAEs that the investigator deems related to study drug(s) via the eCRF created for this purpose. Study drug(s) should be temporarily interrupted in any individual suspected of having a severe or serious allergic reaction to study drug(s). Study drug(s) may be restarted when/if it is safe to do so, in the opinion of the investigator.

10.3.1.8. Injection Site Reactions

Injection site reactions will be collected on the eCRF separate from the hypersensitivity reaction eCRF. At the time of AE occurrence, samples will be collected for measurement of tirzepatide ADA and tirzepatide concentration.

10.3.1.9. Antidrug Antibodies

The occurrence of ADA formation will be assessed as outlined in Section 8.7.

10.3.1.10. Hepatobiliary Disorders

All events of TE biliary colic, cholecystitis, or other suspected events related to gallbladder disease should be evaluated and additional diagnostic tests performed, as needed. In cases of elevated liver markers, hepatic monitoring should be initiated as outlined in Section 7.

10.3.1.11. Severe Gastrointestinal Adverse Events

Tirzepatide may cause severe GI AEs, such as nausea, vomiting, and diarrhea. Information about severe GI AEs as well as antiemetic/antidiarrheal use will be collected in the eCRF/AE form. For detailed information concerning the management of GI AEs, please refer to Section 6.6.2.

10.3.1.12. Acute Renal Events

Renal safety will be assessed based on repeated renal functional assessment as well as assessment of AEs suggestive of acute or worsening of chronic renal failure. Gastrointestinal

AEs have been reported with tirzepatide, including nausea, diarrhea, and vomiting. This is consistent with other GLP-1R agonists (Aroda and Ratner 2011). The events may lead to dehydration, which could cause a deterioration in renal function, including acute renal failure. Participants should be advised to notify investigators in case of severe nausea, frequent vomiting, or symptoms of dehydration.

10.3.1.13. Depression, Suicidal Ideation, or Behavior Monitoring

Participants will be monitored for depression and suicidal ideation or behavior through AE collection and by using the C-SSRS and the PHQ-9. Participants will be referred to an MHP if, in the opinion of the investigator, it is necessary for the safety of the participant, or if the participant had any of the following:

- a PHQ-9 score ≥15
- C-SSRS responses of
  - A “yes” answer to either Question 4 (Active Suicidal Ideation with Some Intent to Act, Without Specific Plan)
  - **or**
  - A “yes” answer to Question 5 (Active Suicidal Ideation with Specific Plan and Intent) on the “Suicidal Ideation” portion of the C-SSRS
  - **or**
  - A “yes” answer to any of the suicide-related behaviors (actual attempt, interrupted attempt, aborted attempt, preparatory act, or behavior) on the “Suicidal Behavior” portion of the C-SSRS.

10.4. Appendix 4: Contraceptive Guidance and Collection of Pregnancy Information

Definitions:

Woman of Childbearing Potential

A woman is considered fertile following menarche and until becoming postmenopausal unless permanently sterile (see below).

If fertility is unclear (for example; amenorrhea in adolescents or athletes) and a menstrual cycle cannot be confirmed before first dose of study intervention, additional evaluation should be considered.

Women in the following categories are not considered women of childbearing potential

Article I. Premenarchal

Article II. Premenopausal female with 1 of the following:

- Documented hysterectomy
- Documented bilateral salpingectomy
- Documented bilateral oophorectomy

For individuals with permanent infertility due to an alternate medical cause other than the above, (for example; Mullerian agenesis, androgen insensitivity), investigator discretion should be applied to determining study entry.

Note: Documentation can come from the site personnel's review of the participant's medical records, medical examination, or medical history interview.

Article III. Postmenopausal female

- A postmenopausal state is defined as either
    - A woman at least 40 years of age with an intact uterus, not on hormone therapy, who has cessation of menses for at least 1 year without an alternative medical cause, AND a follicle-stimulating hormone (FSH) ≥40 mIU/mL; or
    - A woman 55 or older not on hormone therapy, who has had at least 12 months of spontaneous amenorrhea; or
    - A woman at least 55 years of age with a diagnosis of menopause prior to starting hormone replacement therapy.
- Females on HRT and whose menopausal status is in doubt will be required to use one of the nonestrogen hormonal highly effective contraception methods if they wish to continue their HRT during the study. Otherwise, they must discontinue HRT to allow confirmation of postmenopausal status before study enrollment.

Contraception Guidance:

Contraceptive use by men and women should be consistent with local regulations regarding the methods of contraception for those participating in clinical studies.

Two forms of effective contraception, where at least 1 form is highly effective, will be used. Effective contraception may be used as the second therapy. Barrier protection methods without concomitant use of a spermicide are not a reliable or acceptable method. The use of male and female condoms as a double barrier method is not considered acceptable due to the high failure rate when these methods are combined.

Highly Effective Methods of Contraception:

- Combined oral contraceptive pill and mini pill
- NuvaRing
- Implantable contraceptives
- Injectable contraceptives (such as Depo-Provera®)
- Intrauterine device (such as Mirena® and ParaGard®)
- Contraceptive patch – ONLY women <198 pounds or 90 kg
- Total abstinence (if this is their preferred and usual lifestyle) or in a same-sex relationship with no sexual relationship with males (as part of their preferred and usual lifestyle), and agrees to maintain this status throughout trial follow-up

  Note: periodic abstinence (for example, calendar, ovulation, symptothermal, and postovulation methods), declaration of abstinence just for the duration of a trial, and withdrawal are not acceptable methods of contraception

- Vasectomy – for men in clinical studies

  Note: Implantable contraceptives and injectable contraceptives (such as Depo Provera) are only permitted if started more than 18 months prior to screening. Participants should not start these methods of contraception after being enrolled in the study.

Effective Methods of Contraception (must use combination of 2 methods):

- Male condom with spermicide
- Female condom with spermicide
- Diaphragm with spermicide
- Cervical sponge
- Cervical cap with spermicide

Men, regardless of their fertility status, with nonpregnant women of childbearing potential partners must agree to either remain abstinent (if this is their preferred and usual lifestyle) or use condoms plus 1 additional highly effective (less than 1% failure rate) method of contraception (such as combination oral contraceptives, implanted contraceptives, or intrauterine device) or effective method of contraception (such as diaphragms with spermicide or cervical sponge) for the duration of the study and for 5 half-lives of study drug plus 90 days, which is approximately 4 months after the last injection. Periodic abstinence (for example, calendar, ovulation, symptothermal, and postovulation methods), declaration of abstinence just for the duration of a trial, and withdrawal are not acceptable methods of contraception.

Men with pregnant partners should use condoms during intercourse for the duration of the study and until the end of estimated relevant potential exposure in women of childbearing potential.

Men who are abstinent (if this is complete abstinence, as their preferred and usual lifestyle) or in a same-sex relationship (as part of their preferred and usual lifestyle) must agree to either remain abstinent or stay in a same-sex relationship without sexual relationships with females (usual lifestyle). In these situations, men are not required to use contraception.

Men should refrain from sperm donation for the duration of the study and for 5 half-lives of study drug plus 90 days after the last dose of study drug, corresponding to 4 months after the last injection.

Collection of Pregnancy Information

Male participants with partners who become pregnant

- The investigator will attempt to collect pregnancy information on any male participant's female partner who becomes pregnant while the male participant is in this study.
- After obtaining the necessary signed informed consent from the pregnant female partner directly, the investigator will record pregnancy information on the appropriate form and submit it to the Sponsor within 24 hours of learning of the partner's pregnancy. The female partner will also be followed to determine the outcome of the pregnancy. Information on the status of the mother and child will be forwarded to the Sponsor. Generally, the follow-up will be no longer than 6 to 8 weeks following the estimated delivery date. Any termination of the pregnancy will be reported regardless of fetal status (presence or absence of anomalies) or indication for the procedure.

Female Participants who become pregnant

- The investigator will collect pregnancy information on any female participant who becomes pregnant while participating in this study. Information will be recorded on the appropriate form and submitted to the Sponsor within 24 hours of learning of a participant's pregnancy.
- The participant will be followed to determine the outcome of the pregnancy. The investigator will collect follow-up information on the participant and the neonate and the information will be forwarded to the Sponsor. Generally, follow-up will not be required for longer than 6 to 8 weeks beyond the estimated delivery date. Any termination of pregnancy will be reported, regardless of fetal status (presence or absence of anomalies) or indication for the procedure.
- While pregnancy itself is not considered to be an AE or SAE, any pregnancy complication or elective termination of a pregnancy will be reported as an AE or SAE. A spontaneous abortion is always considered to be an SAE and will be reported as such. Any poststudy pregnancy-related SAE considered reasonably related to the study intervention by the investigator will be reported to the Sponsor as described in Section 8.3.2. While the investigator is not obligated to actively seek this information in former study participants, he or she may learn of an SAE through spontaneous reporting.

- Any female participant who becomes pregnant while participating in the study will discontinue study intervention and be withdrawn from the study.

10.5. Appendix 5: Liver Safety: Suggested Actions and Follow-Up Assessments

Hepatic Monitoring Tests

**Hepatic Hematology**[a]	**Haptoglobin**[a]
Hemoglobin	
Hematocrit	**Hepatic Coagulation**[a]
RBC	Prothrombin Time
WBC	Prothrombin Time, INR
Neutrophils, segmented	
Lymphocytes	**Hepatic Serologies**[a,b]
Monocytes	Hepatitis A antibody, total
Eosinophils	Hepatitis A antibody, IgM
Basophils	Hepatitis B surface antigen
Platelets	Hepatitis B surface antibody
	Hepatitis B Core antibody
**Hepatic Chemistry**[a]	Hepatitis C antibody
Total bilirubin	Hepatitis E antibody, IgG
Direct bilirubin	Hepatitis E antibody, IgM
Alkaline phosphatase	
ALT	**Antinuclear antibody**[a]
AST	
GGT	**Alkaline Phosphatase Isoenzymes**[a]
CPK	
	**Anti-smooth muscle antibody (or anti-actin antibody)**[a]

Abbreviations: ALT = alanine aminotransferase; AST = aspartate aminotransferase; CPK = creatinine phosphokinase; GGT = gamma-glutamyl transferase; Ig = immunoglobulin; INR = international normalized ratio; RBC = red blood cells; WBC = white blood cells.

a Assayed by Lilly-designated or local laboratory.

b Reflex/confirmation dependent on regulatory requirements and/or testing availability.

10.6. Appendix 6: Medical Device Incidents: Definition and Procedures for Recording, Evaluating, Follow-Up, and Reporting

Definitions of a Medical Device Incident

The detection and documentation procedures described in this protocol apply to all sponsor medical devices provided for use in the study.

**Medical Device Incident Definition**
• A medical device incident is any malfunction or deterioration in the characteristics and/or performance of a device as well as any inadequacy in the labeling or the instructions for use *which, directly or indirectly, might lead to or might have led to a serious injury, death of a participant/user/other person, or to a serious deterioration in his/her state of health.*<br>• Not all incidents lead to death or serious deterioration in health. The nonoccurrence of such a result might have been due to other fortunate circumstances or to the intervention of health care personnel.

It is sufficient that:

- An **incident** associated with a device happened.
- AND
- The **incident** was such that, if it occurred again, might lead to death or a serious deterioration in health.

A serious deterioration in state of health can include any of the following:

- Life-threatening illness
- Permanent impairment of body function or permanent damage to body structure
- Condition necessitating medical or surgical intervention to prevent one of the above
- Fetal distress, fetal death, or any congenital abnormality or birth defects

**Examples of Incidents**
• A participant, user, caregiver, or health care professional is injured as a result of a medical device failure or its misuse.<br>• A participant's study intervention is interrupted or compromised by a medical device failure.<br>• A misdiagnosis due to a medical device failure leads to inappropriate treatment.<br>• A participant's health deteriorates due to a medical device failure.

Documenting Medical Device Incidents

Medical Device Incident Documenting
• Any medical device incident occurring during the study will be documented in the participant's medical records, in accordance with the investigator's normal clinical practice, and on the appropriate form of the eCRF.<br>• For incidents fulfilling the definition of an AE or an SAE, the appropriate AE/SAE eCRF page will be completed as described in Section 8.3.<br>• A product complaint must be submitted describing the issue or deficiency that may have led to the incident or AE.<br>• The eCRF will be completed as thoroughly as possible and signed by the investigator before transmittal to the Sponsor or designee.<br>• It is very important that the investigator provides his/her assessment of causality (relationship to the medical device provided by the Sponsor) at the time of the initial AE or SAE report and describes any corrective or remedial actions taken to prevent recurrence of the incident.

10.7. Appendix 7: Protocol GPHK Standardized Protocols for the Measurement of Height, Weight, Waist Circumference, Vital Signs, Electrocardiogram, and Oral Glucose Tolerance Test

The following information has been adapted from standardized physical measurement protocols for the World Health Organization's STEPwise approach to Surveillance (STEPS) (WHO 2008) (Available at: https://www.who.int/ncds/surveillance/steps/Section%204%20Step%202%20Physical%20Measurements.pdf) Accessed January 17, 2019.

Measuring Height

Step 1. Ask the participant to remove their footwear and any headgear (light headgear worn for religious reasons can remain, but this should be worn by the participant at every clinic visit when their height is measured).

Step 2. Ask the participant to stand on the calibrated height measuring board (stadiometer) or against a wall with their feet together and their knees straight with their heels against the backboard, the stadiometer, or the wall.

Step 3. Ask the participant to look straight ahead without tilting their head up.

Step 4. Ask the participant to breathe in and stand tall. Measure and record the participant's height in centimeters (cm) to 1 decimal place.

Measuring Weight

- Body weight measurements should be done in a consistent manner using a calibrated electronic scale capable of measuring weight in kilograms (kg) to 1 decimal place.
- All weights for a given participant should be measured using the same scale, whenever possible, at approximately the same time in the morning after evacuation of bladder contents.
- Body weight must be measured in fasting state. If the participant is not fasting, the participant should be called in for a new visit within the visit window to have the fasting body weight measured.

Step 1. Ask the participant to empty their pockets, remove their footwear, outerwear (coat, jacket, etc.), and any headgear (light headgear worn for religious reasons can remain, but this should be worn by the participant at every clinic visit when weight is measured).

Step 2. Make sure the scale is placed on a firm, flat, even surface (not on carpet, on a sloping surface, or a rough, uneven surface).

Step 3. Ask the participant to step onto the scale with 1 foot on each side of the scale.

Step 4. Ask the participant to stand still with arms by sides and then record weight in kilograms (kg) to the nearest one-tenth kg.

Measuring Waist Circumference

- Waist circumference should be measured in the horizontal plane and at the midpoint between the lower margin of the last palpable rib and the top of the iliac crest.
- Measurements should be taken at the end of a normal expiration using a nonstretchable measuring tape. The tape should lie flat against the skin without compressing the soft tissue.
- The waist circumference should be measured twice, rounded to the nearest 0.5 cm. The measuring tape should be removed between the 2 measurements. Both measurements will be recorded in the eCRF. If the difference between the 2 measurements exceeds 1 cm, this set of measurements should be discarded and the 2 measurements repeated.

Step 1. Ask the participant to wear light clothing (if available, patient gowns could also be used).

Step 2. Ask the participant to stand with their feet close together, arms at their side, body weight evenly distributed.

Step 3. Ask the participant to relax and measure the participant's waist circumference.

Vital Sign Measurements (blood pressure and heart rate)

- Vital sign measurements (blood pressure and heart rate, measured by pulse) should be taken before obtaining an ECG tracing and before collection of blood samples for laboratory testing
- The participant should sit quietly for 5 minutes before vital signs measurements are taken
- For each parameter, 3 measurements will be taken using the same arm, preferably the nondominant arm
- The recordings should be taken at least 1 minute apart. Each measurement of sitting pulse and BP needs to be recorded in the eCRF
- Blood pressure must be taken with an automated blood pressure instrument
- If blood pressure and pulse measurements are taken separately, pulse should be taken prior to blood pressure.

Note: In the event pulse measurement cannot be taken via an automated blood pressure instrument, the preferred location for measurement of pulse is the radial artery.

Electrocardiogram

- All digital ECGs will be obtained using centrally provided ECG machines and will be electronically transmitted to a designated central ECG laboratory.
- 12-lead ECGs should be obtained after the subject has rested in a supine position for at least 10 minutes.

- Electrocardiograms should be collected at least 30 minutes prior to collection of blood samples for laboratory testing, including PK samples.
- Triplicate ECGs (obtained at Week 0 and Week 36) should be obtained approximately 1 minute apart, with all 3 tracings to be obtained within approximately 5 minutes. Measurements that deviate substantially from previous readings should be repeated immediately.

2-Hour Oral Glucose Tolerance Test

- Participants should maintain adequate carbohydrate intake for 3 days prior to the scheduled 2-hour OGTT.
- In the 24 hours preceding the test, patients should refrain from drinking any alcohol or performing any extreme physical activity.
- Participants should fast for approximately 8 hours before the administration of the test and should not eat until the test is complete.
- Placement of a venous cannula, preferably in an antecubital vein, is recommended to simplify collection of multiple blood samples for glucose, insulin, and C-peptide at time 0, 30, 60, 90, and 120 minutes.

Note: Placement of a venous cannula may not be required when a 2-hour OGTT is repeated to confirm the diagnosis of diabetes (see Section 4.1.2.3), and only samples at 0 and 120 minutes for glucose measurement will be collected.

- Immediately after collection of the time 0 sample, a 75-gram glucose dose will be given orally, using a commercial product approved for this use (and in a total volume of not more than 350 mL).
- The participant should consume the glucose load within 5 minutes.
- The patient should remain minimally active for the duration of the test.

10.8. Appendix 8: Suggested Visit Structure

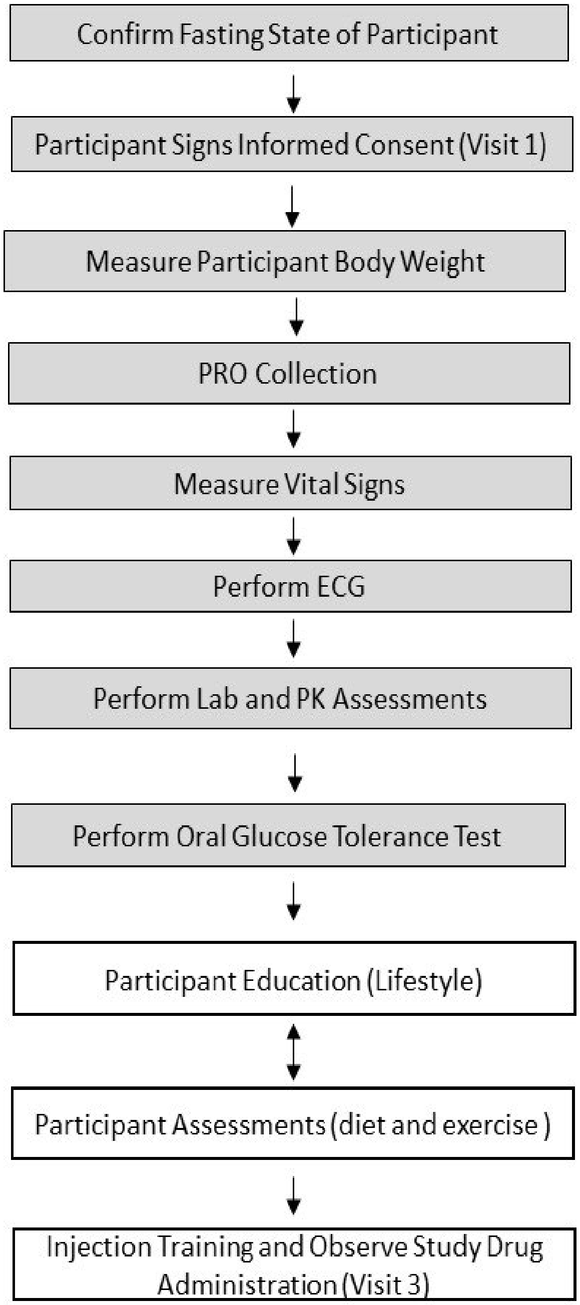

- Activities should be performed in the following order at the specified visits in the SOA
- Shaded areas must be performed in a fasted state and in the order indicated
- Non-shaded areas do not require fasting
- Clinical Assessments, including AEs, and administration of mental health questionnaires may occur anytime before the OGTT is performed
- Mental Health questionnaires must be administered after clinical assessments

Abbreviations: AEs = adverse events; ECG = electrocardiogram; OGTT = 2-hour oral glucose tolerance test; PK = pharmacokinetics; PRO = patient-reported outcomes; SOA = schedule of activities.

10.9. Appendix 9: Management of Gastrointestinal Symptoms

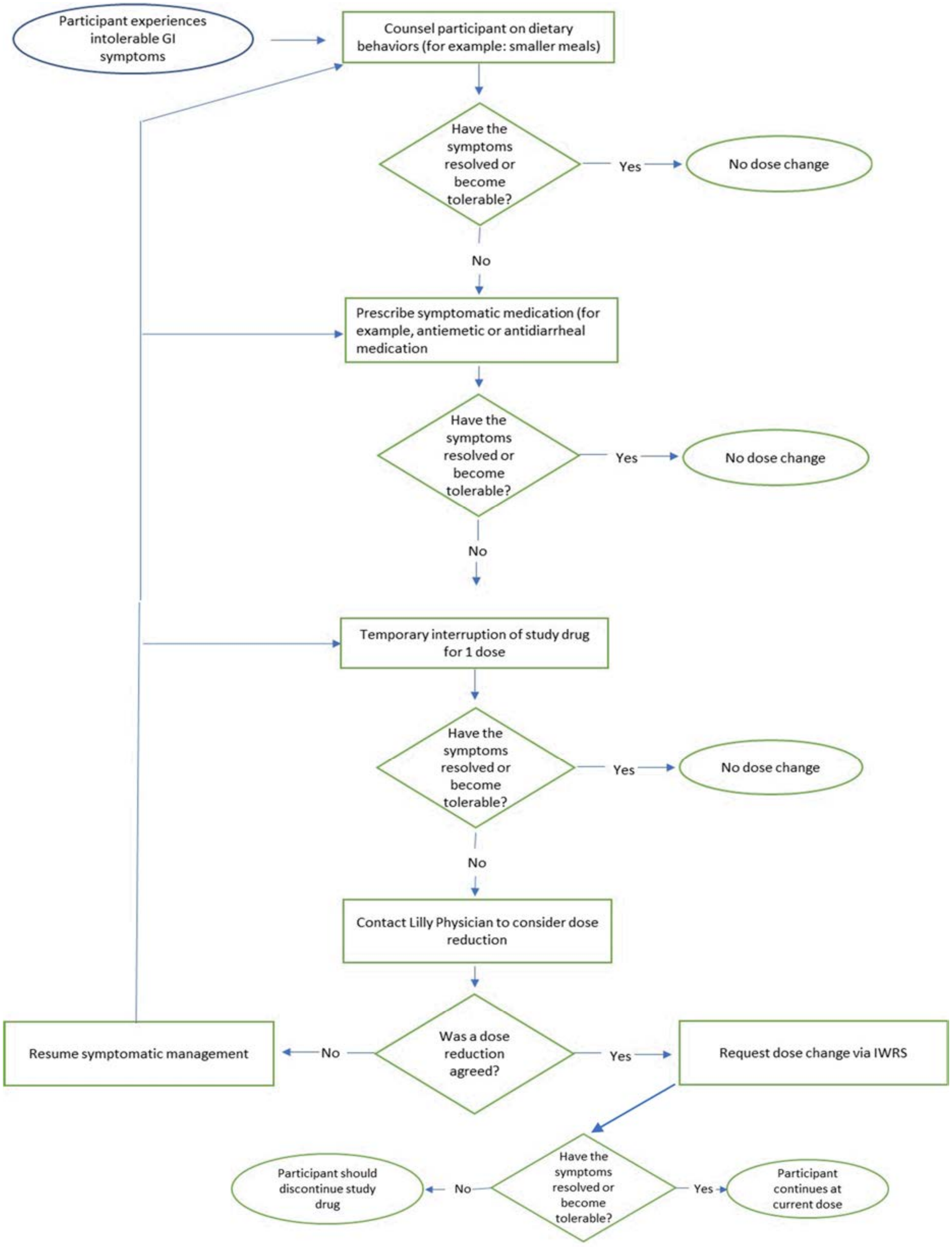

Abbreviations: GI = gastrointestinal; IWRS = interactive web-response system.

10.10. Appendix 10: Provisions for Changes in Study Conduct During Exceptional Circumstances

There may be times of exceptional circumstances, such as pandemics or natural disasters, which may cause disruptions to the conduct of the study. Possible disruptions are limitations in the ability to conduct study procedures or ability to have on-site visits occur by participants.

To mitigate the risk of participants missing visits, to allow participants to safely continue in the study, and to maintain the data integrity of the study in the case of an exceptional circumstance, sites may implement changes to the conduct of the study on a case-by-case basis following Sponsor's written approval (if permitted by local regulations). Good clinical practice compliance and minimization of risks to study integrity are important considerations. Ensuring the safety of study participants is the prevailing consideration.

Additional written guidance will be provided by the Sponsor in the event written approval is granted for changes in study conduct.

The following changes in study conduct captured in this Appendix will not be considered protocol deviations. Missing data will be captured as protocol deviation(s).

1. **Remote visit (telephone/telemedicine)**

   Telephone or IT-assisted virtual visits (telemedicine) to complete appropriate assessments are acceptable. The study site should capture the visit location and method with a specific explanation for any data missing because of missed in-person site visits in source document and eCRF. Examples of assessments to be completed in this manner include AEs and product complaints, concomitant medications, review study participant diary (including study drug compliance), review diet and exercise goals, C-SSRS (Since Last Visit Version), Self-Harm Supplement Form, Self-Harm Follow-up Form (if applicable), and PHQ-9.

2. **Mobile home health visit**

   Mobile visits may be performed at participants' homes when participants cannot travel to the site due to exceptional circumstances. These will be performed by a qualified home nursing service provider or trained site personnel, following Sponsor written approval (if permitted by local regulations). Procedures performed may include, but are not limited to, weight measurement, taking blood samples, conducting physical assessments, administering PROs, and collecting health information. Please note that requirements related to the reporting of SAEs remain unchanged. Every effort should be made for the participant to return to on-site visits as soon as reasonably possible, while ensuring the safety of the participant and investigational site staff.

   Additional consent from the participant will be obtained for those who participate in home health services.

3. **Investigational product and ancillary supplies (including participant diaries)**

   In cases when a participant is unable to come to site to receive trial supplies during normal on-site visit, the site should work with the Sponsor to determine appropriate actions to receive trial supplies. This may include a participant coming to the site to receive trial supplies only from site staff without full completion of a visit, a participant-approved

designee coming to the site to receive trial supplies on a participant's behalf, or delivery to a participant's home.

The following requirements must be met:

- Sponsor approves the alternative method of delivery, taking local regulatory requirements into consideration
- participant consents verbally to alternate method of delivery
- site/Sponsor confirms the participant's receipt of the trial supplies
- site/Sponsor confirm appropriate ethics review board notification
- alternate delivery of investigational product should be performed in a manner that does not compromise treatment blinding and ensures product integrity. The existing protocol requirements for product accountability remain unchanged
- when delivering supplies to a participant's home:
    - participant consent must include provision of any personal information
    - investigator/Sponsor should ensure oversight of the shipping process to ensure accountability and product quality (i.e., storage conditions and intact packaging upon receipt)
- additional instructions should be provided to the participant on how to return any unused or completed trial supplies

4. Local laboratory option

In exceptional circumstances, to ensure participant safety and with the Sponsor's prior written approval, local laboratory testing may be conducted in lieu of central laboratory testing. The local laboratory must be qualified in accordance with local regulations. Clinically significant laboratory findings must be recorded as an adverse event in the AE eCRF.

5. Screening period guidance

To ensure safety of study participants, laboratory values and other eligibility assessments taken at Visit 1 are valid for a maximum of 90 days. Therefore, the following rules will be applied for participants who are paused in an active, non-randomized status due to exceptional circumstances:

- Paused for less than 90 days from Visit 1 to Visit 3: the participant will proceed to the next study visit per the schedule of activities in the protocol and Visit 3 (randomization visit) must be conducted within 90 days from Visit 1.
    - The site should proceed with the next visit if eligibility criteria are confirmed and document the reason for delay in the eCRF.
    - Due to the pause in screening, sites should also reconfirm each participant's consent and document in the source documentation.
- Paused for more than 90 days from Visit 1 to Visit 3: The participant must be discontinued from the study and rescreened. The screening procedures per the schedule of activities in the protocol should be followed (starting at Visit 1) to ensure participant eligibility by randomization visit (Visit 3). Before rescreening, the

participant must sign a new ICF and receive a new identification number through IWRS.

6. **Increasing visit window(s) for primary and secondary endpoint visits**

- Participants should complete primary endpoint visit (Visit 21 or 99) and the final study endpoint visit (Visit 116 or 199) as per original schedule whenever possible and safe to do so, at the investigator's discretion. However, in order to maximize the ability for such on-site visits, minimize missing data, and preserve the intended conduct of the study, the visit windows may be brought forward no sooner than 14 days or extended up to 28 days, upon specific guidance from the Sponsor.
- For the following 2 secondary endpoint visits, Visit 8 (the key secondary endpoint visit) and Visit 12 (the 36-week interim analysis endpoint visit), the visit windows may be brought forward no sooner than 7 days or extended up to 7 days, upon specific guidance from the Sponsor.
- For participants requiring the visit windows to be extended, additional study drug may need to be provided to avoid study drug interruption and maintain overall integrity of the trial. Additional consent from the participant per local regulations will be obtained for those participants who will be dispensed additional study drug during the extended treatment period.

7. **Documentation**

   a. **Changes to study conduct**

Changes to study conduct will be documented as the following:

- Sites will need to identify and document the details of how all participants, visits, methods, and activities conducted were affected by exceptional circumstances. All dispensing/shipment records of IP and relevant communications, including delegation, should be filed with site trial records.
- The site should document the participant's verbal consent for having remote visits and remote dispensing of IP, ancillaries, and diaries, prior to implementation of these activities.
- Source document(s) that are generated at participant's home should be part of the investigator's source documentation and should be transferred to the site in a secure and timely manner.

   b. **Missing data and other protocol deviations**

The study site should capture specific explanation for any missing data and other protocol deviations in source documents and eCRF. While protocol deviations may be unavoidable in an exceptional circumstance, documentation of deviations and missing data will be important for data analysis and reporting.

8. **Informing ethical review boards (ERBs)**

The Sponsor and study investigators will notify ERBs as early as possible to communicate implementation of changes in study conduct due to exceptional circumstances. To protect the safety of study participants, urgent changes may be implemented before such communications are made, but all changes will be reported as early as possible following implementation.

10.11. Appendix 11: Abbreviations

Term	Definition
**ADA**	antidrug antibodies
**AE**	adverse event: Any untoward medical occurrence in a patient or clinical investigation subject administered a pharmaceutical product that does not necessarily have a causal relationship with this treatment. An adverse event can therefore be any unfavorable and unintended sign (including an abnormal laboratory finding), symptom, or disease temporally associated with the use of a medicinal (investigational) product, whether or not related to the medicinal (investigational) product.
**AESI**	adverse events (AEs) of special interest
**ALP**	alkaline phosphatase
**ALT**	alanine aminotransferase
**AST**	aspartate aminotransferase
**BG**	blood glucose
**BMI**	body mass index
**BMR**	basal metabolic rate
**BP**	blood pressure
**CHF**	congestive heart failure
**complaint**	A complaint is any written, electronic, or oral communication that alleges deficiencies related to the identity, quality, purity, durability, reliability, safety or effectiveness, or performance of a drug or drug delivery system.
**compliance**	Adherence to all study-related, good clinical practice (GCP), and applicable regulatory requirements.
**CONSORT**	Consolidated Standards of Reporting Trials
**CRF**	case report form
**CRP**	clinical research physician: Individual responsible for the medical conduct of the study. Responsibilities of the CRP may be performed by a physician, clinical research scientist, global safety physician or other medical officer.
**CSR**	Clinical Study Report
**C-SSRS**	Columbia-Suicide Severity Rating Scale
**CT**	computed tomography

**CVD**	cardiovascular disease
**DMC**	data monitoring committee
**DPP-4**	dipeptidyl-peptidase-4
**EAS**	efficacy analysis set
**ECG**	electrocardiogram
**ED**	early discontinuation of treatment
**EDC**	electronic data capture
**eCRF**	electronic case report form
**eGFR**	estimated glomerular filtration rate
**enroll**	The act of assigning a patient to a treatment. Patients who are enrolled in the study are those who have been assigned to a treatment.
**enter**	Patients entered into a study are those who sign the informed consent form directly or through their legally acceptable representatives.
**ERB**	ethical review board
**FAS**	full analysis set
**FG**	fasting glucose
**FSH**	follicle-stimulating hormone
**GCP**	good clinical practice
**GI**	gastrointestinal
**GIP**	glucose-dependent insulinotropic polypeptide
**GIPR**	glucose-dependent insulinotropic polypeptide receptor
**GLP-1**	glucagon-like peptide-1
**GLP-1R**	glucagon-like peptide-1 receptor
**GPGA**	Study I8F-MC-GPGA
**GPGB**	Study I8F-MC-GPGB
**GPGF**	Study I8F-MC-GPGF
**GPHK (SURMOUNT-1)**	Study I8F-MC-GPHK
**HbA1c**	hemoglobin A1c

**HDL**	high-density lipoprotein
**HR**	heart rate
**HRT**	hormonal replacement therapy
**IB**	Investigator's Brochure
**ICF**	informed consent form
**ICH**	The International Council for Harmonization of Technical Requirements for Pharmaceuticals for Human Use
**Informed consent**	A process by which a patient voluntarily confirms his or her willingness to participate in a particular study, after having been informed of all aspects of the study that are relevant to the patient's decision to participate. Informed consent is documented by means of a written, signed, and dated informed consent form.
**interim analysis**	An interim analysis is an analysis of clinical study data, separated into treatment groups, that is conducted before the final reporting database is created/locked.
**INR**	international normalized ratio
**IP**	investigational product: A pharmaceutical form of an active ingredient or placebo being tested or used as a reference in a clinical trial, including products already on the market when used or assembled (formulated or packaged) in a way different from the authorized form, or marketed products used for an unauthorized indication, or marketed products used to gain further information about the authorized form.
**IRB/IEC**	Institutional Review Boards/Independent Ethics Committees
**ITT**	intention to treat: The principle that asserts that the effect of a treatment policy can be best assessed by evaluating on the basis of the intention to treat a patient (that is, the planned treatment regimen) rather than the actual treatment given. It has the consequence that patients allocated to a treatment group should be followed up, assessed, and analyzed as members of that group irrespective of their compliance to the planned course of treatment.
**IWQOL-Lite-CT**	Impact of Weight on Quality of Life-Lite-Clinical trials
**IWRS**	interactive web-response system
**LDL**	low-density lipoprotein
**Lilly**	Eli Lilly and Company
**MAD**	multiple ascending dose
**MDD**	Major Depressive Disorder
**MedDRA**	Medical Dictionary for Regulatory Activities
**MEN**	multiple endocrine neoplasia

**MHP**	mental health professional
**MI**	myocardial infarction
**mITT**	modified intention-to-treat
**MMRM**	mixed-model for repeated measures
**MRI**	magnetic resonance imaging
**MTC**	medullary thyroid carcinoma
**NYHA**	New York Heart Association
**OGTT**	oral glucose tolerance test
**PC**	Product Complaints
**PGIS**	Patient Global Impression of status for physical activity
**PHQ-9**	Patient Health Questionnaire-9
**PK/PD**	pharmacokinetics/pharmacodynamics
**PROs/ePRO**	patient-reported outcomes
**QW**	once weekly
**SAD**	single ascending dose
**SAE**	serious adverse event
**SAP**	Statistical Analysis Plan
**SBP**	systolic blood pressure
**SC**	subcutaneous
**screen**	The act of determining if an individual meets minimum requirements to become part of a pool of potential candidates for participation in a clinical study.
**SD**	standard deviation
**SF-36v2**	Short-Form-36 Health Survey (SF-36), version 2
**SMQ**	standardized MedDRA queries
**SS**	safety analysis set
**SUSARs**	suspected unexpected serious adverse reactions
**T1DM**	type 1 diabetes mellitus

**T2DM**	type 2 diabetes mellitus
**TBL**	total bilirubin level
**TE**	treatment emergent
**TEAE**	treatment-emergent adverse event: An untoward medical occurrence that emerges during a defined treatment period, having been absent pretreatment, or worsens relative to the pretreatment state, and does not necessarily have to have a causal relationship with this treatment.
**TEE**	total energy exposure
**TSH**	thyroid-stimulating hormone
**ULN**	upper limit of normal

10.12. Appendix 12: Protocol Amendment History

The Protocol Amendment Summary of Changes Table for the current amendment is located directly before the Table of Contents (TOC).

Amendment a: (24-July-2020)

Overall Rationale for the Amendment

The GPHK clinical protocol has been amended to extend the safety follow-up period in order to better characterize the glycemic status of study participants. The overall changes and rationale for the changes made to this protocol are described in the following table. Note that minor edits have been made throughout the protocol, which are not captured in the amendment summary table.

Section # and Name	Description of Change	Brief Rationale
1.2 Schema<br>1.3.2 Schedule of Activities	Figure updated to reflect the extended safety follow-up period and Schedule of Activities changed Visit 802 to 17 weeks posttreatment period	The safety follow-up period has been extended to 17 weeks to address regulatory feedback from Type C advice meeting with FDA (26 June 2019).
9.5 Interim Analysis	Two more interim analyses were added and text revised	Additional interim analyses were requested by an external DMC to perform unblinded safety reviews semi-annually.<br>Details pertaining to the timing of interim analyses was added.
1.1 Synopsis<br>3 Objectives and Endpoint<br>9.1 Statistical Hypothesis<br>9.4.3.2 Key Secondary Analyses	A key secondary objective was added (time to onset of T2DM during the entire study) with the associated endpoint and statistical hypothesis	An additional key secondary objective was added per FDA request from Type C advice meeting with FDA (26 June 2019).
10.10 Appendix 10	An appendix describing mitigation for extenuating circumstances was added as Section 10.10 Appendix 10<br>The Abbreviation Appendix 10 is now Appendix 11	Language was added to address extenuating circumstances due to unforeseen widespread emergencies (such as pandemics or natural disasters).
9.4.3.1 Primary	A sentence related to selection of variance-covariance structure in	The details will be provided in

Section # and Name	Description of Change	Brief Rationale
Analysis	case of nonconvergence was deleted	the Statistical Analysis Plan.
1.3 Schedule of Activities	Allowable deviation (days) for Visit 21 was changed from +7 days to ±7 days	This change allows more flexibility for participants to schedule the primary study endpoint visit.
1.3 Schedule of Activities	Added measurement of waist circumference to Visit 199	The measurement was added to obtain 3 year waist circumference data for participants who discontinue the study treatment early but come to the 3-year endpoint visit.
4.1.1.2.3 Continued Follow-up for Participants with Prediabetes (Visits 101 through 116)	Measurement of waist circumference added to list of visit procedures	The text was added to align with changes in the Schedule of Activities regarding measurement of waist circumference at Visit 199.
1.3 Schedule of Activities	Added an activity entitled “register study visit in IWRS”	The activity was added to the SOA to ensure all visits are registered. This allows monitoring of study activities using real-time IWRS reports.
10.4 Appendix 4 Contraceptive Guidance and Collection of Pregnancy Information	Added text “more than 18 months”	The sentence was revised to maintain consistency with the text from the exclusion criteria.
10.4 Appendix 4 Contraceptive Guidance and Collection of Pregnancy Information	Note regarding follicle stimulating hormone levels used to confirm a postmenopausal state in women has been deleted	The note could lead to misinterpretation of inclusion criterion 4.

11. References

Adams TD, Davidson LE, Litwin SE, Kim J, Kolotkin RL, Nanjee MN, Gutierrez JM, Frogley SJ, Ibele AR, Brinton EA, Hopkins PN, McKinlay RM., Simper SC, Hunt SC. Weight and metabolic outcomes 12 years after gastric bypass. *N Engl J Med.* 2017;377(12):1143-1155.

Allison DB, Downey M, Atkinson RL, Billington CJ, Bray GA, Eckel RH, Finkelstein EA, Jensen MD, Tremblay A. Obesity as a disease: a white paper on evidence and arguments commissioned by the Council of The Obesity Society. *Obesity (Silver Spring).* 2008;16(6):1161-1177.

American Diabetes Association. In Standards of Medical Care in Diabetes-2019. Glycemic Targets: Standards of Medical Care in Diabetes-2019. *Diabetes Care*. 2019a;42(suppl 1):S61-S70.

American Diabetes Association. In Standards of Medical Care in Diabetes-2019. Classification and Diagnosis of Diabetes: Standards of Medical Care in Diabetes-2019. Diabetes Care 2019b; 42(suppl 1): S 13-S28.

[AMA] American Medical Association. American Medical Association House of Delegates Resolution: 420 (A-13) Recognition of Obesity as a Disease, 2013 pp 1-7. Available at: https://www.npr.org/documents/2013/jun/ama-resolution-obesity.pdf Accessed August 30, 2019.

Aroda VR, Ratner R. The safety and tolerability of GLP-1 receptor agonists in the treatment of type 2 diabetes: a review. *Diabetes Metab Res Rev*. 2011;27(6):528-542.

Baggio LL, Drucker DJ. Biology of incretins: GLP-1 and GIP. *Gastroenterol.* 2007;132(6):2131-2157.

Banks PA, Freeman ML; Practice Parameters Committee of the American College of Gastroenterology. Practice guidelines in acute pancreatitis. *Am J Gastroenterol.* 2006;101(10):2379-2400.

Coskun T, Sloop KW, Loghin C, Alsina-Fernandez J, Urva S, Bokvist KB, Cui X, Briere DA, Cabrera O, Roell WC, Kuchibhotla U, Moyers JS, Benson CT, Gimeno RE, D'Alessio DA, Haupt A. LY3298176, a novel dual GIP and GLP-1 receptor agonist for the treatment of type 2 diabetes mellitus: From discovery to clinical proof of concept. *Mol Metab*. 2018;18:3-14.

Council on Science and Public Health. Report of the Council on Science and Public Health. Is obesity a disease? (Resolution 115-A-12). Council on Science and Public Health. Report number: 3-A-13, 2013.

Danne T, Philotheou A, Goldman D, Guo X, Ping L, Cali A, Johnston P. A randomized trial comparing the rate of hypoglycemia – assessed using continuous glucose monitoring – in 125 preschool children with type 1 diabetes treated with insulin glargine or NPH insulin (the PRESCHOOL study). *Pediatr Diabetes*. 2013;14(8):593-601. Erratum in: *Pediatr Diabetes*. 2015;16(6):462.

Dolan P. Modeling valuations for EuroQol Health States. Medical Care 1997; 35(11):1095-1108.

Dombrowski SU, Knittle K, Avenell A, Araújo-Soares V, Sniehotta FF. Long term maintenance of weight loss with non-surgical interventions in obese adults: systematic review and meta-analyses of randomized controlled trials. *BMJ.* 2014;348:g2646.

[EMA] European Medicines Agency. Guideline on clinical evaluation of medicinal products used in weight management. Committee for Medicinal Products for Human Use. June 2016. http://www.ema.europa.eu/docs/en_GB/document_library/Scientific_guideline/2016/07/WC500209942.pdf Accessed November 19, 2018.

EuroQoL Group. EQ-5D-5L User guide: basic information on how to use the EQ-5D-5L instrument. Version 2.1. Available at: https://euroqol.org/wp-content/uploads/2016/09/EQ-5D-5L_UserGuide_2015.pdf. Published April 2015. Accessed January 22, 2019.

FAO/WHO/UNU. Human energy requirements. Report of a joint FAO/WHO/UNU expert , Pogosova N, Raubenheimer PJ, Shaw JE, Sheu WHH, Temelkova-Kurktschiev T, for the REWIMNDconsultation. FAO: food and nutrition technical report series 1. Rome: FAO/WHO/UNU; 2004.

[FDA] Food and Drug Administration. Guidance for Industry. Developing products for weight management. February 2007. Available at: https://www.fda.gov/downloads/Drugs/Guidances/ucm071612.pdf. Accessed November 19, 2018.

Frias JP, Nauck MA, Van J, Kutner ME, Cui X, Benson C, Urva S, Gimeno RE, Milicevic Z, Robins D, Haupt A. Efficacy and safety of LY3298176, a novel dual GIP and GLP-1 receptor agonist, in patients with type 2 diabetes: a randomised, placebo-controlled and active comparator-controlled phase 2 trial. *Lancet.* 2018;392(10160):2180-2193.

Gerstein HC, Colhoun HM, Dagenais GR, Diaz R, Lakshmanan M, Pais P, Pobstfield J, Riesmeyer JS, Riddle MC, Ryden L, Xavier D, Atisso CM, Dyal L, Hall S, Rao-Melacini P, Wong G, Avezum A, Basile J, Chung N, Conget I, Cushman WC, Franek E, Hancu N, Hanefeld M, Holt S, Jansky P, Keltai M, Lanas F, Leiter LA, Lopez-Jaramillo P, Munoz EGC, Pirags V, Pogosova N, Raubenheimer PJ, Shaw JE, Sheu WH-H, Temelkova-Kurktschiev T, for the REWIND Investigators. Dulaglutide and cardiovascular outcomes in type 2 diabetes (REWIND): a double-blind, randomized placebo-controlled trial. *Lancet* 2019;394(10193):121-130.

Jensen MD, Ryan DH, Donato KA, Aprovian, CM, Arrd, JD, Comuzzie AG, Hu FB, Hubbard VS, Jakicic JM, Kushner RF, Loria CM, Millen BE, Nonas CA, Pi-Sunyer FX, Stevens J, Stevens VJ, Wadden TA, Wolfe BM, Uanovski SZ. Executive summary: guidelines (2013) for the management of overweight and obesity in adults: a report of the American College of Cardiology/American Heart Association Task Force on Practice Guidelines and the Obesity Society published by the Obesity Society and American College of Cardiology/American Heart Association Task Force on Practice Guidelines. Based on a systematic review from the Obesity Expert Panel, 2013. *Obesity.* 2014;22:(suppl 2):S5-S39.

Knowler WC, Barrett-Connor E, Fowler SE, Hamman RF, Lachin JM, Walker EA, Nathan DM; Diabetes Prevention Program Research Group. Reduction in the incidence of type 2 diabetes with lifestyle intervention or metformin. *N Engl J Med.* 2002;346(6):393-403.

Koizumi M, Takada T, Kawarada Y, Hirata K, Mayumi T, Yoshida M, Sekimoto M, Hirota M, Kimura Y, Takeda K, Isaji S, Otsuki M, Matsuno S; JPN. JPN Guidelines for the management of acute pancreatitis: diagnostic criteria for acute pancreatitis. *J Hepatobiliary Pancreat Surg*. 2006;13(1):25-32.

Kolotkin RL, Ervin CM, Meincke HH, Hojbjerre L, Fehnel SE. Development of a clinical trials version of the Impact of Weight on Quality of Life-Lite questionnaire (IWQoL-Lite Clinical Trials Version): results from two qualitative studies. *Clin Obes.* 2017;7(5):290-299.

Kolotkin RL, Williams V, Ervin, CM, Meincke HH, Qin S, Smith LvH, Fehnel SE. Validation of a new measure of quality of life in obesity trials: IWQOL – Lite Clinical Trials Version. Poster presented at: Obesity Week 2018; November 13, 2018; Nashville, TN.

Kroenke K, Spitzer RL, Williams JB. The PHQ-9: validity of a brief depression severity measure. *J Gen Intern Med.* 2001;16(9):606-616.

le Roux CW, Astrup A, Fujioka K, Greenway F, Lau DCW, Van Gaal L, Ortiz RV, Wilding JPH, Skjoth TV, Manning LS, Pi-Sunyer X for the SCALE Obesity Prediabetes NN8022-1839 Study Group. 3 years of liraglutide versus placebo for type 2 diabetes risk reduction and weight management in individuals with prediabetes: a randomized, double-blind trial. *Lancet.* 2017;389(10077):1399-1409.

Li G, Zhang P, Wang J, An Y, Gong Q, Gregg EW, Yang W, Zhang B, Shuai Y, Hong J, Engelgau MM, Li H, Roglic G, Hu Y, Bennett PH. Cardiovascular mortality, all-cause mortality, and diabetes incidence after lifestyle intervention for people with impaired glucose tolerance in the Da Qing Diabetes Prevention Study: a 23-year follow-up study. Lancet Diabetes Endocrinol. 2014;2(6):474-480.

Luppino FS, de Wit LM, Bouvy PF, Stijnen T, Cuijpers P, Penninx BW, Zitman FG. Overweight, obesity, and depression: a systematic review and meta-analysis of longitudinal studies. *Arch Gen Psychiatry.* 2010;67(3):220-229.

Marso SP, Daniels GH, Brown-Frandsen K, Kirstensen P, Mann JFE, Nauck MA, Nissen SE, Pocock S, Poulter NR, Tavn LS, Steinberg WM, Stockner M, Zinman B, Bergenstal RM, Buse JB. For the LEADER Steering Committee. Liraglutide and cardiovascular outcomes in type 2 diabetes. *N Engl J Med* 2016a; 375:311-322.

Marso SP, Bain SC, Consoli A, Eliaschewitz FG, Jodar E, Leiter LA, Lingvay I, Rosenstock J, Seufert J, Warren M, Woo V, Hansen O, Holst AG, Pettersson J, Vilsboll T, for the SUSTAIN-6 Investigators. Semaglutide and cardiovascular outcomes in patients with type 2 diabetes. *N Engl J Med* 2016b; 375:1834-1844.

Maruish ME, ed. User’s manual for the SF-36v2 Health Survey. 3rd ed. Lincoln, RI: Quality Metric Incorporated; 2011.

Mertens IL, Van Gaal LF. Overweight, obesity, and blood pressure: the effects of modest weight reduction. *Obes Res.* 2000;8(3):270-278.

Nauck MA, Meier JJ, Schmidt WE. Incretin-based glucose-lowering medications and the risk of acute pancreatitis and/or pancreatic cancer: Reassuring data from cardio-vascular outcome trials. *Diabetes Obes Metab*. 2017;19(9):1327-1328.

SAXENDA® [EMA summary of product characteristics (SmPC)]. Novo Nordisk, Bagsvaerd, Denmark. Available at: https://ec.europa.eu/health/documents/community-register/2015/20150323131125/anx_131125_en.pdf. Accessed April 10, 2019.

SAXENDA® [package insert] Novo Nordisk, Bagsvaerd, Denmark. Available at: https://www.accessdata.fda.gov/drugsatfda_docs/label/2014/206321Orig1s000lbl.pdf. Accessed March 2, 2019.

Skow MA, Bergmann, NC, Knop FK. Diabetes and obesity treatment based on dual incretin receptor activation: "twincretins". *Diabetes Obes Metab.* 2016;18(9):847-854.

Steinberg WM, Buse JB, Ghorbani MLM, Ørsted DD, Nauck MA; LEADER Steering Committee; LEADER Trial Investigators. Amylase, lipase, and acute pancreatitis in people with type 2 diabetes treated with liraglutide: results from the LEADER Randomized Trial. *Diabetes Care.* 2017a;40(7):966-972.

Steinberg WM, Rosenstock J, Wadden TA, Donsmark M, Jensen CB, DeVries JH. Impact of liraglutide on amylase, lipase, and acute pancreatitis in participants with overweight/obesity and normoglycemia, prediabetes, or type 2 diabetes: secondary analyses of pooled data from the SCALE clinical development program. *Diabetes Care.* 2017b;40(7):839-848.

Tomlinson B, Hu M, Zhang Y, Chan P, Liu ZM. An overview of new GLP-1 receptor agonists for type 2 diabetes. *Expert Opin Investig Drugs*. 2016;25(2):145-158.

van Bloemendaal L, Ten Kulve JS, la Fleur SE, Ijzerman RG, Diamant M. Effects of glucagon-like peptide 1 on appetite and body weight: focus on the CNS. *J Endocrinol*. 2014;221(1):T1-T16.

Warkentin LM, Das D, Majumdar SR, Johnson JA, Padwal RS. The effect of weight loss on health-related quality of life: systematic review and meta-analysis of randomized trials. *Obes Rev.* 2014;15(3):169-182.

Weinberg ME, Bacchetti P, Rushakoff RJ. Frequently repeated glucose measurements overestimate the incidence of inpatient hypoglycemia and severe hyperglycemia. *J Diabetes Sci Technol*. 2010;4(3):577-582.

Leo Document ID = 8dd79a8d-d134-4b80-afb3-6ce848a2570c

Approver: PPD

Approval Date & Time: 01-Oct-2021 16:14:59 GMT

Signature meaning: Approved

Approver: PPD

Approval Date & Time: 04-Oct-2021 15:48:26 GMT

Signature meaning: Approved
