# Supplementary material for: Psychiatric Safety of Tirzepatide in People With Obesity and No Known Major Psychopathology: A Post Hoc Analysis of SURMOUNT
Source: Obesity (Silver Spring). 2026 Jan 15;34(3):565–78. doi: 10.1002/oby.70122 (PMC12933222; doi:10.1002/oby.70122)
Supplement: Supplementary file 4 — Supplement S4: Full study protocol for SURMOUNT‐2. [file OBY-34-565-s003.pdf]

SUPPLEMENT 4

Psychiatric Safety of Tirzepatide in People with Obesity and No Known Major Psychopathology: A Pooled Post Hoc Analysis of the SURMOUNT-1, SURMOUNT-2, and SURMOUNT-3 Trials

Thomas A. Wadden, PhD, Maria A. Oquendo, MD, PhD, Robert F. Kushner, MD, Dachuang Cao, PhD, MS, Chrisanthi A. Karanikas, MS, Afton Kechter, PhD, Madhumita A. Murphy, MD, MS, MBA

This supplement provides data on: Study protocol for the SURMOUNT-2 trial.

Title Page

Confidential Information

The information contained in this document is confidential and is intended for the use of clinical investigators. It is the property of Eli Lilly and Company or its subsidiaries and should not be copied by or distributed to persons not involved in the clinical investigation of tirzepatide (LY3298176), unless such persons are bound by a confidentiality agreement with Eli Lilly and Company or its subsidiaries.

Note to Regulatory Authorities: This document may contain protected personal data and/or commercially confidential information exempt from public disclosure. Eli Lilly and Company requests consultation regarding release/redaction prior to any public release. In the United States, this document is subject to Freedom of Information Act (FOIA) Exemption 4 and may not be reproduced or otherwise disseminated without the written approval of Eli Lilly and Company or its subsidiaries.

Protocol Title: Efficacy and Safety of Tirzepatide Once Weekly in Participants with Type 2 Diabetes Who Have Obesity or Are Overweight: A Randomized, Double-Blind, Placebo-Controlled Trial (SURMOUNT-2)

Protocol Number: I8F-MC-GPHL

Amendment Number: This is the initial protocol.

Compound: LY3298176

Study Phase: 3

Short Title: Effect of Tirzepatide versus Placebo in Participants with Type 2 Diabetes Who Have Obesity or Are Overweight (SURMOUNT-2)

Acronym: SURMOUNT-2

Sponsor Name: Eli Lilly and Company

Legal Registered Address: Indianapolis, Indiana USA 46285

Regulatory Agency Identifier Numbers(s)

IND: 139721

Approval Date: Protocol Electronically Signed and Approved by Lilly on date provided below.

Approval Date: 23-Nov-2020 GMT

Medical Monitor Name and Contact Information will be provided separately.

Table of Contents

1. Protocol Summary 6
1.1. Synopsis 6
1.2. Schema 12
1.3. Schedule of Activities (SoA) 13

2. Introduction 20
2.1. Study Rationale 20
2.2. Background 21
2.3. Benefit/Risk Assessment 22

3. Objectives and Endpoints 23

4. Study Design 26
4.1. Overall Design 26
4.1.1. Overview of Study Periods 26
4.1.2. Study Procedures 30
4.2. Scientific Rationale for Study Design 30
4.3. Justification for Dose 31
4.4. End of Study Definition 32

5. Study Population 33
5.1. Inclusion Criteria 33
5.2. Exclusion Criteria 34
5.3. Lifestyle Considerations 39
5.3.1. Meals and Dietary Restrictions 39
5.3.2. Physical Activity 40
5.3.3. Diabetes Education 41
5.4. Screen Failures 41

6. Study Intervention 42
6.1. Study Intervention(s) Administered 42
6.1.1. Medical Devices 42
6.2. Preparation/Handling/Storage/Accountability 43
6.3. Measures to Minimize Bias: Randomization and Blinding 43
6.4. Study Intervention Compliance 44
6.5. Concomitant Therapy 45
6.5.1. Management of T2DM at randomization 45
6.5.2. Initiation of New Antihyperglycemic Medication 45
6.5.3. Management of T2DM after randomization 46
6.6. Dose Modification 47
6.6.1. Tirzepatide 47
6.6.2. Management of Participants with Gastrointestinal Symptoms 47
6.7. Intervention after the End of the Study 47

7. Discontinuation of Study Intervention and Participant Discontinuation/Withdrawal 48
7.1. Discontinuation of Study Intervention 48

7.1.1. Temporary Study Drug Discontinuation 50
7.2. Participant Discontinuation/Withdrawal from the Study 51
7.2.1. Participant disposition and timing of safety follow-up..53
7.2.2. Discontinuation of Inadvertently Enrolled Participants 54
7.3. Lost to Follow-up 54

8. Study Assessments and Procedures 55
8.1. Efficacy Assessments 55
8.1.1. Primary Efficacy Assessments 55
8.1.2. Secondary Efficacy Assessments 55
8.1.3. Patient-Reported Outcomes Assessments..55
8.2. Safety Assessments 57
8.2.1. Physical Examinations 57
8.2.2. Vital Signs 57
8.2.3. Electrocardiograms 57
8.2.4. Clinical Safety Laboratory Assessments 58
8.2.5. Safety Monitoring 59
8.2.6. Depression, Suicidal Ideation, and Behavior Risk Monitoring...................................61
8.3. Adverse Events, Serious Adverse Events, and Product Complaints 61
8.3.1. Timing and Mechanism for Collecting Events 62
8.3.2. Special Safety Topics 64
8.4. Treatment of Overdose 70
8.5. Pharmacokinetics...70
8.6. Pharmacodynamics 70
8.7. Genetics 71
8.8. Biomarkers 71
8.9. Immunogenicity Assessments 72
8.10. Health Economics..72

9. Statistical Considerations 73
9.1. Statistical Hypotheses 73
9.2. Sample Size Determination..74
9.3. Populations for Analyses 74
9.4. Statistical Analyses 75
9.4.1. General Considerations 75
9.4.2. Treatment Group Comparability 76
9.4.3. Efficacy Analyses 76
9.4.4. Safety Analyses 78
9.4.5. Pharmacokinetic/Pharmacodynamic Analyses 79
9.4.6. Evaluation of Immunogenicity...79
9.4.7. Other Analyses 80
9.5. Interim Analyses..80
9.6. Data Monitoring Committee 80

10. Supporting Documentation and Operational Considerations 81
10.1. Appendix 1: Regulatory, Ethical, and Study Oversight Considerations 81

10.1.1. Regulatory and Ethical Considerations 81
10.1.2. Informed Consent Process 82
10.1.3. Data Protection 82
10.1.4. Committees Structure 83
10.1.5. Dissemination of Clinical Study Data 83
10.1.6. Data Quality Assurance 83
10.1.7. Source Documents 84
10.1.8. Study and Site Start and Closure 85
10.1.9. Publication Policy 85
10.2. Appendix 2: Clinical Laboratory Tests 86
10.3. Appendix 3: Laboratory Assessments for Hypersensitivity Events 89
10.4. Appendix 4: Adverse Events: Definitions and Procedures for Recording, Evaluating, Follow-up, and Reporting 91
10.4.1. Definition of AE 91
10.4.2. Definition of SAE 92
10.4.3. Definition of Product Complaint 93
10.4.4. Recording and Follow-Up of AE and/or SAE and Product Complaints 93
10.4.5. Reporting of SAEs 95
10.4.6. Regulatory Reporting Requirements 96
10.5. Appendix 5: Contraceptive Guidance and Collection of Pregnancy Information 97
10.6. Appendix 6: Liver Safety: Suggested Actions and Follow-up Assessments 101
10.7. Appendix 7: Protocol GPHL Standardized Protocols for the Measurement of Height, Weight, Waist Circumference, Vital Signs, and Electrocardiogram 103
10.8. Appendix 8: Suggested Visit Structure 105
10.9. Appendix 9: Management of Gastrointestinal Symptoms 106
10.10. Appendix 10: World Health Organization Classification of Diabetes and Diagnostic Criteria 107
10.11. Appendix 11: Provisions for Changes in Study Conduct During Exceptional Circumstances 108
10.12. Appendix 12: Abbreviations 112
11. References 117

1. Protocol Summary

1.1. Synopsis

Protocol Title: Efficacy and Safety of Tirzepatide Once Weekly in Participants with Type 2 Diabetes who have Obesity or are Overweight: A Randomized, Double-Blind, Placebo-Controlled Trial (SURMOUNT-2)

Short Title: Effect of Tirzepatide versus Placebo in Participants with Type 2 Diabetes Who have Obesity or are Overweight (SURMOUNT-2)

Rationale:

Obesity is a chronic disease whose increasing prevalence is a public health concern associated with rising incidence of type 2 diabetes mellitus (T2DM), increased risk for premature death and increased risk for some cancers (AMA 2013; Council on Science and Public Health 2013; Lauby-Secretan et al. 2016).

For patients with T2DM who have obesity or are overweight, efforts targeting weight loss, including lifestyle, medication, and surgical interventions, are recommended (Davies 2018) because there is strong and consistent evidence that obesity management is beneficial in the treatment of type 2 diabetes and weight-related comorbidities (ADA 2020). Lifestyle changes that result in modest and sustained weight loss produce clinically meaningful reductions in blood glucose (BG), hemoglobin A1c (HbA1c), and triglycerides. Greater weight loss produces even greater benefits, including reductions in blood pressure, improvements in low-density lipoproteins (LDL) and high-density lipoproteins (HDL) cholesterol, and reductions in the need for medications to control BG, blood pressure, and lipids, and may even result in achievement of glycemic goals in the absence of glucose-lowering agent use in some patients (UKPDS Group 1990; Pastors et al. 2002; Wing et al. 2011; Rothberg et al. 2017; ADA 2020).

The gut incretin hormones, glucose-dependent insulinotropic polypeptide (GIP) and glucagon-like peptide-1 (GLP-1) are secreted after meal ingestion and mediate the incretin effect. Both hormones have effects on endocrine cells in the pancreas, increasing insulin biosynthesis and secretion, and modifying glucagon secretion (Skow et al. 2016). Based on these properties, several GLP-1 receptor (GLP-1R) agonists have been approved for pharmacological treatment of T2DM (Tomlinson et al. 2016).

In addition to its pancreatic effects, GLP-1R activation decreases gut motility, slows gastric emptying, and promotes satiety (presumably through a combination of GLP-1R activation in the central and peripheral nervous systems), thereby regulating food intake and body weight (Baggio and Drucker 2007). The US Food and Drug Administration (FDA) and the European Medicines Agency (EMA) approved the GLP-1R agonist liraglutide for the treatment of overweight and obesity (SAXENDA® package insert, 2014; SAXENDA summary of product characteristics, 2015).

Preclinical data indicate that GIP also exerts effects on appetite regulation and food intake, on adipose tissue, and on peripheral energy metabolism. Although studies evaluating effects of GIP on body weight have yielded equivocal results, GIP receptor (GIPR) activation may play a role in body weight regulation and targeting both the GLP-1R and the GIPR simultaneously could

potentially result in additive or synergistic effects of the 2 incretins on body weight (Coskun et al. 2018).

Tirzepatide is a 39-amino acid synthetic peptide with agonist activity at both the GIPR and GLP-1R. Its structure is based on the GIP sequence and includes a C20 fatty diacid moiety (Coskun et al. 2018). It is administered once-weekly (QW) by subcutaneous (SC) injection.

As a dual GIP/GLP-1R agonist, tirzepatide could exceed the efficacy of selective GLP-1R agonists by recruiting metabolically active tissues not targeted by selective GLP-1R agonists (for example, adipose tissue as indicated by the observation of increased energy utilization) (Müller et al. 2018) and has the potential to reach higher efficacy in target tissues that express both GIPR and GLP-1R. Therefore, tirzepatide has the potential to impact several aspects of energy regulation and be a treatment of overweight and obesity.

Compared to people without diabetes, patients with T2DM and obesity or overweight often respond less favorably to weight-management treatments (Pi-Sunyer, 2005) and may face unique safety issues such as risk for hypoglycemia following weight loss, if the dose of certain background medications for the management of hyperglycemia is not appropriately adjusted. Therefore, it is important to conduct a dedicated Phase 3, randomized, double-blind, placebo-controlled study in patients with T2DM and obesity or overweight to evaluate the efficacy and safety of novel chronic weight management products. In addition, weight loss should be demonstrated over the course of at least 1 year before a product can be considered as effective for weight management (FDA 2007).

Study 18F-MC-GPHL is a Phase 3, multicenter, randomized, parallel, placebo-controlled, double-blinded, 72-week study that will investigate the effects of treatment with tirzepatide 10 mg and 15 mg QW compared with placebo, in conjunction with a reduced-calorie diet and increased physical activity, on weight management in participants with T2DM who have obesity (body mass index [BMI] $\geq$30 kg/m^2) or are overweight (BMI $\geq$27 kg/m^2).

Objectives and Endpoints

Objectives	Endpoints
**Primary**	
To demonstrate that tirzepatide 10 mg QW is superior to placebo at 72 weeks for:<br>• Percent change in body weight AND<br>• Proportion of participants with ≥5% body weight reduction<br><br>AND/OR<br><br>To demonstrate that tirzepatide 15 mg QW is superior to placebo at 72 weeks for:<br>• Percent change in body weight AND<br>• Proportion of participants with ≥5% body weight reduction	• Mean percent change in body weight from randomization<br>• Percentage of study participants who achieve ≥5% body weight reduction from randomization
**Key Secondary (controlled for type I error), by dose analysis**	
For QW tirzepatide 10 mg and/or 15 mg doses, to demonstrate superiority to placebo in change from randomization for the following (measured at 72 weeks):	
• Body weight	• Percentage of participants who achieve:<br>○ ≥10% body weight reduction<br>○ ≥15% body weight reduction
• Glycemic control	• Mean change in HbA1c (%)<br>• Percentage of participants who achieve HbA1c <7%<br>• Mean change in fasting glucose (mg/dL)
• Waist circumference	• Mean change in waist circumference (cm)
**Key secondary (controlled for type I error), pooled dose analysis**	

For QW tirzepatide (all doses combined), to demonstrate superiority to placebo in change from randomization for the following (measured at 72 weeks):<br>• Lipid parameters	• Mean change in fasting<br>o Total cholesterol (mg/dL)<br>o LDL-cholesterol (mg/dL)<br>o Triglycerides (mg/dL)
• SBP	• Mean change in SBP (mmHg)
**Additional Secondary, by dose analysis**	
For QW tirzepatide 10 mg and/or 15 mg doses, to demonstrate superiority to placebo in change from randomization for the following (measured at 72 weeks):<br>• Body weight	• Mean change in absolute body weight (kg)<br>• Mean change in BMI (kg/m^2)
• Glycemic control	• Percentage of study participants who achieve HbA1c ≤6.5%<br>• Percentage of study participants who achieve HbA1c <5.7%
• Insulin	• Mean change in fasting insulin (pmol/L)
• Patient-Reported Outcomes	• Mean change in<br>o SF-36v2 acute form Physical Functioning domain score<br>o IWQOL-Lite-CT Physical Function composite score
**Additional Secondary, pooled analysis**	

For QW tirzepatide (all doses combined), to demonstrate superiority to placebo in change from randomization for the following (measured at 72 weeks):<br>• Lipid parameters<br>• DBP	• Mean change in fasting<br>o HDL-cholesterol (mg/dL)<br>o VLDL-cholesterol (mg/dL)<br>o Free fatty acids (mg/dL)<br>• Mean change in DBP (mmHg)
**Pharmacokinetics/Pharmacodynamics**<br>• To characterize the population PK of tirzepatide and explore the relationships between the tirzepatide concentration and efficacy, safety, and tolerability measures	• Population PK and PD parameters

Abbreviations: BMI = body mass index; DBP = diastolic blood pressure; HbA1c = hemoglobin A1c; HDL = high-density lipoprotein; IWQOL-Lite-CT = Impact of Weight on Quality of Life-Lite-Clinical Trials Version; LDL = low-density lipoprotein; PD = pharmacodynamics; PK = pharmacokinetics; QW = once weekly; SBP = systolic blood pressure; SF-36v2 acute form = Short Form-36 Health Survey version 2 acute form; VLDL = very low-density lipoprotein.

Overall Design:

Study I8F-MC-GPHL is a Phase 3, multicenter, randomized, parallel, placebo-controlled, double-blinded 72-week study that will investigate the safety and efficacy of treatment with tirzepatide 10 mg and 15 mg QW SC compared with placebo QW, in conjunction with a reduced-calorie diet and increased physical activity, on weight management in participants with T2DM who have obesity (BMI ≥30 kg/m^2) or are overweight (BMI ≥27 kg/m^2).

Disclosure Statement:

This is a parallel group treatment study that is participant and investigator blinded.

Number of Participants:

A total of approximately 900 participants will be randomized in a 1:1:1 ratio to 10 mg tirzepatide (300 participants), 15 mg tirzepatide (300 participants), or placebo (300 participants). An upper limit of 70% enrollment of women will be used to ensure a sufficiently large sample of men. In addition, an upper limit of 30% enrollment of participants treated with sulfonylurea will be used to allow sufficient enrollment of participants treated with other antihyperglycemic medications (AHMs).

Intervention Groups and Duration:

Study participants will be randomized in a double-blind 1:1:1 ratio (tirzepatide 10 mg QW, tirzepatide 15 mg QW, and placebo), stratified by

- country
- sex, and
- type of baseline AHMs (classified according to its potential effect on body weight).

All participants will undergo a 3-week screening period, a 72-week treatment period (including a 20-week dose escalation and a 52-week dose maintenance period), and a 4-week safety follow-up period.

Data Monitoring Committee: No

1.2. Schema

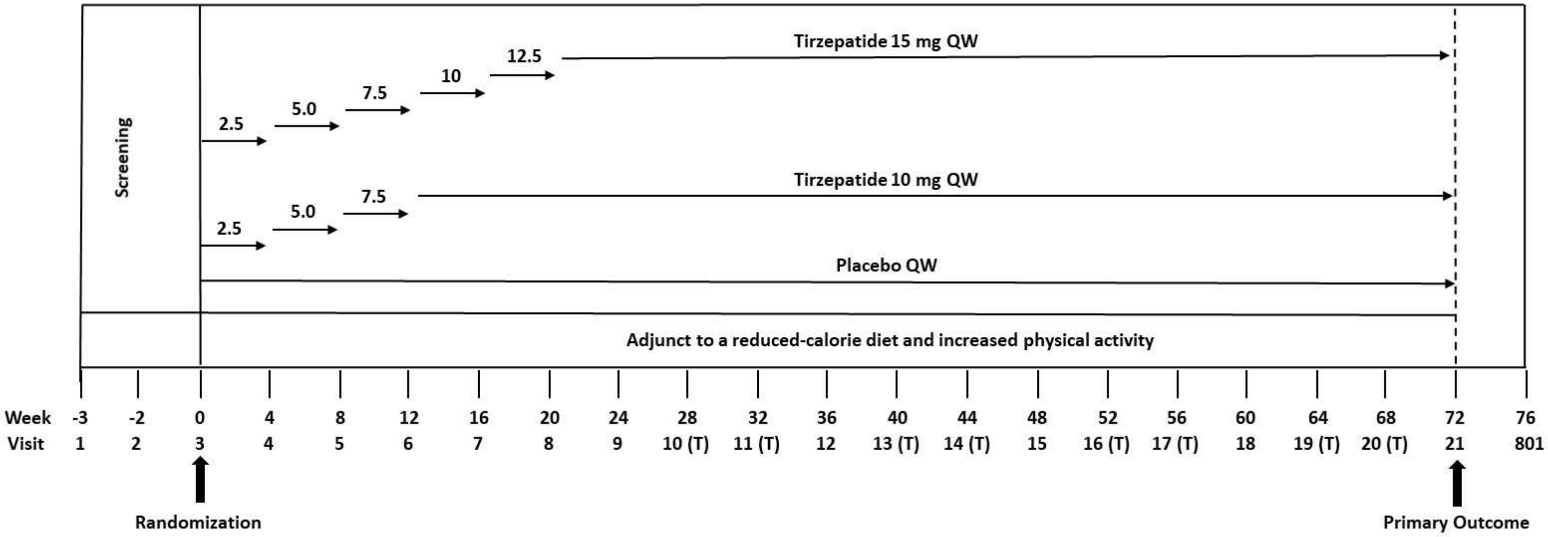

Abbreviations: QW = once weekly, (T) = telephone visit.

1.3. Schedule of Activities (SoA)

The Schedule of Activities described below should be followed for all participants enrolled in Study GPHL. However, for those participants whose participation in this study is affected by exceptional circumstances (such as pandemics or natural disasters), please refer to Section 10.11 Appendix 11 for additional guidance.

Visit	1	2	3	4	5	6	7	8	9	10	11	12	13	14	15	16	17	18	19	20	21	99	ED	801
**Week of Treatment**	-3	-2	0	4	8	12	16	20	24	28	32	36	40	44	48	52	56	60	64	68	72	72		4 wks post end of TxP
**Allowable Interval Tolerance (days)**		±3	±7	±3	+7	±3	+7	±3	±3	±3	±3	+7	±3	±3	±3	±3	±3	±3	±3	±3	±7	±7		±3
**Fasting Visit**	X		X	X	X	X	X	X	X			X			X			X			X	X	X	X
**Telephone Visit**										X	X		X	X		X	X		X	X				
Informed consent	X																							
Inclusion and exclusion criteria review	X	X	X																					
Preexisting conditions and medical history, including relevant surgical history	X																							
	Medical history includes assessment of preexisting conditions (for example, history of gallbladder disease, cardiovascular disease, and medullary thyroid carcinoma) and substance usage (such as alcohol and tobacco).																							
Concomitant medications	X	X	X	X	X	X	X	X	X	X	X	X	X	X	X	X	X	X	X	X	X	X	X	X
Adverse events and product complaints	X	X	X	X	X	X	X	X	X	X	X	X	X	X	X	X	X	X	X	X	X	X	X	X
**Physical Evaluation or Clinical Assessments**																								
Height	X																							
Weight	X		X	X	X	X	X	X	X			X			X			X			X	X	X	X
	Weight measurements should be obtained per the detailed protocol guidance in Section 10.7. Body weight must be measured in the fasting state. If the participant is not fasting, the participant should be called in for a new visit within the visit window to have the fasting body weight measured.																							
Waist circumference			X	X	X	X	X	X	X			X			X			X			X	X	X	X
Vital signs (systolic and diastolic blood pressure, pulse rate)	X		X	X	X	X	X	X	X			X			X			X			X		X	X
	Vital sign measurements should be taken before obtaining an ECG tracing and before collection of blood samples for laboratory testing, per the instruction in Section 10.7.																							

**Visit**	**1**	**2**	**3**	**4**	**5**	**6**	**7**	**8**	**9**	**10**	**11**	**12**	**13**	**14**	**15**	**16**	**17**	**18**	**19**	**20**	**21**	**99**	**ED**	**801**
**Week of Treatment**	**-3**	**-2**	**0**	**4**	**8**	**12**	**16**	**20**	**24**	**28**	**32**	**36**	**40**	**44**	**48**	**52**	**56**	**60**	**64**	**68**	**72**	**72**		**4 wks post end of TxP**
**Allowable Interval Tolerance (days)**		**±3**	**±7**	**±3**	**+7**	**±3**	**+7**	**±3**	**±3**	**±3**	**±3**	**+7**	**±3**	**±3**	**±3**	**±3**	**±3**	**±3**	**±3**	**±3**	**±7**	**±7**		**±3**
**Fasting Visit**	**X**		**X**	**X**	**X**	**X**	**X**	**X**	**X**			**X**			**X**			**X**			**X**	**X**	**X**	**X**
**Telephone Visit**										**X**	**X**		**X**	**X**		**X**	**X**		**X**	**X**				
Dilated Fundoscopic Examination		X																						
	For all participants who remain eligible after Visit 1, a dilated fundoscopic examination will be performed by an eye care professional (ophthalmologist or optometrist) between Visit 2 and Visit 3 to exclude participants with proliferative diabetic retinopathy, diabetic macular edema, or nonproliferative diabetic retinopathy that requires acute treatment. The results from this examination will be recorded on a specific eCRF page (Fundoscopy Exam) as a baseline measure of retinopathy. Follow-up dilated fundoscopic examination should be performed when clinically indicated, and the results recorded on the specific eCRF page.																							
Physical Examination	X																							
Electrocardiogram			X						X												X		X	X
	ECG measurements should be obtained per the instructions in Section 10.7. Electrocardiograms should be obtained prior to collection of blood samples for laboratory testing, including PK samples.																							
**Participant Education and Assessment**																								
Lifestyle Program instruction			X	X	X	X			X			X			X			X			X		X	
	Counselling on diet and exercise, to be performed by a dietitian or equivalent qualified delegate, to include calculation of individualized energy requirement and methods to change dietary composition and amount of physical activity. The Lifestyle Program instruction may be delivered on a separate day from the rest of that visit's study procedures but must occur within the visit window. Beginning at Week 8, the consultation may be delivered by phone.																							
Review diet and exercise goals			X	X	X	X	X	X	X	X	X	X	X	X	X	X	X	X	X	X	X		X	
	All training should be repeated as needed to ensure participant compliance.<br>Study personnel to provide reinforcement and encouragement for lifestyle modifications.																							
Diabetes education (includes BG meter dispensing and SMBG training)		X																						

Visit	1	2	3	4	5	6	7	8	9	10	11	12	13	14	15	16	17	18	19	20	21	99	ED	801
**Week of Treatment**	-3	-2	0	4	8	12	16	20	24	28	32	36	40	44	48	52	56	60	64	68	72	72		4 wks post end of TxP
**Allowable Interval Tolerance (days)**		±3	±7	±3	+7	±3	+7	±3	±3	±3	±3	+7	±3	±3	±3	±3	±3	±3	±3	±3	±7	±7		±3
**Fasting Visit**	X		X	X	X	X	X	X	X			X			X			X			X	X	X	X
**Telephone Visit**										X	X		X	X		X	X		X	X				
	Diabetes education to be performed by personnel who are qualified to educate participants on symptoms and management of hyperglycemia and hypoglycemia, SMBG, self-injection, and diabetes management. Participants are required to measure fasting SMBG values weekly and record them in the study diary. It is recommended that participants measure their fasting glucose on the same day each week. All trainings should be repeated as needed to ensure participant compliance.																							
Dispense BG meter/supplies as needed		X	X	X	X	X	X	X	X			X			X			X			X		X	
Remind participants about 7-point SMBG		X						X												X				
	Participant is required to collect two 7-point SMBGs on nonconsecutive days within 2 weeks prior to the next visit. A 7-point SMBG consists of measurements before and 2 hours after each of 3 main meals within the same day and at bedtime.																							
Injection training with autoinjector demonstration device			X																					
**Participant Diary (paper)**																								
Dispense diary, instruct in use		X	X	X	X	X	X	X	X			X			X			X			X		X	
	All training should be repeated as needed to encourage participant compliance.																							
Diary review, including hypoglycemia			X	X	X	X	X	X	X	X	X	X	X	X	X	X	X	X	X	X	X		X	X
Review 7-point SMBG values collected in the diary			X						X												X			
**Patient Reported Outcomes**																								
PGIs	X		X																		X		X	
SF-36v2 acute form	X		X																		X		X	

Visit	1	2	3	4	5	6	7	8	9	10	11	12	13	14	15	16	17	18	19	20	21	99	ED	801
**Week of Treatment**	-3	-2	0	4	8	12	16	20	24	28	32	36	40	44	48	52	56	60	64	68	72	72		4 wks post end of TxP
**Allowable Interval Tolerance (days)**		±3	±7	±3	+7	±3	+7	±3	±3	±3	±3	+7	±3	±3	±3	±3	±3	±3	±3	±3	±7	±7		±3
**Fasting Visit**	X		X	X	X	X	X	X	X			X			X			X			X	X	X	X
**Telephone Visit**										X	X		X	X		X	X		X	X				
IWQOL-Lite-CT	X		X																		X		X	
EQ-5D-5L			X																		X		X	
**Mental Health Questionnaires**																								
Patient Health Questionnaire-9 (PHQ-9)	X		X			X			X			X			X			X			X	X	X	X
	PHQ-9 is self-administered and should be completed *__after__* assessment of adverse events.																							
Columbia-Suicide Severity Rating Scale (C-SSRS) (Baseline/Screening Version)	X																							
	The C-SSRS should be administered *__after__* assessment of adverse events. For this study, the C-SSRS is adapted for the assessment of the ideation and behavior categories only. The Intensity of Ideation and Lethality of Behavior sections are removed.																							
Columbia-Suicide Severity Rating Scale (C-SSRS) (Since Last Visit Version)		X	X	X	X	X	X	X	X	X	X	X	X	X	X	X	X	X	X	X	X	X	X	X
	The C-SSRS should be administered *__after__* assessment of adverse events and capture information since most recent C-SSRS administration. For this study, the C-SSRS is adapted for the assessment of the ideation and behavior categories only. The Intensity of Ideation and Lethality of Behavior sections are removed.																							
Self-Harm Supplement Form	X	X	X	X	X	X	X	X	X	X	X	X	X	X	X	X	X	X	X	X	X	X	X	X
	The Self-Harm Supplement Form should be administered *__after__* assessment of adverse events.																							
Self-Harm Follow-up Form	X	X	X	X	X	X	X	X	X	X	X	X	X	X	X	X	X	X	X	X	X	X	X	X
	Self-Harm Follow-up Form is only required if triggered by the Self-Harm Supplement Form, per instructions in the form.																							
**Laboratory Tests and Sample Collections**																								
Hematology	X					X			X												X		X	X
HbA1c	X		X			X			X			X			X			X			X		X	X

Visit	1	2	3	4	5	6	7	8	9	10	11	12	13	14	15	16	17	18	19	20	21	99	ED	801
**Week of Treatment**	-3	-2	0	4	8	12	16	20	24	28	32	36	40	44	48	52	56	60	64	68	72	72		4 wks post end of TxP
**Allowable Interval Tolerance (days)**		±3	±7	±3	+7	±3	+7	±3	±3	±3	±3	+7	±3	±3	±3	±3	±3	±3	±3	±3	±7	±7		±3
**Fasting Visit**	X		X	X	X	X	X	X	X			X			X			X			X	X	X	X
**Telephone Visit**										X	X		X	X		X	X		X	X				
Chemistry panel (includes Creatinine for eGFR calculation and glucose)	X		X			X			X			X			X			X			X		X	X
	The CKD-EPI equation will be used by the central lab to estimate and report eGFR.																							
Lipid panel	X		X						X												X		X	X
Insulin			X						X												X		X	X
C-peptide			X						X												X		X	X
Free fatty acids	X		X						X												X		X	X
Serum pregnancy	X																							
	For women of childbearing potential only																							
Urine pregnancy (local)			X			X			X			X			X			X			X		X	
	A urine pregnancy test must be performed at Visit 3 with the result available prior to first injection of study drug(s) for women of childbearing potential only. Additional pregnancy tests (beyond those required per the SoA) should be performed at any time during the trial if a menstrual period is missed, there is clinical suspicion of pregnancy, or as required by local law or regulation.																							
Follicle-stimulating hormone (FSH)	X																							
	Only for postmenopausal women at least 40 years of age with an intact uterus, not on hormone therapy, and who have had spontaneous amenorrhea for more than 1 year without an alternative medical cause.																							
Calcitonin	X					X			X												X		X	X
Pancreatic amylase	X		X			X			X												X		X	X
Lipase	X		X			X			X												X		X	X
Urinary albumin/creatinine ratio	X								X												X		X	X
Cystatin-c	X								X												X		X	X
Thyroid-stimulating hormone (TSH)	X																							
Immunogenicity (includes TZP PK sample)			X	X		X			X						X						X		X	X
	In the event of systemic drug hypersensitivity reactions (immediate or nonimmediate), additional unscheduled samples should be collected as detailed in Section 8.3.2.7 (Hypersensitivity Reactions). Immunogenicity samples and PK samples for immunogenicity must be taken prior to drug administration.																							

Visit	1	2	3	4	5	6	7	8	9	10	11	12	13	14	15	16	17	18	19	20	21	99	ED	801
**Week of Treatment**	-3	-2	0	4	8	12	16	20	24	28	32	36	40	44	48	52	56	60	64	68	72	72		4 wks post end of TxP
**Allowable Interval Tolerance (days)**		±3	±7	±3	+7	±3	+7	±3	±3	±3	±3	+7	±3	±3	±3	±3	±3	±3	±3	±3	±7	±7		±3
**Fasting Visit**	X		X	X	X	X	X	X	X			X			X			X			X	X	X	X
**Telephone Visit**										X	X		X	X		X	X		X	X				
TZP PK					X		X					X												
	PK samples will be collected from a subset of participants at these visits at time windows of 1 to 24 hours, 24 to 96 hours, OR 120 to 168 hours post dose, as assigned by IWRS. Depending on the time-windows to which a participant gets assigned, they may be required to come to site for PK-specific visits.																							
**Stored Samples**																								
Pharmacogenetic sample			X																					
Nonpharmacogenetic sample			X			X			X						X						X		X	X
**Randomization and Dosing**																								
Randomization			X																					
Dispense study drug			X	X	X	X	X	X	X			X			X			X						
Observe participant administer study drug			X																					
	Participants should administer their first dose of study drug at the end of the Visit 3, after other study procedures and randomization have been completed.																							
Participant returns study interventions and injection supplies				X	X	X	X	X	X			X			X			X			X		X	
Assess study intervention(s) compliance				X	X	X	X	X	X	X	X	X	X	X	X	X	X	X	X	X	X		X	

Abbreviations: BG = blood glucose; CKD-EPI = Chronic Kidney Disease-Epidemiology; ECG = electrocardiogram; eCRF= electronic case report form; ED = early treatment discontinuation; eGFR = estimated glomerular filtration rate; HbA1c = hemoglobin A1c; HR=heart rate; IWQOL-Lite-CT = Impact of Weight on Quality of Life-Lite-Clinical Trials Version; IWRS = interactive web-response system; PGIs = Patient Global Impression of status for physical activity; PK = pharmacokinetics; SF-36v2 acute form = Short Form-36 Version 2 Health Survey acute form; SMBG = self-monitoring of blood glucose; SoA = Schedule of Activities; TxP = treatment period; TZP= tirzepatide; wks = weeks.

Note:

- Visit 3 baseline assessments must be completed before processing the randomization in the IWRS.
- The visit date is determined in relation to the date of Visit 3 (randomization).

- Visit 99 is only applicable to participants who discontinue the double-blind study treatment prematurely and decline to complete the remaining scheduled study visits. Participants wanting to discontinue the study before Week 72 will be asked to return for Visit 99, 72 weeks ± 7 days after randomization, primarily for body weight measurement and assessment of adverse events. If the participant is unwilling to attend Visit 99, it should be documented in the participant medical record that the participant has refused to attend.
- Participants who are unable or unwilling to continue the study treatment for any reason will perform an ED visit. If the participant is discontinuing during an unscheduled visit or a scheduled visit, that visit should be performed as an ED visit.
- Visit 801 (safety follow-up visit) should be performed 4 weeks after the last visit of the participant’s treatment period (see Section 7.2.1.).
- For all office visits (except for Visit 2), remind participants to report to the site before taking study drug(s) in a fasting condition, after a period of approximately 8 hours without eating, drinking (except water), or any significant physical activity. Since some screening procedures need to be completed in the fasting state, Visit 1 may be conducted over more than 1 day to ensure necessary conditions are met. The participant should not take any antihyperglycemic medications prior to the fasting visit.

2. Introduction

Obesity is a chronic disease whose increasing prevalence is a public health concern associated with rising incidence of type 2 diabetes mellitus (T2DM), increased risk for premature death, and increased risk for some cancers (AMA 2013; Council on Science and Public Health 2013; Lauby-Secretan et al. 2016).

For patients with T2DM who have obesity or are overweight, efforts targeting weight loss, including lifestyle, medication, and surgical interventions, are recommended (Davies 2018) because there is strong and consistent evidence that obesity management is beneficial in the treatment of type 2 diabetes and weight-related comorbidities (ADA 2020). Lifestyle changes that result in modest and sustained weight loss produce clinically meaningful reductions in blood glucose (BG), hemoglobin A1c (HbA1c), and triglycerides. Greater weight loss produces even greater metabolic benefits, including reductions in blood pressure, improvements in low-density lipoprotein (LDL) and high-density lipoprotein (HDL) cholesterol, and reductions in the need for medications to control BG, blood pressure, and lipids, and may even result in achievement of glycemic goals in the absence of glucose-lowering agent use in some patients (ADA 2020; UKPDS Group 1990; Pastors et al. 2002; Wing et al. 2011; Rothberg et al. 2017).

However, therapeutic weight loss in patients with T2DM has been very difficult to achieve (Pi-Sunyer et al. 2005). Lifestyle therapies fail to achieve sustainable weight loss in the majority of patients with obesity (Dombrowski et al. 2014). Currently, only a limited number of prescription drugs are approved by the Food and Drug Administration (FDA) and other regulatory agencies for chronic weight management in adults. They have rather modest efficacy (Müller et al. 2018) and their central nervous system, gastrointestinal (GI), and cardiovascular side effects (Srivastava and Apovian 2018a, 2018b) limit their use for many people with obesity. Patients with T2DM who have obesity or who are overweight would benefit from greater availability of pharmacotherapeutic agents that are effective in lowering body weight.

2.1. Study Rationale

The incretin hormones, glucose-dependent insulinotropic polypeptide (GIP) and glucagon-like peptide-1 (GLP-1), are secreted after meal ingestion and mediate the incretin effect. Both hormones have effects on endocrine cells in the pancreas, increasing insulin biosynthesis and secretion, and modifying glucagon secretion (Skow et al. 2016). Based on these properties, several GLP-1 receptor (GLP-1R) agonists have been approved for pharmacological treatment of T2DM (Tomlinson et al. 2016).

In addition to its pancreatic effects, GLP-1R activation decreases gut motility, slows gastric emptying, and promotes satiety (presumably through a combination of GLP-1R activation in the central and peripheral nervous systems), thereby regulating food intake and body weight (Baggio and Drucker 2007). The US FDA and the European Medicines Agency (EMA) approved the GLP-1R agonist liraglutide for the treatment of overweight and obesity (SAXENDA package insert, 2014; SAXENDA summary of product characteristics 2015).

Preclinical data indicate that GIP also exerts effects on appetite regulation and food intake, on adipose tissue, and on peripheral energy metabolism. Although studies evaluating effects of GIP

on body weight have yielded equivocal results, GIP receptor (GIPR) activation may play a role in body weight regulation and targeting both the GLP-1R and the GIPR simultaneously could potentially result in additive or synergistic effects of the 2 incretins on body weight (Coskun et al. 2018).

Tirzepatide is a 39-amino acid synthetic peptide with agonist activity at both the GIPR and GLP-1R. Its structure is based on the GIP sequence and includes a C20 fatty diacid moiety (Coskun et al. 2018). It is administered once-weekly (QW) by subcutaneous (SC) injection. As a dual GIP/GLP-1R agonist, tirzepatide could exceed the efficacy of selective GLP-1R agonists by recruiting metabolically active tissues not targeted by selective GLP-1R agonists (for example, adipose tissue as indicated by the observation of increased energy utilization) (Müller et al. 2018) and has the potential to reach higher efficacy in target tissues that express both GIPR and GLP-1R. Therefore, tirzepatide has the potential to impact several aspects of energy regulation and be a treatment for overweight and obesity.

Compared to people without diabetes, patients with T2DM and obesity or overweight often respond less favorably to weight-management treatments (Pi-Sunyer, 2005) and may face unique safety issues such as risk for hypoglycemia following weight loss, if the dose of certain background medications for the management of hyperglycemia is not appropriately adjusted. Therefore, it is important to conduct a dedicated phase 3, randomized, double-blind, placebo-controlled study in patients with T2DM and obesity or overweight to evaluate the efficacy and safety of novel chronic weight management products. In addition, weight loss should be demonstrated over the course of at least 1 year before a product can be considered as effective for weight management (FDA 2007).

Study 18F-MC-GPHL is a Phase 3, multicenter, randomized, parallel, placebo-controlled, double-blinded study that will investigate the effects of treatment with tirzepatide 10 mg and 15 mg QW compared with placebo, in conjunction with a reduced-calorie diet and increased physical activity, on weight management at 72 weeks in participants with T2DM who have obesity or are overweight.

2.2. Background

There remains an unmet need in the pharmacologic treatment of obesity for drugs that are safe, efficacious, and well-tolerated. There are currently only a few medications FDA-approved for long-term use for the treatment of obesity that yield a placebo-adjusted average weight loss between 3% and 7% (Srivastava and Apovian 2018b, FDA 2020). Although moderate weight loss of 5% to 10% in individuals with obesity/overweight has long been shown to yield significant metabolic benefits including improvements in cholesterol, blood pressure, and glucose parameters (Goldstein 1992; Wing et al. 2011), greater weight loss can maximize these benefits and may be required to realize clinically meaningful improvements in other obesity-related comorbidities such as sleep apnea, nonalcoholic steatohepatitis, and cardiovascular disease (Ryan et al. 2017). In addition to moderate efficacy, some centrally-acting weight loss agents to-date have had adverse neurocognitive, psychiatric, or cardiovascular effects, further limiting their application in clinical practice (Srivastava and Apovian 2018b).

Weight loss induced by GLP-1R agonists, while appearing to be centrally-mediated through a combination of hormonal inputs to satiety centers (van Bloemendaal et al. 2014), has not been consistently associated with changes in mental health or with potential for addiction in long-term

studies conducted to establish cardiovascular safety in patients with diabetes (Marso et al. 2016a, 2016b; Gerstein et al. 2019). Tirzepatide, which is both a GLP-1R and GIPR agonist, has been associated with predominantly mild to moderate GI adverse effects similar to currently marketed GLP-1R agonists, but has also demonstrated significant weight loss at both the 10 mg and 15 mg doses in Phase 2 studies with nearly half of the participants in both dose arms achieving ≥10% weight loss (Frias et al. 2018).

Dose selection for obesity treatment has been informed by 3 clinical trials: a Phase 1 study, Study I8F-MC-GPGA (GPGA), and 2 Phase 2 studies, Study I8F-MC-GPGB (GPGB) and I8F-MC-GPGF (GPGF).

Phase 1 Study GPGA was a combination of single ascending dose (SAD) and multiple ascending dose (MAD) study in healthy subjects followed by a multiple dose study in patients with T2DM. A total of 142 participants (89 healthy subjects and 53 patients with T2DM) received at least 1 dose of treatment. Doses of tirzepatide ranged from:

- 0.25 mg to 8 mg in the SAD (with maximum tolerated dose achieved at 5 mg) in healthy subjects,
- multiple doses in the MAD from 0.5 mg to 4.5 mg QW and titrated doses up to 10 mg QW for 4 weeks in healthy subjects, and
- multiple doses at 0.5 mg and 5 mg QW and titrated to 15 mg QW for 4 weeks in patients with T2DM.

The safety and tolerability and pharmacokinetic/pharmacodynamic (PK/PD) profiles of tirzepatide at doses and escalation regimens administered in this Phase 1 study supported further development of tirzepatide for QW dosing in patients with T2DM.

Phase 2 studies I8F-MC-GPGB (GPGB) and I8F-MC-GPGF (GPGF) provided initial safety, tolerability, and efficacy data in the tirzepatide 1 to 15 mg dose-range when used in treatment of patients with T2DM. In the dose range of 5 to 15 mg, tirzepatide provided significantly greater reductions in HbA1c and body weight compared with placebo. In addition, 10-mg and 15-mg dose arms demonstrated significantly greater weight loss compared to the GLP-1R agonist dulaglutide 1.5 mg QW. The most common adverse events (AEs), which were also dose dependent, were mild to moderate nausea, vomiting, and diarrhea. Study GPGF showed that adjustments in the tirzepatide dose escalation algorithm resulted in additional reductions in the frequency of GI AEs and reduced the frequency of treatment discontinuations due to GI AEs.

2.3. Benefit/Risk Assessment

More detailed information about the known and expected benefits and risks and reasonably expected AEs of tirzepatide may be found in the Investigator's Brochure (IB).

3. Objectives and Endpoints

Objectives	Endpoints
**Primary**	
To demonstrate that tirzepatide 10 mg QW is superior to placebo at 72 weeks for:<br>• Percent change in body weight AND<br>• Proportion of participants with ≥5% body weight reduction<br><br>AND/OR<br><br>To demonstrate that tirzepatide 15 mg QW is superior to placebo at 72 weeks for:<br>• Percent change in body weight AND<br>• Proportion of participants with ≥5% body weight reduction	• Mean percent change in body weight from randomization<br>• Percentage of study participants who achieve ≥5% body weight reduction from randomization
**Key Secondary (controlled for type I error), by dose analysis**	
For QW tirzepatide 10 mg and/or 15 mg doses, to demonstrate superiority to placebo in change from randomization for the following (measured at 72 weeks):<br>• Body weight	• Percentage of participants who achieve:<br>○ ≥10% body weight reduction<br>○ ≥15% body weight reduction
• Glycemic control	• Mean change in HbA1c (%)<br>• Percentage of participants who achieve HbA1c <7%<br>• Mean change in fasting glucose (mg/dL)
• Waist circumference	• Mean change in waist circumference (cm)
**Key secondary (controlled for type I error), pooled dose analysis**	

For QW tirzepatide (all doses combined) to demonstrate superiority to placebo in change from randomization for the following (measured at 72 weeks):<br><br>• Lipid parameters<br><br>• SBP	• Mean change in fasting<br>o Total cholesterol (mg/dL)<br>o LDL-cholesterol (mg/dL)<br>o Triglycerides (mg/dL)<br><br>• Mean change in SBP (mmHg)
**Additional Secondary, by dose analysis**	
For QW tirzepatide 10 mg and/or 15 mg doses, to demonstrate superiority to placebo in change from randomization for the following (measured at 72 weeks):<br>• Body weight<br><br>• Glycemic control<br><br>• Insulin<br><br>• Patient-Reported Outcomes	• Mean change in absolute body weight (kg)<br>• Mean change in BMI (kg/m^2)<br><br>• Percentage of study participants who achieve HbA1c ≤6.5%<br>• Percentage of study participants who achieve HbA1c <5.7%<br><br>• Mean change in fasting insulin (pmol/L)<br><br>• Mean change in<br>o SF-36v2 acute form Physical Functioning domain score<br>o IWQOL-Lite-CT Physical Function composite score
**Additional Secondary, pooled analysis**	

For QW tirzepatide (all doses combined), to demonstrate superiority to placebo in change from randomization for the following (measured at 72 weeks):<br>• Lipid parameters<br><br><br><br>• DBP	• Mean change in fasting<br>o HDL-cholesterol (mg/dL)<br>o VLDL-cholesterol (mg/dL)<br>o Free fatty acids (mg/dL)<br><br>• Mean change in DBP (mmHg)
**Pharmacokinetics/Pharmacodynamics**<br>• To characterize the population PK of tirzepatide and explore the relationships between the tirzepatide concentration and efficacy, safety, and tolerability measures	• Population PK and PD parameters

Abbreviations: BMI = body mass index; DBP = diastolic blood pressure; HbA1c = hemoglobin A1c; HDL = high-density lipoprotein; IWQOL-Lite-CT = Impact of Weight on Quality of Life-Lite-Clinical Trials Version; LDL = low-density lipoprotein; PD = pharmacodynamics; PK = pharmacokinetics; QW = once weekly; SBP = systolic blood pressure; SF-36v2 acute form = Short Form-36 version 2 Health Survey acute form; VLDL = very low-density lipoprotein.

4. Study Design

4.1. Overall Design

Study 18F-MC-GPHL is a Phase 3, multicenter, randomized, parallel, placebo-controlled, double-blinded, 72-week study that will investigate the safety and efficacy of treatment with tirzepatide 10 mg and 15 mg QW SC compared with placebo QW SC, in conjunction with a reduced-calorie diet and increased physical activity, on weight management in participants with T2DM who have obesity (body mass index [BMI] ≥30 kg/m^2) or are overweight (BMI ≥27 kg/m^2).

Study participants will be randomized in a 1:1:1 ratio (tirzepatide 10 mg QW, tirzepatide 15 mg QW, and placebo). An upper limit of 70% enrollment of women will be used to ensure a sufficiently large sample of men. In addition, an upper limit of 30% enrollment of participants treated with SU will be used to allow sufficient enrollment of participants treated with other antihyperglycemic medications (AHMs).

All participants will undergo a 3-week screening period, a 72-week treatment period (including a 20-week dose escalation and a 52-week dose maintenance period), and a 4-week safety follow-up period.

4.1.1. Overview of Study Periods

4.1.1.1. Visit Structure for all Office Visits

On all office visits (other than Visit 2), study participants are required to report to the site in a fasting condition, after a period of approximately 8 hours without eating, drinking (except water), or performing any significant physical activity and before taking study drug(s). The participant should not take any AHMs prior to the visit. If participants are adversely affected by the fasting condition, they are allowed to eat; however, specific study procedures need to be completed while fasting. See Section 10.8 Appendix 8 for a suggested order of activities that occurs at office visits.

4.1.1.2. Main Study Period

4.1.1.2.1. Screening Period

Visit 1

The purpose of screening procedures at Visit 1 is to establish initial eligibility and to obtain blood samples for laboratory assessments needed to confirm eligibility. The participant must sign the informed consent form (ICF) before the study procedures are performed, as outlined in the Schedule of Activities (SoA), Section 1.3. Since some screening procedures need to be completed in the fasting state (approximately 8 hours without eating, drinking [except water], or any significant physical activity), Visit 1 may be conducted over more than 1 day to ensure necessary conditions are met.

Patient-reported outcomes (PROs) questionnaires should be administered as early as possible, as per Suggested Visit Structure (Section 10.8). Preferred recommended administration order is:

1. Patient Global Impression of status for physical activity (PGIs)
2. Short Form-36 version 2 Health Survey acute form (SF-36v2 acute form)
3. Impact of Weight on Quality of Life-Lite-Clinical Trials Version (IWQOL-Lite-CT)

Participants who meet all applicable inclusion criteria and none of the applicable exclusion criteria at Visit 1 will continue to Visit 2.

Visit 2

At Visit 2, the screening laboratory results will be reviewed. For all participants who remain eligible after Visit 1, a dilated fundoscopic examination will be performed by an eye care professional (ophthalmologist or optometrist) between Visit 2 and Visit 3 to exclude participants with proliferative diabetic retinopathy, diabetic macular edema, or nonproliferative diabetic retinopathy that requires acute treatment.

Participants will be provided diaries and be trained to record key study information, as appropriate.

Diabetes education will be performed by personnel who are qualified to educate participants on symptoms and management of hyperglycemia and hypoglycemia, self-monitored BG, self-injection and diabetes management according to American Diabetes Association Standards of Medical Care in Diabetes (ADA 2020) or local standards. Participants will be trained on how to utilize BG meters and how to collect self-monitoring of blood glucose (SMBG), including 7-point measurements as appropriate. Blood glucose meters/supplies will be provided to measure SMBG values and participants will be required to record them in the study diary.

4.1.1.2.2. Treatment Period

Randomization (Visit 3)

At Visit 3, eligible participants (who meet all applicable inclusion criteria and none of the applicable exclusion criteria) will perform all required randomization study procedures (including the collection of all baseline laboratory measures, electrocardiograms (ECGs), and questionnaires) prior to randomization and prior to taking the first dose of study drug.

Participants should arrive at the clinic in the fasting state (after a period of approximately 8 hours without eating, drinking [except water], or any significant physical activity and before taking AHMs). Patient-reported outcomes questionnaires should be administered as early as possible in the visit, as per Suggested Visit Structure (Section 10.8). Preferred administration order:

1. PGIs
2. SF-36v2 acute form
3. IWQOL-Lite-CT
4. EQ-5D-5L

The mental health questionnaires (Patient Health Questionnaire-9 [PHQ-9], Columbia-Suicide Severity Rating Scale [C-SSRS], and Self-Harm Form) should be completed after the assessment for AEs.

Participants will receive an initial consultation with a dietician, or equivalent qualified delegate, according to local standards. Lifestyle goals for caloric intake and physical activity will be set (Section 5.3).

Following randomization, study site personnel will demonstrate use of the autoinjector (also referred to as single-dose pen) using the provided demonstration device and observe the study participant inject the first dose of tirzepatide or placebo. The date, time, and location of the first dose of study drug will be recorded on the electronic case report form (eCRF). Beginning at randomization, all participants will receive study drug according to the randomized treatment arm for the duration of the 72-week treatment period including a dose escalation period of up to 20 weeks.

Treatment Period (End of Visit 3 to Visit 21)

During the treatment period, office visits will occur monthly through 24 weeks and then every 12 weeks thereafter, to 72 weeks. The participant should be fasting for all office visits. Telephone visits will occur at 4-week intervals between the office visits starting at Week 28.

Office visit procedures should be conducted according to the SoA (Section 1.3), and will include:

- weight, waist circumference, and vital signs measurements
- laboratory testing
- administration of PROs questionnaires
- collection of AEs, product complaints (PCs), and concomitant medications
- review of SMBG values and hypoglycemic events collected in the diary
- Mental Health questionnaires
- review of participant diary information (to include reinforcement and compliance assessments for study drug administration and lifestyle goals)
- study drug and supplies dispensing

Patient-reported outcomes questionnaires should be administered as early as possible in the visit, as per Suggested Visit Structure (Section 10.8). Preferred administration order is the same as Randomization visit.

Mental Health questionnaires should be completed after the assessment of AEs.

Lifestyle consultations continue at Weeks 4, 8, 12, and then every 12 weeks thereafter, to Week 72. Study drug and injection supplies will be returned per the SoA (Section 1.3) and according to local requirements. New supplies will be dispensed as needed.

Study drug dose escalation is double-blinded and will be managed via the interactive web-response system (IWRS). The starting dose is 2.5 mg QW (or matching placebo) for 4 weeks, then the dose is increased by 2.5 mg (or matching placebo) every 4 weeks (2.5 to 5 to 7.5 to 10 mg) for those randomized to 10 mg, continuing to 12.5 to 15 mg for those randomized to 15 mg. The dose is then maintained for the remainder of the study. In participants who experience intolerable GI symptoms, the dose can be changed as described in the Section 6.6.2.

At each of the 8 scheduled telephone visits, procedures will include:

- collection of AEs, PCs, and concomitant medications
- review of SMBG values and hypoglycemic events collected in the diary

- administration of Mental Health questionnaires
- review of participant diary information (to include reinforcement and compliance assessments for study drug administration and lifestyle goals)

Participants should be instructed to contact the investigative site for assistance as soon as possible if they experience any difficulties administering their study medication. Participants should also be advised about the appropriate course of action in the event that study drug is not taken at the required time (late/missing doses).

Study participants will be permitted to use concomitant medications that they require during the study, except certain excluded medications (see Section 5.2) that may interfere with the assessment of efficacy and safety characteristics of the study treatments.

Visit 99

Visit 99 is only applicable to participants who discontinue the study treatment prematurely (before Week 72) and decline to complete the remaining scheduled study visits. These participants will be asked to return for Visit 99 at 72 weeks ± 7 days after randomization (See Section 7.2.1.). This visit is critical to ensure complete data collection for the primary weight loss endpoint.

Participants should attend this visit in the fasting state. Procedures to be completed are:

- measurement of weight and waist circumference
- listing of concomitant medications
- assessment of AEs, and
- completion of the Mental Health questionnaires (after the AE assessment).

For participants unwilling to attend this visit, their refusal to attend should be documented in the participant medical record.

4.1.1.2.3. Early Discontinuation of Treatment Visit

Participants unable or unwilling to continue the study treatment for any reason will perform an early discontinuation (ED) of treatment visit (Section 7.1). If the participant is discontinuing during an unscheduled visit or a scheduled visit, that visit should be performed as an ED visit. Procedures should be completed according to the SoA. Patient-reported outcomes questionnaires should be administered as early as possible, as per Suggested Visit Structure (Section 10.8). Administration of Mental Health questionnaires should follow assessment for AEs.

4.1.1.3. Safety Follow-up Period

Visit 801

All participants are required to complete a safety follow-up visit (Visit 801), according to the SoA. Participants discontinuing the study early and performing an ED visit will also be asked to perform the safety follow-up visit. During the safety follow-up period, participants will not receive study drug. Participants may be treated with another glucose-lowering intervention at the discretion of the investigator or participant’s usual diabetes care physician (see Section 6.5.2). If any new AHM is initiated during the safety follow-up period, it will not be classified as rescue

therapy (see Section 6.5.3.1). Participants are also required to return any remaining study diaries to the study site at the end of this period.

For participants who discontinue or complete the study, Visit 801 (safety follow-up visit) should be performed 4 weeks after the last visit of the treatment period (Visit 21 or 99) or 4 weeks after the ED visit for those who decline to return for Visit 99.

4.1.2. Study Procedures

Participants will perform study procedures listed in the SoA.

Participants will be permitted to use concomitant medications that they require during the study, except certain medications that may interfere with the assessment of efficacy and safety characteristics of the study treatments (Section 5.2).

Study governance considerations are described in detail in Section 10.1 (Appendix 1).

4.2. Scientific Rationale for Study Design

Tirzepatide is a 39-amino acid synthetic peptide with agonist activity at both the GIPR and GLP-1R. Its structure is based on the GIP sequence and includes a C20 fatty di-acid moiety (Coskun et al. 2018). It is administered QW by SC injection.

As a dual GIP/GLP-1R agonist, tirzepatide could exceed the efficacy of selective GLP-1R analogs by recruiting metabolically active tissues not targeted by selective GLP-1R analogs (for example, adipose tissue as indicated by the observation of increased energy utilization) (Müller et al. 2018) and has the potential to reach higher efficacy in target tissues, such as insulin-producing pancreatic beta-cells that express both GIPR and GLP-1R, before reaching its therapeutic limitation. Results from a Phase 2 study (GPGB) demonstrated that tirzepatide use in participants with T2DM was associated with a substantial, dose-dependent weight loss, greater than the weight change observed with dulaglutide, a specific GLP-1R agonist. General safety characteristics of all studied doses of tirzepatide were similar to that of the GLP-1R agonist class, consisting mainly of nausea, vomiting, and diarrhea. In general, these events were transient and mild or moderate in severity, with few severe episodes. This suggests that tirzepatide has the potential to become a medication for chronic weight management.

Although GI AEs were more common in the 15-mg arm of tirzepatide in the Phase 2 study (GPGB), this dose demonstrated the highest efficacy in terms of weight loss. An optimized dose escalation regimen proposed in the Study GPHL to improve tolerability (and supported by a dose escalation algorithm from study GPGF and PK/PD modelling) should enable a use of higher doses to maximize effects on body weight.

Study GPHL is designed to determine the comparative benefits and risks of tirzepatide 10 mg and 15 mg QW versus placebo in participants with T2DM who have obesity or are overweight. A double-blind design was selected to minimize participant and investigator bias in assessments for efficacy, safety, and study drug tolerability.

A placebo comparator was selected for this trial in accordance with regulatory guidance (FDA 2007; EMA 2016). In addition, all participants, regardless of treatment assignment, will receive lifestyle modification counselling and diabetes education consistent with current guidelines for weight management (Jensen et al. 2014) and T2DM management (ADA 2020). Specifically,

participants will consult with a dietician, or equivalent qualified delegate, throughout the study to achieve approximately a 500 kcal/day energy deficit through a combination of caloric restriction and increased physical activity.

The planned duration of treatment for the primary endpoint at 72 weeks allows for a 52-week treatment period at the dose achieved following dose escalation to either 10 or 15 mg. This duration is considered appropriate to assess the full effects and benefit/risk of each maintenance dose of tirzepatide compared with placebo on body weight and is consistent with regulatory guidelines (FDA 2007; EMA 2016).

The effects of study drug cessation will be assessed in the 4-week safety follow-up/observational period. To minimize the potential confounding effect of changes to concomitant medications, participants will be permitted to use concomitant medications that do not interfere with the assessment of efficacy or safety characteristics of the study treatments.

4.3. Justification for Dose

Dose selection for the tirzepatide program focused on addressing the unmet need of achieving greater than 10% weight loss (Srivastava and Apovian 2018a; Ganguly et al. 2018) in order to provide greater metabolic and functional benefits to people with obesity.

Tirzepatide doses of 10 mg and 15 mg QW will be evaluated in this study. These doses and associated escalation schemes were selected based on assessment of safety, efficacy (weight loss), and GI tolerability data in Phase 1 and 2 studies with T2DM, followed by exposure response modeling of the data that predicted weight loss in patients with overweight or obesity.

In a 26-week, Phase 2 study (GPGB) of participants with T2DM, tirzepatide reduced body weight by 4.8 kg, 8.7 kg, and 11.3 kg at dose levels of 5, 10, and 15 mg, respectively, whereas the weight loss observed in participants on dulaglutide, a selective GLP-1R agonist, used at the dose of 1.5 mg, was 2.7 kg (Frias et al. 2018).

Similar to the GLP-1R agonist class, most of tirzepatide AEs were dose dependent and GI-related, consisting mainly of nausea, vomiting, and diarrhea. In general, these events were mild or moderate in severity, with few severe episodes, and transient.

Tirzepatide doses of 10 mg and 15 mg were selected based principally on the following criteria:

- each dose provides robust weight loss relative to placebo,
- the percent of patients achieving >10% is higher with 15 mg than 10 mg,
- both doses are expected to address the unmet need of achieving greater than 10% weight loss, and
- safety and tolerability of 10 mg and 15 mg were supported by Phase 2 results and/or PK/PD modeling.

Tirzepatide dose of 5 mg is not selected as a maintenance dose since it is unlikely to help address the unmet need of achieving greater than 10% weight loss. This is further supported by the following data from the T2DM Phase 2 Study GPGB:

- only 16.7% of participants on 5 mg achieved ≥10% weight loss compared to 45.5% and 54.3% of participants on 10 mg and 15 mg, respectively, in the 26-week on treatment analysis

- patients treated with tirzepatide 5 mg demonstrated a placebo-corrected mean body weight loss of 4.4 kg and mean percentage weight loss of 4.7%
    - among the 4 currently FDA-approved medications for chronic weight management, placebo-corrected mean body weight loss and mean percentage weight loss from baseline range from 2.9 to 8.6 kg and from 2.9% to 8.6%, respectively (Apovian et al. 2015)
    - although there were no head-to-head clinical trials comparing tirzepatide with the current FDA-approved medications for chronic weight management, based on data from GPGB, it is unlikely that tirzepatide 5 mg will demonstrate significant benefit over existing treatment options and address the unmet need of achieving greater than 10% weight loss to provide greater metabolic and functional benefits to people with obesity

To address the decreased tolerability and increased treatment discontinuation in the 15-mg arm observed in GPGB, Lilly conducted the Phase 2 study GPGF to investigate the impact of different starting doses and escalation regimens on tolerability. Slower dose escalation and smaller dose increments are anticipated to improve tolerability. Dosing algorithms starting at a low dose of 2.5 mg accompanied by dose escalation of 2.5 mg increments every 4 weeks should permit time for development of tolerance to GI events and are predicted to minimize GI tolerability concerns. The maximum proposed dose of 15 mg maintains an exposure multiple of 1.6 to 2.4 to the no-observed adverse effect level doses in 6-month monkey and rat toxicology studies.

4.4. End of Study Definition

Section 7.2 describes the criteria used to determine if a participant has completed the study. The end of the study is defined as the date of the last visit of the last participant in the study or last scheduled procedure shown in the SoA for the last participant in the trial globally.

5. Study Population

Prospective approval of protocol deviations to recruitment and enrollment criteria, also known as protocol waivers or exemptions, is not permitted.

5.1. Inclusion Criteria

Participants are eligible to be included in the study only if all of the following criteria apply:

Type of Participant and Disease Characteristics

1. Have a BMI ≥27 kg/m^2
2. Have a diagnosis of T2DM according to the World Health Organization (WHO) classification or other locally applicable standards (see Section 10.10), with HbA1c ≥7% (≥53 mmol/mol) to ≤10% (86 mmol/mol) at screening, on stable therapy for the last 3 months prior to screening. Type 2 diabetes mellitus may be treated with diet/exercise alone or any **oral** glycemic-lowering agent (as per local labeling) EXCEPT dipeptidyl peptidase 4 (DPP-4) inhibitors or GLP-1R agonists
3. Have a history of at least 1 self-reported unsuccessful dietary effort to lose body weight
4. In the investigator's opinion, are well-motivated, capable, and willing to:
   - perform finger stick BG monitoring, including weekly fasting glucose measurements and scheduled BG profiles with up to 7 measurements in 1 day
   - learn how to self-inject study drug, as required for this protocol (visually impaired persons who are not able to perform the injections must have the assistance of a sighted individual trained to inject the study drug; persons with physical limitations who are not able to perform the injections must have the assistance of an individual trained to inject the study drug)
   - inject study drug (or receive an injection from a trained individual if visually impaired or with physical limitations)
   - follow study procedures for the duration of the study, including, but not limited to follow lifestyle advice (for example, dietary restrictions and exercise plan), maintain a study diary, and complete required questionnaires

Participant Characteristics

5. Are at least 18 years of age and age of majority per local laws and regulations
   a. Male participants:
      - Male participants with partners of childbearing potential should be willing to use reliable contraceptive methods throughout the study and

for 5 half-lives of study drug plus 90 days, corresponding to 4 months after the last injection

b. Female participants:
   - Female participants not of childbearing potential may participate and include those who are:
     - infertile due to surgical sterilization (hysterectomy, bilateral oophorectomy, or tubal ligation), congenital anomaly such as Mullerian agenesis; or
     - postmenopausal – defined as either:
       - A woman at least 40 years of age with an intact uterus, not on hormone therapy, who has cessation of menses for at least 1 year without an alternative medical cause, AND a follicle-stimulating hormone (FSH) $\geq$ 40 mIU/mL; women in this category must test negative in pregnancy test prior to study entry

       or

       - A woman 55 or older not on hormone therapy, who has had at least 12 months of spontaneous amenorrhea

       or

       - A woman at least 55 years of age with a diagnosis of menopause prior to starting hormone replacement therapy (HRT)
   - Female participants of childbearing potential (not surgically sterilized and between menarche and 1-year postmenopausal) must:
     - test negative for pregnancy at Visit 1 based on a serum pregnancy test,
     - if sexually active, agree to use 2 forms of effective contraception, where at least 1 form is highly effective for the duration of the trial plus 30 days, corresponding to 2 months after the last injection, and
     - not be breastfeeding

Note: Contraceptive use by men or women should be consistent with local regulations regarding the methods of contraception for those participating in clinical studies.

Informed Consent

6. Capable of giving signed informed consent as described in Appendix 1 which includes compliance with the requirements and restrictions listed in the ICF and in this protocol

5.2. Exclusion Criteria

Participants are excluded from the study if any of the following criteria apply:

Medical Conditions

Diabetes-related

7. Have type 1 diabetes mellitus (T1DM), history of ketosis or hyperosmolar state/coma, or any other types of diabetes except T2DM
8. Have had 1 or more episode of severe hypoglycemia and/or 1 or more episode of hypoglycemia unawareness within the 6 months prior to Visit 1
9. Have at least 2 confirmed fasting SMBG values >270 mg/dL (15.0 mmol/L) (on 2 nonconsecutive days) prior to Visit 3
10. Have history of:
    - proliferative diabetic retinopathy OR
    - diabetic macular edema OR
    - nonproliferative diabetic retinopathy that requires acute treatment.

    Note: A dilated fundoscopic examination performed by an ophthalmologist or optometrist between Visit 2 and Visit 3 is required to confirm eligibility.
11. Current or prior treatment (within 3 months prior to Visit 1) with DPP-4 inhibitors, oral GLP-1R agonist, or **any** injectable therapy for T2DM

Obesity-related

12. Have a self-reported change in body weight >5 kg within 3 months prior to screening
13. Have a prior or planned surgical treatment for obesity (excluding liposuction or abdominoplasty if performed >1 year prior to screening)
14. Have or plan to have endoscopic and/or device-based therapy for obesity or have had device removal within the last 6 months prior to screening
    - mucosal ablation
    - gastric artery embolization
    - intragastric balloon
    - duodenal-jejunal endoluminal liner

Other medical

15. Have renal impairment measured as estimated glomerular filtration rate (eGFR) <30 mL/min/1.73 m^2, calculated by Chronic Kidney Disease-Epidemiology as determined by central laboratory during screening

    Note*:* For participants on metformin therapy, their renal function must be greater than the country-specific threshold criteria for discontinuing metformin therapy per local label.

16. Have a known clinically significant gastric emptying abnormality (for example, severe gastroparesis or gastric outlet obstruction) or chronically take drugs that directly affect GI motility
17. Have a history of chronic or acute pancreatitis
18. Have a thyroid-stimulating hormone (TSH) outside of 0.4 to 6.0 mIU/L at the screening visit

    ***Note**:* Participants receiving treatment for hypothyroidism may be included, provided their thyroid hormone replacement dose has been stable for at least 3 months and their TSH at screening falls within the range indicated above.

    Note: Participants with a history of subclinical hypothyroidism but a TSH at screening within the range indicated above, may be included if, in the investigator's opinion, the participant is unlikely to require initiation of thyroid hormone replacement during the course of the study.
19. Have obesity induced by other endocrinologic disorders (for example Cushing syndrome) or diagnosed monogenetic or syndromic forms of obesity (for example, Melanocortin 4 Receptor deficiency or Prader Willi Syndrome)
20. Have a history of significant active or unstable Major Depressive Disorder (MDD) or other severe psychiatric disorder (for example, schizophrenia, bipolar disorder, or other serious mood or anxiety disorder) within the last 2 years

    Note: Participants with MDD or generalized anxiety disorder whose disease state is considered stable for the past 2 years and expected to remain stable throughout the course of the study, in the opinion of the investigator, may be considered for inclusion if they are not on excluded medications.
21. Have any lifetime history of a suicide attempt
22. Have a PHQ-9 score of 15 or more on or before Visit 3
23. On the C-SSRS at Visits 1, 2, or 3, prior to randomization:
    - a "yes" answer to Question 4 (Active Suicidal Ideation with Some Intent to Act, Without Specific Plan) on the "Suicidal Ideation" portion of the C-SSRS

    or

    - a "yes" answer to Question 5 (Active Suicidal Ideation with Specific Plan and Intent) on the "Suicidal Ideation" portion of the C-SSRS

    or

    - a "yes" answer to any of the suicide-related behaviors (actual attempt, interrupted attempt, aborted attempt, preparatory act or behavior) on the "Suicidal Behavior" portion of the C-SSRS

and

- the ideation or behavior occurred within the past month

24. Have uncontrolled hypertension (systolic blood pressure [SBP] ≥160 mmHg and/or diastolic blood pressure ≥ 100 mmHg)
25. Have any of the following cardiovascular conditions within 3 months prior to randomization: acute myocardial infarction, cerebrovascular accident (stroke), unstable angina, or hospitalization due to congestive heart failure (CHF)
26. Have NYHA Functional Classification class IV CHF
27. Have acute or chronic hepatitis, signs and symptoms of any liver disease other than nonalcoholic fatty liver disease (NAFLD), or any of the following, as determined by the central laboratory during screening:
    - alanine aminotransferase (ALT) level >3.0X the upper limit of normal (ULN) for the reference range,
    - alkaline phosphatase (ALP) level >1.5X the ULN for the reference range, or
    - total bilirubin level (TBL) >1.2X the ULN for the reference range (except for cases of known Gilbert's Syndrome)

    Note: Participants with NAFLD <u>are eligible</u> to participate in this trial if their ALT level is ≤3.0X the ULN for the reference range.
28. Have a serum calcitonin level (at Visit 1) of:
    - ≥20 ng/L, if eGFR ≥60 mL/min/1.73 m^2
    - ≥35 ng/L, if eGFR <60 mL/min/1.73 m^2
29. Have a family or personal history of medullary thyroid carcinoma (MTC) or multiple endocrine neoplasia (MEN) syndrome type 2
30. Have a history of an active or untreated malignancy or are in remission from a clinically significant malignancy (other than basal or squamous cell skin cancer, in situ carcinomas of the cervix, or in situ prostate cancer) for less than 5 years
31. Have any other condition not listed in this section (for example, hypersensitivity or intolerance) that is a contraindication to GLP-1R agonists
32. Have a history of any other condition (such as known drug or alcohol abuse, diagnosed eating disorder, or other psychiatric disorder) that, in the opinion of the investigator, may preclude the participant from following and completing the protocol
33. Have history of use of marijuana or tetrahydrocannabinol (THC)-containing products within 3 months of enrollment or unwillingness to abstain from marijuana or THC-containing products use during the trial.

Note: If a participant has used cannabidiol oil during the past 3 months but agrees to refrain from use for the duration of the study, the participant can be enrolled.

34. Have had a transplanted organ (corneal transplants [keratoplasty] allowed) or awaiting an organ transplant
35. Have any hematological condition that may interfere with HbA1c measurement (for example, hemolytic anemias, sickle cell disease)

Prior/Concomitant Therapy

36. Are receiving or have received within 3 months prior to screening chronic (>2 weeks or >14 days) systemic glucocorticoid therapy (excluding topical, intraocular, intranasal, intraarticular, or inhaled preparations) or have evidence of a significant, active autoimmune abnormality (for example, lupus or rheumatoid arthritis) that has required (within the last 3 months) or is likely to require, in the opinion of the investigator, concurrent treatment with systemic glucocorticoids (excluding topical, intraocular, intranasal, intraarticular, or inhaled preparations) during the course of the study
37. Have a current or history of (within 3 months prior to randomization) treatment with medications that may cause significant weight gain, including but not limited to: tricyclic antidepressants, atypical antipsychotics, and mood stabilizers

    Examples:
    - imipramine
    - amitriptyline
    - mirtazapine
    - paroxetine
    - phenelzine
    - chlorpromazine
    - thioridazine
    - clozapine
    - olanzapine
    - valproic acid (and its derivatives), or
    - lithium

    Note: Selective serotonin reuptake inhibitors (SSRIs) other than paroxetine are permitted. Antihyperglycemic medications (for example, sulfonylureas, thiazolidinediones) for the management of T2DM are permitted.

38. Have taken within 3 months prior to randomization, medications (prescribed or over-the-counter) or alternative remedies that promote weight loss

    Examples include, but are not limited to:

    - Saxenda (liraglutide 3.0 mg)
    - Xenical®/Alli® (orlistat)

- Meridia® (sibutramine)
- Acutrim® (phenylpropanolamine)
- Sanorex® (mazindol)
- Apidex® (phentermine)
- BELVIQ® (lorcaserin)
- Bontril® (phendimetrazine)
- Qsymia™ (phentermine/topiramate combination)
- Contrave® (naltrexone/bupropion)

Note: Antihyperglycemic medications for the management of T2DM (for example, sodium-glucose cotransporter [SGLT]-2 inhibitors) are permitted.

39. Have started implantable or injectable contraceptives (such as Depo Provera®) within 18 months prior to screening

Prior/Concurrent Clinical Study Experience

40. Are currently enrolled in any other clinical study involving an investigational product (IP) or any other type of medical research judged not to be scientifically or medically compatible with this study
41. Within the last 30 days, have participated in a clinical study and received treatment, whether active or placebo. If the study involved an IP, 5 half-lives or 30 days, whichever is longer, should have passed
42. Have previously completed or withdrawn from this study or any other study investigating tirzepatide after receiving at least 1 dose of IP

Other Exclusions

43. Are investigator site personnel directly affiliated with this study and/or their immediate family. Immediate family is defined as a spouse, parent, child, or sibling, whether biological or legally adopted
44. Are Lilly employees

5.3. Lifestyle Considerations

Per the SoA, participants will consult a dietitian, or equivalent qualified delegate, according to local standards, to receive lifestyle management counseling at Weeks 0, 4, 8, and 12 during dose escalation and then at Week 24 and every 12 weeks thereafter through 72 weeks.

Diet and exercise goals established during the lifestyle consultation and the importance of adherence to the lifestyle component of the trial will be reinforced at each trial contact by study staff.

Study participants should be instructed not to donate blood or blood products during the study.

5.3.1. Meals and Dietary Restrictions

At Visit 3 and subsequent visits study participants will receive dietary counseling by a dietician, or equivalent qualified delegate, according to local standard. Dietary counseling will consist of

advice on healthy food choices and focus on calorie restriction using a hypocaloric diet with macronutrient composition of:

- maximum 30% of energy from fat,
- approximately 20% of energy from protein,
- approximately 50% of energy from carbohydrates, and
- an energy deficit of approximately 500 kcal/day compared to the participant's estimated total energy expenditure (TEE).

To encourage adherence, it is recommended that a 3-day diet and exercise log be completed prior to each counseling visit. During each visit, the participant's diet is reviewed and advice to maximize adherence is provided if needed.

The hypocaloric diet is continued throughout the treatment period. If a BMI ≤22 kg/m^2 is reached, the recommended energy intake should be recalculated with no kcal deficit for the remainder of the trial.

Total energy expenditure is calculated by multiplying the estimated basal metabolic rate (BMR) (see table below) with a Physical Activity Level value of 1.3 (FAO/WHO/UNU 2004), which reflects an inactive lifestyle. This calculation provides a conservative estimate of caloric requirements:

TEE (kcal/day) = BMR X 1.3

Equations for estimating BMR in kcal/day*

Sex	Age	BMR (kcal/day)
Men	18 to 30 years	15.057 X actual weight in kg + 692.2
	31 to 60 years	11.472 X actual weight in kg + 873.1
	>60 years	11.711 X actual weight in kg + 587.7
Women	18 to 30 years	14.818 X actual weight in kg + 486.6
	31 to 60 years	8.126 X actual weight in kg + 845.6
	>60 years	9.082 X actual weight in kg + 658.5

Abbreviation: BMR = basal metabolic rate; WHO = World Health Organization.
*Revised WHO equations (adapted from: FAO/WHO/UNU 2004).

5.3.2. Physical Activity

At Visit 3 and all subsequent visits, participants will be advised to increase their physical activity to moderate intensity (for example, brisk walking) for at least 150 minutes per week.

5.3.3. Diabetes Education

Diabetes education will be performed by personnel who are qualified to educate participants on symptoms and management of hyperglycemia and hypoglycemia, SMBG, self-injection and diabetes management. It will be provided to study participants at Visit 2 according to American Diabetes Association Standards of Medical Care in Diabetes (ADA 2020) or local standards. Additional trainings may be provided during the study as needed.

5.4. Screen Failures

Screen failures are defined as participants who consent to participate in the clinical study but are not subsequently randomly assigned to study intervention. A minimal set of screen failure information is required to ensure transparent reporting of screen failure participants to meet the Consolidated Standards of Reporting Trials (CONSORT) publishing requirements and to respond to queries from regulatory authorities. Minimal information includes demography, screen failure details, eligibility criteria, and any serious adverse event (SAE).

Individuals who do not meet the criteria for participation in this study (screen failure) may not be rescreened.

6. Study Intervention

Study intervention is defined as any investigational intervention(s), marketed product(s), placebo, or medical device(s) intended to be administered to/used by a study participant according to the study protocol.

6.1. Study Intervention(s) Administered

**Arm Name**	Tirzepatide 10 mg	Tirzepatide 15 mg	Placebo
**Dose**	10 mg QW	15 mg QW	N/A
**Route of Administration**	SC		
**Sourcing**	Provided centrally by the Sponsor and dispensed via IWRS		
**Packaging and Labeling**	Study intervention will be provided in autoinjectors (single-dose pens), packaged in cartons to be dispensed. Clinical study materials will be labeled according to country regulatory requirements.		

Abbreviations: IWRS = interactive web-response system; N/A = not applicable, QW = once weekly, SC = subcutaneous.

There are no restrictions on the time of day each weekly dose of study drug is given, but it is advisable to administer the SC injections on the same day and same time each week. The actual date, time, and injection site location of all dose administrations will be recorded in the diary by the participant. If a dose of study drug is missed, the participant should take it as soon as possible unless it is within 72 hours of the next dose, in which case, that dose should be skipped and the next dose should be taken at the appropriate time. The day of weekly administration can be changed if necessary, as long as the last dose was administered 72 or more hours before.

All participants will inject study drug subcutaneously in the abdomen or thigh using the injection supplies provided; a caregiver may administer the injection in the participant's upper arm. The injection site location of all dose administrations will be recorded by the participant. A new autoinjector will be used for each injection. If study drug is to always be injected in the same body region, participants should be advised to rotate injection sites each week.

6.1.1. Medical Devices

The combination products provided for use in the study are tirzepatide investigational autoinjector (or matching placebo). Any AEs resulting from device deficiencies, misuse, or malfunctions must be detected, documented, and reported by the investigator throughout the study (see Section 10.4.3).

6.2. Preparation/Handling/Storage/Accountability

- The investigator or designee must confirm appropriate storage conditions have been maintained during transit for all study intervention received and any discrepancies are reported and resolved before use of the study intervention.
- Only participants enrolled in the study may receive study intervention. Only study personnel may supply, prepare, or administer study intervention. All study intervention must be stored in a secure, environmentally controlled, and monitored (manual or automated) area in accordance with the labeled storage conditions with access limited to the investigator and authorized study personnel.
- The investigator or authorized study personnel are responsible for study intervention accountability, reconciliation, and record maintenance (in other words, receipt, reconciliation, and final disposition records).
- Further guidance and information for the final disposition of unused study interventions are provided in the study training materials.

6.3. Measures to Minimize Bias: Randomization and Blinding

This is a double-blind, randomized study.

Participants who meet all criteria for enrollment will be randomized to 1 of the study treatment groups at Visit 3. Assignment to treatment groups will be determined by a computer-generated random sequence using an IWRS. Participants will be randomized in a 1:1:1 ratio to receive tirzepatide 10 mg, tirzepatide 15 mg, or placebo.

The randomization will be stratified by country, sex (female, male), and type of AHM used at randomization (classified according to its potential effect on body weight, see table below).

Stratification Based on the Use of Antihyperglycemic Medications

Stratum	Potential Body Weight Effect	Participants to be Included in the Corresponding Stratum
1	Significant weight loss	• The use of an SGLT-2 inhibitor alone or in combination with any other AHMs except for an SU or TZD
2	Significant weight gain	• The use of an SU alone or in combination with any other AHMs except for an SGLT-2 inhibitor<br>OR<br>• The use of a TZD alone or in combination with any other AHMs except for an SGLT-2 inhibitor
3	Weight neutral and others	• Lifestyle management only: no background AHMs<br>OR<br>• Metformin monotherapy<br>OR<br>• The use of an SGLT-2 inhibitor in combination with an SU and/or TZD, with or without other AHMs<br>Note: The body weight effect of SGLT-2 inhibitor may be offset

Stratum	Potential Body Weight Effect	Participants to be Included in the Corresponding Stratum
		by SU/TZD, and vice-versa, when used in combination.<br>OR<br>• All other monotherapy (such as glinides and AGIs) or combination therapy which does not fall under stratum 1 or 2

Abbreviations: AGI = alpha-glucosidase inhibitor; AHM = antihyperglycemic medication; SGLT = sodium-glucose cotransporter; SU = sulfonylurea; TZD = thiazolidinedione.

Investigators, site staff, clinical monitors, and participants will remain blinded to the treatment assignments until the study is complete.

Emergency unblinding may be performed through the IWRS. This option may be used ONLY if the participant's well-being requires knowledge of the participant's treatment assignment. All unblinding events are recorded and reported by the IWRS.

If an investigator, site personnel performing assessments or participant is unblinded, the participant must be discontinued from the study. In cases where there are ethical reasons to have the participant remain on study drug, the investigator must obtain specific approval from a Lilly clinical research physician (CRP) for the participant to continue in the study.

In case of an emergency, the investigator has the sole responsibility for determining if unblinding of a participant's treatment assignment is warranted for medical management of the event. The participant's safety must always be the first consideration in making such a determination. If a participant's treatment assignment is unblinded, Lilly must be notified immediately. If the investigator decides that unblinding is warranted, it is the responsibility of the investigator to promptly document the decision and rationale and notify Lilly as soon as possible.

6.4. Study Intervention Compliance

Participant compliance with study intervention will be assessed at each visit. Compliance will be assessed by direct questioning and counting of unused study drug and/or empty cartons returned. Study drug compliance will be determined by the following:

- Study drug administration data will be recorded by the participant and reviewed by the investigator at each study visit.
- The participants will be instructed to return any unused study drug and/or empty cartons at the next visit to the study site for the purpose of performing drug accountability.

Treatment compliance for each visit interval is defined as taking at least 75% of the required doses of study drug. Similarly, a participant will be considered significantly noncompliant if he or she is judged by the investigator to have intentionally or repeatedly taken more than the prescribed amount of medication (more than 125%).

In addition to the assessment of a participant's compliance with the study drug administration, other aspects of compliance with the study treatments will be assessed at each visit based on the participant's adherence to the visit schedule, completion of study diaries, and any other parameters the investigator considers necessary.

Participants considered to be poorly compliant with their medication and/or the study procedures will receive additional training and instruction, as required, and will be reminded of the importance of complying with the protocol.

6.5. Concomitant Therapy

Participants will be permitted to use concomitant medications that they require during the study, except certain medications (for example, other medications for weight management, see Section 5) that may interfere with the assessment of efficacy and safety characteristics of the study treatments.

Investigative site staff will inform participants that they must consult with the investigator or a designated site staff member upon being prescribed any new medications during the study. This may not be possible when initiated for treatment of medical emergencies, in which case, the participant will inform the investigator or a designated site staff member as soon as possible.

Nonstudy medications taken by participants who are screened but not randomized will not be reported to Lilly unless an SAE or AE occurs that the investigator believes may have been caused by a study procedure.

6.5.1. Management of T2DM at randomization

Use of concomitant glucose lowering medications will be permitted at randomization, with the exception of GLP-1R agonist, DPP-4 inhibitors, or other injectable therapies (for example, insulin). **To minimize the risk for hypoglycemia, participants taking insulin secretagogues (for example, sulfonylurea) at randomization should have their dose halved (or stopped if already on the lowest dose) at randomization.** All other AHM will be continued at their current dose at randomization.

6.5.2. Initiation of New Antihyperglycemic Medication

The introduction of new AHMs other than study drug is expected during the study ONLY in the following situations:

- As a rescue therapy for the management of severe, persistent hyperglycemia as defined in Section 6.5.3.1
- In those participants who require permanent discontinuation of study drug, but remain in the study
- During the safety follow-up period

Amylin analogues/agonists, GLP-1R agonist and DPP-4 inhibitors are *not allowed* at any time during study, including the situations described above (after permanent discontinuation of study drug, as rescue therapy, and during safety follow-up).

6.5.3. Management of T2DM after randomization

6.5.3.1. Management of Severe, Persistent Hyperglycemia

Participants who develop persistent severe hyperglycemia during the treatment period may be candidates for glycemic rescue and should be considered for addition of or dose increase in antihyperglycemic therapies. The choice of new antihyperglycemic therapy (with the exception of amylin analogues/agonists, GLP-1R agonists, or DPP-4 inhibitors) or amount of dose increase will be at the discretion of the investigator or the participant's usual diabetes care physician, according to local and international guidelines for individualized treatment of patients with T2DM. Metformin is recommended to be used as a first-line rescue therapy for participants who receive diet/exercise alone; basal insulin may be considered for participants already receiving combination therapy of ≥3 AHMs at the time of study entry. The use of rescue therapy should be recorded on a specific eCRF page.

Glycemic rescue criteria will be primarily determined from weekly fasting SMBG values recorded in participant diaries. Investigators and/or the participant's usual care physician may initiate rescue therapy for the management of hyperglycemia if at least 2 confirmed values are:

- >270 mg/dL (15.0 mmol/L) over at least a consecutive 2-week period at any time during the first 8 weeks post-randomization OR
- >240 mg/dL (13.3 mmol/L) over at least a consecutive 2-week period at any time 9 to 16 weeks post-randomization OR
- >200 mg/dL (11.1 mmol/L) over at least a consecutive 2-week period at any time beyond the first 16 weeks post-randomization
  OR
- HbA1c ≥8.5% (69 mmol/mol) on and after Week 24 (only 1 value needed)

Note: Investigators should first confirm that the patient is fully compliant with the assigned therapeutic regimen and that he or she does not have an acute condition causing severe hyperglycemia.

Short-term treatment with insulin for less than 14 days is allowed for certain clinical situations (for example, elective surgery, during hospitalization, hyperosmolar states, or acute illness). This is not considered as rescue therapy and it must be differentiated from rescue therapy when reported in the eCRF.

For participants who discontinue study treatment permanently and continue to participate in the study, doses of background AHMs may be increased and new AHMs may be initiated at the discretion of investigators and/or participant's usual care physician. This is not considered as rescue therapy. It must be differentiated from rescue therapy when reported in the eCRF. Similarly, during the safety follow-up period, any increase in the dose of background AHM or the initiation of new AHM is not considered as rescue therapy either and must be differentiated from rescue therapy when reported in the eCRF.

6.5.3.2. Management of Hypoglycemia Risk

Participants will be trained in the signs and symptoms of hypoglycemia and its treatment and instructed to record pertinent information about hypoglycemic events in their study diary.

Participants who develop persistent or recurrent unexplained hypoglycemia during the treatment period will be asked to reduce the dose or discontinue any concomitant AHM, starting preferably with the ones commonly associated with hypoglycemia (for example, sulfonylurea). Monitoring for hypoglycemia includes capture of events as described in Sections 8.3.2.1 and 10.4 Appendix 4.

6.6. Dose Modification

6.6.1. Tirzepatide

Tirzepatide is given QW by SC injection. There are no restrictions on the time of day each weekly dose of tirzepatide is given, but it is advisable to administer the SC injections on the same day of the week and similar time each week. If a dose of tirzepatide is missed, the participant should take it as soon as possible unless it is within 72 hours of the next dose, in which case, that dose should be skipped and the next dose should be taken at the appropriate time.

Study drug dose modification is not permitted, except for management of intolerable GI symptoms (see Section 6.6.2).

6.6.2. Management of Participants with Gastrointestinal Symptoms

Participants who experience intolerable GI symptoms (for example, nausea, vomiting, or diarrhea) at any time during the study should first be counseled on dietary behaviors that may help mitigate nausea and vomiting (for example, eating smaller meals, splitting 3 daily meals into 4 or more smaller ones, and stopping eating when they feel full) (see Section 10.9). If symptoms persist, the participant should be prescribed, at the investigator’s discretion, symptomatic medication (for example, antiemetic or antidiarrheal medication). A temporary interruption of study drug for 1 dose is permitted, provided the participant has taken the last 3 weekly doses. Study treatment should be resumed immediately, either alone or in combination with symptomatic medication, which can also be utilized to manage symptoms. Management of study drug after interruptions >1 dose is discussed in Section 7.1.1.

If intolerable GI symptoms (for example, nausea, vomiting, or diarrhea) persist despite the above measures, the investigator should contact Lilly to consider reinitiating study drug at the next-lower maintenance dose in a blinded fashion (for example, 15 mg and 12.5 mg reduced to 10 mg, 10 mg or lower reduced to placebo). Only 1 dose reduction per participant will be permitted during the course of the study.

If intolerable GI symptoms persist despite symptomatic treatment, temporary drug interruption, and resumption at a lower dose of study drug, the participant should be discontinued from the study drug. All participants who discontinue study drug should continue to attend scheduled study visits. All dose adjustments will be managed through IWRS.

6.7. Intervention after the End of the Study

Tirzepatide will not be made available to participants after conclusion of the study.

7. Discontinuation of Study Intervention and Participant Discontinuation/Withdrawal

7.1. Discontinuation of Study Intervention

In rare instances, it may be necessary for a participant to permanently discontinue (definitive discontinuation) study intervention. If study intervention is definitively discontinued, the participant will remain in the study to be evaluated for any trial endpoint at the end of the study. See the SoA for data to be collected at the time of discontinuation of study intervention and follow-up and for any further evaluations that need to be completed.

Possible reasons leading to permanent discontinuation of IP:

- **Participant Decision**
    - The participant requests to discontinue IP
- **Clinical Considerations**
    - Initiation of open-label GLP-1R agonist, amylin analogues/agonists, or DPP-4 inhibitor, if participants will not or cannot discontinue them
    - Intolerable GI symptoms despite management as described in Section 6.6.2
    - BMI ≤18.5 kg/m^2 is reached at any time during the treatment period

      Note: The investigator should contact the sponsor CRP to discuss whether it is medically appropriate for the participant to continue study treatment.
    - Diagnosis of T1DM
    - Diagnosis of MTC or MEN syndrome type 2
    - Significant elevation of serum calcitonin (Section 8.3.2.4)
    - Diagnosis of acute or chronic pancreatitis
    - Diagnosis of an active or untreated malignancy (other than basal or squamous cell skin cancer, in situ carcinomas of the cervix, or in situ prostate cancer)
    - If the investigator, after consultation with the sponsor-designated medical monitor, determines that a systemic hypersensitivity reaction has occurred related to study drug administration, the participant should be permanently discontinued from the investigational drug
    - Onset of pregnancy in a female participant
    - Occurrence of any other treatment-emergent AE (TEAE), SAE, or clinically significant finding for which the investigator believes that permanent study drug discontinuation is the appropriate measure to be taken

- Inadvertent enrollment if continued treatment with study drug would not be medically appropriate.
- PHQ-9 score ≥15
  - Participants should be referred to a mental health professional (MHP) to assist in deciding whether the subject should be discontinued from study drug. If a participant's psychiatric disorder can be adequately treated with psycho- and/or pharmacotherapy, then the subject, at the discretion of the investigator (in agreement with the MHP), may be continued in the trial on randomized therapy.
- in addition, study drug may be discontinued if participants:
  - answered "yes" to Question 4 (Active Suicidal Ideation with Some Intent to Act, Without Specific Plan) on the "Suicidal Ideation" portion of the C-SSRS

  or

  - answered "yes" to Question 5 (Active Suicidal Ideation with Specific Plan and Intent) on the "Suicidal Ideation" portion of the C-SSRS

  or

  - answered "yes" to any of the suicide-related behaviors (Actual attempt, Interrupted attempt, Aborted attempt, Preparatory act or behavior) on the "Suicidal Behavior" portion of the C-SSRS

Note: A psychiatrist or appropriately trained professional may assist in the decision to discontinue the participant.

- **Discontinuation due to a hepatic event or liver test abnormality**
  - Participants who are discontinued from IP due to a hepatic event or liver test abnormality should have additional hepatic safety data collected via eCRF
  - Discontinuation of the IP for abnormal liver tests **should be** considered by the investigator when a participant meets 1 of the following conditions after consultation with the Lilly designated medical monitor:
    - ALT or aspartate aminotransferase (AST) >8X ULN
    - ALT >2X baseline value or ≥300 U/L, whichever occurs first, if baseline ALT ≥2X ULN
    - ALT or AST >5X ULN for more than 2 weeks
    - ALT or AST >3X ULN and TBL >2X ULN or international normalized ratio (INR) >1.5

- ALT or AST >3X ULN with the appearance of fatigue, nausea, vomiting, right-upper-quadrant pain or tenderness, fever, rash, and/or eosinophilia (>5%)
- ALP >3X ULN
- ALP >2.5X ULN and TBL >2X ULN
- ALP >2.5X ULN with the appearance of fatigue, nausea, vomiting, right-upper-quadrant pain or tenderness, fever, rash, and/or eosinophilia (>5%)

Participants who stop the study drug permanently should continue to attend all scheduled study visits to collect all planned efficacy and safety measurements. Participants who stop the study treatment post-randomization and prior to 72 weeks but are unwilling to attend remaining scheduled visits should return for Visit 99 for a final weight measurement. If these participants are unwilling to attend Visit 99, their refusal to attend should be documented in the patient medical record.

See the SoA for data to be collected at the time of intervention discontinuation and follow-up and for any further evaluations that need to be completed.

7.1.1. Temporary Study Drug Discontinuation

In certain situations, after randomization, the investigator may need to temporarily interrupt study drug. Every effort should be made by the investigator to maintain participants on study drug and to restart study drug after any temporary interruption, as soon as it is safe to do so. Distribution of study medication at the correct dose will be per IWRS instructions.

If study drug interruption is…	then…
2 consecutive doses or less	participant restarts study drug at last administered dose, as per escalation schedule.
3 consecutive doses or more	participant restarts study drug (at 5 mg, managed by IWRS) and repeats dose escalation scheme.
due to an AE	the event is to be documented and followed according to the procedures in Section 8.3 of this protocol.
due to intolerable persistent GI AE	participants should be treated as suggested in Section 6.6.2.

Abbreviations: AE = adverse event; GI = gastrointestinal; IWRS = interactive web response service.

Investigators should inform the sponsor that study drug has been temporarily interrupted. The data related to temporary interruption of study treatment will be documented in source documents and entered on the eCRF.

7.2. Participant Discontinuation/Withdrawal from the Study

To minimize the amount of missing data and to enable assessment of study objectives as planned in the study protocol, every attempt will be made to keep participants in the study irrespective of the following:

- adherence to or discontinuation from study drug
- adherence to visit schedule
- missing assessments
- study drug discontinuation due to AE
- development of comorbidities
- development of clinical outcomes

The circumstances listed above are ***not*** valid reasons for discontinuation from the study.

A participant may withdraw from the study:

- at any time at his/her own request
- at the request of his/her designee (for example, parents or legal guardian)
- at the discretion of the investigator for safety, behavioral, compliance, or administrative reasons
- if enrolled in any other clinical study involving an IP or enrolled in any other type of medical research judged not to be scientifically or medically compatible with this study
- if the participant, for any reason, requires treatment (pharmacological, device based or surgical) that has been demonstrated to be effective for treatment of the study indication, discontinuation from the study occurs prior to introduction of the new treatment

Female participants will be withdrawn from the study if the participant becomes pregnant.

Participation in the study can be stopped for medical, safety, regulatory, or other reasons consistent with applicable laws, regulations, and good clinical practice (GCP). Participants who agree to provide information relevant to any trial endpoint at the end of the study are not considered to have discontinued from the study.

At the time of discontinuing from the study, if possible, an ED visit should be conducted, as shown in the SoA (Section 1.3) (see also Section 4.1.1). See SoA for data to be collected at the time of study discontinuation and safety follow-up and for any further evaluations that need to be

completed. The participant will be permanently discontinued both from the study intervention and from the study at that time.

If the participant withdraws consent for disclosure of future information, the sponsor may retain and continue to use any data collected before such a withdrawal of consent. If a participant withdraws from the study, he/she may request destruction of any samples taken and not tested, and the investigator must document this in the site study records.

7.2.1. Participant disposition and timing of safety follow-up

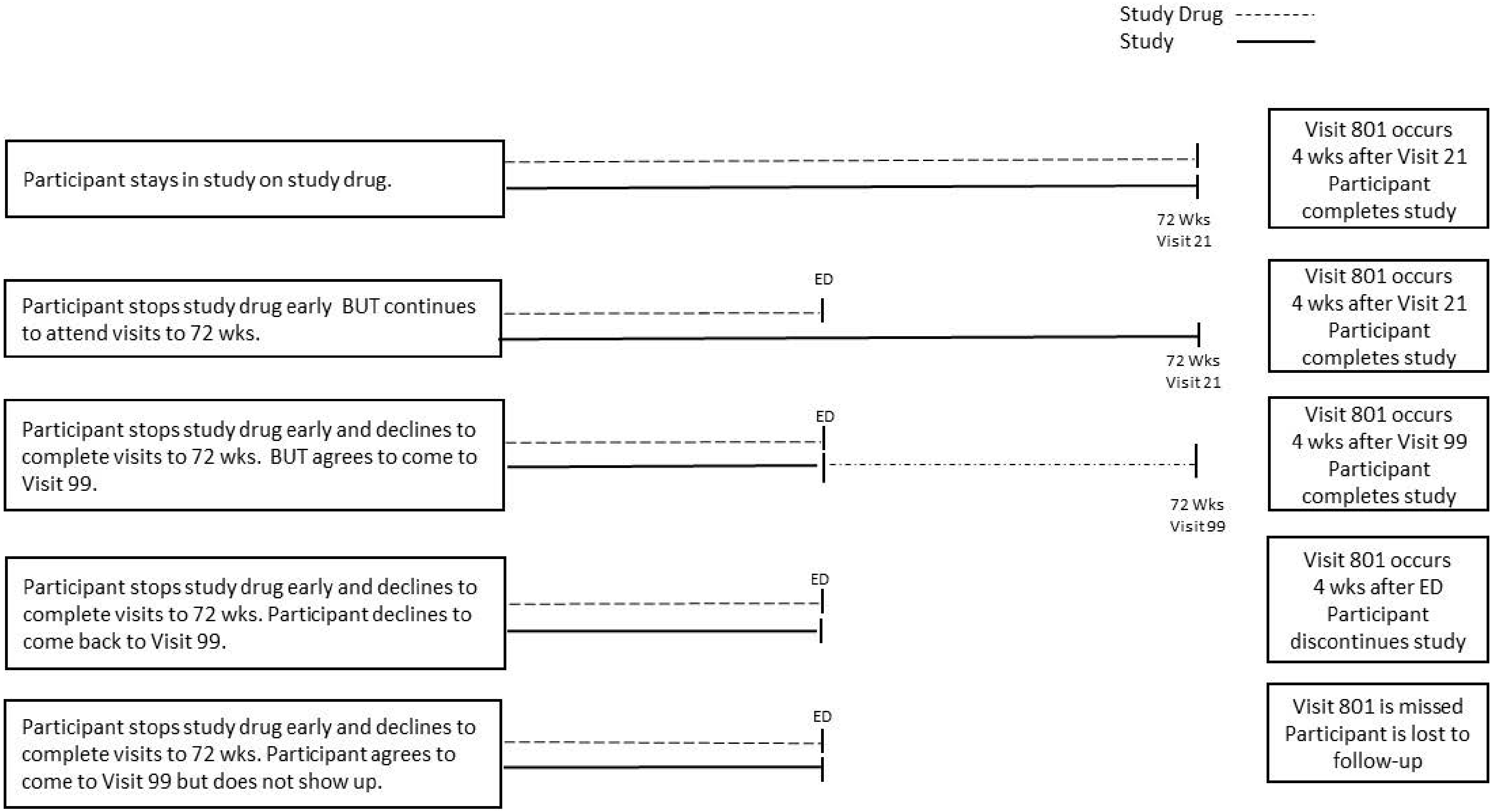

Abbreviations: ED= early discontinuation, wks= weeks.

7.2.2. Discontinuation of Inadvertently Enrolled Participants

If the sponsor or investigator identifies a participant who did not meet enrollment criteria and was inadvertently enrolled, the investigator and the sponsor CRP must agree whether continuing the study treatment is medically appropriate. Continuation of inadvertently enrolled participants in the study, with or without study drug treatment, requires documented approval from the sponsor CRP. Safety follow-up should be performed as outlined in Section 1.3 (SoA), Section 8.3 (Adverse Events and Serious Adverse Events), and Section 8.2 (Safety Assessments) of the protocol.

7.3. Lost to Follow-up

A participant will be considered lost to follow-up if he or she repeatedly fails to return for scheduled visits and is unable to be contacted by the study site. Site personnel or designee are expected to make diligent attempts to contact participants who fail to return for a scheduled visit or were otherwise unable to be followed up by the site.

Site personnel, or an independent third party, will attempt to collect the vital status of the participant within legal and ethical boundaries for all participants randomized, including those who did not get IP. Public sources may be searched for vital status information. If vital status is determined to be deceased, this will be documented, and the participant will not be considered lost to follow-up.

Lilly personnel will not be involved in any attempts to collect vital status information.

Discontinuation of specific sites or of the study as a whole are handled as part of Section 10.1 (Appendix 1).

8. Study Assessments and Procedures

- Study procedures and their timing are summarized in the SoA (Section 1.3).
- Immediate safety concerns should be discussed with the sponsor immediately upon occurrence or awareness to determine if the participant should continue or discontinue study intervention.
- Adherence to the study design requirements, including those specified in the SoA, is essential and required for study conduct.
- All screening evaluations must be completed and reviewed to confirm that potential participants meet all eligibility criteria. The investigator will maintain a screening log to record details of all participants screened and to confirm eligibility or record reasons for screening failure, as applicable.

8.1. Efficacy Assessments

8.1.1. Primary Efficacy Assessments

The primary efficacy measurement in this study is body weight. Body weight measurements will be collected at specific clinic visits as summarized in the SoA. Methods for measuring body weight are described in Section 10.7 (Appendix 7).

8.1.2. Secondary Efficacy Assessments

The following secondary efficacy measures will be collected at the times shown in the SoA:

- BMI (derived using body weight in kilograms divided by the square of height in meters)
- Waist circumference (measuring method is described in Section 10.7)
- HbA1c (measured through central lab)
- Fasting glucose (measured through central lab)
- Fasting insulin (measured through central lab)
- Blood pressure (measuring method is described in Section 10.7)
- Lipids (measured through central lab)

8.1.3. Patient-Reported Outcomes Assessments

The self-administered questionnaires will be translated into the native language of the region, linguistically validated and administered according to the SoA (Section 1.3). At these visits, the questionnaires should be completed before the participant has discussed their medical condition or progress in the study with the investigator and/or site staff, if the participant is not adversely affected by their fasting condition.

8.1.3.1. Short Form-36 version 2 Health Survey acute form, 1-week recall version

The SF-36v2 acute form, 1-week recall version is a 36-item generic, participant-administered measure designed to assess the following 8 domains:

- Physical Functioning
- Role-Physical
- Bodily Pain
- General Health
- Vitality
- Social Functioning
- Role-Emotional
- Mental Health.

The Physical Functioning domain assesses limitations due to health “now” while the remaining domains assess functioning “in the past week.” Each domain is scored individually and information from these 8 domains are further aggregated into 2 health-component summary scores: Physical Component Summary and Mental Component Summary. Items are answered on Likert scales of varying lengths (3-point, 5-point, or 6-point scales). Scoring of each domain and both summary scores are norm-based and presented in the form of T-scores, with a mean of 50 and standard deviation (SD) of 10; higher scores indicate better levels of function and/or better health (Maruish 2011).

8.1.3.2. Impact of Weight on Quality of Life-Lite Clinical Trials Version

The IWQOL-Lite-CT is a 20-item, obesity-specific PRO instrument developed for use in obesity clinical trials. It assesses 2 primary domains of obesity-related health-related quality of life (HRQoL): Physical (7 items) and Psychosocial (13 items). A 5-item subset of the Physical domain – the Physical Function composite – is also supported. Items in the Physical Function composite describe physical impacts related to general and specific physical activities. All items are rated on either a 5-point frequency (“never” to “always”) scale or a 5-point truth (“not at all true” to “completely true”) scale (Kolotkin et al. 2017, 2019).

8.1.3.3. EQ-5D-5L

Generic HRQoL will be assessed using the EQ-5D-5L (EuroQoL Research Foundation 2019). The EQ-5D-5L is a standardized 5-item instrument for use as a measure of health outcome. It provides a simple descriptive profile and a single index value for health status that can be used in the clinical and economic evaluation of health care as well as population health surveys. The EQ-5D-5L comprises 5 dimensions of health (mobility, self-care, usual activities, pain/discomfort, and anxiety/depression). The 5L version, introduced in 2005, scores each dimension at 5 levels (no problems, slight problems, moderate problems, severe problems, unable to perform/extreme problems) for a total of 3125 possible health states. In addition to the health profile, a single health state index value can be derived based on a formula that attaches weights to each of the levels in each dimension. This index value ranges between less than 0

(where 0 is a health state equivalent to death; negative values are valued as worse than dead) to 1 (perfect health) (Dolan 1997). In addition, the EuroQol Visual Analog Scale records the respondent's self-rated health status on a vertical graduated (0 to 100) visual analog scale. In conjunction with the health state data, it provides a composite picture of the respondent's health status.

The EQ-5D-5L is used worldwide and is available in more than 170 different languages. Details on the instrument, scoring, organizing, and presenting the data collected can be found in the EQ-5D-5L User Guide (EuroQoL Research Foundation 2019).

8.1.3.4. Patient Global Impression of Status for Physical Activity

Study participants will be asked to complete a PGIs item specifically developed for this study. This is a participant-rated assessment of current limitation on physical activity due to health and is rated on a 5-point scale ranging from "1- not at all limited" to "5- extremely limited."

8.2. Safety Assessments

Planned time points for all safety assessments are provided in the SoA.

8.2.1. Physical Examinations

- A complete physical examination will include, at a minimum, assessments of the cardiovascular, respiratory, GI, and neurological systems, as well as a thyroid exam. Height, weight, and waist circumference will also be measured and recorded, per Section 10.7.
- Investigators should pay special attention to clinical signs related to previous serious illnesses.

8.2.2. Vital Signs

For each participant, vital signs measurements should be conducted according to the SoA (Section 1.3) and Section 10.7 (Appendix 7).

Any clinically significant findings from vital signs measurement that result in a diagnosis should be reported to Lilly or its designee as an AE via the eCRF.

8.2.3. Electrocardiograms

For each participant, single tracing 12-Lead ECGs should be collected according to the SoA and Section 10.7.

Electrocardiograms will initially be interpreted by a qualified physician (the investigator or qualified designee) at the site as soon after the time of ECG collection as possible and ideally while the participant is still present, for immediate subject management, should any clinically relevant findings be identified. Any clinically significant findings from ECGs that result in a diagnosis should be reported to Lilly or its designee as an AE via the eCRF.

All digital ECGs will be obtained using centrally provided ECG machines and will be electronically transmitted to a designated central ECG laboratory. The central ECG laboratory will perform a basic quality control check (for example, demographics and study details) and

then store the ECGs in a database. At a future time, the stored ECG data may be overread by a cardiologist at the central ECG laboratory for further evaluation of machine-read measurements or to meet regulatory requirements. The machine-read ECG intervals and heart rate may be used for data analysis and report-writing purposes, unless a cardiology overreading of the ECGs is conducted prior to completion of the final study report (in which case, the overread data would be used).

8.2.4. Clinical Safety Laboratory Assessments

- See Section 10.2 Appendix 2: "Clinical Laboratory Tests" for the list of clinical laboratory tests to be performed and to the SoA for the timing and frequency.

  Note: See Section 10.2 Appendix 2: "Clinical Laboratory Tests" for clinical laboratory tests reported or not reported to the investigators

- The investigator must review the laboratory results, document this review, and report any clinically relevant changes occurring during the study as an AE. The laboratory results must be retained with source documents unless a Source Document Agreement or comparable document cites an electronic location that accommodates the expected retention duration. Clinically significant abnormal laboratory findings are those which are not associated with the underlying disease, unless judged by the investigator to be more severe than expected for the participant's condition.

- All laboratory tests with values considered clinically significantly abnormal during participation in the study or within 4 weeks after the last dose of study intervention should be repeated until the values return to normal or baseline or are no longer considered clinically significant by the investigator or medical monitor.

  - If such values do not return to normal/baseline within a period of time judged reasonable by the investigator, the etiology should be identified, and the sponsor notified.

  - All protocol-required laboratory assessments, as defined in Section 10.2 Appendix 2, must be conducted in accordance with the SoA, standard collection requirements, and laboratory manual.

- If laboratory values from nonprotocol-specified laboratory assessments performed at an investigator-designated local laboratory require a change in participant management or are considered clinically significant by the investigator (e.g., SAE or AE or dose modification), then report the information as an AE.

Repeat or unscheduled samples may be taken for safety reasons or for technical issues with the samples. Unless otherwise stated in the subsections below, all samples collected for specified laboratory tests will be destroyed within 60 days of receipt of confirmed test results. Certain

samples may be retained for a longer period, if necessary, to comply with applicable laws, regulations, or laboratory certification standards.

8.2.5. Safety Monitoring

Lilly will periodically review evolving aggregate safety data within the study by appropriate methods. The study team will review safety reports in a blinded fashion according to the schedule provided in the Trial-Level Safety Review plan. Lilly will also review SAEs within time frames mandated by company procedures. The Lilly CRP will, as appropriate, consult with the functionally independent Global Patient Safety therapeutic area physician or clinical scientist.

8.2.5.1. Hepatic Safety Monitoring

Close hepatic monitoring

Laboratory tests (Section 10.6 Appendix 6), including ALT, AST, ALP, TBL, direct bilirubin, gamma-glutamyltransferase, and creatine kinase, should be repeated within 48 to 72 hours to confirm the abnormality and to determine if it is increasing or decreasing, if 1 or more of these conditions occur:

If a participant with baseline results of…	develops the following elevations:
ALT or AST <1.5X ULN	ALT or AST ≥3X ULN
ALP <1.5X ULN	ALP ≥2X ULN
TBL <1.5X ULN	TBL ≥2X ULN (except for patients with Gilbert's syndrome)
ALT or AST ≥1.5X ULN	ALT or AST ≥2X baseline
ALP ≥1.5X ULN	ALP ≥2X baseline
TBL ≥1.5X ULN	TBL ≥1.5X baseline (except for patients with Gilbert's syndrome)

Abbreviations: ALP = alkaline phosphatase; ALT = alanine aminotransferase; AST = aspartate aminotransferase; TBL = total bilirubin level; ULN = upper limit of normal.

If the abnormality persists or worsens, clinical and laboratory monitoring and evaluation for possible causes of abnormal liver tests should be initiated by the investigator in consultation with the Lilly-designated medical monitor. At a minimum, this evaluation should include physical examination and a thorough medical history, including symptoms, recent illnesses (for example, heart failure, systemic infection, hypotension, or seizures), recent travel, history of concomitant medications (including over-the-counter), herbal and dietary supplements, history of alcohol drinking, and other substance abuse.

Initially, monitoring of symptoms and hepatic biochemical tests should be done at a frequency of 1 to 3 times weekly, based on the participant's clinical condition and hepatic biochemical tests. Subsequently, the frequency of monitoring may be lowered to once every 1 to 2 weeks, if the participant's clinical condition and lab results stabilize. Monitoring of ALT, AST, ALP, and TBL should continue until levels normalize or return to approximate baseline levels.

Comprehensive hepatic evaluation

A comprehensive evaluation should be performed to search for possible causes of liver injury if 1 or more of these conditions occur:

If a participant with baseline results of…	develops the following elevations:
ALT or AST <1.5X ULN	ALT or AST ≥3X ULN with hepatic signs/symptoms*, or ALT or AST ≥5X ULN
ALP <1.5X ULN	ALP ≥3X ULN
TBL <1.5X ULN	TBL ≥2X ULN (except for patients with Gilbert's syndrome)
ALT or AST ≥1.5X ULN	ALT or AST ≥2X baseline with hepatic signs/symptoms*, or ALT or AST ≥3X baseline
ALP ≥1.5X ULN	ALP ≥2X baseline
TBL ≥1.5X ULN	TBL ≥2X baseline (except for patients with Gilbert's syndrome)

Abbreviations: ALP = alkaline phosphatase; ALT = alanine aminotransferase; AST = aspartate aminotransferase; TBL = total bilirubin level; ULN = upper limit of normal.

* Hepatic signs/symptoms are severe fatigue, nausea, vomiting, jaundice, right-upper-quadrant abdominal pain, fever, rash, and/or eosinophilia >5%.

At a minimum, this evaluation should include physical examination and a thorough medical history, as outlined above, as well as tests for prothrombin time -INR; tests for viral hepatitis A, B, C, or E; tests for autoimmune hepatitis; and an abdominal imaging study (for example, ultrasound or computed tomography [CT] scan).

Based on the patient's history and initial results, further testing should be considered in consultation with the Lilly-designated medical monitor, including tests for hepatitis D virus, cytomegalovirus, Epstein-Barr virus, acetaminophen levels, acetaminophen protein adducts, urine toxicology screen, Wilson's disease, blood alcohol levels, urinary ethyl glucuronide, and blood phosphatidylethanol. Based on the circumstances and the investigator's assessment of the participant's clinical condition, the investigator should consider referring the participant for a hepatologist or gastroenterologist consultation, magnetic resonance cholangiopancreatography, endoscopic retrograde cholangiopancreatography, cardiac echocardiogram, or a liver biopsy.

Additional hepatic data collection (hepatic safety CRF) in study participants who have abnormal liver tests during the study

Additional hepatic safety data collection in hepatic safety case report form (CRF) should be performed in study participants who meet 1 or more of the following 5 conditions:

1. Elevation of serum ALT to ≥5X ULN on 2 or more consecutive blood tests (if baseline ALT <1.5X ULN)
    - In participants with baseline ALT ≥1.5X ULN, the threshold is ALT ≥3X baseline on 2 or more consecutive tests

2. Elevated TBL to ≥ 2X ULN (if baseline TBL <1.5X ULN) (except for cases of known Gilbert's syndrome)
    - In participants with baseline TBL ≥1.5X ULN, the threshold should be TBL ≥2X baseline
3. Elevation of serum ALP to ≥2X ULN on 2 or more consecutive blood tests (if baseline ALP <1.5X ULN)
    - In participants with baseline ALP ≥1.5X ULN, the threshold is ALP ≥2X baseline on 2 or more consecutive blood tests
4. Hepatic event considered to be an SAE
5. Discontinuation of study drug due to a hepatic event

Note: The interval between the 2 consecutive blood tests should be at least 2 days.

8.2.6. Depression, Suicidal Ideation, and Behavior Risk Monitoring

Patients who have obesity or are overweight are at increased risk for depression (Luppino et al. 2010). Depression can increase the risk for suicidal ideation and behavior. Therefore, study participants will be screened at trial entry and monitored during the study for depression, suicidal ideation, and behavior.

Participants should be monitored appropriately and observed closely for suicidal ideation and behavior or any other unusual changes in behavior, especially at the beginning and end of the course of treatment, or at the time of dose changes, either increases or decreases. Consideration should be given to discontinuing the study medication in subjects who experience signs of suicidal ideation or behavior, following a risk assessment (Section 7.1).

Baseline and treatment-emergent (TE) assessment of depression, suicidal ideation, and behavior will be monitored during the study using the C-SSRS and PHQ-9 (Section 8.3.2.13).

8.3. Adverse Events, Serious Adverse Events, and Product Complaints

The definitions of the following events can be found in Section 10.4 Appendix 4:

- Adverse events (AEs)
- Serious adverse events (SAEs)
- Product complaints (PCs)

These events will be reported by the participant (or, when appropriate, by a caregiver, surrogate, or the participant's legally authorized representative).

The investigator and any qualified designees are responsible for detecting, documenting, and recording events that meet these definitions and remain responsible for following up events that are serious, considered related to the study intervention, study device or device constituent, or study procedures, or that caused the participant to discontinue the study intervention (see Section 7).

Care will be taken not to introduce bias when detecting events. Open-ended and nonleading verbal questioning of the participant is the preferred method to inquire about event occurrences.

After the initial report, the investigator is required to proactively follow each participant at subsequent visits/contacts. All SAEs will be followed until resolution, stabilization, the event is otherwise explained, or the participant is lost to follow-up (as defined in Section 7.3). For product complaints, the investigator is responsible for ensuring the follow-up includes any supplemental investigations as indicated to elucidate the nature and/or causality. Further information on follow-up procedures is provided in Section 10.4 (Appendix 4).

8.3.1. Timing and Mechanism for Collecting Events

This table describes the timing, deadlines, and mechanism for collecting events.

**Event**	**Collection Start**	**Collection Stop**	**Timing for Reporting to Sponsor or Designee**	**Mechanism for Reporting**	**Back-up Method of Reporting**
**Adverse Event**					
AE	signing of the informed consent form (ICF)	the safety follow-up visit OR participation in study has ended	As soon as possible upon site awareness	AE eCRF	N/A
**Serious Adverse Event**					
SAE and SAE updates – prior to start of study intervention **and** deemed reasonably possibly related with study procedures	signing of the ICF	start of intervention	Within 24 hours of awareness	SAE eCRF	SAE paper form
SAE and SAE updates – after start of study intervention	start of intervention	The safety follow-up visit OR participation in study has ended	Within 24 hours of awareness	SAE eCRF	SAE paper form

Event	Collection Start	Collection Stop	Timing for Reporting to Sponsor or Designee	Mechanism for Reporting	Back-up Method of Reporting
SAE – after participant's study participation has ended **and** the investigator becomes aware	After participant's study participation has ended	N/A	Promptly	SAE paper form	N/A
**Pregnancy**					
Pregnancy in female participants and female partners of male participants	After the start of study intervention	Four months after the last injection for female partners of male participants and 2 months after the last injection for female participants	Within 24 hours of learning of the pregnancy	eCRF	SAE paper form
**Product Complaints**					
PC associated with an SAE or might have led to an SAE	Start of study intervention	End of study intervention	Within 24 hours of awareness	Product Complaint form	N/A
PC not associated with an SAE	Start of study intervention	End of study intervention	Within 1 business day of awareness	Product Complaint form	N/A
Updated PC information	—	—	As soon as possible upon site awareness	Originally completed Product Complaint form with all changes signed and dated by the investigator	N/A

Event	Collection Start	Collection Stop	Timing for Reporting to Sponsor or Designee	Mechanism for Reporting	Back-up Method of Reporting
PC (after participant's study participation has ended **and** the investigator becomes aware)	After participant's study participation has ended	N/A	Promptly	Product Complaint form	N/A

Abbreviations: AE = adverse event; eCRF = electronic case report form; N/A = not applicable; PC = product complaint; SAE = serious adverse event.

8.3.2. Special Safety Topics

8.3.2.1. Hypoglycemia

Upon ICF signing, all participants will be educated about signs and symptoms of hypoglycemia, how to treat hypoglycemia, and how to collect appropriate information for each episode of hypoglycemia.

Participants who develop persistent or recurrent unexplained hypoglycemia during the treatment period will be asked to reduce the dose or discontinue any concomitant AHMs commonly associated with hypoglycemia (for example, sulfonylurea).

All hypoglycemic episodes will be recorded on a specific eCRF (Hypoglycemic Events eCRF) and should not be recorded on the AE eCRF, unless the event meets serious criteria. If a hypoglycemic event meets severe criteria (see definition below), it should be recorded as serious on the AE and SAE eCRFs, and reported to Lilly as an SAE. All post-treatment hypoglycemic events collected on the hypoglycemic eCRF will be considered as treatment-emergent and included in the analysis of incidence and rate of hypoglycemic events.

Investigators should use the following definitions and criteria when diagnosing and categorizing an episode considered to be related to hypoglycemia (the BG values in this section refer to values determined by a laboratory or International Federation of Clinical Chemistry and Laboratory Medicine blood-equivalent glucose meters and strips) in accordance with the 2020 American Diabetes Association position statement on glycemic targets (ADA 2020):

Glucose alert value (Level 1):

- **Documented symptomatic hypoglycemia** is defined as any time a participant feels that he or she is experiencing symptoms and/or signs associated with hypoglycemia and has a BG level of <70 mg/dL (<3.9 mmol/L).

- **Documented asymptomatic hypoglycemia** is defined as any event not accompanied by typical symptoms of hypoglycemia but with a measured BG <70 mg/dL (<3.9 mmol/L).
- **Documented unspecified hypoglycemia** is defined as any event with no information about symptoms of hypoglycemia available but with a measured BG <70 mg/dL (<3.9 mmol/L).

Clinically significant hypoglycemia (Level 2):

- **Documented symptomatic hypoglycemia** is defined as any time a participant feels that he or she is experiencing symptoms and/or signs associated with hypoglycemia and has a BG level of <54 mg/dL (<3.0 mmol/L).
- **Documented asymptomatic hypoglycemia** is defined as any event not accompanied by typical symptoms of hypoglycemia but with a measured BG level <54 mg/dL (<3.0 mmol/L).
- **Documented unspecified hypoglycemia** is defined as any event with no information about symptoms of hypoglycemia available but with a measured BG level <54 mg/dL (<3.0 mmol/L).

Severe hypoglycemia (Level 3):

- **Severe hypoglycemia** is defined as an episode with severe cognitive impairment requiring the assistance of another person to actively administer carbohydrate, glucagon, or other resuscitative actions. These episodes may be associated with sufficient neuroglycopenia to induce seizure or coma. Blood glucose measurements may not be available during such an event, but neurological recovery attributable to the restoration of BG to normal is considered sufficient evidence that the event was induced by a low BG concentration.

Nocturnal hypoglycemia:

- **Nocturnal hypoglycemia** is defined as any hypoglycemic event that occurs between bedtime and waking.

To avoid duplicate reporting, all consecutive BG values <70 mg/dL (3.9 mmol/L) occurring within a 1-hour period may be considered to be a single hypoglycemic event (Weinberg et al. 2010; Danne et al. 2013).

8.3.2.2. Pancreatitis

Diagnosis of acute pancreatitis

Acute pancreatitis is an AE of interest in all studies with tirzepatide, including this study. The diagnosis of acute pancreatitis requires 2 of the following 3 features (Banks et al. 2006; Koizumi et al. 2006):

- abdominal pain, characteristic of acute pancreatitis (that is, epigastric pain radiating to the back, often associated with nausea and vomiting)

- serum amylase (total, pancreatic, or both) and/or lipase ≥3X ULN
- characteristic findings of acute pancreatitis on CT scan or magnetic resonance imaging (MRI)

If acute pancreatitis is suspected, the investigator should:

- obtain appropriate laboratory tests, including pancreatic amylase (p-amylase) and lipase
- perform imaging studies, such as abdominal CT scan with or without contrast, abdominal MRI, or gallbladder ultrasound

***Note*:** Abdominal ultrasound may be used as an alternative method only if CT and MRI cannot be performed

- evaluate for possible causes of acute pancreatitis, including alcohol use, gallstone/gall bladder disease, hypertriglyceridemia, and concomitant medications.

Discontinuation for acute pancreatitis

If acute pancreatitis is diagnosed, the participant must discontinue use of the IP, but will continue in the study.

Case adjudication and data entry

An independent clinical endpoint committee (CEC) will adjudicate all suspected cases of acute pancreatitis. In addition, AEs of severe or serious abdominal pain of unknown etiology will also be submitted to the adjudication committee to assess for possible pancreatitis or other pancreatic disease.

Asymptomatic elevation of pancreatic amylase and/or lipase

Serial measures of pancreatic enzymes have limited clinical value for predicting episodes of acute pancreatitis in asymptomatic patients (Nauck et al. 2017; Steinberg et al. 2017a, 2017b). Therefore, further diagnostic follow-up of cases of asymptomatic elevation of pancreatic enzymes (lipase and/or p-amylase ≥3X ULN) is not mandated but may be performed based on the investigator’s clinical judgment and assessment of the participant’s overall clinical condition.

8.3.2.3. Thyroid Malignancies and C-Cell Hyperplasia

Individuals with personal or family history of MTC and/or MEN-2 will be excluded from the study. Participants who are diagnosed with MTC and/or MEN-2 during the study will have study drug stopped and should continue follow-up with an endocrinologist.

The assessment of thyroid safety during the trial will include reporting of any case of thyroid malignancy (including MTC, papillary carcinoma, and others) and measurements of calcitonin. These data will be captured in specific eCRFs. The purpose of calcitonin measurements is to assess the potential of tirzepatide to affect thyroid C-cell function, which may indicate development of C-cell hyperplasia and neoplasms.

8.3.2.4. Calcitonin Measurements

If an increased calcitonin value (see definitions below) is observed in a participant who has been administered a medication that is known to increase serum calcitonin, then this medication should be stopped, and calcitonin levels should be measured after an appropriate washout period.

For participants who require additional endocrine assessment because of increased calcitonin concentration as defined in this section, data from the follow-up assessment will be collected in the specific section of the eCRF.

Calcitonin Measurements in Participants with eGFR ≥60 mL/min/1.73 m^2

A significant increase in calcitonin for participants with eGFR ≥60 mL/min/1.73 m^2 is defined below. If a participant's laboratory results meet these criteria, these clinically significant laboratory results should be recorded as an AE.

- *Serum calcitonin value ≥20 ng/L and <35 ng/L AND ≥50% increase from the screening value.* These participants will be asked to repeat the measurement within 1 month. If this repeat value is increasing (≥10% increase), the study drug should be stopped, and the participant encouraged to undergo additional endocrine assessment and longer term, follow-up by an endocrinologist to exclude any serious adverse effect on the thyroid.

- *Serum calcitonin value ≥35 ng/L AND ≥50% over the screening value*. In these participants, study drug should be stopped, and the participant recommended to immediately undergo additional endocrine assessments and longer term, follow-up by an endocrinologist.

Calcitonin Measurement in Participants with eGFR <60 mL/min/1.73 m^2

A significant increase in calcitonin for participants with eGFR <60 mL/min/1.73 m^2 is defined as a *serum calcitonin value ≥35 ng/L AND ≥50% over the screening value.* If a participant's labs meet these criteria, these clinically significant labs should be recorded as an AE.

In these participants, study drug should be discontinued (after first confirming the value) and the participant recommended to immediately undergo additional endocrine assessments and longer-term follow-up by an endocrinologist to exclude any serious adverse effect on the thyroid.

8.3.2.5. Major Adverse Cardiovascular Events

Deaths and nonfatal cardiovascular AEs will be adjudicated by a committee of physicians external to Lilly with cardiology expertise. This committee will be blinded to treatment assignment. The nonfatal cardiovascular AEs to be adjudicated include:

- myocardial infarction
- hospitalization for unstable angina
- hospitalization for heart failure
- coronary interventions (such as coronary artery bypass graft or percutaneous coronary intervention), and

- cerebrovascular events, including cerebrovascular accident (stroke) and transient ischemic attack.

8.3.2.6. Supraventricular Arrhythmias and Cardiac Conduction Disorders

Treatment-emergent cardiac conduction disorders will be further evaluated. Participants who develop any event from these groups of disorders should undergo an ECG, which should be submitted to the central reading center. Additional diagnostic tests to determine exact diagnosis should be performed, as needed. The specific diagnosis will be recorded as an AE. Events that meet criteria for serious conditions as described in Section 10.4 must be reported as SAEs.

8.3.2.7. Hypersensitivity Reactions

Many drugs, but particularly biologic agents, carry the risk of systemic hypersensitivity reactions. If such a reaction occurs, additional data describing each symptom should be provided to the sponsor in the eCRF.

Sites should have appropriately trained medical staff and appropriate medical equipment available when study participants are receiving study drug. It is recommended that participants who experience a systemic hypersensitivity reaction be treated per national and international guidelines.

In the case of generalized urticaria or anaphylaxis, additional blood and urine samples should be collected as described in Section 10.3 Appendix 3 "Recommended Laboratory Testing for Hypersensitivity Events". Laboratory results are provided to the sponsor via the central laboratory.

8.3.2.8. Injection Site Reactions

Symptoms of a local injection site reaction may include erythema, induration, pain, pruritus, and edema. If an injection site event is reported, the AE will be recorded, and additional data will be provided to the sponsor in the Injection Site Reaction eCRF.

At the time of AE occurrence, samples will be collected for measurement of tirzepatide antidrug antibody (ADA) and tirzepatide concentration.

8.3.2.9. Antidrug Antibodies

The occurrence of ADA formation will be assessed as outlined in Section 8.9.

8.3.2.10. Hepatobiliary Disorders

All events of TE biliary colic, cholecystitis, or other suspected events related to gallbladder disease should be evaluated and additional diagnostic tests performed, as needed. In cases of elevated liver markers, hepatic monitoring should be initiated as outlined in Section 8.2.5.1.

8.3.2.11. Severe Gastrointestinal Adverse Events

Tirzepatide may cause severe GI AEs, such as nausea, vomiting, and diarrhea. Information about severe GI AEs as well as antiemetic/antidiarrheal use will be collected in the eCRF/AE form. For detailed information concerning the management of GI AEs, please refer to Section 6.6.2.

8.3.2.12. Acute Renal Events

Renal safety will be assessed based on repeated renal function assessment as well as assessment of AEs suggestive of acute or worsening of chronic renal failure. Gastrointestinal AEs have been reported with tirzepatide, including nausea, diarrhea, and vomiting. This is consistent with other GLP-1R agonists (Aroda and Ratner 2011). The events may lead to dehydration, which could cause a deterioration in renal function, including acute renal failure. Participants should be advised to notify investigators in case of severe nausea, frequent vomiting, or symptoms of dehydration.

8.3.2.13. Depression, Suicidal Ideation or Behavior Monitoring

Participants will be monitored for depression and suicidal ideation or behavior through AE collection and by using the C-SSRS and the PHQ-9. Participants will be referred to a MHP if in the opinion of the investigator it is necessary for the safety of the participant or if the participant had any of the following:

- a PHQ-9 score ≥15
- C-SSRS responses of:
  - A “yes” answer to Question 4 (Active Suicidal Ideation with Some Intent to Act, Without Specific Plan) on the “Suicidal Ideation” portion of the C-SSRS

    or

  - A “yes” answer to Question 5 (Active Suicidal Ideation with Specific Plan and Intent) on the “Suicidal Ideation” portion of the C-SSRS

    or

  - A “yes” answer to any of the suicide-related behaviors (actual attempt, interrupted attempt, aborted attempt, preparatory act, or behavior) on the “Suicidal Behavior” portion of the C-SSRS

8.3.2.14. Amputation/Peripheral Revascularization

All cases of amputation and peripheral revascularization should be reported as an AE.

8.3.2.15. Metabolic Acidosis, Including Diabetic Ketoacidosis

Ketoacidosis, a serious life-threatening condition requiring urgent hospitalization, has been reported rarely in patients with T2DM. Participants who present with signs and symptoms consistent with severe metabolic acidosis should be assessed for ketoacidosis regardless of presenting BG levels, as ketoacidosis may be present even if BG levels are less than 250 mg/dL. If ketoacidosis is suspected, SGLT-2 inhibitor should be discontinued (if used), patients should be evaluated, and prompt treatment should be instituted. Treatment of ketoacidosis will require insulin, and carbohydrate replacement.

Lactic acidosis is a risk for participants with T2DM who excessively drink alcohol, have impaired kidney function (eGFR <30 ml/min/m^2), and are on metformin therapy. Routine bicarbonate assessment will be performed during the course of the study. If lactic acidosis is

suspected, metformin should temporarily be discontinued (if used) until the resolution of the event.

8.3.2.16. Diabetic Retinopathy Complications

Dilated retinal fundoscopic exam will be performed by an eye care professional (ophthalmologist or optometrist) for all patients between Visit 2 and Visit 3 to exclude participants with proliferative diabetic retinopathy, diabetic macular edema, or nonproliferative diabetic retinopathy that requires acute treatment.

The results from this exam will be recorded on a specific eCRF (Fundoscopy Exam) as a baseline measure of retinopathy. A follow-up, dilated fundoscopic exam should be performed when clinically indicated by any AE suspected of worsening retinopathy, and the findings should be recorded on the specific eCRF page.

8.4. Treatment of Overdose

Study drug overdose (more than the specified number of injections) will be reported as an AE. In the event of overdose, refer to the IB for tirzepatide.

8.5. Pharmacokinetics

Pharmacokinetic samples will be collected from approximately the first 300 randomized participants, approximately 100 participants from each treatment arm.

A separate written consent for the PK blood draws will be obtained at the time of the initial trial consent, where applicable.

Plasma tirzepatide concentrations will only be determined from blood samples obtained from participants receiving tirzepatide treatment. Blood samples for PK assessment will be collected at Weeks 8, 16, and 36 following tirzepatide treatment per the SoA. At each visit prior to a PK assessment, each participant will be assigned via IWRS to 1 of the sampling PK time windows of 1 to 24 hours, 24 to 96 hours, or 120 to 168 hours postdose.

The date and time of the most recent tirzepatide SC injection administered prior to collecting the PK sample must be recorded on the eCRF from the study diaries.

The date and time at which each sample was drawn must be recorded on the laboratory accession page.

Concentrations of tirzepatide will be assayed using a validated liquid chromatography mass spectrometry method. Bioanalytical samples collected to measure IP concentrations will be retained for a maximum of 1 year following last subject visit for the study. During this time, samples remaining after the bioanalyses may be used for exploratory analyses such as metabolism work, protein binding, and/or bioanalytical method cross-validation.

8.6. Pharmacodynamics

Samples to assess the PD properties of tirzepatide are included in the efficacy measures and not applicable in this section.

8.7. Genetics

A whole blood sample will be collected for pharmacogenetic analysis as specified in the SoA (Section 1.3) where local regulations allow.

Samples will not be used to conduct unspecified disease or population genetic research either now or in the future. Samples will be used to investigate variable response to tirzepatide and to investigate genetic variants thought to play a role in obesity. Assessment of variable response may include evaluation of AEs or differences in efficacy.

All samples will be coded with the participant number. These samples and any data generated can be linked back to the participant only by the investigator site personnel.

Samples will be retained at a facility selected by Lilly or its designee for a maximum of 15 years after the last participant visit for the study, or for a shorter period if local regulations and/or ethical review boards (ERBs)/institutional review boards (IRBs) impose shorter time limits. This retention period enables use of new technologies, response to regulatory questions, and investigation of variable response that may not be observed until later in the development of tirzepatide or after tirzepatide become(s) commercially available.

Molecular technologies are expected to improve during the 15-year storage period and therefore cannot be specifically named. However, existing approaches include whole genome or exome sequencing, genome wide association studies, and candidate gene studies. Regardless of technology, utilized genotyping data generated will be used only for the specific research scope described in this section.

8.8. Biomarkers

Biomarker research is performed to address questions of relevance to drug disposition, target engagement, PD, mechanism of action, variability of participant response (including safety), and clinical outcome. Sample collection is incorporated into clinical studies to enable examination of these questions through measurement of biomolecules including deoxyribonucleic acid (DNA), proteins, lipids, and other cellular elements.

Serum and plasma samples for biomarker research (nonpharmacogenetic stored sample) will be collected at the times specified in the SoA (Section 1.3) where local regulations allow.

Samples will be used for research on the drug target, disease process, variable response to tirzepatide, pathways associated with obesity, mechanism of action of tirzepatide, and/or research method or in validating diagnostic tools or assay(s) related to obesity.

All samples will be coded with the participant number. These samples and any data generated can be linked back to the participant only by the investigator site personnel.

Samples will be retained at a facility selected by Lilly or its designee for a maximum 15 years after the last participant visit for the study, or for a shorter period if local regulations and ERBs impose shorter time limits. This retention period enables use of new technologies, response to regulatory questions, and investigation of variable response that may not be observed until later in the development of tirzepatide or after tirzepatide becomes commercially available.

8.9. Immunogenicity Assessments

Where local regulations and ERBs allow, blood samples for immunogenicity testing will be collected to determine antibody production against tirzepatide as specified in the SoA (Section 1.3).

For immunogenicity testing, venous blood samples will be collected from each participant according to the SoA (Section 1.3) to determine antibody production against tirzepatide. To interpret the results of immunogenicity, a venous blood sample will be collected at the same time points to determine the plasma concentrations of tirzepatide. All samples for immunogenicity should be taken predose when applicable and possible.

In the event of systemic drug hypersensitivity reactions (immediate or nonimmediate), additional unscheduled samples should be collected as detailed in Section 8.3.2.7 (Hypersensitivity Reactions). Instructions for the collection and handling of blood samples will be provided by the sponsor. The actual date and time (24-hour clock time) of each sampling will be recorded. Sample collected at Visit 801 will assess immunogenicity at washout of tirzepatide (5 half-lives post end of treatment).

Immunogenicity will be assessed by a validated assay designed to detect ADAs in the presence of tirzepatide at a laboratory approved by the sponsor. Antibodies may be further characterized for their ability to neutralize the activity of tirzepatide on GIP and GLP-1Rs. Positive tirzepatide ADA samples will be tested for cross-reactivity with native GIP and GLP-1, and, if positive, may then be tested for neutralizing antibodies against native GIP and/or GLP-1. In vivo laboratory indicators for effect on weight loss and PK will be utilized to detect potential neutralizing effect of ADAs against tirzepatide.

Treatment-emergent ADAs are defined in Section 9.4.6.

Samples will be retained for a maximum of 15 years after the last participant visit, or for a shorter period if local regulations and ERBs allow, at a facility selected by the sponsor. The duration allows the sponsor to respond to future regulatory requests related to tirzepatide. Any samples remaining after 15 years will be destroyed.

Concentrations of tirzepatide will be assayed using a validated liquid chromatography mass spectrometry method. Bioanalytical samples collected to measure tirzepatide concentrations will be retained for a maximum of 1 year following last subject visit for the study. During this time, samples remaining after the bioanalyses may be used for exploratory analyses such as metabolism work, protein binding, and/or bioanalytical method cross-validation.

8.10. Health Economics

Health Economics parameters are not evaluated in this study.

9. Statistical Considerations

9.1. Statistical Hypotheses

The alternative hypotheses for the primary objective are the following:

- $H_{10,1}$: QW tirzepatide 10 mg is superior to placebo for mean percent change in body weight from randomization AND proportion of participants who achieve ≥5% body weight reduction at 72 weeks.
- $H_{15,1}$: QW tirzepatide 15 mg is superior to placebo for percent change in body weight from randomization AND proportion of participants who achieve ≥5% body weight reduction at 72 weeks.

The above 2 hypotheses will be tested in parallel, each at a 2-sided significance level of 0.025.

The alternative hypotheses for the key secondary objective controlling for type 1 error rate are the following:

- $H_{10,2}$: QW tirzepatide 10 mg is superior to placebo for proportion of participants who achieve ≥10% body weight reduction at 72 weeks.
- $H_{15,2}$: QW tirzepatide 15 mg is superior to placebo for proportion of participants who achieve ≥10% body weight reduction at 72 weeks.
- $H_{10,3}$: QW tirzepatide 10 mg is superior to placebo for proportion of participants who achieve ≥15% body weight reduction at 72 weeks.
- $H_{15,3}$: QW tirzepatide 15 mg is superior to placebo for proportion of participants who achieve ≥15% body weight reduction at 72 weeks.
- $H_{10,4}$: QW tirzepatide 10 mg is superior to placebo for change from randomization in HbA1c (%) at 72 weeks.
- $H_{15,4}$: QW tirzepatide 15 mg is superior to placebo for change from randomization in HbA1c (%) at 72 weeks.
- $H_{10,5}$: QW tirzepatide 10 mg is superior to placebo for proportion of participants who achieve the target value of HbA1c <7% at 72 weeks.
- $H_{15,5}$: QW tirzepatide 15 mg is superior to placebo for proportion of participants who achieve the target value of HbA1c <7% at 72 weeks.
- $H_{10,6}$: QW tirzepatide 10 mg is superior to placebo for change from randomization in waist circumference (cm) at 72 weeks.
- $H_{15,6}$: QW tirzepatide 15 mg is superior to placebo for change from randomization in waist circumference (cm) at 72 weeks.

- $H_{10,7}$: QW tirzepatide 10 mg is superior to placebo for change from randomization in Fasting Glucose (mg/dL) at 72 weeks.
- $H_{15,7}$: QW tirzepatide 15 mg is superior to placebo for change from randomization in Fasting Glucose (mg/dL) at 72 weeks.
- $H_{p,8}$: tirzepatide (all tirzepatide doses combined) is superior to placebo for change in triglycerides (mg/dL) from randomization at 72 weeks.
- $H_{p,9}$: tirzepatide (all doses combined) is superior to placebo for change in LDL cholesterol (mg/dL) from randomization at 72 weeks.
- $H_{p,10}$: tirzepatide (all doses combined) is superior to placebo for change in total cholesterol (mg/dL) from randomization at 72 weeks.
- $H_{p,11}$: tirzepatide (all doses combined) is superior to placebo for change in SBP (mmHg) from randomization at 72 weeks.

The details of type 1 error rate control strategy for the above key secondary objectives will be provided in the statistical analysis plan (SAP).

9.2. Sample Size Determination

Approximately 1300 participants will be screened to achieve 900 randomly assigned to study intervention (300 participants per intervention group).

The sample size determination assumes that evaluation of superiority of tirzepatide 10 mg and tirzepatide 15 mg to placebo will be conducted in parallel, each at a 2-sided significance level of 0.025 using a 2-sample t-test. Additionally, a difference of at least 11% mean body weight percentage reduction from randomization at 72 weeks for tirzepatide 10 mg and/or tirzepatide 15 mg compared to placebo, a common SD of 10%, and a dropout rate of 25% are assumed for statistical power calculations. Under the assumptions above, randomizing 900 participants in a 1:1:1 ratio to tirzepatide 10 mg (300), tirzepatide 15 mg (300), and placebo (300) provides more than 90% power to demonstrate superiority of each tirzepatide dose to placebo.

The chosen sample size and randomization ratio also provides >90% power to establish superiority of 10 mg tirzepatide and 15 mg tirzepatide dose to placebo in terms of proportion of participants achieving at least 5% body weight reduction at 72 weeks, conducted in parallel using a Chi-square test, each at a 2-sided significance level of 0.025, assuming 25% placebo treated participants and 90% tirzepatide treated participants achieving the goal and a dropout rate of 25%.

9.3. Populations for Analyses

For purposes of analysis, the following analysis sets are defined:

Analysis Set	Description
Entered Participants	All participants who sign informed consent
Randomized Participants	All participants who are randomly assigned a study treatment
Modified Intent-to-Treat (mITT)	All randomly assigned participants who are exposed to at least 1 dose of study drug. Participants will be included in the treatment group they were randomized

	to.
Efficacy Analysis Set (EAS)	Data obtained during treatment period from mITT, excluding data after initiating rescue antihyperglycemic medication or stopping study drug prematurely (last dose + 7 days).
Full Analysis Set (FAS)	Data obtained during treatment period from mITT, regardless of adherence to study drug or initiation of rescue antihyperglycemic medication.
Safety Analysis Set (SS)	Data obtained during the treatment period plus safety follow up from mITT, regardless of adherence to study drug or initiation of rescue antihyperglycemic medication.

9.4. Statistical Analyses

9.4.1. General Considerations

Statistical analysis of this study will be the responsibility of Lilly or its designee. Any change to the data analysis methods described in the protocol will require an amendment ONLY if it changes a principal feature of the protocol. Any other change to the data analysis methods described in the protocol, and the justification for making the change, will be described in the SAP or the clinical study report (CSR). Additional exploratory data analyses may be conducted as deemed appropriate.

Unless otherwise noted, all tests of treatment effects will be conducted at a 2-sided alpha level of 0.05, and the confidence interval will be given at a 2-sided 95% level. In statistical summaries and analyses, data will be analyzed as randomized.

Unless specified otherwise, efficacy analyses will be conducted using efficacy analysis set (EAS) and safety analyses will be conducted using safety analysis set (SS). Baseline is defined as the last nonmissing data collected prior to or at randomization.

Summary statistics for continuous measures will include sample size, mean, SD, median, minimum, and maximum. The analysis model to make comparisons among treatment groups relative to continuous measurements assessed over time will be a mixed model for repeated measures (MMRM), with terms of treatment, visit, treatment-by-visit interaction, stratification factors and baseline measurement as a covariate. An unstructured covariance structure will model relationship of within-patient errors.

Kaplan-Meier method will be used for estimation of cumulative event-free survival rates over time, and cox proportional hazards regression analysis will be used to compare hazard rates among treatments.

Summary statistics for categorical measures (including categorized continuous measures) will include sample size, frequency, and percentages. Logistic regression will be used to examine the treatment difference in binary efficacy outcomes if there is a need to adjust for covariates. Otherwise, Fisher's exact test will be used to examine the treatment difference in categorical outcomes.

Summary statistics for discrete count measures will include sample size, mean, SD, median, minimum, and maximum. The negative binomial regression model will be used for the treatment comparison of discrete count measures.

Other statistical methods may be used, as appropriate, and details will be described in the SAP.

9.4.2. Treatment Group Comparability

9.4.2.1. Participant Disposition

A detailed description of participant disposition will be provided at the end of the study.

Frequency counts and percentages of all participants screened, randomized, and receiving at least 1 dose of study drug will be presented by treatment groups. Of the randomized population, frequency counts and percentages of participants who completed the study or prematurely discontinued the study (and/or study drug), including reason for premature discontinuation, will be presented by treatment groups.

A Kaplan-Meier analysis of time from randomization to premature discontinuation from study and/or treatment by treatment group will be provided.

9.4.2.2. Participant Characteristics

Demographics and other baseline characteristics (including years of education) will be summarized by treatment group for all randomized participants.

9.4.2.3. Concomitant Therapy

Concomitant medications, including previous therapy, will be summarized by treatment arm for FAS.

9.4.2.4. Treatment Compliance

Frequency counts and percentages of participants compliant to study drug will be summarized by treatment arms and visits for FAS.

9.4.3. Efficacy Analyses

9.4.3.1. Primary Analyses

There will be 2 estimands of interest in comparing efficacy of tirzepatide doses with placebo.

For the FDA, the primary efficacy analysis will be guided by the "hybrid" estimand and conducted using the FAS. This assessment will analyze percent change in body weight obtained at 72 weeks visit using an analysis of covariance (ANCOVA) and the proportion of participants achieving at least 5% body weight reduction obtained at 72 weeks visit using logistic regression model. Both models (ANCOVA and logistic regression model) will include terms of treatment, stratification factors, and baseline body weight as a covariate. Missing data of the primary outcome measurement due to all other Intercurrent Events (ICEs) will be imputed based on retrieved dropouts in the same treatment arm, defined as observed primary outcome measurements from participants in the same treatment group who had their efficacy assessed after early discontinuation of study drug. In cases where there are not enough retrieved dropouts to provide a reliable imputation model (for example, the model implemented by the SAS program does not converge), an alternative multiple imputation method with reference to the placebo group (placebo multiple imputation) will be used. Analysis will be conducted with multiple imputations. Since the mean percent change in body weight and percentage of

participants with ≥5% body weight reduction needs to be achieved at the same time, no multiplicity adjustment is planned for these 2 tests.

For all other purposes, the primary efficacy assessment will be guided by the "efficacy" estimand, which represents efficacy prior to discontinuation of study drug without confounding effects of antihyperglycemic rescue therapy, and conducted using the EAS. The primary analysis model will be a MMRM for body weight percent change over time and longitudinal logistic regression for proportion of participants achieving at least 5% body weight reduction over time. The response variable of MMRM will be the percent change in body weight from randomization values obtained at each scheduled postbaseline visit. The response variable of longitudinal logistic regression will be the proportion of participants achieving at least 5% body weight reduction at each scheduled post-randomization visit. The independent variables of both analysis models are treatment group (tirzepatide 10 mg, tirzepatide 15 mg, and placebo), visit, and treatment-by-visit interaction, stratification factors, and baseline body weight as covariate. An unstructured covariance structure will model relationship of within-patient errors. The Kenward-Roger approximation will be used to estimate denominator degrees of freedom.

9.4.3.2. Key Secondary Analyses

- Superiority of each tirzepatide dose to placebo for the proportion of participants who achieve ≥10% body weight reduction at 72 weeks
- Superiority of each tirzepatide dose to placebo for the proportion of participants who achieve ≥15% body weight reduction at 72 weeks
- Superiority of each tirzepatide dose to placebo for the change from randomization in HbA1c (%) values at 72 weeks
- Superiority of each tirzepatide dose to placebo for the proportion of participants who achieve the target value of HbA1c <7% at 72 weeks
- Superiority of each tirzepatide dose to placebo for the change from randomization in waist circumference (cm) at 72 weeks
- Superiority of each tirzepatide dose to placebo for the change from randomization in fasting glucose (mg/dL) at 72 weeks
- Superiority of tirzepatide (all doses combined) to placebo for change from randomization in triglycerides (mg/dL) at 72 weeks visit
- Superiority of tirzepatide (all doses combined) to placebo for change from randomization in LDL cholesterol (mg/dL) at 72 weeks visit
- Superiority of tirzepatide (all doses combined) to placebo for change from randomization in total cholesterol (mg/dL) at 72 weeks visit
- Superiority of tirzepatide (all doses combined) to placebo for change from randomization in SBP (mmHg) at 72 weeks visit

Additional details, including analysis methods for key secondary endpoints and the strategy for controlling overall type 1 error rate at a 2-sided alpha of 0.05 of primary and key secondary endpoint evaluation, will be provided in the SAP.

9.4.4. Safety Analyses

Unless specified otherwise, safety assessments will be guided by an estimand comparing safety of tirzepatide doses with placebo irrespective of adherence to study drug or initiation of rescue AHM. Thus, safety analysis will be conducted using SS.

9.4.4.1. Study Drug Exposure

Exposure to each study treatment will be calculated for each participant and summarized by treatment group.

9.4.4.2. Adverse Events

Adverse events will be coded from the actual term using the Medical Dictionary for Regulatory Activities (MedDRA) and reported with preferred terms and system organ class. Counts and proportions of participants experiencing events will be reported for each treatment group, and Fisher's exact test will be used to compare the treatment groups.

The proportion of participants experiencing TEAE, SAE, and discontinuation due to AE will be summarized by treatment group.

9.4.4.3. Special Safety Topics

This section includes areas of interest whether due to observed safety findings, potential findings based on drug class, or agreed upon consultation with regulatory agencies for the reasons previously mentioned.

The following AEs are AEs of special interest (AESIs) for this study:

- Severe hypoglycemia
- Major adverse cardiovascular events (adjudicated). Includes, but not limited to cardiovascular death, nonfatal myocardial infarction, nonfatal stroke, and hospitalization for heart failure
- TE Supraventricular arrhythmias and Cardiac Conduction Disorders
- Hepatobiliary disorders. Includes biliary colic, cholecystitis, and other gallbladder disease
- Severe GI events
- Acute renal events
- MDD/Suicidal Behavior and Ideation
- Pancreatitis (adjudicated)
- C-Cell Hyperplasia and thyroid malignancies
- Allergic/Hypersensitivity Reactions. Includes injection site reactions and ADA formation
- Diabetic retinopathy complications
- Metabolic acidosis, including diabetic ketoacidosis
- Amputation/peripheral revascularization.

Summaries and analyses for incidence of AESIs will be provided by treatment. The details of analysis of AESI will be provided in SAP.

9.4.4.4. Other Adverse Event Assessments

9.4.4.4.1. Gastrointestinal Events

Summaries and analyses for incidence and severity of nausea, vomiting, and diarrhea will be provided by each treatment.

9.4.4.4.2. Events Related to Potential Abuse Liability

Summaries and analyses for incidence of potential abuse liability TEAEs will be provided by treatment. The details will be provided in SAP.

9.4.4.4.3. Depression, Suicidal Ideation and Behavior

In addition to the summary of TEAEs, suicidal ideation and behavior will be assessed by C-SSRS, and depression related symptoms will be assessed using PHQ-9.

The analysis details will be provided in the SAP.

9.4.4.4.4. Central Laboratory Measures, Vital Signs, and Electrocardiograms

Actual values and change from randomization to post-randomization values of central laboratory measures, vital signs, and selected ECG parameters will be summarized at each scheduled visit. Change from randomization to post-randomization value will be summarized for participants who have both a randomization and at least 1 post-randomization result.

The percentages of participants with TE abnormal, high, or low measures (including laboratory, vital, and ECG parameters) at any time will be summarized and compared between treatment groups using Fisher's exact test.

The analysis details will be provided in SAP.

9.4.4.4.5. Hypoglycemic Events

Incidence and rate of documented symptomatic hypoglycemic events and severe hypoglycemia will be summarized and compared between tirzepatide doses and placebo.

9.4.5. Pharmacokinetic/Pharmacodynamic Analyses

Tirzepatide concentration data will be analyzed using a population PK approach via nonlinear mixed-effects modeling with the NONMEM® software. The relationship between tirzepatide dose and/or concentration and efficacy, tolerability, and safety endpoints will be characterized. Additionally, the impact of intrinsic and extrinsic participant factors such as age, weight, sex, and renal function on PK and/or PD parameters may be examined as needed. If ADA titers are detected from immunogenicity testing, then the impact of immunogenicity titers on tirzepatide PK or any relevant PD parameters may also be examined.

9.4.6. Evaluation of Immunogenicity

The frequency and percentage of participants with preexisting ADA and with treatment-emergent antidrug antibody positive (TE ADA+) to tirzepatide will be tabulated. Treatment-emergent ADAs are defined as those with a titer 2-fold (1 dilution) greater than the minimum required dilution (1:10) of the ADA assay if no ADAs were detected at baseline (treatment-induced ADA) or those with a 4-fold (2 dilutions) increase in titer compared with

baseline if ADAs were detected at baseline (treatment-boosted ADA). For the TE ADA+ participants, the distribution of maximum titers will be described. The frequency of neutralizing antibodies, if assessed, and cross reactivity to native GIP and GLP-1 may also be tabulated in TE ADA+ participants.

The relationship between the presence of antibodies and the PK parameters and PD response including safety and efficacy to tirzepatide may be assessed.

9.4.7. Other Analyses

9.4.7.1. Health Outcomes

Analyses of actual and change from randomization in the domains and/or summary scores of PROs questionnaires will be conducted using EAS. The details on questionnaire-specific analyses will be provided in the SAP.

9.4.7.2. Subgroup Analyses for Primary Analysis

Details of the subgroup analyses will be shown in the SAP.

The following subgroup variables will be considered (but not limited to):

- Age (<65 years and ≥65 years)
- Sex (female and male)
- Baseline BMI (<30, ≥30 and <35, ≥35 and <40, ≥40 kg/m^2)
- Race
- Ethnicity
- Type of antihyperglycemic agent used at randomization (associated weight gain, weight loss, and weight neutral)
- Baseline HbA1c (≤8.5% [≤69 mmol/mol] and >8.5% [>69 mmol/mol])

The outcome measures for the subgroup analyses will include:

- percent change in body weight from randomization at 72 weeks
- proportion of participants achieving at least 5% body weight reduction at 72 weeks.

9.5. Interim Analyses

No interim analyses are planned for this study. If an unplanned interim analysis is deemed necessary for reasons other than a safety concern, the protocol must be amended.

9.6. Data Monitoring Committee

Not applicable.

10. Supporting Documentation and Operational Considerations

10.1. Appendix 1: Regulatory, Ethical, and Study Oversight Considerations

10.1.1. Regulatory and Ethical Considerations

- This study will be conducted in accordance with the protocol and with the following:
  - Consensus ethical principles derived from international guidelines including the Declaration of Helsinki and Council for International Organizations of Medical Sciences (CIOMS) International Ethical Guidelines
  - Applicable International Council for Harmonization of Technical Requirements for Pharmaceuticals for Human Use (ICH) GCP Guidelines
  - Applicable laws and regulations
- The protocol, protocol amendments, ICF, IB, and other relevant documents (for example, advertisements) must be submitted to an IRB/Independent Ethics Committees (IEC) by the investigator and reviewed and approved by the IRB/IEC before the study is initiated.
- Any amendments to the protocol will require health authority approval before implementation of changes made to the study design, except for changes necessary to eliminate an immediate hazard to study participants.
- Protocols and any substantial amendments to the protocol will require health authority approval prior to initiation except for changes necessary to eliminate an immediate hazard to study participants.
- The investigator will be responsible for the following:
  - Providing written summaries of the status of the study to the IRB/IEC annually or more frequently in accordance with the requirements, policies, and procedures established by the IRB/IEC
  - Notifying the IRB/IEC of SAEs or other significant safety findings as required by IRB/IEC procedures
  - Providing oversight of study conduct for participants under their responsibility and adherence to requirements of 21 Code of Federal Regulations (CFR), ICH guidelines, the IRB/IEC, European regulation 536/2014 for clinical studies (if applicable), and all other applicable local regulations

- Investigator sites are compensated for participation in the study as detailed in the Clinical Trial Agreement (CTA).

10.1.2. Informed Consent Process

- The investigator or his/her representative will explain the nature of the study, including the risks and benefits, to the participant or his/her legally authorized representative and answer all questions regarding the study.
- Participants must be informed that their participation is voluntary. Participants or their legally authorized representative will be required to sign a statement of informed consent that meets the requirements of 21 CFR 50, local regulations, ICH guidelines, Health Insurance Portability and Accountability Act (HIPAA) requirements, where applicable, and the IRB/IEC or study center.
- The medical record must include a statement that written informed consent was obtained before the participant was entered in the study and the date the written consent was obtained. The authorized person obtaining the informed consent must also sign the ICF.
- Participants must be reconsented to the most current version of the ICF(s) during their participation in the study.
- A copy of the ICF(s) must be provided to the participant or the participant's legally authorized representative and is kept on file.

10.1.3. Data Protection

- Participants will be assigned a unique identifier by the sponsor. Any participant records, datasets or tissue samples that are transferred to the sponsor will contain the identifier only; participant names or any information which would make the participant identifiable will not be transferred.
- The participant must be informed that his/her personal study-related data will be used by the sponsor in accordance with local data protection law. The level of disclosure must also be explained to the participant who will be required to give consent for his/her data to be used as described in the informed consent.
- The participant must be informed that his/her medical records may be examined by Clinical Quality Assurance auditors or other authorized personnel appointed by the sponsor, by appropriate IRB/IEC members, and by inspectors from regulatory authorities.
- The sponsor has processes in place to ensure data protection, information security, and data integrity. These processes include appropriate contingency plan(s) for appropriate and timely response in the event of a data security breach.

10.1.4. Committees Structure

Prospective adjudication of major adverse cardiovascular events and pancreatic AEs will be performed for this study. Sections 8.3.2.2, and 8.3.2.5 outlines additional information on pancreatic and cardiovascular adjudication committees.

10.1.5. Dissemination of Clinical Study Data

Required clinical trial registries (for example, ClinicalTrials.gov) will be updated with the results from registered clinical trials regardless of the research outcome in accordance with local laws and regulations.

All CSRs, amendments, and addenda will be submitted to external regulatory authorities, external partners (as applicable), and sites.

The publication policy for Study I8F-MC-GPHL is outlined in Section 10.1.9 and further described in the CTA.

10.1.6. Data Quality Assurance

- All participant data relating to the study will be recorded on printed or eCRF unless transmitted to the sponsor or designee electronically (for example, laboratory data). The investigator is responsible for verifying that data entries are accurate and correct by physically or electronically signing the CRF.
- The investigator must maintain accurate documentation (source data) that supports the information entered in the CRF.
- The investigator must permit study-related monitoring, audits, IRB/IEC review, and regulatory agency inspections and provide direct access to source data documents.
- Monitoring details describing strategy (for example, risk-based initiatives in operations and quality such as risk management and mitigation strategies and analytical risk-based monitoring), methods, responsibilities, and requirements, including handling of noncompliance issues and monitoring techniques, are provided in the Monitoring Plan.
- The sponsor or designee is responsible for the data management of this study, including quality checking of the data.
- The sponsor assumes accountability for actions delegated to other individuals (for example, contract research organizations).
- Study monitors will perform ongoing source data verification to confirm that data entered into the eCRF by authorized site personnel are accurate, complete, and verifiable from source documents; that the safety and rights of participants are being protected; and that the study is being conducted in accordance with the currently approved protocol and any other study agreements, ICH GCP, and all applicable regulatory requirements.

- Records and documents, including signed ICFs, pertaining to the conduct of this study must be retained by the investigator for the time period outlined in the CTA unless local regulations or institutional policies require a longer retention period. No records may be destroyed during the retention period without the written approval of the sponsor. No records may be transferred to another location or party without written notification to the sponsor.
- In addition, the sponsor or its representatives will periodically check a sample of the participant data recorded against source documents at the study site. The study may be audited by the sponsor or its representatives, and/or regulatory agencies at any time. Investigators will be given notice before an audit occurs.

Data Capture System

The investigator is responsible for ensuring the accuracy, completeness, legibility, and timeliness of the data reported to the sponsor.

An electronic data capture system (EDC) will be used in this study for the collection of CRF data. The investigator maintains a separate source for the data entered by the investigator or designee into the sponsor provided EDC system. The investigator is responsible for the identification of any data to be considered source and for the confirmation that data reported are accurate and complete by signing the eCRF.

Additionally, clinical outcome assessment (COA) data (participant-focused outcome instrument) and other data (for example, diary) will be collected by the participant, caregiver, or authorized study personnel, via a paper source document and will be transcribed by the authorized study personnel into the EDC system.

Data collected via the sponsor-provided data capture system(s) will be stored at third-parties. The investigator will have continuous access to the data during the study and until decommissioning of the data capture system(s). Prior to decommissioning, the investigator will receive an archival copy of pertinent data for retention.

Data managed by a central vendor, such as laboratory test data, will be stored electronically in the central vendor's database system. Data will subsequently be transferred from the central vendor to the sponsor data warehouse.

Data from complaint forms submitted to sponsor will be encoded and stored in the global PC management system.

10.1.7. Source Documents

- Source documents provide evidence for the existence of the participant and substantiate the integrity of the data collected. Source documents are filed at the investigator's site.
- Data reported on the CRF or entered in the eCRF that are transcribed from source documents must be consistent with the source documents or the discrepancies must be explained. The investigator may need to request previous medical records or transfer records, depending on the study. Also, current medical records must be available.

- Definition of what constitutes source data can be found in study training material

10.1.8. Study and Site Start and Closure

The study start date is the date on which the clinical study will be open for recruitment of participants.

The sponsor or designee reserves the right to close the study site or terminate the study at any time for any reason at the sole discretion of the sponsor. Study sites will be closed upon study completion. A study site is considered closed when all required documents and study supplies have been collected and a study-site closure visit has been performed.

The investigator may initiate study-site closure at any time, provided there is reasonable cause and sufficient notice is given in advance of the intended termination.

Reasons for the early closure of a study site by the sponsor or investigator may include but are not limited to:

- Failure of the investigator to comply with the protocol, the requirements of the IRB/IEC or local health authorities, the sponsor's procedures, or GCP guidelines
- Inadequate recruitment of participants by the investigator
- Discontinuation of further study intervention development.

If the study is prematurely terminated or suspended, the sponsor shall promptly inform the investigators, the IECs/IRBs, the regulatory authorities, and any contract research organization(s) used in the study of the reason for termination or suspension, as specified by the applicable regulatory requirements. The investigator shall promptly inform the participant and assures appropriate participant therapy and/or follow-up.

10.1.9. Publication Policy

In accordance with the sponsor's publication policy, the results of this study will be submitted for publication by a peer-reviewed journal:

- The sponsor will comply with the requirements for publication of study results.
- Authorship will be determined by mutual agreement and in line with International Committee of Medical Journal Editors authorship requirements.
- The publication policy for Study I8F-MC-GPHL is described in the CTA.

10.2. Appendix 2: Clinical Laboratory Tests

- The tests detailed below will be performed by a central lab unless designated as local in the SoA and in the table below.
- In circumstances where the sponsor approves local laboratory testing in lieu of central laboratory testing (in the table below), the local laboratory must be qualified in accordance with applicable local regulations.
- Protocol-specific requirements for inclusion or exclusion of participants are detailed in Section 5 of the protocol.
- Additional tests may be performed at any time during the study as determined necessary by the investigator or required by local regulations.
- Pregnancy testing (refer to Section 5.1 Inclusion Criteria for screening pregnancy criteria).
- Investigators must document their review of the laboratory safety results.
- Laboratory results that could unblind the study will not be reported to investigative sites or other blinded personnel and are denoted in the table below.

Clinical Laboratory Tests

**Hematology[a]**	**Clinical Chemistry[a]**
Hemoglobin	Bicarbonate
Hematocrit	Sodium
Erythrocyte count (RBC)	Potassium
Mean cell volume	Total bilirubin
Mean cell hemoglobin concentration	Direct bilirubin
Leukocytes (WBC)	Alkaline phosphatase
Neutrophils, segmented	Alanine aminotransferase (ALT)
Lymphocytes	Aspartate aminotransferase (AST)
Monocytes	Blood urea nitrogen (BUN)
Eosinophils	Creatinine
Basophils	Uric acid
Platelets	Calcium
	Glucose
**Urine Chemistries[a]**	Albumin
Albumin	Creatine kinase (CK)
Creatinine	
**Cystatin-C[a]**	**Hormones (females)**
	Pregnancy Test serum[a] and/or urine (local)
	Follicle-stimulating hormone (FSH)[a]
**HbA1c[a]**	
**Endocrine[a]**	
Calcitonin	**Pancreas (exocrine)[a]**
Thyroid-stimulating hormone (TSH)	Pancreatic amylase
	Lipase
**Nonpharmacogenetic Stored Samples[a,b]**	**Immunogenicity[a,b]**
Serum	Anti-tirzepatide antibodies
EDTA plasma	Anti-tirzepatide antibody neutralization
P800 plasma	Pharmacokinetic Sample for Immunogenicity
	**Lipid Panel[a]**
	Triglycerides
**Pharmacogenetics Sample[a,b]**	VLDL-C
Whole blood (EDTA)	HDL-C
	LDL-C[c]
	Total cholesterol
**Free Fatty Acids[a]**	**Calculations** [a]
**Insulin[a]**	eGFR (calculated by CKD-EPI equation)
**C-peptide[a]**	UACR

Abbreviations: CKD-EPI = Chronic Kidney Disease-Epidemiology; EDTA = ethylenediaminetetraacetic acid; eGFR = estimated glomerular filtration rate; HbA1c = hemoglobin A1c; HDL-C = high-density lipoprotein cholesterol; LDL-C = low-density lipoprotein cholesterol; RBC = red blood cells; UACR = urine albumin/creatinine ratio; VLDL-C = very low-density lipoprotein cholesterol; WBC = white blood cells.

[a] All tests will be performed by a Lilly-designated central laboratory, unless otherwise noted.

[b] Results will not be provided to the investigative sites.

[c] This value will be calculated. If triglycerides are >400 mg/dL, the direct LDL will be assayed.

10.3. Appendix 3: Laboratory Assessments for Hypersensitivity Events

- Laboratory assessments should be performed if the participant experiences generalized urticaria or if anaphylaxis is suspected.
- Collect sample after the participant has been stabilized, and within 1 to 2 hours of the event; however, samples may be obtained as late as 12 hours after the event as analytes can remain altered for an extended period of time. Record the time at which the sample was collected.
- Obtain a follow-up sample at the next regularly scheduled visit or after 4 weeks, whichever is later.

The table below summarizes the laboratory parameters that will be evaluated. These laboratory tests are bundled in the hypersensitivity laboratory testing kit.

Clinical Lab Tests for Hypersensitivity Events

**Hypersensitivity Tests**	**Notes**<br>Selected test may be obtained in the event of anaphylaxis or systemic allergic/hypersensitivity reactions.
Tirzepatide ADAs (immunogenicity/ADA)	Assayed by Lilly-designated laboratory. Results will not be provided to the investigative sites.
Tirzepatide concentrations (PK)	Assayed by Lilly-designated laboratory. Results will not be provided to the investigative sites.
Tryptase	Assayed by Lilly-designated laboratory. Results will not be provided to the investigative sites.<br>***Note***: If a tryptase sample is obtained more than 2 hours after the event (that is, within 2 to 12 hours), or is not obtained because more than 12 hours have lapsed since the event, obtain urine sample for N-methylhistamine testing. Note that for tryptase serum samples obtained within 2 to 12 hours of the event, urine N-methylhistamine testing is performed in addition to tryptase testing. Collect the first void urine sample following the event. Obtain a follow-up urine sample for N-methylhistamine testing at the next regularly scheduled visit or after 4 weeks, whichever is later.
N-methylhistamine	Assayed by Lilly-designated laboratory. Results will not be provided to the investigative sites.
Drug-specific IgE	Will be performed if a validated assay is available.<br>Assayed by Lilly-designated laboratory. Results will not be provided to the investigative sites.
Basophil activation test	Will be performed if a validated assay is available.<br>Assayed by Lilly-designated laboratory. Results will not be provided to the investigative sites.<br>***Note:*** The basophil activation test is an in vitro cell based assay that only requires a serum sample. It is a surrogate assay for drug specific IgE, but is not specific for IgE.
Complement (C3, C3a, and C5a)	Assayed by Lilly-designated laboratory. Results will not be provided to the investigative sites.
Cytokine panel (IL-6, IL-1β, IL-10)	Assayed by Lilly-designated laboratory. Results will not be provided to the investigative sites.

Abbreviations: ADA = antidrug antibody; IgE = immunoglobulin E; IL = interleukin; PK = pharmacokinetic.

10.4. Appendix 4: Adverse Events: Definitions and Procedures for Recording, Evaluating, Follow-up, and Reporting

10.4.1. Definition of AE

AE Definition
• An AE is any untoward medical occurrence in a participant administered a pharmaceutical product and which does not necessarily have a causal relationship with the study intervention. An AE can therefore be any unfavourable and unintended sign (including an abnormal laboratory finding), symptom, or disease (new or exacerbated) temporally associated with the use of a medicinal (investigational) product, whether or not related to the medicinal (investigational) product.

Events <u>Meeting</u> the AE Definition
• Any abnormal laboratory test results (hematology, clinical chemistry, or urinalysis) or other safety assessments (for example, ECG, radiological scans, vital signs measurements), including those that worsen from baseline, considered clinically significant in the medical and scientific judgment of the investigator (that is, not related to progression of underlying disease).<br>• Exacerbation of a chronic or intermittent pre-existing condition including either an increase in frequency and/or intensity of the condition.<br>• New conditions detected or diagnosed after study intervention administration even though they may have been present before the start of the study.<br>• Signs, symptoms, or the clinical sequelae of a suspected drug-drug interaction.<br>• Signs, symptoms, or the clinical sequelae of a suspected overdose of either study intervention or a concomitant medication.<br>• "Lack of efficacy" or "failure of expected pharmacological action" per se will not be reported as an AE or SAE. Such instances will be captured in the efficacy assessments. However, the signs, symptoms, and/or clinical sequelae resulting from lack of efficacy will be reported as AE or SAE if they fulfill the definition of an AE or SAE.

Events <u>NOT</u> Meeting the AE Definition
• Any clinically significant abnormal laboratory findings or other abnormal safety assessments which are associated with the underlying disease, unless judged by the investigator to be more severe than expected for the participant's condition.<br>• The disease/disorder being studied or expected progression, signs, or symptoms of the disease/disorder being studied, unless more severe than expected for the

participant's condition.<br>• Medical or surgical procedure (for example, endoscopy, appendectomy): the condition that leads to the procedure is the AE.<br>• Situations in which an untoward medical occurrence did not occur (social and/or convenience admission to a hospital).<br>• Anticipated day-to-day fluctuations of pre-existing disease(s) or condition(s) present or detected at the start of the study that do not worsen.

10.4.2. Definition of SAE

If an event is not an AE per definition above, then it cannot be an SAE even if serious conditions are met (for example, hospitalization for signs/symptoms of the disease under study, death due to progression of disease).

**SAE is defined as any untoward medical occurrence that, at any dose:**
**a. Results in death**
**b. Is life-threatening**<br>The term 'life-threatening' in the definition of 'serious' refers to an event in which the participant was at risk of death at the time of the event. It does not refer to an event, which hypothetically might have caused death, if it were more severe.
**c. Requires inpatient hospitalization or prolongation of existing hospitalization**<br>• In general, hospitalization signifies that the participant has been admitted to hospital for observation and/or treatment that would not have been appropriate in the physician's office or outpatient setting. Complications that occur during hospitalization are AEs. If a complication prolongs hospitalization or fulfills any other serious criteria, the event is serious. When in doubt as to whether "hospitalization" occurred or was necessary, the AE should be considered serious.<br>• Hospitalization for elective treatment of a pre-existing condition that did not worsen from baseline is not considered an AE.
**d. Results in persistent disability/incapacity**<br>• The term disability means a substantial disruption of a person's ability to conduct normal life functions.<br>• This definition is not intended to include experiences of relatively minor medical significance such as uncomplicated headache, nausea, vomiting, diarrhea, influenza, and accidental trauma (for example, sprained ankle) which may interfere with or prevent everyday life functions but do not constitute a substantial disruption.
**e. Is a congenital anomaly/birth defect**

	• Abnormal pregnancy outcomes (for example, spontaneous abortion, fetal death, stillbirth, congenital anomalies, ectopic pregnancy) are considered SAEs
**f.**	**Other situations:**<br>• Medical or scientific judgment should be exercised in deciding whether SAE reporting is appropriate in other situations such as important medical events that may not be immediately life-threatening or result in death or hospitalization but may jeopardize the participant or may require medical or surgical intervention to prevent 1 of the other outcomes listed in the above definition. These events should usually be considered serious.<br>• Examples of such events include invasive or malignant cancers, intensive treatment in an emergency room or at home for allergic bronchospasm, blood dyscrasias or convulsions that do not result in hospitalization, or development of drug dependency or drug abuse.
**g.**	Resulted in medical or surgical intervention to prevent life-threatening illness or injury or permanent impairment to a body structure or a body function.

10.4.3. Definition of Product Complaint

**Product Complaint**
• A product complaint is any written, electronic, or oral communication that alleges deficiencies related to the identity, quality, durability, reliability, safety, effectiveness, or performance of a study intervention. When the ability to use the study intervention safely is impacted, the following are also PCs:<br>o Deficiencies in labeling information, and<br>o Use errors for device or drug-device combination products due to ergonomic design elements of the product.<br>• Product complaints related to study interventions used in clinical trials are collected in order to ensure the safety of participants, monitor quality, and to facilitate process and product improvements.<br>• Investigators will instruct participants to contact the site as soon as possible if he or she has a product complaint or problem with the study intervention so that the situation can be assessed.<br>• An event may meet the definition of both a product complaint and an AE/SAE. In such cases, it should be reported as both a product complaint and as an AE/SAE.

10.4.4. Recording and Follow-Up of AE and/or SAE and Product Complaints

**AE, SAE and Product Complaint Recording**
• When an AE/SAE/product complaint occurs, it is the responsibility of the investigator to review all documentation (for example, hospital progress notes, laboratory reports, and diagnostics reports) related to the event.

• The investigator will then record all relevant AE/SAE/product complaint information in the participant's medical records, in accordance with the investigator's normal clinical practice. AE/SAE information is reported on the appropriate (e)CRF page and product complaint information is reported on the product complaint Form.<br><br>Note: An event may meet the definition of both a product complaint and an AE/SAE. In such cases, it should be reported as both a product complaint and as an AE/SAE.<br><br>• It is **not** acceptable for the investigator to send photocopies of the participant's medical records to sponsor or designee in lieu of completion of the (e)CRF page for AE/SAE and the Product Complaint form for product complaints.<br>• There may be instances when copies of medical records for certain cases are requested by sponsor or designee. In this case, all participant identifiers, with the exception of the participant number, will be redacted on the copies of the medical records before submission to sponsor or designee.<br>• The investigator will attempt to establish a diagnosis of the event based on signs, symptoms, and/or other clinical information. Whenever possible, the diagnosis (not the individual signs/symptoms) will be documented as the AE/SAE.
**Assessment of Intensity**
The investigator will make an assessment of intensity for each AE and SAE reported during the study and assign it to 1 of the following categories:<br><br>• Mild: A type of AE that is easily tolerated by the participant, causing usually transient and may require only minimal discomfort and treatment or therapeutic intervention. The event does not interfering generally interfere with everyday usual activities of daily living.<br>• Moderate: A type of AE that causes sufficient discomfort and is usually alleviated with additional specific therapeutic intervention. The event interferes with normal everyday usual activities of daily living, causing discomfort but poses no significant or permanent risk of harm to the research participant.<br>• Severe: A type of AE that prevents normal everyday interrupts usual activities of daily living, or significantly affects clinical status, or may require intensive therapeutic intervention. An AE that is assessed as severe should not be confused with a SAE. Severe is a category utilized for rating the intensity of an event; and both AEs and SAEs can be assessed as severe.<br><br>An event is defined as 'serious' when it meets at least 1 of the predefined outcomes as described in the definition of an SAE, NOT when it is rated as severe.

**Assessment of Causality**

• The investigator is obligated to assess the relationship between study intervention and each occurrence of each AE/SAE.<br>• A "reasonable possibility" of a relationship conveys that there are facts, evidence, and/or arguments to suggest a causal relationship, rather than a relationship cannot be ruled out.<br>• The investigator will use clinical judgment to determine the relationship.<br>• Alternative causes, such as underlying disease(s), concomitant therapy, and other risk factors, as well as the temporal relationship of the event to study intervention administration will be considered and investigated.<br>• The investigator will also consult the IB and/or Product Information, for marketed products, in his/her assessment.<br>• For each AE/SAE, the investigator **must** document in the medical notes that he/she has reviewed the AE/SAE and has provided an assessment of causality.<br>• There may be situations in which an SAE has occurred and the investigator has minimal information to include in the initial report to sponsor or designee. However, it is very important that the investigator always make an assessment of causality for every event before the initial transmission of the SAE data to sponsor or designee.<br>• The investigator may change his/her opinion of causality in light of follow-up information and send a SAE follow-up report with the updated causality assessment.<br>• The causality assessment is 1 of the criteria used when determining regulatory reporting requirements.

**Follow-up of AEs and SAEs**
• The investigator is obligated to perform or arrange for the conduct of supplemental measurements and/or evaluations as medically indicated or as requested by sponsor or designee to elucidate the nature and/or causality of the AE or SAE as fully as possible. This may include additional laboratory tests or investigations, histopathological examinations, or consultation with other health care professionals.<br>• If a participant dies during participation in the study or during a recognized follow-up period, the investigator will provide sponsor or designee with a copy of any postmortem findings including histopathology.

10.4.5. Reporting of SAEs

**SAE Reporting via an Electronic Data Collection Tool**
• The primary mechanism for reporting an SAE will be the electronic data collection tool.<br>• If the electronic system is unavailable, then the site will use the paper SAE data

collection tool (see next section) in order to report the event within 24 hours.<br>• The site will enter the SAE data into the electronic system as soon as it becomes available.<br>• After the study is completed at a given site, the electronic data collection tool will be taken off-line to prevent the entry of new data or changes to existing data.<br>• If a site receives a report of a new SAE from a study participant or receives updated data on a previously reported SAE after the electronic data collection tool has been taken off-line, then the site can report this information on a paper SAE form (see next section) or to the sponsor contact for SAE reporting by telephone.<br>• Contacts for SAE reporting can be found in study training material.

SAE Reporting via Paper CRF
• Facsimile transmission of the SAE paper CRF is the preferred method to transmit this information to the sponsor contact for SAE reporting.<br>• Initial notification via telephone does not replace the need for the investigator to complete and sign the SAE CRF pages within the designated reporting time frames.<br>• Contacts for SAE reporting can be found in study training material.

10.4.6. Regulatory Reporting Requirements

SAE Regulatory Reporting
• Prompt notification by the investigator to the sponsor of a SAE is essential so that legal obligations and ethical responsibilities towards the safety of participants and the safety of a study intervention under clinical investigation are met.<br>• The sponsor has a legal responsibility to notify both the local regulatory authority and other regulatory agencies about the safety of a study intervention under clinical investigation. The sponsor will comply with country-specific regulatory requirements relating to safety reporting to the regulatory authority, IRB/IEC, and investigators.<br>• An investigator who receives an investigator safety report describing a SAE or other specific safety information (for example, summary or listing of SAEs) from the sponsor will review and then file it along with the IB and will notify the IRB/IEC, if appropriate according to local requirements.

10.5. Appendix 5: Contraceptive Guidance and Collection of Pregnancy Information

Definitions

Woman of Childbearing Potential:

A woman is considered fertile following menarche and until becoming postmenopausal unless permanently sterile (see below).

If fertility is unclear (for example, amenorrhea in adolescents or athletes) and a menstrual cycle cannot be confirmed before first dose of study intervention, additional evaluation should be considered.

Women in the following categories are not considered women of childbearing potential:

Article I. Premenarchal

Article II. Premenopausal female with 1 of the following:

- Documented hysterectomy
- Documented bilateral salpingectomy
- Documented bilateral oophorectomy

For individuals with permanent infertility due to an alternate medical cause other than the above, (for example, Mullerian agenesis, androgen insensitivity), investigator discretion should be applied to determining study entry.

Note: Determination can come from the site personnel's: review of the participant's medical records, medical examination, or medical history interview.

Article III. Postmenopausal female:

- A postmenopausal state is defined as either:
  - A woman at least 40 years of age with an intact uterus, not on hormone therapy, who has cessation of menses for at least 1 year without an alternative medical cause, AND a FSH ≥40 mIU/mL; or
  - A woman 55 or older not on hormone therapy, who has had at least 12 months of spontaneous amenorrhea; or
  - A woman at least 55 years of age with a diagnosis of menopause prior to starting HRT.
- Females on HRT and whose menopausal status is in doubt will be required to use 1 of the non estrogen hormonal highly effective contraception methods if they wish to continue their HRT during the study. Otherwise, they must discontinue HRT to allow confirmation of postmenopausal status before study enrollment.

Contraception Guidance

Contraceptive use by men and women should be consistent with local regulations regarding the methods of contraception for those participating in clinical studies.

Two forms of effective contraception, where at least 1 form is highly effective, will be used. Effective contraception may be used as the second therapy. Barrier protection methods without concomitant use of a spermicide are not a reliable or acceptable method. The use of male and female condoms as a double barrier method is not considered acceptable due to the high failure rate when these methods are combined.

Highly Effective Methods of Contraception:

- Combined oral contraceptive pill and mini pill
- NuvaRing
- Implantable contraceptives
- Injectable contraceptives (such as Depo-Provera®)
- Intrauterine device (such as Mirena® and ParaGard®)
- Contraceptive patch – ONLY women <198 pounds or 90 kg
- Total abstinence (if this is their preferred and usual lifestyle) or in a same-sex relationship with no sexual relationship with males (as part of their preferred and usual lifestyle), and agrees to maintain this status throughout trial follow-up

Note: Periodic abstinence (for example, calendar, ovulation, symptothermal, and postovulation methods), declaration of abstinence just for the duration of a trial, and withdrawal are not acceptable methods of contraception.

- Vasectomy – for men in clinical studies

Note: Implantable contraceptives and injectable contraceptives (such as Depo Provera) are only permitted if started more than 18 months prior to screening. Participants should not start these methods of contraception after being enrolled in the study.

Effective Methods of Contraception (must use combination of 2 methods):

- Male condom with spermicide
- Female condom with spermicide
- Diaphragm with spermicide
- Cervical sponge
- Cervical cap with spermicide

Men, regardless of their fertility status, with nonpregnant WOCBP partners must agree to either remain abstinent (if this is their preferred and usual lifestyle) or use condoms plus 1 additional highly effective (less than 1% failure rate) method of contraception (such as combination oral contraceptives, implanted contraceptives, or intrauterine device) or effective method of

contraception (such as diaphragms with spermicide or cervical sponge) for the duration of the study and for 5 half-lives of study drug plus 90 days, which is approximately 4 months after the last injection. Periodic abstinence (for example, calendar, ovulation, symptothermal, and postovulation methods), declaration of abstinence just for the duration of a trial, and withdrawal are not acceptable methods of contraception.

Men with pregnant partners should use condoms during intercourse for the duration of the study and until the end of estimated relevant potential exposure in women of childbearing potential.

Men who are abstinent (if this is complete abstinence, as their preferred and usual lifestyle) or in a same-sex relationship (as part of their preferred and usual lifestyle) must agree to either remain abstinent or stay in a same-sex relationship without sexual relationships with females. In these situations, men are not required to use contraception.

Men should refrain from sperm donation for the duration of the study and for 5 half-lives of study drug plus 90 days after the last dose of study drug, corresponding to 4 months after the last injection.

Collection of Pregnancy Information

Male participants with partners who become pregnant

- The investigator will attempt to collect pregnancy information on any male participant's female partner who becomes pregnant while the male participant is in this study.
- After obtaining the necessary signed informed consent from the pregnant female partner directly, the investigator will record pregnancy information on the appropriate form and submit it to the sponsor within 24 hours of learning of the partner's pregnancy. The female partner will also be followed to determine the outcome of the pregnancy. Information on the status of the mother and child will be forwarded to the sponsor. Generally, the follow-up will be no longer than 6 to 8 weeks following the estimated delivery date. Any termination of the pregnancy will be reported regardless of fetal status (presence or absence of anomalies) or indication for the procedure.

Female Participants who become pregnant

- The investigator will collect pregnancy information on any female participant who becomes pregnant while participating in this study. The initial information will be recorded on the appropriate form and submitted to the sponsor within 24 hours of learning of a participant's pregnancy.

- The participant will be followed to determine the outcome of the pregnancy. The investigator will collect follow-up information on the participant and the neonate and the information will be forwarded to the sponsor. Generally, follow-up will not be required for longer than 6 to 8 weeks beyond the estimated delivery date. Any termination of pregnancy will be reported, regardless of fetal status (presence or absence of anomalies) or indication for the procedure.
- While pregnancy itself is not considered to be an AE or SAE, any pregnancy complication or elective termination of a pregnancy for medical reasons will be reported as an AE or SAE.
- A spontaneous abortion (occurring at <20 weeks gestational age) or still birth (occurring at >20 weeks gestational age) is always considered to be an SAE and will be reported as such.
- Any poststudy pregnancy related SAE considered reasonably related to the study intervention by the investigator will be reported to the sponsor as described in protocol Section 8.3.1. While the investigator is not obligated to actively seek this information in former study participants, he or she may learn of an SAE through spontaneous reporting.
- Any female participant who becomes pregnant while participating in the study will discontinue study intervention and be withdrawn from the study.

10.6. Appendix 6: Liver Safety: Suggested Actions and Follow-up Assessments

- For testing selected, analysis is required to be completed by the Lilly designated central laboratory, except for microbiology.
- Local testing may be performed <u>in addition to central testing</u> when required for immediate participant management.
- Results will be reported if a validated test or calculation is available.

Hepatic Evaluation Labs

**Hematology**	**Clinical Chemistry**
Hemoglobin	Total bilirubin
Hematocrit	Direct bilirubin
Erythrocytes (RBCs - red blood cells)	Alkaline phosphatase (ALP)
Leukocytes (WBCs - white blood cells)	Alanine aminotransferase (ALT)
Differential:	Aspartate aminotransferase (AST)
Neutrophils, segmented	Gamma-glutamyl transferase (GGT)
Lymphocytes	Creatine kinase (CK)
Monocytes	**Other Chemistry**
Basophils	Acetaminophen
Eosinophils	Acetaminophen protein adducts
Platelets	Alkaline phosphatase isoenzymes
Cell morphology (RBC and WBC)	Ceruloplasmin
**Coagulation**	Copper
Prothrombin Time, INR (PT-INR)	Ethyl Alcohol
**Serology**	Haptoglobin
Hepatitis A Virus (HAV) testing:	Immunoglobulin IgA (Quantitative)
HAV total antibody	Immunoglobulin IgG (Quantitative)
HAV IgM antibody	Immunoglobulin IgM (Quantitative)
Hepatitis B Virus (HBV) Testing:	Phosphatidylethanol (PEth)
Hepatitis B surface antigen (HBsAg)	**Urine Chemistry**
Hepatitis B surface antibody (Anti-HBs)	Drug Screen
Hepatitis B core total antibody (Anti-HBc)	Ethyl glucuronide (EtG)
Hepatitis B core IgM antibody	**Other Serology**
Hepatitis B core IgG antibody	Anti-nuclear antibody (ANA)

HBV DNA[a]	Anti-smooth muscle antibody (ASMA) [b]
Hepatis C Virus (HCV) Testing:	Anti-actin antibody[c]
HCV antibody	Epstein-Barr Virus (EBV) Testing:
HCV RNA[a]	EBV antibody
Hepatitis D Virus (HDV) Testing:	EBV DNA[a]
HDV antibody	Cytomegalovirus (CMV) Testing:
Hepatitis E Virus (HEV) Testing:	CMV antibody
HEV IgG antibody	CMV DNA[a]
HEV IgM antibody	Herpes Simplex Virus (HSV) Testing:
HEV RNA[a]	HSV (Type 1 and 2) antibody
**Microbiology**[d]	HSV (Type 1 and 2) DNA[a]
Culture:	Liver Kidney Microsomal Type 1 (LKM-1) Antibody
Blood	
Urine	

Abbreviations: Ig = immunoglobulin; INR = international normalized ratio; PT = prothrombin time.

a Reflex/confirmation dependent on regulatory requirements, testing availability, or both.

b This is not required if anti-actin antibody is tested.

c This is not required if anti-smooth muscle antibody is tested.

d Assayed by investigator-designated local laboratory ONLY. No central testing available.

10.7. Appendix 7: Protocol GPHL Standardized Protocols for the Measurement of Height, Weight, Waist Circumference, Vital Signs, and Electrocardiogram

The following information has been adapted from standardized physical measurement protocols for the WHO's STEPwise approach to Surveillance (STEPS) (WHO 2017).

Measuring Height

Step 1. Ask the participant to remove their footwear and any headgear (light headgear worn for religious reasons can remain, but this should be worn by the participant at every clinic visit when their height is measured).

Step 2. Ask the participant to stand on the calibrated height measuring board (stadiometer) or against a wall with their feet together and their knees straight with their heels against the backboard, the stadiometer, or the wall.

Step 3. Ask the participant to look straight ahead without tilting their head up.

Step 4. Ask the participant to breathe in and stand tall. Measure and record the participant's height in centimeters to 1 decimal place.

Measuring Weight

- Body weight measurements should be done in a consistent manner using a calibrated electronic scale capable of measuring weight in kilograms to 1 decimal place.
- All weights for a given participant should be measured using the same scale, whenever possible, at approximately the same time in the morning after evacuation of bladder contents.
- Body weight must be measured in fasting state. If the participant is not fasting, the participant should be called in for a new visit within the visit window to have the fasting body weight measured.

Step 1. Ask the participant to empty their pockets, remove their footwear, outerwear (coat, jacket, etc.), and any headgear (light headgear worn for religious reasons can remain, but this should be worn by the participant at every clinic visit when weight is measured).

Step 2. Make sure the scale is placed on a firm, flat, even surface (not on carpet, on a sloping surface, or a rough, uneven surface).

Step 3. Ask the participant to step onto the scale with 1 foot on each side of the scale.

Step 4. Ask the participant to stand still with arms by sides and then record weight in kilograms to the nearest one-tenth kilogram.

Measuring Waist Circumference

- Waist circumference should be measured in the horizontal plane and at the midpoint between the lower margin of the last palpable rib and the top of the iliac crest.

- Measurements should be taken at the end of a normal expiration using a nonstretchable measuring tape. The tape should lie flat against the skin without compressing the soft tissue.
- The waist circumference should be measured twice, rounded to the nearest 0.5 cm. The measuring tape should be removed between the 2 measurements. Both measurements will be recorded in the eCRF. If the difference between the 2 measurements exceeds 1 cm, this set of measurements should be discarded and the 2 measurements repeated.

Step 1: Ask the participant to wear little clothing (if available, garments could also be used).

Step 2: Ask the participant to stand with their feet close together, arms at their side, body weight evenly distributed.

Step 3: Ask the participant to relax and measure the participant's waist circumference.

Vital Sign Measurements

- Vital sign measurements (blood pressure and heart rate, measured by pulse) should be taken before obtaining an ECG tracing and before collection of blood samples for laboratory testing.
- The participant should sit quietly for 5 minutes before vital signs measurements are taken.
- For each parameter, 2 measurements will be taken using the same arm, preferably the nondominant arm.
- The recordings should be taken at least 1 minute apart. Each measurement of sitting pulse and BP needs to be recorded in the eCRF.
- BP must be taken with an automated blood pressure instrument.
- If blood pressure and pulse measurements are taken separately, pulse should be taken prior to blood pressure.

Note: In the event pulse measurement cannot be taken via an automated blood pressure instrument, the preferred location for measurement of pulse is the radial artery.

Electrocardiogram

- All digital ECGs will be obtained using centrally provided ECG machines and will be electronically transmitted to a designated central ECG laboratory.
- 12-lead ECGs should be obtained after the subject has rested in a supine position for at least 10 minutes.
- Electrocardiograms should be collected at least 30 minutes prior to collection of blood samples for laboratory testing, including PK samples.

10.8. Appendix 8: Suggested Visit Structure

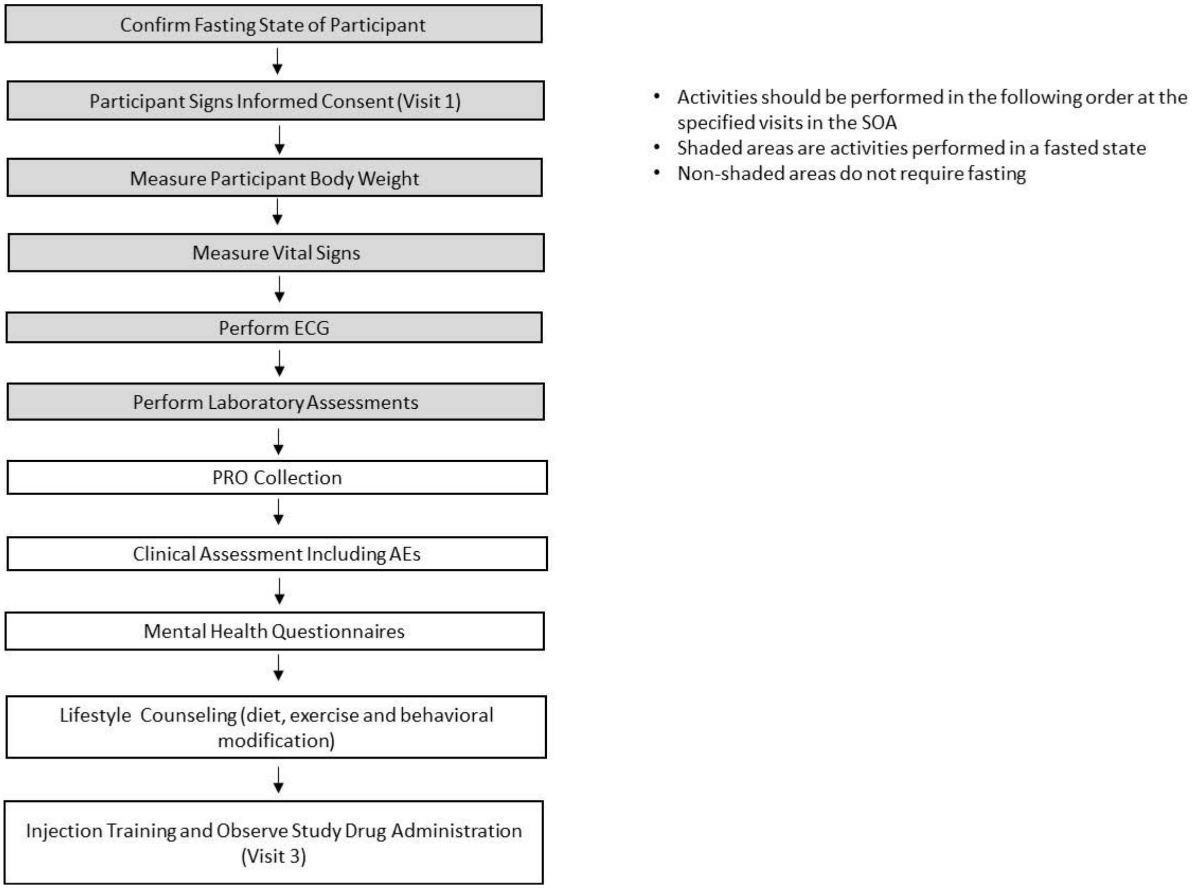

Abbreviations: AE = adverse event; ECG = electrocardiogram; PRO = patient reported outcome; SOA = schedule of activities.

10.9. Appendix 9: Management of Gastrointestinal Symptoms

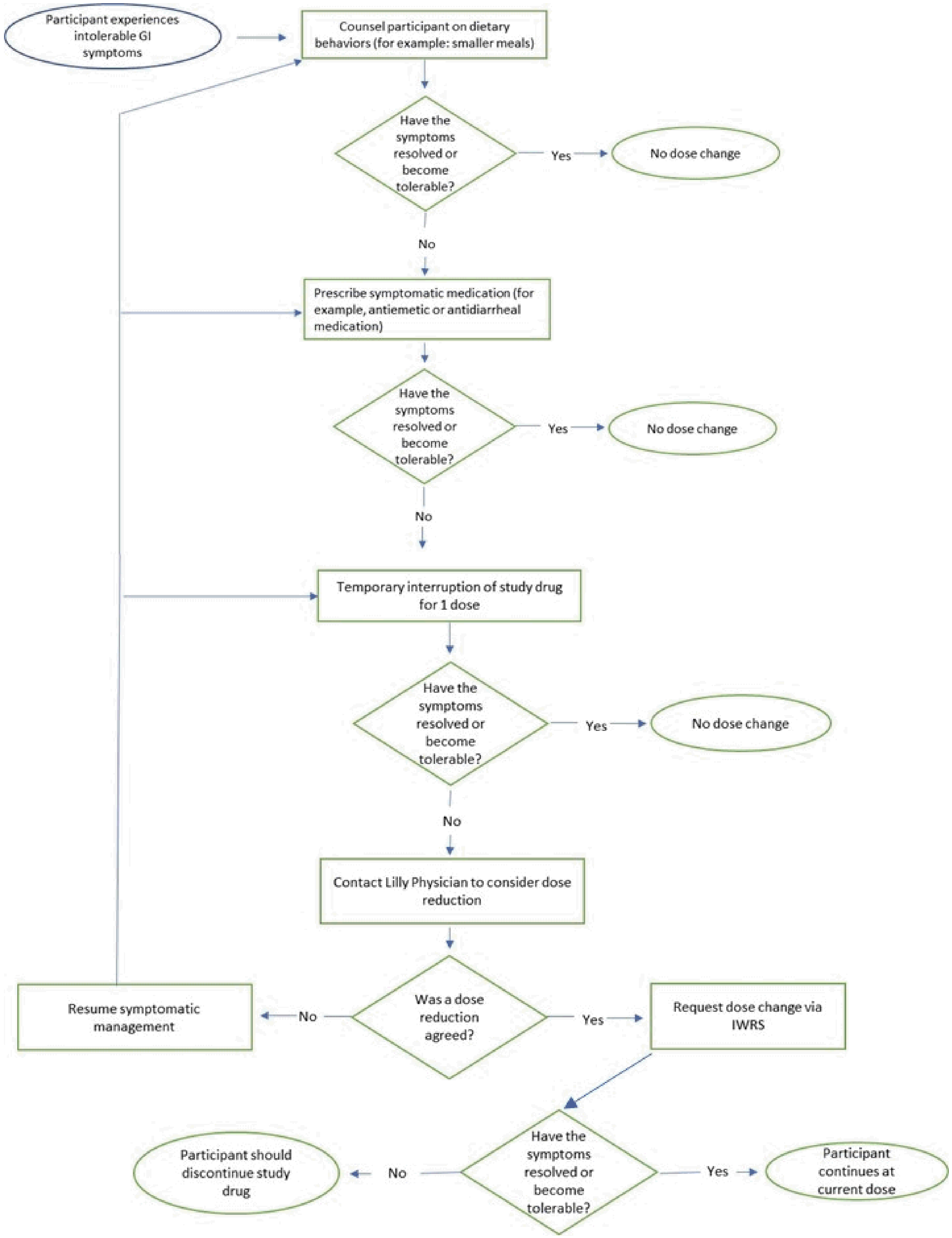

Abbreviations: GI = gastrointestinal; IWRS = interactive web-response system

10.10. Appendix 10: World Health Organization Classification of Diabetes and Diagnostic Criteria

Type 1 Diabetes: Type 1 diabetes is judged to be present when the classical symptoms of diabetes (thirst, polyuria, wasting and stupor, or coma) are associated with readily detectable concentrations of glucose and ketone bodies in the blood and urine. Insulin treatment is necessary not only to control hyperglycemia but also to prevent spontaneous ketosis and death.

Type 2 Diabetes: Type 2 diabetes, although often asymptomatic, may also present with classical hyperglycemic symptoms (thirst, polyuria, weight loss), but despite hyperglycemia, ketone bodies are present in only low concentrations in the blood and urine. Coma is rare in type 2 diabetes, but may result from extreme hyperglycemia and hyperosmolarity; lactic acidosis or ketoacidosis can also occur in fulminating illness (for example, severe infection or mesenteric artery thrombosis) due to an acute increase in insulin requirements, but spontaneous ketosis does not occur. Some patients with type 2 diabetes later progress to a state of absolute insulin deficiency (Alberti and Zimmet 1998).

10.11. Appendix 11: Provisions for Changes in Study Conduct During Exceptional Circumstances

Implementation of this appendix

The changes to procedures described in this appendix are temporary measures intended to be used only during specific time periods as directed by the sponsor in partnership with the investigator.

Exceptional circumstances

Exceptional circumstances are rare events that may cause disruptions to the conduct of the study. Examples include pandemics or natural disasters. These disruptions may limit the ability of the investigators, participants, or both to attend onsite visits or to conduct planned study procedures.

Implementing changes under exceptional circumstances

In an exceptional circumstance, after receiving the sponsor's written approval, sites may implement changes if permitted by local regulations.

After approval by local ERBs, regulatory bodies, and any other relevant local authorities, implementation of these exceptional circumstance changes will not typically require additional notification to these groups, unless they have specific conditions in which notification is required. To protect the safety of study participants, urgent changes may be implemented before approval but need to be reported as soon as possible. All approvals must be retained in the study records.

If the sponsor grants written approval for changes in study conduct, the sponsor will also provide additional written guidance, if needed.

Considerations for making a change

The prevailing consideration for making a change is ensuring the safety of study participants. Additional important considerations for making a change are compliance with GCP, enabling participants to continue safely in the study and maintaining the integrity of the study.

Informed Consent

Additional consent from the participant will be obtained, if required, for:

- participation in remote visits, as defined in Section "Remote Visits,"
- dispensation of additional study intervention during an extended treatment period,
- alternate delivery of study intervention and ancillary supplies, and
- provision of their personal or medical information required prior to implementation of these activities.

Changes in Study Conduct During Exceptional circumstances

Changes in study conduct not described in this appendix or not consistent with applicable local regulations, are not allowed.

The following changes in study conduct will not be considered protocol deviations.

1. **Remote visits**

In source documents and the eCRF, the study site should capture the visit method, with a specific explanation for the reason of conducting a remote visit instead of an onsite visit and for any data missing because of missed in-person site visits.

Telemedicine:
Telephone or technology-assisted virtual visits, or both, are acceptable to complete appropriate assessments. Assessments to be completed in this manner include, but are not limited to, concomitant medications, review of study participant diary (including study drug compliance), review diet and exercise goals, C-SSRS (Since Last Visit Version), Self-Harm Supplement Form, Self-Harm Follow-up Form (if applicable), and PHQ-9.

Mobile healthcare:
Healthcare visits may be performed by a mobile healthcare provider at locations other than the study site when participants cannot travel to the site due to an exceptional circumstance if written approval is provided by the sponsor. Procedures performed at such visits include, but are not limited to, weight and waist measurements, physical assessments, vital signs, ECG, Patient Reported Outcome questionnaires administration, collection of blood samples and health information.

Other alternative locations:
Laboratory draws may be done at an alternate location in exceptional circumstances.

Regardless of the type of remote visits implemented, the protocol requirements regarding the reporting of AEs, SAEs, and product complaints remain unchanged. Furthermore, every effort should be made to enable participants to return to on-site visits as soon as reasonably possible, while ensuring the safety of both the participants and the site staff.

2. Local laboratory testing option

Local laboratory testing may be conducted in lieu of central laboratory testing. The local laboratory must be qualified in accordance with applicable local regulations.

3. Study intervention and ancillary supplies (including participant diaries)

When a participant is unable to go to the site to receive study supplies during normal on-site visits, the site should work with the sponsor to determine appropriate actions. These actions may include:

- asking the participant to go to the site and receive study supplies from site staff without completion of a full study visit
- asking the participant’s designee to go to the site and receive study supplies on a participant’s behalf, and
- arranging delivery of study supplies.

These requirements must be met before action is taken:

- Alternate delivery of study intervention should be performed in a manner that does not compromise treatment blinding and ensures product integrity. The existing protocol requirements for product accountability remain unchanged, including verification of participant's receipt of study supplies.
- When delivering supplies to a location other than the study site (for example, participant's home), the investigator, sponsor, or both should ensure oversight of the shipping process to ensure accountability and product quality (that is, storage conditions maintained and intact packaging upon receipt).
- Instructions may be provided to the participant or designee on the final disposition of any unused or completed study supplies.

4. Screening period guidance

To ensure safety of study participants, laboratory values and other eligibility assessments taken prior to Visit 3 are valid for a maximum of 90 days. The following rules will be applied for active, nonrandomized participants whose participation in the study must be paused due to exceptional circumstances:

- If paused for less than 90 days from Visit 1 to Visit 3: The participant will proceed to the next study visit per the usual SoA, provided that Visit 3 must be conducted within 90 days from Visit 1.
    - The site should conduct the next visit if the participant's eligibility criteria are confirmed, and the site should document the reason for delay in the CRF.
        - Due to the pause in screening, sites should also reconfirm the impacted participant's consent and document this confirmation in the source documentation.
- If paused for more than 90 days from Visit 1 to Visit 3: The participant must be discontinued because of screening interruption due to an exceptional circumstance. This is documented as a screen failure in the CRF. The participant can reconsent and be rescreened as a new participant. The screening procedures per the usual SoA should be followed, starting at Visit 1 to ensure participant eligibility by Visit 3.

5. Adjustments to Visit Windows

Whenever possible and safe to do so, as determined by the investigator's discretion, participants should complete the usual SoA. To maximize the possibility that these visits can be conducted as on-site visits, the windows for visits may be adjusted, upon further guidance from the sponsor. This minimizes missing data and preserves the intended conduct of the study.

Primary endpoint visit (Visit 21 or 99) should be completed as per original schedule whenever possible and safe to do so. However, the visit windows may be brought forward no sooner than 14 days or extended up to 28 days relative to the target visit date.

For participants whose visits have extended windows, additional study intervention may need to be provided to avoid interruption and maintain overall integrity of the study.

Documentation

Changes to study conduct will be documented:

- Sites will identify and document the details of how participants, visits types, and conducted activities were affected by exceptional circumstances. Dispensing/shipment records of study intervention and relevant communications, including delegation, should be filed with site study records.
- Source documents generated at a location other than the study site should be part of the investigator's source documentation and should be transferred to the site in a secure and timely manner.

10.12. Appendix 12: Abbreviations

Term	Definition
**ADA**	antidrug antibody
**AE**	adverse event: Any untoward medical occurrence in a patient or clinical investigation subject administered a pharmaceutical product that does not necessarily have a causal relationship with this treatment. An adverse event can therefore be any unfavorable and unintended sign (including an abnormal laboratory finding), symptom, or disease temporally associated with the use of a medicinal (investigational) product, whether or not related to the medicinal (investigational) product.
**AESI**	adverse event of special interest
**AHM**	antihyperglycemic medications
**ALP**	alkaline phosphatase
**ALT**	alanine aminotransferase
**ANCOVA**	analysis of covariance
**AST**	aspartate aminotransferase
**BG**	blood glucose
**BMI**	body mass index
**BMR**	basal metabolic rate
**CEC**	clinical endpoint committee
**CFR**	Code of Federal Regulations
**CHF**	congestive heart failure
**CIOMS**	Council for International Organizations of Medical Sciences
**CK**	creatine kinase
**CKD-EPI**	Chronic Kidney Disease-Epidemiology
**CONSORT**	Consolidated Standards of Reporting Trials
**complaint**	A complaint is any written, electronic, or oral communication that alleges deficiencies related to the identity, quality, purity, durability, reliability, safety or effectiveness, or performance of a drug or drug delivery system.
**compliance**	Adherence to all study-related, GCP, and applicable regulatory requirements.
**CONSORT**	Consolidated Standards of Reporting Trials

**CRF**	case report form
**CRP**	clinical research physician: Individual responsible for the medical conduct of the study. Responsibilities of the CRP may be performed by a physician, clinical research scientist, global safety physician or other medical officer.
**CSR**	clinical study report
**C-SSRS**	Columbia-Suicide Severity Rating Scale
**CT**	computed tomography
**CTA**	Clinical Trial Agreement
**DNA**	deoxyribonucleic acid
**DPP-4**	dipeptidyl-peptidase-4
**EAS**	efficacy analysis set
**ECG**	electrocardiogram
**eCRF**	electronic case report form
**ED**	early discontinuation
**EDC**	electronic data capture
**eGFR**	estimated glomerular filtration rate
**EMA**	European Medicines Agency
**enroll**	The act of assigning a participant to a treatment. Participants who are enrolled in the study are those who have been assigned to a treatment.
**enter**	Participants entered into a study are those who sign the informed consent form directly or through their legally acceptable representatives.
**ERB**	ethical review board
**FAS**	full analysis set
**FDA**	Food and Drug Administration
**FSH**	follicle-stimulating hormone
**GCP**	good clinical practice
**GGT**	gamma-glutamyltransferase
**GI**	gastrointestinal
**GIP**	glucose-dependent insulinotropic polypeptide

**GIPR**	glucose-dependent insulinotropic polypeptide receptor
**GLP-1**	glucagon-like peptide-1
**GLP-1R**	glucagon-like peptide-1 receptor
**HbA1c**	hemoglobin A1c
**HDL**	high-density lipoprotein
**HIPAA**	Health Insurance Portability and Accountability Act
**HRT**	hormonal replacement therapy
**HRQoL**	health-related quality of life
**IB**	Investigator's Brochure
**ICF**	informed consent form
**ICH**	The International Council for Harmonization of Technical Requirements for Pharmaceuticals for Human Use
**informed consent**	A process by which a patient voluntarily confirms his or her willingness to participate in a particular study, after having been informed of all aspects of the study that are relevant to the patient's decision to participate. Informed consent is documented by means of a written, signed and dated informed consent form.
**INR**	international normalized ratio
**interim analysis**	An interim analysis is an analysis of clinical study data, separated into treatment groups, that is conducted before the final reporting database is created/locked.
**IP**	Investigational product; a pharmaceutical form of an active ingredient or placebo being tested or used as a reference in a clinical trial, including products already on the market when used or assembled (formulated or packaged) in a way different from the authorized form, or marketed products used for an unauthorized indication, or marketed products used to gain further information about the authorized form.
**IRB/IEC**	Institutional Review Boards /Independent Ethics Committees
**IWQOL-Lite-CT**	Impact of Weight on Quality of Life-Lite-Clinical Trials Version
**IWRS**	interactive web-response system
**LDL**	low-density lipoprotein
**MAD**	multiple ascending dose
**MDD**	Major Depressive Disorder
**MedDRA**	Medical Dictionary for Regulatory Activities
**MEN**	multiple endocrine neoplasia

**MHP**	mental health professional
**mITT**	modified intent-to-treat
**MMRM**	mixed model for repeated measures
**MRI**	magnetic resonance imaging
**MTC**	medullary thyroid carcinoma
**NAFLD**	nonalcoholic fatty liver disease
**NYHA**	New York Heart Association
**p-amylase**	pancreatic amylase
**PC**	product complaint
**PGIs**	Patient Global Impression of status for physical activity
**PHQ-9**	Patient Health Questionnaire-9
**PK/PD**	pharmacokinetics/pharmacodynamics
**PRO**	patient-reported outcome
**QW**	once weekly
**SAD**	single ascending dose
**SAE**	serious adverse event
**SAP**	statistical analysis plan
**SBP**	systolic blood pressure
**SC**	subcutaneous
**screen**	The act of determining if an individual meets minimum requirements to become part of a pool of potential candidates for participation in a clinical study.
**SD**	standard deviation
**SF-36 v2**	Short Form-36 Health Survey (SF-36), version 2 acute form
**SGLT**	sodium-glucose cotransporter
**SMBG**	self-monitoring of blood glucose
**SoA**	Schedule of Activities
**SS**	safety analysis set
**SSRI**	selective serotonin reuptake inhibitor

**T1DM**	Type 1 diabetes mellitus
**T2DM**	Type 2 diabetes mellitus
**TBL**	total bilirubin level
**TE**	treatment-emergent
**TE ADA+**	treatment-emergent antidrug antibody positive
**TEAE**	treatment-emergent adverse event: An untoward medical occurrence that emerges during a defined treatment period, having been absent pretreatment, or worsens relative to the pretreatment state, and does not necessarily have to have a causal relationship with this treatment.
**TEE**	total energy expenditure
**THC**	tetrahydrocannabinol
**TSH**	thyroid-stimulating hormone
**ULN**	upper limit of normal
**WHO**	World Health Organization

11. References

[ADA]. Glycemic targets: standards of medical care in diabetes-2020. *Diabetes Care*. 2020;43(suppl 1):S66–S76. https://doi.org/10.2337/dc20-S006

Alberti, K.G. and Zimmet, P.Z. (1998) Definition, Diagnosis and Classification of Diabetes Mellitus and Its Complications. Part 1: Diagnosis and Classification of Diabetes Mellitus Provisional Report of a WHO Consultation. Diabetic Medicine, 15, 539-553. http://doi.org/10.1002/(SICI)1096-9136(199807)15:7<539::AID-DIA668>3.0.CO;2-S

[AMA]. American Medical Association House of Delegates resolution: 420 (A-13): recognition of obesity as a disease. May 2013. Accessed May 21, 2020. https://media.npr.org/documents/2013/jun/ama-resolution-obesity.pdf

Apovian CM, Aronne LJ, Bessesen DH, et al. Pharmacological management of obesity: an endocrine society clinical practice guideline. *J Clin Endocrinol Metab*. 2015;100(2):342-362. https://doi.org/10.1210/jc.2014-3415

Aroda VR, Ratner R. The safety and tolerability of GLP-1 receptor agonists in the treatment of type 2 diabetes: a review. *Diabetes Metab Res Rev*. 2011;27(6):528-542. https://doi.org/10.1002/dmrr.1202

Baggio LL, Drucker DJ. Biology of incretins: GLP-1 and GIP. *Gastroenterology*. 2007;132(6):2131-2157. https://doi.org/10.1053/j.gastro.2007.03.054

Banks PA, Freeman ML, Practice Parameters Committee of the American College of Gastroenterology. Practice guidelines in acute pancreatitis. *Am J Gastroenterol.* 2006;101(10):2379-2400. https://doi.org/10.1111/j.1572-0241.2006.00856.x

Coskun T, Sloop KW, Loghin C, et al. LY3298176, a novel dual GIP and GLP-1 receptor agonist for the treatment of type 2 diabetes mellitus: from discovery to clinical proof of concept. *Mol Metab*. 2018;18:3-14. https://doi.org/10.1016/j.molmet.2018.09.009

Council on Science and Public Health. Report of the Council on Science and Public Health: is obesity a disease? (Resolution 115-A-12): Report number: 3-A-13. 2013. Accessed May 21, 2020. https://www.ama-assn.org/sites/ama-assn.org/files/corp/media-browser/public/about-ama/councils/Council%20Reports/council-on-science-public-health/a13csaph3.pdf.

Danne T, Philotheou A, Goldman D, et al. A randomized trial comparing the rate of hypoglycemia – assessed using continuous glucose monitoring – in 125 preschool children with type 1 diabetes treated with insulin glargine or NPH insulin (the PRESCHOOL study). *Pediatr Diabetes*. 2013;14(8):593-601. https://doi/10.1111/pedi.12051

Davies MJ, D'Alessio DA, Fradkin J, et al. Management of hyperglycemia in type 2 diabetes, 2018. A consensus report by the American Diabetes Association (ADA) and the European Association for the Study of Diabetes (EASD). *Diabetes Care* 2018;41(12):2669-2701. https://doi.org/10.2337/dci18-0033

Dolan P. Modeling valuations for EuroQol health states. *Med Care.* 1997;35(11):1095-1108. https://doi/10.1097/00005650-199711000-00002

Dombrowski SU, Knittle K, Avenell A, et al. Long-term maintenance of weight loss with non-surgical interventions in obese adults: systematic review and meta-analyses of randomised controlled trials. *BMJ.* 2014;348:g2646. https://doi.org/10.1136/bmj.g2646

[EMA]. Guideline on clinical evaluation of medicinal products used in weight management. EMA/CHMP/311805/2014. Published June 23, 2016. Accessed May 22, 2020https://www.ema.europa.eu/en/documents/scientific-guideline/guideline-clinical-evaluation-medicinal-products-used-weight-management-revision-1_en.pdf. Published 23 June 2016.

EuroQol Research Foundation. EQ-5D-5L User Guide, Version 3.0. Updated September 2019. Accessed March 13, 2020. https://euroqol.org/publications/user-guides.

[FAO/WHO/UNU] Food and Agriculture Organization of the United Nations/World Health Organization/United Nations University. Human energy requirements: report of a joint FAO/WHO/UNU expert consultation. Published October 2004 Accessed May 21, 2020.http://www.fao.org/3/y5686e/y5686e00.htm.

[FDA]. Guidance for industry: developing products for weight management. Published February 2007. Accessed May 22, 2020. https://www.fda.gov/media/71252/download.

[FDA]. FDA requests the withdrawal of the weight-loss drug Belviq, Belviq XR (lorcaserin) from the market: potential risk of cancer outweighs the benefits. Published 14 January 2020. Accessed June 5, 2020. https://www.fda.gov/media/135189/download.

Frias JP, Nauck MA, Van J, et al. Efficacy and safety of LY3298176, a novel dual GIP and GLP-1 receptor agonist, in patients with type 2 diabetes: a randomized, placebo-controlled and active comparator-controlled phase 2 trial. *Lancet.* 2018;392(10160):2180-2193. https://doi.org/10.1016/S0140-6736(18)32260-8

Ganguly R, Tian Y, Kong SX, et al. Persistence of newer anti-obesity medications in a real-world setting. *Diabetes Res Clin Pract.* 2018;143:348-356. https://doi.org/10.1016/j.diabres.2018.07.017

Gerstein HC, Colhoun HM, Dagenais GR, et al. Dulaglutide and cardiovascular outcomes in type 2 diabetes (REWIND): a double-blind, randomized placebo-controlled trial. *Lancet.* 2019;394(10193):121-130. https://doi.org/10.1016/S0140-6736(19)31149-3

Goldstein DJ. Beneficial health effects of modest weight loss. *Int J Obes Relat Metab Disord*. 1992;16(6):397-415.

Jensen MD, Ryan DH, Donato KA, et al. Executive summary: guidelines (2013) for the management of overweight and obesity in adults: a report of the American College of Cardiology/American Heart Association Task Force on Practice Guidelines and the Obesity Society published by the Obesity Society and American College of Cardiology/American Heart Association Task Force on Practice Guidelines. based on a systematic review from the Obesity Expert Panel, 2013. *Obesity.* 2014;22(suppl 2):S5-S39. https://doi.org/10.1002/oby.20821

Koizumi M, Takada T, Kawarada Y, et al. JPN guidelines for the management of acute pancreatitis: diagnostic criteria for acute pancreatitis. *J Hepatobiliary Pancreat Surg*. 2006;13(1):25-32. https://doi.org/10.1007/s00534-005-1048-2

Kolotkin RL, Ervin CM, Meincke HH, et al. Development of a clinical trials version of the impact of weight on quality of life-lite questionnaire (IWQOL-Lite clinical trials version): results from two qualitative studies. *Clin Obes.* 2017;7(5):290-299. https://doi.org/10.1111/cob.12197

Kolotkin RL, Williams VSL, Ervin CM, et al. Validation of a new measure of quality of life in obesity trials: impact of weight on quality of life-lite clinical trials version. *Clin Obes.* 2019;9(3):e12310. https://doi.org/10.1111/cob.12310

Lauby-Secretan, B, Scoccianti C, Loomis D, et al.; International Agency for Research on Cancer Handbook Working Group. Body fatness and cancer-viewpoint of the IARC Working Group. *N Engl J Med.* 2016;375(8):794-798. https://doi.org/10.1056/NEJMsr1606602

Luppino FS, de Wit LM, Bouvy PF, et al. Overweight, obesity, and depression: a systematic review and meta-analysis of longitudinal studies. *Arch Gen Psychiatry.* 2010;67(3):220-229. https://doi.org/10.1001/archgenpsychiatry.2010.2.

Marso SP, Bain SC, Consoli A, et al. Semaglutide and cardiovascular outcomes in patients with type 2 diabetes. *N Engl J Med.* 2016a;375(19):1834-1844. https://doi.org/10.1056/NEJMoa1607141

Marso SP, Daniels GH, Brown-Frandsen K, et al. Liraglutide and cardiovascular outcomes in type 2 diabetes. *N Engl J Med.* 2016b;375(4):311-322. https://doi.org/ 10.1056/NEJMoa1603827

Maruish ME, ed. *User's Manual for the SF-36v2 Health Survey*. 3rd ed. Lincoln, RI: Quality Metric Incorporated; 2011.

Müller TD, Clemmensen C, Finan B, et al. Anti-obesity therapy: from rainbow pills to polyagonists. *Pharmacol Rev*. 2018;70(4):712-746. https://doi.org/10.1124/pr.117.014803

Nauck MA, Meier JJ, Schmidt WE. Incretin-based glucose-lowering medications and the risk of acute pancreatitis and/or pancreatic cancer: reassuring data from cardio-vascular outcome trials. *Diabetes Obes Metab*. 2017;19(9):1327-1328. https://doi.org/10.1111/dom.12981

Pastors JG, Warshaw H, Daly A, et al. The evidence for the effectiveness of medical nutrition therapy in diabetes management. *Diabetes Care.* 2002;25(3):608-613. https://doi.org/10.2337/diacare.25.3.608

Pi-Sunyer FX. Weight loss in type 2 diabetic patients. *Diabetes Care.* 2005;28(6):1526-1527. https://doi.org/10.2337/diacare.28.6.1526

Rothberg AE, McEwen LN, Kraftson AT, et al. Impact of weight loss on waist circumference and the components of the metabolic syndrome. *BMJ Open Diabetes Res Care*. 2017;5(1):e000341. https://doi.org/10.1136/bmjdrc-2016-000341

Ryan DH, Yockey SR. Weight loss and improvement in comorbidity: differences at 5%, 10%, 15%, and over. *Curr Obes Rep.* 2017;6(2):187-194. https://doi.org/10.1007/s13679-017-0262-y

SAXENDA [summary of product characteristics]. Bagsværd, Denmark: Novo Nordisk, 2015.

SAXENDA [package insert]. Bagsvaerd, Denmark: Novo Nordisk; 2014.

Skow MA, Bergman NC, Knop FK. Diabetes and obesity treatment based on dual incretin receptor activation: "twincretins". *Diabetes Obesity Metab.* 2016;18(9):847-854. https://doi.org/10.1111/dom.12685

Srivastava G, Apovian C. Future pharmacotherapy for obesity: new anti-obesity drugs on the horizon. *Curr Obes Rep.* 2018a;7(2):147-161. https://doi.org/ 10.1007/s13679-018-0300-4

Srivastava G, Apovian CM. Current pharmacotherapy for obesity. *Nat Rev Endocrinol.* 2018b;14(1):12-24. https://doi.org/10.1038/nrendo.2017.122

Steinberg WM, Buse JB, Ghorbani MLM, et al. Amylase, lipase, and acute pancreatitis in people with type 2 diabetes treated with liraglutide: results from the LEADER randomized trial. *Diabetes Care.* 2017a;40(7):966-972. https://doi.org/10.2337/dc16-2747

Steinberg WM, Rosenstock J, Wadden TA, et al. Impact of liraglutide on amylase, lipase, and acute pancreatitis in participants with overweight/obesity and normoglycemia, prediabetes, or type 2 diabetes: secondary analyses of pooled data from the SCALE clinical development program. *Diabetes Care.* 2017b;40(7):839-848. https://doi.org/10.2337/dc16-2684

Tomlinson B, Hu M, Zhang Y, et al. An overview of new GLP-1 receptor agonists for type 2 diabetes. *Expert Opin Investig Drugs*. 2016;25(2):145-158. https://doi.org/10.1517/13543784.2016.1123249

van Bloemendaal L, Ten Kulve JS, la Fleur SE, et al. Effects of glucagon-like peptide 1 on appetite and body weight: focus on the CNS. *J Endocrinol.* 2014;221(1):T1-T16. https://doi.org/10.1530/JOE-13-0414

UKPDS Group. UK Prospective Diabetes Study 7: response of fasting plasma glucose to diet therapy in newly presenting type II diabetic patients. *Metabolism.* 1990;39(9):905–912. https://doi.org/10.1016/0026-0495(90)90299-R

Weinberg ME, Bacchetti P, Rushakoff RJ. Frequently repeated glucose measurements overestimate the incidence of inpatient hypoglycemia and severe hyperglycemia. *J Diabetes Sci Technol.* 2010;4(3):577-582. https://doi.org/10.1177/193229681000400311

[WHO]. WHO STEPS surveillance manual: the WHO STEPwise approach to noncommunicable disease risk factor surveillance. Updated 26 January 2017. Accessed May 4, 2020. https://www.who.int/ncds/surveillance/steps/STEPS_Manual.pdf.

Wing RR, Lang W, Wadden TA, et al.; Look AHEAD Research Group. Benefits of modest weight loss in improving cardiovascular risk factors in overweight and obese individuals with type 2 diabetes. *Diabetes Care*. 2011; 34(7):1481-1486. https://doi.org/ 10.2337/dc10-2415

Leo Document ID = 19b74ae5-962b-4a66-aa8c-fc057d6b206c

Approver: PPD

Approval Date & Time: 23-Nov-2020 21:00:11 GMT

Signature meaning: Approved

Approver: PPD

Approval Date & Time: 23-Nov-2020 21:39:12 GMT

Signature meaning: Approved
