# Supplementary material for: Psychiatric Safety of Tirzepatide in People With Obesity and No Known Major Psychopathology: A Post Hoc Analysis of SURMOUNT
Source: Obesity (Silver Spring). 2026 Jan 15;34(3):565–78. doi: 10.1002/oby.70122 (PMC12933222; doi:10.1002/oby.70122)
Supplement: Supplementary file 5 — Supplement S5: Full study protocol for SURMOUNT‐3. [file OBY-34-565-s001.pdf]

SUPPLEMENT 5

Psychiatric Safety of Tirzepatide in People with Obesity and No Known Major Psychopathology: A Pooled Post Hoc Analysis of the SURMOUNT-1, SURMOUNT-2, and SURMOUNT-3 Trials

Thomas A. Wadden, PhD, Maria A. Oquendo, MD, PhD, Robert F. Kushner, MD, Dachuang Cao, PhD, MS, Chrisanthi A. Karanikas, MS, Afton Kechter, PhD, Madhumita A. Murphy, MD, MS, MBA

This supplement provides data on: Study protocol for the SURMOUNT-3 trial.

Title Page

Confidential Information

The information contained in this document is confidential and is intended for the use of clinical investigators. It is the property of Eli Lilly and Company or its subsidiaries and should not be copied by or distributed to persons not involved in the clinical investigation of tirzepatide (LY3298176), unless such persons are bound by a confidentiality agreement with Eli Lilly and Company or its subsidiaries.

Note to Regulatory Authorities: This document may contain protected personal data and/or commercially confidential information exempt from public disclosure. Eli Lilly and Company requests consultation regarding release/redaction prior to any public release. In the United States, this document is subject to Freedom of Information Act (FOIA) Exemption 4 and may not be reproduced or otherwise disseminated without the written approval of Eli Lilly and Company or its subsidiaries.

Protocol Title: Efficacy and Safety of Tirzepatide Once Weekly versus Placebo After an Intensive Lifestyle Program in Participants without Type 2 Diabetes who have Obesity or are Overweight with Weight-Related Comorbidities: A Randomized, Double-Blind, Placebo-Controlled Trial (SURMOUNT-3)

Protocol Number: I8F-MC-GPHM

Amendment number: This is the initial protocol.

Compound: LY3298176

Study Phase: 3

Short Title: Effect of Tirzepatide versus Placebo after Intensive Lifestyle Program (SURMOUNT-3)

Acronym: SURMOUNT-3

Sponsor Name: Eli Lilly and Company

Legal Registered Address: Indianapolis, Indiana USA 46285

Regulatory Agency Identifier Number(s)

IND: 139721

Approval Date: Protocol Electronically Signed and Approved by Lilly on date provided below.

Approval Date: 19-Nov-2020 GMT

Medical Monitor Name and Contact Information will be provided separately.

Table of Contents

1. Protocol Summary 6
1.1. Synopsis 6
1.2. Schema 11
1.3. Schedule of Activities (SoA) 12

2. Introduction 18
2.1. Study Rationale 18
2.2. Background 19
2.3. Benefit/Risk Assessment 20

3. Objectives and Endpoints 21

4. Study Design 23
4.1. Overall Design 23
4.1.1. Overview of Study Periods 23
4.1.2. Study Procedures 27
4.2. Scientific Rationale for Study Design 28
4.3. Justification for Dose 29
4.4. End of Study Definition 30

5. Study Population 31
5.1. Inclusion Criteria 31
5.2. Exclusion Criteria 33
5.3. Lifestyle Considerations 37
5.3.1. Meals and Dietary Restrictions 37
5.3.2. Physical Activity 38
5.4. Screen Failures 39

6. Study Intervention 40
6.1. Study Intervention(s) Administered 40
6.1.1. Medical Devices 40
6.2. Preparation/Handling/Storage/Accountability 41
6.3. Measures to Minimize Bias: Randomization and Blinding 41
6.4. Study Intervention Compliance 42
6.5. Concomitant Therapy 42
6.6. Dose Modification 43
6.6.1. Tirzepatide 43
6.6.2. Management of Participants with Gastrointestinal Symptoms 43
6.7. Intervention after the End of the Study 43

7. Discontinuation of Study Intervention and Participant Discontinuation/Withdrawal 44
7.1. Discontinuation of Study Intervention 44
7.1.1. Temporary Discontinuation 47
7.2. Participant Discontinuation/Withdrawal from the Study 47
7.2.1. Participant Disposition and Timing of Safety Follow-Up 49
7.2.2. Discontinuation of Inadvertently Enrolled Participants 50

7.3. Lost to Follow up 50

8. Study Assessments and Procedures 51
8.1. Efficacy Assessments 51
8.1.1. Primary Efficacy Assessments 51
8.1.2. Secondary Efficacy Assessments 51
8.1.3. Patient-Reported Outcomes Assessments 51
8.2. Safety Assessments 53
8.2.1. Physical Examinations 53
8.2.2. Vital Signs 53
8.2.3. Electrocardiograms 53
8.2.4. Clinical Safety Laboratory Assessments 54
8.2.5. Safety Monitoring 55
8.2.6. Depression, Suicidal Ideation and Behavior Risk Monitoring 57
8.3. Adverse Events, Serious Adverse Events, and Product Complaints 57
8.3.1. Timing and Mechanism for Collecting Events 58
8.3.2. Special Safety Topics 60
8.4. Treatment of Overdose 65
8.5. Pharmacokinetics 65
8.6. Pharmacodynamics 65
8.7. Genetics 65
8.8. Biomarkers 66
8.9. Immunogenicity Assessments 66
8.10. Health Economics 67

9. Statistical Considerations 68
9.1. Statistical Hypotheses 68
9.2. Sample Size Determination 68
9.3. Populations for Analyses 69
9.4. Statistical Analyses 69
9.4.1. General Considerations 69
9.4.2. Treatment Group Comparability 70
9.4.3. Efficacy Analyses 71
9.4.4. Safety Analyses 72
9.4.5. Pharmacokinetic/Pharmacodynamic Analyses 73
9.4.6. Evaluation of Immunogenicity 73
9.4.7. Other Analyses 74
9.5. Interim Analyses 74
9.6. Data Monitoring Committee (DMC) 74

10. Supporting Documentation and Operational Considerations 75
10.1. Appendix 1: Regulatory, Ethical, and Study Oversight Considerations 75
10.1.1. Regulatory and Ethical Considerations 75
10.1.2. Informed Consent Process 75
10.1.3. Data Protection 76
10.1.4. Committees Structure 76

10.1.5. Dissemination of Clinical Study Data 76
10.1.6. Data Quality Assurance 77
10.1.7. Source Documents 78
10.1.8. Study and Site Start and Closure 78
10.1.9. Publication Policy 79
10.2. Appendix 2: Clinical Laboratory Tests 80
10.3. Appendix 3: Laboratory Assessments for Hypersensitivity Events 83
10.4. Appendix 4: Adverse Events: Definitions and Procedures for Recording, Evaluating, Follow-up, and Reporting 85
10.4.1. Definition of AE 85
10.4.2. Definition of SAE 86
10.4.3. Definition of Product Complaint 87
10.4.4. Recording and Follow-Up of AE and/or SAE and Product Complaints 87
10.4.5. Reporting of SAEs 89
10.4.6. Regulatory Reporting Requirements 90
10.5. Appendix 5: Contraceptive Guidance and Collection of Pregnancy Information 91
10.6. Appendix 6: Liver Safety: Suggested Actions and Follow-up Assessments 95
10.7. Appendix 7: Protocol GPHM Standardized Protocols for the Measurement of Height, Weight, Waist Circumference, Vital Signs and Electrocardiogram 97
10.8. Appendix 8: Suggested Visit Structure 99
10.9. Appendix 9: Management of Gastrointestinal Symptoms 100
10.10. Appendix 10: Provisions for Changes in Study Conduct During Exceptional Circumstances 101
10.11. Appendix 11: Abbreviations 105

11. References 110

1. Protocol Summary

1.1. Synopsis

Protocol Title: Efficacy and Safety of Tirzepatide Once Weekly versus Placebo After an Intensive Lifestyle Program in Participants without Type 2 Diabetes who have Obesity or are Overweight with Weight-Related Comorbidities: A Randomized, Double-Blind, Placebo-Controlled Trial (SURMOUNT-3)

Short Title: Effect of Tirzepatide versus Placebo after Intensive Lifestyle Program (SUMROUNT-3)

Rationale:

Obesity is a chronic disease and its increasing prevalence is a public health concern associated with rising incidence of Type 2 diabetes mellitus (T2DM), increased risk for premature death, and increased risk for some cancers (American Medical Association [AMA] 2013; Council on Science and Public Health 2013; Lauby-Secretan et al. 2016). The cornerstone of obesity treatment is lifestyle-based therapy combining reduced-calorie diet, physical activity, and behavioral counseling (NHLBI 2000; Jensen et al. 2014; Garvey et al. 2016). Evidence-based clinical approaches to caloric restriction include moderate energy deficits of 500 to 750 kcal/day based on calculation of an individual's energy needs or more intensive low-calorie diets (LCDs) with gender-specific energy intake targets of 1200 to 1800 kcal/day. Ad libitum approaches involving restriction or elimination of particular foods without specific energy targets may also be used (Jensen et al. 2014; Garvey et al. 2016).

Lifestyle interventions lead to 5 to 10% body weight loss, which has been shown to reduce obesity-related cardiovascular risk factors, and in some cases improve health-related quality of life (Mertens and Van Gaal 2000; Knowler et al. 2002; Jensen et al. 2014; Kolotkin and Andersen 2017). However, diet- and exercise-induced weight loss is not maintained in 80% of patients, in part due to metabolic adaptations that promote regain in response to caloric deficits (Leibel et al. 1995; Sumithran et al. 2011; Doucet et al. 2018). Moreover, greater weight losses can maximize metabolic and health-related quality of life benefits and may be required to realize clinically meaningful improvements in other obesity-related comorbidities such as sleep apnea, nonalcoholic steatohepatitis, and cardiovascular disease (Garvey et al. 2016; Kolotkin and Anderson 2017; Ryan and Yockey 2017).

There is widespread consensus that pharmacotherapy for weight management should only be used as an adjunct to lifestyle-based approaches (NHLBI 2000; Jensen et al. 2014; Garvey et al. 2016). There seems to be less clarity, however, on when and in whom obesity treatment should be advanced from lifestyle-based monotherapy to lifestyle plus medication combination therapy. While some guidelines recommend pharmacotherapy only after diet and exercise fail to achieve weight loss over 6 months (NHLBI 2000), others add that it should also be considered if initial weight loss is not maintained over time (Jensen et al. 2014), and still others imply that even responders to diet and exercise may benefit from additional weight loss with medications (Garvey et al. 2016).

The gut incretin hormones, glucose-dependent insulinotropic polypeptide (GIP) and glucagon-like peptide-1 (GLP-1), are secreted after meal ingestion and mediate the incretin effect. Both hormones have effects on endocrine cells in the pancreas, increasing insulin biosynthesis and secretion, and modifying glucagon secretion (Skow et al. 2016). Based on these properties, several GLP-1 receptor (GLP-1R) agonists have been approved for pharmacological treatment of T2DM (Tomlinson et al. 2016).

In addition to its pancreatic effects, GLP-1R activation decreases gut motility, slows gastric emptying, and promotes satiety (presumably through a combination of GLP-1R activation in the central and peripheral nervous systems), thereby regulating food intake and body weight (Baggio and Drucker 2007). The US Food and Drug Administration and the European Medicines Agency approved the GLP-1R agonist liraglutide for the treatment of overweight and obesity (SAXENDA® package insert, 2014; SAXENDA summary of product characteristics, 2015).

Preclinical data indicate that GIP also exerts effects on appetite regulation and food intake, on adipose tissue, and on peripheral energy metabolism. Although studies evaluating effects of GIP on body weight have yielded equivocal results, GIP receptor (GIPR) activation may play a role in body weight regulation and targeting both the GLP-1R and the GIPR simultaneously could potentially result in additive or synergistic effects of the 2 incretins on body weight (Coskun et al. 2018).

Tirzepatide is a 39-amino acid synthetic peptide dual GIPR and GLP-1R agonist. Its structure is based on the GIP sequence and includes a C20 fatty diacid moiety (Coskun et al. 2018). It is administered once-weekly (QW) by subcutaneous (SC) injection.

As a dual GIP/GLP-1R agonist, tirzepatide could exceed the efficacy of selective GLP-1R agonists by recruiting metabolically active tissues not targeted by selective GLP-1R agonists (for example, adipose tissue as indicated by the observation of increased energy utilization) (Müller et al. 2018) and has the potential to reach higher efficacy in target tissues that express both GIPR and GLP-1R. Therefore, tirzepatide has the potential to impact several aspects of energy regulation and to be a treatment of overweight and obesity.

In a 26-week Phase 2 study in patients with T2DM, tirzepatide 10 mg and 15 mg doses produced statistically significant and clinically relevant weight loss compared to placebo and compared to the GLP-1 R agonist, dulaglutide. Tirzepatide is subsequently being evaluated in Phase 3 studies in patients with obesity for its effects on weight loss as an adjunct to a 500 kcal/day deficit diet and increased physical activity. There is a need to better understand the benefits of tirzepatide after a more intensive lifestyle program, especially as current recommendations vary with regards to when to prescribe pharmacotherapy to diet and exercise responders and goals of treatment in such patients may range from promoting maintenance to augmenting the initial response.

Study 18F-MC-GPHM (GPHM; SURMOUNT-3) is a Phase 3, multicenter, randomized, parallel-arm, double-blind, placebo-controlled, 84-week study that will investigate the effects of treatment with maximum tolerated dose (MTD) of tirzepatide (10 mg or 15 mg QW), compared with placebo, on body weight management in study participants who do not have T2DM and have obesity (body mass index [BMI] ≥30 kg/m^2) or are overweight (BMI ≥27 kg/m^2) with at least 1 weight-related comorbid condition and achieved ≥5.0% weight loss after a 12-week lead-in period with an intensive lifestyle modification program.

Objectives and Endpoints

Objectives	Endpoints
**Primary**	
To demonstrate that tirzepatide MTD is superior to placebo from randomization for the following (measured at 72 weeks):<br><br>• Percent change in body weight AND<br>• Proportion of participants with ≥5% body weight reduction	• Mean percent change in body weight<br>• Percentage of study participants who achieve ≥5% body weight reduction
**Key Secondary (controlled for type I error)**	
To demonstrate that tirzepatide MTD is superior to placebo from randomization for the following (measured at 72 weeks):<br><br>• Maintaining body weight reduction achieved during the 12-week lead-in period	• Percentage of study participants who maintain ≥80% of the body weight lost during the 12-week lead-in period
• Body weight	• Percentage of study participants who achieve:<br>o ≥10% body weight reduction<br>o ≥15% body weight reduction
• Waist circumference	• Mean change in waist circumference (cm)
**Additional Secondary**	
To demonstrate that tirzepatide MTD is superior to placebo from randomization for the following (measured at 72 weeks):<br><br>• Body weight and BMI	• Mean change in body weight (kg)<br>• Mean change in BMI (kg/m^2)
• Blood pressure	• Mean change in<br>o systolic blood pressure (mmHg)<br>o diastolic blood pressure (mmHg)
• Lipid parameters	• Mean change in:<br>o Total cholesterol (mg/dL)<br>o HDL-cholesterol (mg/dL)<br>o LDL-cholesterol (mg/dL)<br>o VLDL-cholesterol (mg/dL)<br>o Triglycerides (mg/dL)<br>o Free fatty acids (mg/dL)

Objectives	Endpoints
• Glycemic control	• Mean change in<br>o fasting glucose (mg/dL)<br>o HbA1c (%)
• Insulin	• Mean change in fasting insulin (pmol/L)
• Patient-reported outcomes	• Mean change in<br>o SF-36v2 acute form Physical Functioning domain score<br>o IWQOL-Lite-CT Physical Function composite score
To demonstrate that tirzepatide MTD is superior to placebo from Visit 2 for the following (measured at 72 weeks):	
• Body weight and BMI	• Mean change in absolute body weight (kg)<br>• Mean percent change in body weight<br>• Mean change in BMI (kg/m^2)
• Waist circumference	• Mean change in waist circumference (cm)

Abbreviations: BMI = body mass index; HbA1c = hemoglobin A1c; HDL = high-density lipoprotein; IWQOL-Lite-CT = Impact of Weight on Quality of Life-Lite Clinical Trials Version; LDL = low-density lipoprotein; MTD = maximum tolerated dose; SF-36v2 acute form = Short Form-36 Version 2 Health Survey acute form; VLDL = very low-density lipoprotein.

Overall Design

Study GPHM is a Phase 3, multicenter, randomized, parallel-arm, double-blind, placebo-controlled, 84-week study that will investigate the effects of treatment with tirzepatide MTD (10 mg or 15 mg QW), compared with placebo, on body weight management in study participants who have obesity (BMI ≥30 kg/m^2) or are overweight (BMI ≥27 kg/m^2) with at least 1 weight-related comorbid condition and achieved ≥5.0% weight loss after a 12-week lead-in period with an intensive lifestyle modification program.

Randomization at the end of the 12-week lead-in period will be stratified by country, sex, and percent weight loss at the end of lead-in (<10% versus ≥10%).

Disclosure Statement: This is a study including 2 parallel treatment arms (tirzepatide MTD versus placebo) that is participant- and investigator-blinded.

Number of Participants:

Approximately 1100 participants will be screened and 800 participants enrolled into the 12-week intensive lifestyle modification lead-in period in order to get approximately 600 participants who will be randomized in a 1:1 ratio to tirzepatide MTD (300 participants) or placebo (300 participants). An upper limit of 70% enrollment of women will be used to ensure a sufficiently large sample of men.

Intervention Groups and Duration:

Study participants will be randomized in a 1:1 ratio (tirzepatide MTD QW and placebo QW), stratified by country, sex and percent weight loss at the end of lead-in period (<10% versus ≥10%).

Study GPHM will consist of 4 periods: a 2-week screening period; a 12-week lead-in period during which participants undertake an intensive lifestyle modification program to achieve ≥5.0% body weight loss; a 72-week double-blind placebo-controlled dose escalation and treatment period; and a 4-week safety follow-up period.

Data Monitoring Committee: No

1.2. Schema

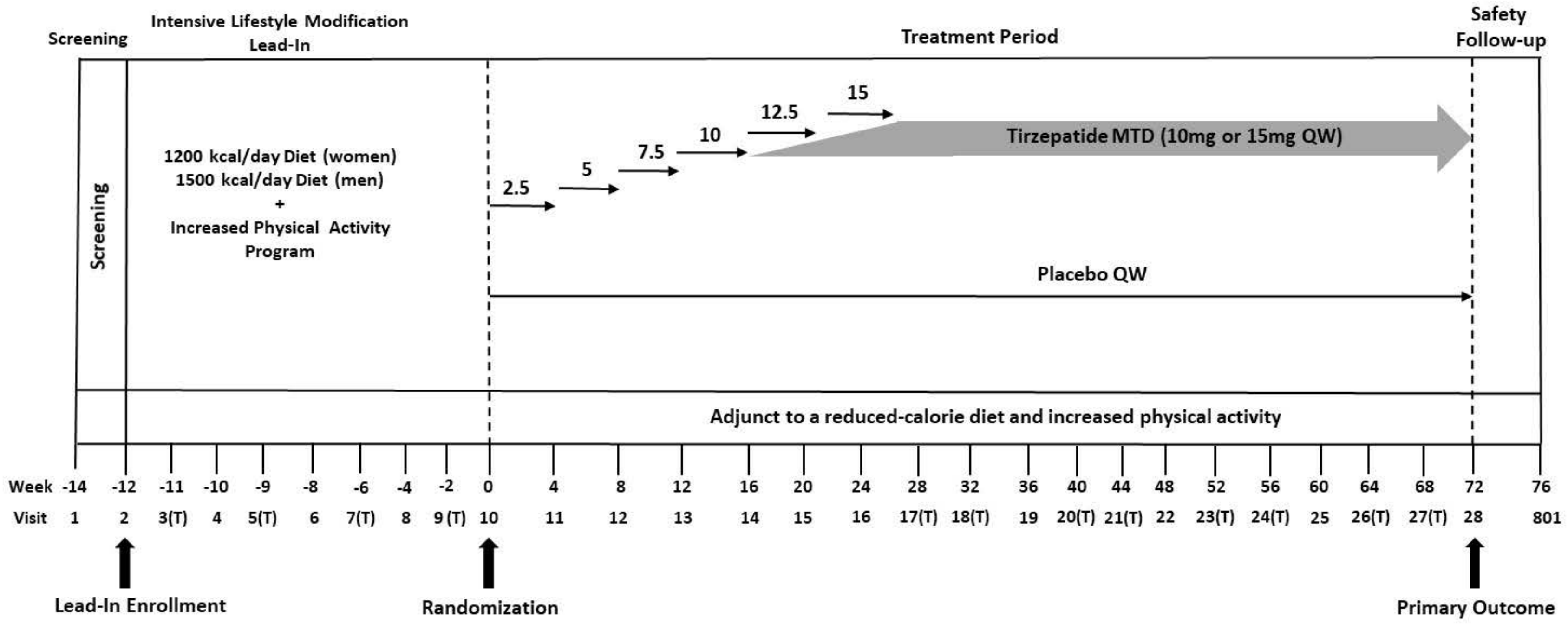

Abbreviations: MTD = maximum tolerated dose, QW = once weekly, (T) = telephone visit.

1.3. Schedule of Activities (SoA)

The Schedule of Activities described below should be followed for all participants enrolled in Study GPHM. However, for those participants whose participation in this study is affected by exceptional circumstances (such as pandemics or natural disasters), please refer to Section 10.10 (Appendix 10) for additional guidance.

Visit	1	2	3	4	5	6	7	8	9	10	11	12	13	14	15	16	17	18	19	20	21	22	23	24	25	26	27	28	99	ED	801
**Week of Treatment**	-14	-12	-11	-10	-9	-8	-6	-4	-2	0	4	8	12	16	20	24	28	32	36	40	44	48	52	56	60	64	68	72	72		4 wks Post TxP
**Allowable Deviation (days)**		±3	±3	±3	±3	±3	±3	±3	±3	±3	±3	±3	±3	±3	±3	±3	±3	±3	±3	±3	±3	±3	±3	±3	±3	±3	±3	±7	±7		±3
**Fasting Visit**	X	X		X		X		X		X	X	X	X	X	X	X			X			X			X			X	X	X	X
**Telephone Visit**			X		X		X		X								X	X		X	X		X	X		X	X				
**Lifestyle Program Instruction only**			X		X		X		X																						
Informed consent	X																														
Inclusion and exclusion criteria review	X	X																													
Preexisting conditions and medical history, including relevant surgical history	X																														
	Medical history includes assessment of preexisting conditions (for example, history of gallbladder disease, cardiovascular disease, and medullary thyroid carcinoma) and substance usage (such as alcohol and tobacco).																														
Concomitant medications	X	X		X		X		X		X	X	X	X	X	X	X	X	X	X	X	X	X	X	X	X	X	X	X	X	X	X
Adverse events and product complaints	X	X		X		X		X		X	X	X	X	X	X	X	X	X	X	X	X	X	X	X	X	X	X	X	X	X	X
**Physical Evaluation or Clinical Assessments**																															
Height	X																														
Weight	X	X		X		X		X		X	X	X	X	X	X	X			X			X			X			X	X	X	X
	Weight measurements should be obtained per the detailed protocol guidance in Section 10.7. Body weight must be measured in the fasting state. If the participant is not fasting, the participant should be called in for a new visit within the visit window to have the fasting body weight measured.																														
Waist circumference		X		X		X		X		X	X	X	X	X	X	X			X			X			X			X	X	X	X
Vital Signs (systolic and diastolic blood pressure, pulse rate)	X	X		X		X		X		X	X	X	X	X	X	X			X			X			X			X		X	X
	Vital sign measurements should be taken before obtaining an ECG tracing and before collection of blood samples for laboratory testing, per the instructions in Section 10.7.																														
Physical examination	X																														
Electrocardiogram		X								X						X												X		X	X

Visit	1	2	3	4	5	6	7	8	9	10	11	12	13	14	15	16	17	18	19	20	21	22	23	24	25	26	27	28	99	ED	801
**Week of Treatment**	-14	-12	-11	-10	-9	-8	-6	-4	-2	0	4	8	12	16	20	24	28	32	36	40	44	48	52	56	60	64	68	72	72		4 wks Post TxP
**Allowable Deviation (days)**		±3	±3	±3	±3	±3	±3	±3	±3	±3	±3	±3	±3	±3	±3	±3	±3	±3	±3	±3	±3	±3	±3	±3	±3	±3	±3	±7	±7		±3
**Fasting Visit**	X	X		X		X		X		X	X	X	X	X	X	X			X			X			X			X	X	X	X
**Telephone Visit**			X		X		X		X								X	X		X	X		X	X		X	X				
**Lifestyle Program Instruction only**			X		X		X		X																						
	ECG measurements should be obtained per the instructions in Section 10.7. ECGs should be collected at least 30 minutes prior to collection of blood samples for laboratory testing.																														
**Participant Education and Assessment**																															
Lifestyle Program Instruction		X	X	X	X	X	X	X	X	X			X			X			X			X			X			X		X	
	Counselling on diet and exercise, to be performed by a dietician or equivalent qualified delegate, to include calculation of individualized energy requirement and behavior modification strategies to change dietary composition and amount of physical activity (See Section 5.3 for details). The Lifestyle Program Instruction may be delivered on a separate day from the rest of that visit's study procedures but must occur within the visit window. After Week 0 (Visit 10, randomization), the remaining Lifestyle Program Instruction may be delivered by phone.																														
Review diet and exercise goals		X	X	X	X	X	X	X	X	X	X	X	X	X	X	X	X	X	X	X	X	X	X	X	X	X	X	X		X	
	All training should be repeated as needed to encourage participant compliance. Study personnel to provide reinforcement and encouragement for lifestyle modifications.																														
Injection training with autoinjector demonstration device										X																					
**Participant Diary (paper)**																															
Dispense diary, instruct in use										X	X	X	X	X	X	X			X			X			X						
	All training should be repeated as needed to encourage participant compliance.																														
Diary review											X	X	X	X	X	X	X	X	X	X	X	X	X	X	X	X	X	X		X	
**Patient Reported Outcomes**																															
PGIs		X								X																		X		X	
SF-36 v2, acute form		X								X																		X		X	
IWQOL-Lite-CT		X								X																		X		X	

Visit	1	2	3	4	5	6	7	8	9	10	11	12	13	14	15	16	17	18	19	20	21	22	23	24	25	26	27	28	99	ED	801
**Week of Treatment**	-14	-12	-11	-10	-9	-8	-6	-4	-2	0	4	8	12	16	20	24	28	32	36	40	44	48	52	56	60	64	68	72	72		4 wks Post TxP
**Allowable Deviation (days)**		±3	±3	±3	±3	±3	±3	±3	±3	±3	±3	±3	±3	±3	±3	±3	±3	±3	±3	±3	±3	±3	±3	±3	±3	±3	±3	±7	±7		±3
**Fasting Visit**	X	X		X		X		X		X	X	X	X	X	X	X			X			X			X			X	X	X	X
**Telephone Visit**			X		X		X		X								X	X		X	X		X	X		X	X				
**Lifestyle Program Instruction only**			X		X		X		X																						
EQ-5D-5L		X								X																		X		X	
**Mental Health Questionnaires**																															
PHQ-9	X									X			X			X			X			X			X			X	X	X	X
	The PHQ-9 is self-administered and should be completed *after* assessment of adverse events.																														
C-SSRS (Baseline/Screening Version)	X																														
	The C-SSRS should be administered *after* assessment of adverse events. For this study, the C-SSRS is adapted for the assessment of the ideation and behavior categories only. The Intensity of Ideation and Lethality of Behavior sections are removed.																														
C-SSRS (Since Last Visit Version)		X		X		X		X		X	X	X	X	X	X	X	X	X	X	X	X	X	X	X	X	X	X	X	X	X	X
	The C-SSRS should be administered *after* assessment of adverse events and capture information since most recent C-SSRS administration. For this study, the C-SSRS is adapted for the assessment of the ideation and behavior categories only. The Intensity of Ideation and Lethality of Behavior sections are removed.																														
Self-Harm Supplement Form	X	X		X		X		X		X	X	X	X	X	X	X	X	X	X	X	X	X	X	X	X	X	X	X	X	X	X
	The Self-Harm Supplement Form should be administered *after* assessment of adverse events.																														
Self-Harm Follow-up Form	X	X		X		X		X		X	X	X	X	X	X	X	X	X	X	X	X	X	X	X	X	X	X	X	X	X	X
	The Self-Harm Follow-up Form is only required if triggered by the Self-Harm Supplement Form, per instructions in the form.																														
**Laboratory Tests and Sample Collections**																															
Hematology	X									X			X			X												X		X	X
HbA1c	X									X			X			X			X			X			X			X		X	X

**Visit**	**1**	**2**	**3**	**4**	**5**	**6**	**7**	**8**	**9**	**10**	**11**	**12**	**13**	**14**	**15**	**16**	**17**	**18**	**19**	**20**	**21**	**22**	**23**	**24**	**25**	**26**	**27**	**28**	**99**	**ED**	**801**
**Week of Treatment**	**-14**	**-12**	**-11**	**-10**	**-9**	**-8**	**-6**	**-4**	**-2**	**0**	**4**	**8**	**12**	**16**	**20**	**24**	**28**	**32**	**36**	**40**	**44**	**48**	**52**	**56**	**60**	**64**	**68**	**72**	**72**		**4 wks Post TxP**
**Allowable Deviation (days)**		**±3**	**±3**	**±3**	**±3**	**±3**	**±3**	**±3**	**±3**	**±3**	**±3**	**±3**	**±3**	**±3**	**±3**	**±3**	**±3**	**±3**	**±3**	**±3**	**±3**	**±3**	**±3**	**±3**	**±3**	**±3**	**±3**	**±7**	**±7**		**±3**
**Fasting Visit**	**X**	**X**		**X**		**X**		**X**		**X**	**X**	**X**	**X**	**X**	**X**	**X**			**X**			**X**			**X**			**X**	**X**	**X**	**X**
**Telephone Visit**			**X**		**X**		**X**		**X**								**X**	**X**		**X**	**X**		**X**	**X**		**X**	**X**				
**Lifestyle Program Instruction only**			**X**		**X**		**X**		**X**																						
Chemistry panel (includes Cr for eGFR calculation and glucose)	X									X			X			X			X			X			X			X		X	X
	The CKD-EPI equation will be used by the central lab to estimate and report eGFR.																														
Insulin	X									X			X			X			X			X			X			X		X	X
C-peptide	X									X			X			X			X			X			X			X		X	X
Lipid panel	X									X						X												X		X	X
Free fatty acids	X									X						X												X		X	X
Serum pregnancy	X									X																					
	For women of childbearing potential only.																														
Urine pregnancy (local)										X			X			X			X			X			X			X		X	
	A urine pregnancy test must be performed at Visit 10 with the result available prior to first injection of study drug(s) for women of childbearing potential only. Additional pregnancy tests (beyond those required by the SoA) should be performed at any time during the trial if a menstrual period is missed, there is clinical suspicion of pregnancy, or as required by local law or regulation.																														
Follicle-stimulating hormone test	X																														
	Follicle-stimulating hormone test is to be performed at Visit 1 for postmenopausal women at least 40 years of age with an intact uterus, not on hormone therapy, and who have had spontaneous amenorrhea for more than 1 year without an alternative medical cause.																														
Calcitonin	X									X			X			X												X		X	X
Pancreatic amylase	X									X			X			X												X		X	X
Lipase	X									X			X			X												X		X	X
Urinary albumin/creatinine ratio	X									X						X												X		X	X
Cystatin-c	X									X						X												X		X	X

Visit	1	2	3	4	5	6	7	8	9	10	11	12	13	14	15	16	17	18	19	20	21	22	23	24	25	26	27	28	99	ED	801
**Week of Treatment**	-14	-12	-11	-10	-9	-8	-6	-4	-2	0	4	8	12	16	20	24	28	32	36	40	44	48	52	56	60	64	68	72	72		4 wks Post TxP
**Allowable Deviation (days)**		±3	±3	±3	±3	±3	±3	±3	±3	±3	±3	±3	±3	±3	±3	±3	±3	±3	±3	±3	±3	±3	±3	±3	±3	±3	±3	±7	±7		±3
**Fasting Visit**	X	X		X		X		X		X	X	X	X	X	X	X			X			X			X			X	X	X	X
**Telephone Visit**			X		X		X		X								X	X		X	X		X	X		X	X				
**Lifestyle Program Instruction only**			X		X		X		X																						
Thyroid stimulating hormone	X																														
Immunogenicity (includes PK sample)										X	X		X			X						X						X		X	X
	In the event of systemic drug hypersensitivity reactions (immediate or nonimmediate), additional unscheduled samples should be collected as detailed in Section 8.3.2.7 (Hypersensitivity Reactions). Immunogenicity samples and PK samples for immunogenicity must be taken prior to drug administration.																														
**Stored Samples**																															
Pharmacogenetic sample										X																					
Nonpharmacogenetic sample	X									X			X			X						X						X		X	X
**Randomization and Dosing**																															
Randomization										X																					
Dispense study intervention(s)										X	X	X	X	X	X	X			X			X			X						
Observe participant administer study drug intervention										X																					
	Participants should administer their first dose of study drug at the end of Visit 10, after other study procedures are completed.																														
Participant returns study drug intervention and injection supplies											X	X	X	X	X	X			X			X			X			X		X	
Assess study intervention(s) compliance											X	X	X	X	X	X	X	X	X	X	X	X	X	X	X	X	X	X		X	

Abbreviations: CKD-EPI = Chronic Kidney Disease-Epidemiology; C-SSRS = Columbia Suicide Severity Rating Scale; Cr = creatinine; ED = early discontinuation; eGFR = estimated glomerular filtration rate; HbA1c = hemoglobin A1c; IWQOL-Lite-CT = Impact of Weight on Quality of Life-Lite-Clinical Trials Version; PGIs = Patient Global Impression of status for physical activity; PHQ-9 = Patient Health Questionnaire-9; PK = pharmacokinetics; SF-36v2 acute form = Short Form-36 Version 2 Health Survey acute form; SoA = schedule of activities; TxP = treatment period; wks = weeks.

Note:

- The visit date is determined in relation to the date of Visit 2 for the lead-in period and Visit 10 (randomization) for the treatment period.
- For all office visits, remind participants to report to the site in a fasting condition, after a period of approximately 8 hours without eating, drinking (except water), or any significant physical activity Since some screening procedures need to be completed in the fasting state, Visit 1 may be conducted over more than 1 day to ensure necessary conditions are met. Eligible participants enter a lifestyle modification lead-in period, during which they receive instruction in an intensive dietary (1200 kcal/day for women or 1500 kcal/day for men) and exercise program to achieve a ≥5.0% body weight loss.
- On Lifestyle Program Instruction Only Visits, all spontaneously reported adverse events by participant will be documented by the investigator and any qualified designee.
- Visit 10 assessments must be completed before processing in the interactive web-response system (IWRS).
- Visit 99 is only applicable to participants who discontinue the double-blind study treatment prematurely (after Week 0 and before Week 72) and decline to complete the remaining scheduled study visits. Participants wanting to discontinue the study treatment after randomization and before Week 72 will be asked to return for Visit 99 (72 weeks ±7 days after Visit 10) primarily for body weight measurement and assessment of adverse events. If the participant is unwilling to attend Visit 99, it should be documented in the participant medical record that the participant has refused to attend.
- Participants who are unable or unwilling to continue the study intensive lifestyle intervention (during lead-in period) or study drug intervention (during treatment period) for any reason will perform an ED visit. If the participant is discontinuing during an unscheduled visit or a scheduled visit, that visit should be performed as an ED visit.
- Visit 801 (safety follow-up visit) should be performed 4 weeks after the last visit of the participant's treatment period (after randomization, see Section 4.1.1.2.3)

2. Introduction

Obesity is a chronic disease and its increasing prevalence is a public health concern associated with rising incidence of type 2 diabetes mellitus (T2DM), increased risk for premature death, and increased risk for some cancers (American Medical Association [AMA] 2013; Council on Science and Public Health 2013; Lauby-Secretan et al. 2016). The cornerstone of obesity treatment is lifestyle-based therapy combining reduced-calorie diet, physical activity and behavioral counseling (NHLBI 2000; Jensen et al. 2014; Garvey et al. 2016). Evidence-based clinical approaches to caloric restriction include moderate energy deficits of 500 to 750 kcal/day based on calculation of an individual's energy needs or more intensive low-calorie diets (LCDs) with gender-specific energy intake targets of 1200 to 1800 kcal/day. Ad libitum approaches involving restriction or elimination of particular foods without specific energy targets may also be used (Jensen et al. 2014; Garvey et al. 2016).

These lifestyle interventions lead to 5 to 10% body weight loss that has been shown to reduce obesity-related cardiovascular risk factors, and in some cases improve health-related quality of life (Mertens and Van Gaal 2000; Knowler et al. 2002; Jensen et al. 2014; Kolotkin and Andersen 2017). However, diet- and exercise-induced weight loss is not maintained in 80% of patients, in part due to metabolic adaptations that promote regain in response to caloric deficits (Leibel et al. 1995; Sumithran et al. 2011; Doucet et al. 2018). Adjunctive therapies, including medications, devices, and surgical treatments, for obesity are evolving as are the clinical guidelines for the application of these therapies in the clinical management of patients with obesity.

2.1. Study Rationale

The gut incretin hormones, glucose-dependent insulinotropic polypeptide (GIP) and glucagon-like peptide-1 (GLP-1), are secreted after meal ingestion and mediate the incretin effect.

Both hormones have effects on endocrine cells in the pancreas, increasing insulin biosynthesis and secretion, stimulating beta-cell neogenesis and proliferation, and protecting beta cells from apoptosis. They also exert actions on alpha cells, modifying glucagon secretion (Skow et al. 2016). Based on these properties, several GLP-1 receptor (GLP-1R) agonists have been approved for pharmacological treatment of T2DM (Tomlinson et al. 2016).

In addition to its pancreatic effects, GLP-1R activation decreases gut motility, slows gastric emptying, and promotes satiety (presumably through a combination of GLP-1R activation in the central and peripheral nervous systems), thereby regulating food intake and body weight (Baggio and Drucker 2007). The US Food and Drug Administration (FDA) and the European Medicines Agency (EMA) approved the GLP-1R agonist liraglutide for the treatment of overweight and obesity (SAXENDA package insert, 2014; SAXENDA summary of product characteristics, 2015).

Preclinical data indicate that GIP also exerts effects on appetite regulation and food intake as well as on adipose tissue and peripheral energy metabolism. Although studies evaluating effects of GIP on body weight have yielded equivocal results, GIP receptor (GIPR) activation may play

a role in body weight regulation and targeting both the GLP-1R and the GIPR simultaneously could potentially result in additive or synergistic effects of the 2 incretins on body weight (Coskun et al. 2018).

Tirzepatide is a 39-amino acid synthetic peptide with agonist activity at both the GIPR and GLP-1R. Its structure is based on the GIP sequence and includes a C20 fatty diacid moiety (Coskun et al. 2018). It is administered once-weekly (QW) by subcutaneous (SC) injection. As a dual GIP/GLP-1R agonist, tirzepatide could exceed the efficacy of selective GLP-1R agonists by recruiting metabolically active tissues not targeted by selective GLP-1R agonists (for example, adipose tissue as indicated by the observation of increased energy utilization) (Müller et al. 2018) and has the potential to reach higher efficacy in target tissues that express both GIPR and GLP-1R. Therefore, tirzepatide has the potential to impact several aspects of energy regulation and be a treatment for overweight and obesity.

There is widespread consensus that pharmacotherapy for weight management should only be used as an adjunct to lifestyle-based approaches (NHLBI 2000; Jensen et al. 2014; Garvey et al. 2016). There seems to be less clarity, however, on when and in whom obesity treatment should be advanced from lifestyle-based monotherapy to lifestyle plus medication combination therapy. While some guidelines recommend pharmacotherapy only after diet and exercise fail to achieve weight loss over 6 months (NHLBI 2000), others add that it should also be considered if initial weight loss is not maintained over time (Jensen et al. 2014), and still others imply that even responders to diet and exercise may benefit from additional weight loss with medications (Garvey et al. 2016). There is a need to better understand the benefits of tirzepatide after initial success with lifestyle-induced weight loss, especially as current recommendations vary and goals of treatment in such patients may range from promoting maintenance to augmenting weight loss.

Study 18F-MC-GPHM is a Phase 3, multicenter, randomized, parallel-arm, double-blind, placebo-controlled, 84-week study that will investigate the effects of treatment with maximum tolerated dose (MTD) of tirzepatide (10 mg or 15 mg QW), compared with placebo, on body weight management in study participants who have obesity (body mass index [BMI] $\geq$30 kg/m^2) or are overweight (BMI $\geq$27 kg/m^2) with at least one weight-related comorbid condition and achieved $\geq$5.0% weight loss after a 12-week lead-in period with an intensive lifestyle modification program.

2.2. Background

There remains an unmet need in the pharmacologic treatment of obesity for drugs that are safe, efficacious, and well tolerated. There are currently only a few medications FDA-approved for long-term use for the treatment of obesity that yield a placebo-adjusted average weight loss between 3% and 7% (Srivastava and Apovian 2018; FDA 2020). Although moderate weight loss of 5% to 10% in individuals with obesity/overweight has long been shown to yield significant metabolic benefits including improvements in cholesterol, blood pressure, and glucose parameters (Goldstein 1992; Wing et al. 2011), greater weight loss can maximize these benefits and may be required to realize clinically meaningful improvements in other obesity-related comorbidities such as sleep apnea, non-alcoholic steatohepatitis, and cardiovascular disease (Ryan and Yockey 2017). In addition to moderate efficacy, some centrally-acting weight-loss agents to date have had adverse neurocognitive, psychiatric or cardiovascular effects, further limiting their application in clinical practice (Srivastava and Apovian 2018).

Weight loss induced by GLP-1R agonists, while appearing to be centrally-mediated through a combination of hormonal inputs to satiety centers (van Bloemendaal et al. 2014), has not been consistently associated with changes in mental health or with potential for addiction in long-term studies conducted to establish cardiovascular safety in patients with diabetes (Marso et al. 2016a, 2016b, Gerstein et al. 2019). Tirzepatide, which is both a GLP-1R and GIPR agonist, has been associated with predominantly mild to moderate gastrointestinal (GI) adverse effects similar to currently marketed GLP-1R agonists, but has also demonstrated significant weight loss at both the 10-mg and 15-mg doses in Phase 2 studies with nearly half of the participants in each dose arm achieving ≥10% weight loss (Frias et al. 2018).

Tirzepatide dose selection for obesity treatment has been informed by 3 clinical trials: a Phase 1 study, Study I8F-MC-GPGA (GPGA), and 2 Phase 2 studies, Study I8F-MC-GPGB (GPGB) and I8F-MC-GPGF (GPGF).

Phase 1 Study GPGA was a combination of single-ascending dose (SAD) and multiple-ascending dose (MAD) study in healthy subjects followed by a multiple-dose study in patients with T2DM. A total of 142 participants (89 healthy subjects and 53 patients with T2DM) received at least 1 dose of treatment. Doses of tirzepatide ranged from:

- 0.25 mg to 8 mg in the SAD (with maximum tolerated dose [MTD] achieved at 5 mg) in healthy subjects,
- multiple doses in the MAD from 0.5 mg to 4.5 mg QW and titrated doses up to 10 mg QW for 4 weeks in healthy subjects, and
- multiple doses at 0.5 mg and 5 mg QW and titrated to 15 mg QW for 4 weeks in patients with T2DM.

The safety and tolerability and pharmacokinetic/pharmacodynamic (PK/PD) profiles of tirzepatide at doses and escalation regimens administered in this Phase 1 study supported further development of tirzepatide for QW dosing in patients with T2DM.

Phase 2 studies GPGB and GPGF provided initial safety, tolerability and efficacy data in the tirzepatide 1-mg to 15-mg dose-range when used in treatment of patients with T2DM. In the dose range of 5 mg to 15 mg, tirzepatide provided significantly greater reductions in hemoglobin A1c (HbA1c) and body weight compared with placebo. In addition, the 10-mg and 15-mg dose arms demonstrated significantly greater weight loss compared to the GLP-1R agonist, dulaglutide 1.5 mg QW. The most common adverse events (AEs), which were also dose-dependent, were mild-to-moderate nausea, vomiting, and diarrhea. Study GPGF showed that adjustments in the tirzepatide dose-escalation algorithm resulted in additional reductions in the frequency of GI AEs and reduced the frequency of treatment discontinuations due to GI AEs.

2.3. Benefit/Risk Assessment

More detailed information about the known and expected benefits and risks and reasonably expected AEs of tirzepatide may be found in the Investigator’s Brochure (IB).

3. Objectives and Endpoints

Objectives	Endpoints
**Primary**	
To demonstrate that tirzepatide MTD is superior to placebo from randomization for the following (measured at 72 weeks)<br>• Percent change in body weight AND<br>• Proportion of participants with ≥5% body weight reduction	• Mean percent change in body weight<br>• Percentage of study participants who achieve ≥5% body weight reduction
**Key Secondary (controlled for type I error)**	
To demonstrate that tirzepatide MTD is superior to placebo from randomization for the following (measured at 72 weeks):<br>• Maintaining body weight reduction achieved during the 12-week lead-in period	• Percentage of study participants who maintain ≥80% of the body weight lost during the 12-week lead-in period
• Body weight	• Percentage of study participants who achieve:<br>o ≥10% body weight reduction<br>o ≥15% body weight reduction
• Waist circumference	• Mean change in waist circumference (cm)
**Additional Secondary**	
To demonstrate that tirzepatide MTD is superior to placebo from randomization for the following (measured at 72 weeks):<br>• Body weight and BMI	• Mean change in body weight (kg)<br>• Mean change in BMI (kg/m^2)
• Blood pressure	• Mean change in<br>o systolic blood pressure (mmHg)<br>o diastolic blood pressure (mmHg)
• Lipid parameters	• Mean change in:<br>o Total cholesterol (mg/dL)<br>o HDL-cholesterol (mg/dL)<br>o LDL-cholesterol (mg/dL)<br>o VLDL-cholesterol (mg/dL)<br>o Triglycerides (mg/dL)

Objectives	Endpoints
	o Free fatty acids (mg/dL)
• Glycemic control	• Mean change in<br>o fasting glucose (mg/dL)<br>o HbA1c (%)
• Insulin	• Mean change in fasting insulin (pmol/L)
• Patient-reported outcomes	• Mean change in<br>o SF-36v2 acute form Physical Functioning domain score<br>o IWQOL-Lite-CT Physical Function composite score
To demonstrate that tirzepatide MTD is superior to placebo from Visit 2 for the following (measured at 72 weeks):	
• Body weight and BMI	• Mean change in absolute body weight (kg)<br>• Mean percent change in body weight<br>• Mean change in BMI (kg/m^2)
• Waist circumference	• Mean change in waist circumference (cm)

Abbreviations: BMI = body mass index; HbA1c = hemoglobin A1c; HDL = high-density lipoprotein; IWQOL-Lite-CT = Impact of Weight on Quality of Life-Lite Clinical Trials Version; LDL = low-density lipoprotein, MTD = maximum tolerated dose; SF-36v2 acute form = Short Form-36 Version 2 Health Survey acute form; VLDL = very low-density lipoprotein.

4. Study Design

4.1. Overall Design

Study 18F-MC-GPHM is a Phase 3, multicenter, randomized, parallel-arm, double-blind placebo-controlled, 84-week study that will investigate the effects of treatment with MTD of tirzepatide (10 mg or 15 mg QW), compared with placebo, on body weight management in study participants who have obesity (BMI ≥30 kg/m^2) or are overweight (BMI ≥27 kg/m^2) with at least one weight-related comorbid condition and achieved ≥5.0% weight loss after a 12-week lead-in period with an intensive lifestyle modification program.

Study GPHM will consist of 4 periods: a 2-week screening period; a 12-week lead-in period during which participants undertake an intensive lifestyle modification program to achieve ≥5.0% body weight loss; a 72-week double-blind placebo-controlled treatment period (including a 20-week dose escalation period); and a 4-week safety follow-up period. Study participants who achieve at least 5.0% weight loss will be randomized in a 1:1 ratio (tirzepatide MTD QW or placebo) at the end of the lead-in period. An upper limit of 70% enrollment of women will be used to ensure a sufficiently large sample of men.

4.1.1. Overview of Study Periods

4.1.1.1. Visit Structure for all Office Visits

On all designated fasting office visits, study participants are required to report to the site in a fasting condition, after a period of approximately 8 hours without eating, drinking (except water), or performing any significant physical activity. If a participant is adversely affected by the fasting condition, they are allowed to eat; however, specific study procedures need to be completed while fasting. See Section 10.8 for a suggested order of activities that occurs at office visits.

4.1.1.2. Main Study Period

4.1.1.2.1. Screening Period

Visit 1

The purpose of screening procedures at Visit 1 is to establish initial eligibility, and to obtain blood samples for laboratory assessments needed to confirm eligibility. The participant must sign the informed consent form (ICF) before the study procedures are performed, as outlined in the SoA, Section 1.3. Since some screening procedures need to be completed in the fasting state (approximately 8 hours without eating, drinking [except water], or any significant physical activity), Visit 1 may be conducted over more than 1 day to ensure necessary conditions are met. Participants who meet all applicable inclusion criteria and none of the applicable exclusion criteria at Visit 1 will continue to Visit 2.

The Mental Health questionnaires (Patient Health Questionnaire-9 [PHQ-9], Columbia-Suicide Severity Rating Scale [C-SSRS], and C-SSRS Self-Harm Form) should be completed after the assessment for AEs.

Visit 2

At Visit 2, the screening laboratory results will be reviewed. For all participants who remain eligible, lifestyle program instruction will be provided by a dietician, or equivalent qualified delegate, for an intensive dietary and exercise program.

Patient-reported outcomes (PROs) questionnaires should be administered as early as possible, as per Suggested Visit Structure (Section 10.8). Preferred administration order is:

1. Patient Global Impression of status for physical activity (PGIs)
2. Short Form-36 version 2 Health Survey (SF-36v2) acute form
3. Impact of Weight on Quality of Life-Lite-Clinical Trials Version (IWQOL-Lite-CT)
4. EQ-5D-5L.

The Mental Health questionnaires should be completed after the assessment for AEs.

4.1.1.2.2. Intensive Lifestyle Modification Lead-in Period

During the intensive lifestyle modification lead-in period (Visits 2-10), participants will receive instruction from a dietician, or equivalent qualified delegate, according to local standards, to reduce their daily caloric energy intake to approximately

- 1200 kcal/day for women, or
- 1500 kcal/day for men

for a period of 12 weeks (Heymsfield et al. 2003). The dietary intervention may include up to 2 meal replacements per day. Participants will be encouraged to exercise on a regular basis, with a recommendation of at least 150 minutes per week of moderate intensity activity (for example, brisk walking). Participants will be counselled on behavior modification strategies to help implement and adhere to the diet and exercise recommendations. For more details see Sections 5.3.1 and 5.3.2.

Participants who have ≥5.0% weight loss at Visit 10 will then proceed to randomization to either tirzepatide or placebo.

Mental Health questionnaires should be completed after the assessment of AEs.

4.1.1.2.3. Treatment Period

Visit 10 (Randomization)

At Visit 10, eligible participants will perform all required randomization study procedures (including the collection of all laboratory measures and questionnaires) prior to randomization and prior to taking the first dose of study drug. Only participants who have achieved ≥5.0% weight loss at Visit 10 will be randomized and continue in the study.

Participants will be provided diaries and be trained to record key study information, as appropriate.

PRO questionnaires should be administered as early as possible, as per Suggested Visit Structure (Section 10.8). Preferred administration order is:

1. PGIs

2. SF-36 v2 acute form
3. IWQOL-Lite-CT
4. EQ-5D-5L.

Mental Health questionnaires should be completed after the assessment of AEs.

Participants will receive consultation with a dietician or qualified delegate, according to local standards, to set lifestyle goals for caloric intake and physical activity (Section 5.3) during the treatment period.

Following randomization, study site personnel will demonstrate use of the autoinjector (also referred to as single-dose pen) using the provided demonstration device and observe the study participant inject the first dose of tirzepatide or placebo. The date, time and location of the first dose of study drug will be recorded on the electronic case report form (eCRF). Beginning at randomization, all participants will receive study drug according to the randomized treatment arm for the duration of the 72-week dose escalation and treatment period.

End of Visit 10 to Visit 28

During the treatment period, office visits will occur as indicated in the SoA (Section 1.3). Telephone visits will occur at 4-week intervals between the office visits starting at Visit 17.

Office visit procedures should be conducted according to the SoA (Section 1.3), and will include

- weight, waist circumference and vital signs measurements
- laboratory testing
- administration of PRO questionnaires
- collection of AEs, product complaints and concomitant medications
- Mental Health questionnaires
- review of participant diary information (to include reinforcement and compliance assessments for study drug administration and lifestyle goals)
- drug dispensing.

Patient-reported outcomes questionnaires should be administered as early as possible, as per Suggested Visit Structure (Section 10.8). Preferred administration order is the same as Visit 2.

Mental Health questionnaires should be completed after the assessment of AEs.

Dietician consultations occur every 12 weeks from Week 0 to Week 72. Study drug and injection supplies will be returned per the SoA (Section 1.3) and according to local requirements. New supplies will be dispensed as needed.

The starting dose of tirzepatide is 2.5 mg QW (or matching placebo) for 4 weeks, then the dose is increased by 2.5 mg (or matching placebo) every 4 weeks (2.5 to 5 to 7.5 to 10 to 12.5 to 15 mg) up to MTD (either 10 mg or 15 mg).

Interventions to optimize study drug tolerance and adherence may be employed throughout the study and include, but are not limited to, brief temporary interruptions and use of additional medications to manage GI symptoms (for example, nausea, vomiting, and diarrhea, see Section 6.6.2. for details).

During the first 24 weeks of the treatment period (20-week dose escalation plus 4 weeks), participants unable to tolerate 2.5 mg or 5 mg despite the above measures will be discontinued from the study drug but remain in the study for continued follow up. For participants unable to tolerate any dose escalation between 7.5 mg and 15 mg inclusive, despite the above measures, the investigator should contact Lilly to consider a dose de-escalation step with subsequent re-escalation in a blinded fashion to reach either the 10-mg or 15-mg dose as described below (only 1 cycle of dose de-escalation and re-escalation is permitted during the first 24 weeks of the treatment period):

- 7.5 mg to 5 mg, then the dose is increased by 2.5 mg every 4 weeks up to MTD (either 10 mg or 15 mg)
- 10 mg to 7.5 mg, then the dose is increased by 2.5 mg every 4 weeks up to MTD (either 10 mg or 15 mg)
- 12.5 mg to 10 mg, then the dose is increased by 2.5 mg every 4 weeks up to MTD (either 10 mg or 15 mg)
- 15 mg to 12.5 mg, then the dose is increased by 2.5 mg every 4 weeks up to MTD (either 10 mg or 15 mg)

Participants who tolerate 15 mg will continue on 15 mg as their MTD dose.

Participants who tolerate 10 mg, but do not tolerate 12.5 mg or 15 mg even after one de-escalation and re-escalation attempt, will continue on 10 mg as their MTD dose.

Participants who tolerate 12.5 mg but do not tolerate 15 mg even after 1 de-escalation and re-escalation attempt, will continue on 10 mg as their MTD dose.

Participants who do not tolerate up to 10 mg even after 1 de-escalation and re-escalation attempt, will be discontinued from the study drug but remain in the study for continued follow up.

At each of the 8 scheduled treatment period telephone visits, procedures will include

- collection of AEs, product complaints, and concomitant medications
- administration of Mental Health questionnaires, and
- review of participant diary information (to include reinforcement and compliance assessments for study drug administration and lifestyle goals).

Participants should be instructed to contact the investigative site for assistance as soon as possible if they experience any difficulties administering their study medication. Participants should also be advised about the appropriate course of action in the event that study drug is not taken at the required time (late/missing doses).

Visit 99

Visit 99 is only applicable to participants who discontinue the study treatment prematurely (between Visit 10 and Visit 28) and decline to complete the remaining scheduled study visits. These participants will be asked to return for Visit 99 at 72 weeks ±7 days after randomization (Section 7.2.1). This visit is critical to ensure complete data collection for the primary endpoint.

Participants should attend this visit in the fasting state. Procedures to be completed are

- measurement of weight and waist circumference,
- listing of concomitant medications,
- assessment of AEs, and
- completion of the Mental Health questionnaires (after the AE assessment).

For participants unwilling to attend this visit, their refusal to attend should be documented in the participant medical record.

4.1.1.2.4. Early Discontinuation of Treatment Visit

Participants unable or unwilling to continue the study intensive lifestyle intervention (during lead-in period) or study drug intervention (during treatment period) for any reason will perform an early discontinuation (ED) visit (Section 7.2.1). If the participant is discontinuing during an unscheduled visit or a scheduled visit, that visit should be performed as an ED visit. Procedures should be completed according to the SoA. Patient-reported outcomes questionnaires should be administered as early as possible, as per Suggested Visit Structure (Section 10.8). Administration of Mental Health questionnaires should follow assessment of AEs.

4.1.1.3. Safety Follow-Up Period

Visit 801

All randomized participants are required to complete a safety follow-up visit (Visit 801), according to the SoA. Participants discontinuing the study drug early and performing an ED visit will also be asked to perform the safety follow-up visit (see Section 7.2). During the safety follow-up period, participants will not receive study drug. Participants are also required to return any remaining study diaries to the study site at the end of this period.

Visit 801 (safety follow-up visit) should be performed 4 weeks after the last visit in the treatment period (Visit 28 or Visit 99) or 4 weeks after the ED visit for randomized participants who decline to return for Visit 99.

4.1.2. Study Procedures

Participants will perform study procedures listed in the SoA (Section 1.3).

Study participants will be permitted to use concomitant medications that they require during the study, except certain excluded medications (see Section 5.2) that may interfere with the assessment of efficacy and safety characteristics of the study treatments.

Study governance considerations are described in detail in Section 10.1 (Appendix 1).

4.2. Scientific Rationale for Study Design

Tirzepatide is a 39-amino acid synthetic peptide with agonist activity at both the GIPR and GLP-1R. Its structure is based on the GIP sequence and includes a C20 fatty diacid moiety (Coskun et al. 2018). It is administered QW by SC injection.

As a dual GIP/GLP-1R agonist, tirzepatide could exceed the efficacy of selective GLP-1R analogs by recruiting metabolically active tissues not targeted by selective GLP-1R analogs (for example, adipose tissue as indicated by the observation of increased energy utilization) (Müller 2018) and has the potential to reach higher efficacy in target tissues, such as insulin-producing pancreatic beta-cells that express both GIPR and GLP-1R, before reaching its therapeutic limitation. Results from a Phase 2 study (GPGB) demonstrated that tirzepatide use in participants with T2DM was associated with a substantial, dose-dependent weight loss, greater than the weight change observed with dulaglutide, a specific GLP-1R agonist. General safety characteristics of all studied doses of tirzepatide were similar to that of the GLP-1R agonist class, consisting mainly of nausea, vomiting, and diarrhea. In general, these events were transient and mild or moderate in severity, with few severe episodes. These data suggest that tirzepatide has the potential to be a pharmacologic treatment for chronic weight management.

Although GI AEs were more common in the 15-mg arm of tirzepatide in the Phase 2 study (GPGB), this dose demonstrated the highest efficacy in terms of weight loss. An optimized dose escalation regimen proposed in the current Phase 3 Study (GPHM) to improve tolerability (and supported by a dose-escalation algorithm from study GPGF and PK/PD modelling) should enable a use of the 15-mg dose to maximize effects on body weight.

Study GPHM is designed to determine the impact of treatment with tirzepatide MTD (10 mg or 15 mg QW) compared with placebo on body weight management after an intensive lifestyle modification program in study participants without T2DM who have obesity or are overweight.

Study GPHM consists of a lead-in period of 12 weeks during which participants will undertake an intensive lifestyle modification program (Heymsfield et al. 2003). Traditionally, guidelines have restricted pharmacotherapy use to those who do not respond to diet and exercise but newer guidelines are recommending the addition of pharmacotherapy in those who lose weight with diet and exercise but may not be able to maintain the weight loss or may require additional weight loss. The goal of the lead-in period, therefore, is to identify participants who initially respond to lifestyle-based therapy so that the benefit of tirzepatide can be evaluated in this population. Only those participants who achieve ≥5.0% weight loss during the intensive lifestyle lead-in period will be randomized.

A low-calorie diet (LCD) was chosen for the lead-in period because it is a discrete, structured and intensive intervention that is recognized as the centerpiece of dietary therapy for overweight and obesity (NHLBI 2000). In those who can adhere to it, an LCD may achieve greater weight loss in the short-term than the moderate 500 kcal/day deficit diet being applied in combination with tirzepatide throughout the obesity Phase 3 program. Moreover, an LCD was chosen over the option of a very low-calorie diet (VLCD) because VLCDs are only used in limited circumstances by specialized practitioners experienced in their use and with special monitoring and supplementation (NHLBI 2000). Because VLCDs are not recommended as routine therapy for overweight or obesity, using a VLCD would have affected the generalizability of the results.

Clinical trials have also shown that LCDs are as effective as VLCDs at 1 year in achieving weight loss (Wadden et al. 1994).

After randomization, participants in both the placebo and MTD tirzepatide arms will continue to receive lifestyle modification counselling consistent with current guidelines for weight management (Jensen et al. 2014; NHLBI 2013). Specifically, participants will consult with a dietician, or equivalent qualified delegate, throughout the post-randomization treatment period to achieve an approximately 500 kcal/day energy deficit through a combination of caloric restriction and increased physical activity (see Section 5.3).

The planned duration of treatment for the primary endpoint at 72 weeks allows for a 52-week treatment period at the dose achieved following dose escalation to either 10 mg or 15 mg. This duration is considered appropriate to assess the full effects and balance of risk and benefit for tirzepatide MTD compared with placebo on body weight and is consistent with regulatory guidelines (FDA 2007; EMA 2016). A placebo comparator was selected for this trial in accordance with regulatory guidance (FDA 2007; EMA 2016).

The effects of drug cessation will be assessed in the 4-week safety follow-up/observational period. To minimize the potential confounding effect of changes to concomitant medications, participants will be permitted to use concomitant medications that do not interfere with the assessment of efficacy and safety characteristics of the study treatments.

4.3. Justification for Dose

Tirzepatide MTD (10 mg or 15 mg QW) will be evaluated in this study. These doses and associated escalation schemes were selected based on assessment of safety, efficacy (weight loss), and GI tolerability data in Phase 1 and 2 studies in patients with T2DM, followed by exposure-response modeling of the data that predicted weight loss in patients with overweight or obesity.

In a 26-week, Phase 2 study (GPGB) of participants with T2DM, tirzepatide reduced body weight by 4.8 kg, 8.7 kg, and 11.3 kg at dose levels of 5, 10, and 15 mg, respectively, whereas the weight loss observed in participants on dulaglutide, a selective GLP-1R agonist, used at the dose of 1.5 mg, was 2.7 kg (Frias et al. 2018).

Similar to the GLP-1R agonist class, most of the tirzepatide AEs were dose-dependent and GI-related, consisting mainly of nausea, vomiting, and diarrhea. In general, these events were mild or moderate in severity, with few severe episodes, and transient.

Tirzepatide doses of 10 mg and 15 mg were selected based principally on the following criteria:

- each dose provides robust weight loss relative to placebo
- the percent of patients achieving ≥10% weight loss is higher with 15 mg than 10 mg, and
- safety and tolerability were supported by Phase 2 results and/or PK/PD modeling.

Dosing algorithms starting at a low dose of 2.5 mg accompanied by dose escalation of 2.5-mg increments every 4 weeks should permit time for development of tolerance to GI events and are predicted to minimize GI tolerability concerns. The maximum proposed dose of 15 mg maintains an exposure multiple of 1.6 to 2.4 to the no-observed adverse effect level doses in 6-month monkey and rat toxicology studies.

4.4. End of Study Definition

Section 7.2 describes the criteria used to determine if a participant has completed the study.

The end of the study is defined as the date of the last visit of the last participant in the study or last scheduled procedure shown in the SoA for the last participant in the trial globally.

5. Study Population

Prospective approval of protocol deviations to recruitment and enrollment criteria, also known as protocol waivers or exemptions, *is not permitted.*

5.1. Inclusion Criteria

Participants are eligible to be included in the study only if all of the following criteria apply:

Type of Participant and Disease Characteristics

1. Have a BMI of:
    - ≥30 kg/m^2 or
    - ≥27 kg/m^2 and previously diagnosed with at least 1 of the following weight-related comorbidities
        - Hypertension: treated or with systolic blood pressure ≥130 mmHg or diastolic blood pressure ≥80 mmHg
        - Dyslipidemia: treated or with low-density lipoprotein (LDL) ≥160 mg/dL (4.1 mmol/L) or triglycerides ≥150 mg/dL (1.7 mmol/L), or high-density lipoprotein (HDL) <40 mg/dL (1.0 mmol/L) for men or HDL <50 mg/dL (1.3 mmol/L) for women
        - Obstructive sleep apnea
        - Cardiovascular disease (for example, ischemic cardiovascular disease, New York Heart Association [NYHA] Functional Classification Class I-III heart failure)
2. Have a history of at least 1 self-reported unsuccessful dietary effort to lose body weight
3. In the investigator’s opinion, are well-motivated, capable, and willing to:
    - learn how to self-inject study drug, as required for this protocol (visually impaired persons who are not able to perform the injections must have the assistance of a sighted individual trained to inject the study drug; persons with physical limitations who are not able to perform the injections must have the assistance of an individual trained to inject the study drug)
    - inject study drug (or receive an injection from a trained individual if visually impaired or with physical limitations)
    - follow study procedures for the duration of the study, including, but not limited to following lifestyle advice (for example, dietary restrictions, exercise plan), maintaining a study diary, and completing required questionnaires

Participant Characteristics

4. Are at least 18 years of age and age of majority per local laws and regulations
    a. Male participants:

- Male participants with partners of childbearing potential should be willing to use reliable contraceptive methods throughout the study and for 5 half-lives of study drug plus 90 days, corresponding to 4 months after the last injection.

b. Female participants:

- Female participants not of childbearing potential may participate and include those who are:
    - infertile due to surgical sterilization (hysterectomy, bilateral oophorectomy, or tubal ligation) or congenital anomaly (such as Mullerian agenesis) or
    - Post-menopausal – defined as either:
        - A woman at least 40 years of age with an intact uterus, not on hormone therapy, who has cessation of menses for at least 1 year without an alternative medical cause, AND a follicle-stimulating hormone (FSH) ≥40 mIU/mL; women in this category must test negative in pregnancy test prior to study entry

    or

        - A woman 55 or older not on hormone therapy, who has had at least 12 months of spontaneous amenorrhea

    or

        - A woman at least 55 years of age with a diagnosis of menopause prior to starting hormone replacement therapy

- Female participants of child-bearing potential (not surgically sterilized and between menarche and 1-year postmenopausal) must:
    - test negative for pregnancy at Visit 1 based on a serum pregnancy test
    - if sexually active, agree to use 2 forms of effective contraception, where at least 1 form is highly effective for the duration of the trial plus 30 days, corresponding to 2 months after the last injection, and
    - not be breastfeeding

Note: Contraceptive use by men or women should be consistent with local regulations regarding the methods of contraception for those participating in clinical studies.

Informed Consent

5. Capable of giving signed informed consent as described in Section 10.1 (Appendix 1), which includes compliance with the requirements and restrictions listed in the ICF and in this protocol.

5.2. Exclusion Criteria

Participants are excluded from study enrollment if they meet any of the following criteria at screening:

Medical Conditions

Diabetes-Related

6. Have Type 1 diabetes mellitus (T1DM) or T2DM, history of ketoacidosis, or hyperosmolar state/coma
7. Have at least 1 laboratory value suggestive of diabetes mellitus during screening, including 1 or more of: HbA1c ≥6.5% (≥48 mmol/mol), fasting glucose ≥126 mg/dL (≥7.0 mmol/L), or random glucose ≥200 mg/dL (≥11.1 mmol/L)

Obesity-Related

8. Have a self-reported change in body weight >5 kg within 3 months prior to screening
9. Have a prior or planned surgical treatment for obesity (excluding liposuction or abdominoplasty, if performed >1 year prior to screening)
10. Have or plan to have endoscopic and/or device-based therapy for obesity or have had device removal within the last 6 months prior to screening
    - mucosal ablation
    - gastric artery embolization
    - intragastric balloon
    - duodenal-jejunal endoluminal liner

Other Medical

11. Have renal impairment measured as estimated glomerular filtration rate (eGFR) <30 mL/min/1.73 m^2, calculated by Chronic Kidney Disease-Epidemiology (CKD-EPI) as determined by central laboratory during screening
12. Have a known clinically significant gastric emptying abnormality (for example, severe gastroparesis or gastric outlet obstruction) or chronically take drugs that directly affect GI motility
13. Have a history of chronic or acute pancreatitis
14. Have thyroid-stimulating hormone (TSH) outside of the range of 0.4-6.0 mIU/L at the screening visit

    Note: Participants receiving treatment for hypothyroidism may be included, provided their thyroid hormone replacement dose has been stable for at least 3 months and their TSH at screening falls within the range indicated above.

Note: Participants with a history of subclinical hypothyroidism but a TSH at screening within the range indicated above, may be included if in the investigator's opinion, the patient is unlikely to require initiation of thyroid hormone replacement during the course of the study.

15. Have obesity induced by other endocrinologic disorders (for example, Cushing syndrome) or diagnosed monogenetic or syndromic forms of obesity (for example, Melanocortin 4 Receptor deficiency or Prader Willi Syndrome)

16. Have a history of significant active or unstable Major Depressive Disorder (MDD) or other severe psychiatric disorder (for example, schizophrenia, bipolar disorder, or other serious mood or anxiety disorder) within the last 2 years

    Note: Participants with MDD or generalized anxiety disorder whose disease state is considered stable for the past 2 years and expected to remain stable throughout the course of the study, in the opinion of the investigator, may be considered for inclusion if they are not on excluded medications

17. Have a lifetime history of suicide attempt

18. Have a PHQ-9 score of 15 or more at Visit 1

19. On the C-SSRS at any time from Visit 1 to Visit 2:

    - a "yes" answer to Question 4 (Active Suicidal Ideation with Some Intent to Act, Without Specific Plan) on the "Suicidal Ideation" portion of the C-SSRS

    or

    - a "yes" answer to Question 5 (Active Suicidal Ideation with Specific Plan and Intent) on the "Suicidal Ideation" portion of the C-SSRS

    or

    - a "yes" answer to any of the suicide-related behaviors (actual attempt, interrupted attempt, aborted attempt, preparatory act or behavior) on the "Suicidal Behavior" portion of the C-SSRS

    and

    - the ideation or behavior occurred within the past month

20. Have uncontrolled hypertension (systolic blood pressure above or equal to 160 mmHg and/or diastolic blood pressure above or equal to 100 mmHg)

21. Have any of the following cardiovascular conditions within 3 months prior to Visit 2:
    - acute myocardial infarction
    - cerebrovascular accident (stroke)
    - unstable angina, or
    - hospitalization due to congestive heart failure (CHF)

22. Have NYHA Functional Classification Class IV CHF

23. Have acute or chronic hepatitis, signs and symptoms of any other liver disease other than nonalcoholic fatty liver disease (NAFLD), or any of the following, as determined by the central laboratory during screening:
    - alanine aminotransferase (ALT) level >3.0X upper limit of normal (ULN) for the reference range
    - alkaline phosphatase (ALP) level >1.5X ULN for the reference range, or
    - total bilirubin (TBL) level >1.2X ULN for the reference range (except for cases of known Gilbert's Syndrome)

    Note: Participants with NAFLD <u>are eligible</u> to participate in this trial if their ALT level is ≤3.0X ULN for the reference range.

24. Have a serum calcitonin level (at Visit 1) of
    - ≥20 ng/L, if eGFR ≥60 mL/min/1.73 m^2
    - ≥35 ng/L, if eGFR <60 mL/min/1.73 m^2

25. Have a family or personal history of medullary thyroid carcinoma (MTC) or multiple endocrine neoplasia (MEN) syndrome type 2

26. Have a history of an active or untreated malignancy or are in remission from a clinically significant malignancy (other than basal or squamous cell skin cancer, in situ carcinomas of the cervix, or in situ prostate cancer) for less than 5 years

27. Have any other condition not listed in this section (for example, hypersensitivity or intolerance) that is a contraindication to GLP-1R agonists

28. Have a history of any other condition (such as known drug or alcohol abuse, diagnosed eating disorder, or other psychiatric disorder) that, in the opinion of the investigator, may preclude the participant from following and completing the protocol

29. Have history of use of marijuana or tetrahydrocannabinol (THC)-containing products within 3 months of enrollment or unwillingness to abstain from marijuana or THC-containing products use during the trial

    Note: If a participant has used cannabidiol oil during the past 3 months but agrees to refrain from use for the duration of the study, the participant can be enrolled.

30. Have had a transplanted organ (corneal transplants [keratoplasty] are allowed) or are awaiting an organ transplant

31. Have any hematological condition that may interfere with HbA1c measurement (for example, hemolytic anemias, sickle cell disease)

Prior/Concomitant Therapy

32. Are receiving or have received within 3 months prior to screening chronic (>2 weeks or 14 days) systemic glucocorticoid therapy (excluding topical, intra-ocular, intranasal, intra-articular, or inhaled preparations) or have evidence of a significant, active autoimmune abnormality (for example, lupus or rheumatoid arthritis) that has required

(within the last 3 months) or is likely to require, in the opinion of the investigator, concurrent treatment with systemic glucocorticoids (excluding topical, intra-ocular, intranasal, intra-articular, or inhaled preparations) during the course of the study

33. Have current treatment with or history of (within 3 months prior to Visit 2) treatment with medications that may cause significant weight gain, including but not limited to: tricyclic antidepressants, atypical antipsychotics, and mood stabilizers

Examples:

- imipramine
- amitriptyline
- mirtazapine
- paroxetine
- phenelzine
- chlorpromazine
- thioridazine
- clozapine
- olanzapine
- valproic acid (and its derivatives), or
- lithium

Note: Selective serotonin reuptake inhibitors (SSRIs) other than paroxetine are permitted.

34. Have taken, within 3 months prior to Visit 2, medications (prescribed or over-the-counter) or alternative remedies that promote weight loss

Examples include, but are not limited to:

- Saxenda (liraglutide 3.0 mg)
- Xenical®/Alli® (orlistat)
- Meridia® (sibutramine)
- Acutrim® (phenylpropanolamine)
- Sanorex® (mazindol)
- Apidex® (phentermine)
- BELVIQ® (lorcaserin)
- Bontril® (phendimetrazine)
- Qsymia™ (phentermine/topiramate combination)
- Contrave® (naltrexone/bupropion)

Note: Use of metformin, or any other glucose-lowering medication, whether prescribed for polycystic ovarian syndrome (PCOS) or diabetes prevention is not permitted.

35. Have started implantable or injectable contraceptives (such as Depo Provera®) within 18 months prior to screening

Prior/Concurrent Clinical Study Experience

36. Are currently enrolled in any other clinical study involving an investigational product (IP) or any other type of medical research judged not to be scientifically or medically compatible with this study
37. Within the last 30 days, have participated in a clinical study and received treatment, whether active or placebo. If the study involved an IP, 5 half-lives or 30 days, whichever is longer, should have passed
38. Have previously completed or withdrawn from this study or any other study investigating tirzepatide after receiving at least 1 dose of IP

Other Exclusions

39. Are investigator site personnel directly affiliated with this study and/or their immediate families. Immediate family is defined as a spouse, parent, child, or sibling, whether biological or legally adopted
40. Are Lilly employees

5.3. Lifestyle Considerations

Per the SoA (Section 1.3), participants will consult with a dietician, or equivalent qualified delegate, according to local standards, to receive lifestyle management counseling at Weeks -12, -11, -10, -9, -8, -6, -4 and -2 prior to randomization and then at Weeks 0, 12, 24, 36, 48, 60 and 72 post-randomization.

Diet and exercise goals established during the lifestyle consultation and the importance of adherence to the lifestyle component of the trial will be reinforced at each trial contact by study staff.

5.3.1. Meals and Dietary Restrictions

During the intensive lifestyle modification lead-in period, participants will receive instruction from a dietician, or equivalent qualified delegate, to reduce their daily caloric energy intake to approximately 1200 kcal/day for women or 1500 kcal/day for men for 12 weeks. During this lead-in period, up to 2 liquid meal replacements per day are permitted, but not required, to achieve the targeted energy deficit. In addition to the diet modification, participants will be encouraged to exercise on a regular basis, with a recommendation of at least 150 minutes per week of moderate intensity activity (for example, brisk walking). Participants will be counseled on behavior modification strategies to help implement and adhere to the diet and exercise recommendations. Participants who achieve a ≥5.0% body weight loss at the end of the 12-week lead-in period (Week 0) will proceed to randomization to either tirzepatide or placebo.

After randomization, participants will be advised to maintain their daily energy intake at 500 kcal below their individualized energy requirements (Garvey et al. 2016), as calculated by the Food and Agriculture Organization of the United Nations/World Health Organization [WHO]/United Nations University (FAO/WHO/UNU) estimates of human energy requirements, using a “sedentary” physical activity level (PAL) of 1.3 (FAO/WHO/UNU 2004).

Participants will be encouraged to maintain moderate intensity physical activity of at least 150 minutes per week. Liquid meal replacement is permitted but will not be provided during the post-randomization treatment period. The recommended macronutrient distribution will be

- maximum 30% of energy from fat
- approximately 20% of energy from protein
- approximately 50% of energy from carbohydrates, and
- an energy deficit of approximately 500 kcal/day compared to the participant's estimated total energy expenditure (TEE).

To encourage adherence, it is recommended that behavior modification tools be used, such as completion of a 3-day diet and exercise log prior to each counseling visit. During each visit, the participant's diet will be reviewed and behavior modification strategies to maximize adherence will be provided as needed.

The hypocaloric diet will be continued throughout the treatment period. If a BMI $\leq$22 kg/m^2 is reached, the recommended energy intake should be recalculated with no kcal deficit for the remainder of the trial.

Total energy expenditure is calculated by multiplying the estimated Basal Metabolic Rate (BMR) (see table below) with a PAL value of 1.3 (FAO/WHO/ UNU 2004), which reflects an inactive lifestyle. This calculation provides a conservative estimate of caloric requirements:

$$\text{TEE (kcal/day)} = \text{BMR X 1.3}$$

Equations for Estimating BMR in kcal/Day*

Sex	Age	BMR (kcal/day)
Men	18-30 years	15.057 X actual weight in kg + 692.2
	31-60 years	11.472 X actual weight in kg + 873.1
	>60 years	11.711 X actual weight in kg + 587.7
Women	18-30 years	14.818 X actual weight in kg + 486.6
	31-60 years	8.126 X actual weight in kg + 845.6
	>60 years	9.082 X actual weight in kg + 658.5

Abbreviation: BMR = basal metabolic rate; WHO = World Health Organization.
*Revised WHO equations (adapted from: FAO/WHO/UNU 2004).

5.3.2. Physical Activity

At Visit 2 and all subsequent visits, participants will be advised to increase their physical activity to moderate intensity (for example, brisk walking) for at least 150 minutes per week.

5.4. Screen Failures

Screen failures are defined as participants who consent to participate in the clinical study but are not subsequently enrolled in the study. A minimal set of screen failure information is required to ensure transparent reporting of screen failure participants to meet the Consolidated Standards of Reporting Trials (CONSORT) publishing requirements and to respond to queries from regulatory authorities. Minimal information includes demography, screen failure details, eligibility criteria, and any serious adverse event (SAE).

Individuals who do not meet the criteria for participation in this study (screen failure) may not be rescreened.

6. Study Intervention

Study intervention is defined as any study drug intervention, investigational intervention(s), marketed product(s), placebo, or medical device(s) intended to be administered to/used by a study participant according to the study protocol.

6.1. Study Intervention(s) Administered

**Arm Name**	Tirzepatide MTD	Placebo
**Dose**	10 mg or 15 mg QW	N/A
**Route of Administration**	SC	
**Sourcing**	Provided centrally by the sponsor and dispensed via IWRS	
**Packaging and Labeling**	Study drug intervention will be provided in autoinjectors (single-dose pens) packaged in cartons to be dispensed. Clinical study materials will be labeled according to country regulatory requirements.	

Abbreviations: IWRS = interactive web-response system; MTD = maximum tolerated dose; N/A = not applicable; QW = once-weekly; SC = subcutaneous.

There are no restrictions on the time of day each weekly dose of study drug is given, but it is advisable to administer the SC injections on the same day and same time each week. The actual date, time, and injection site location of all dose administrations will be recorded in the diary by the participant. If a dose of study drug is missed, the participant should take it as soon as possible unless it is within 72 hours of the next dose, in which case, that dose should be skipped and the next dose should be taken at the appropriate time. The day of weekly administration can be changed if necessary, as long as the last dose was administered 72 or more hours before.

All participants will inject study drug SC in the abdomen or thigh using the injection supplies provided; a caregiver may administer the injection in the participant's upper arm. The injection-site location of all dose administrations will be recorded in the diary by the participant. A new autoinjector will be used for each injection. If study drug is to always be injected in the same body region, participants should be advised to rotate injection sites each week.

6.1.1. Medical Devices

The combination products provided for use in the study are tirzepatide investigational autoinjector (or matching placebo). Any AE resulting from device deficiencies, misuse, or malfunctions must be detected, documented, and reported by the investigator throughout the study (see Section 10.4.3).

6.2. Preparation/Handling/Storage/Accountability

- The investigator or designee must confirm appropriate storage conditions have been maintained during transit for all study drug intervention received and any discrepancies are reported and resolved before use of the study drug intervention.
- Only participants randomized in the study may receive study drug intervention. Only study personnel may supply, prepare, or administer study drug intervention. All study drug intervention must be stored in a secure, environmentally controlled, and monitored (manual or automated) area in accordance with the labeled storage conditions with access limited to the investigator and authorized study personnel.
- The investigator or authorized study personnel are responsible for study drug intervention accountability, reconciliation, and record maintenance (for example, receipt, reconciliation, and final disposition records).
- Further guidance and information for the final disposition of unused study drug interventions are provided in the study training materials.

6.3. Measures to Minimize Bias: Randomization and Blinding

This is a double-blind, randomized study.

Participants who meet all criteria for enrollment will undergo a 12-week intensive lifestyle modification lead-in period. Those who achieve ≥5.0% weight loss at Visit 10 will be randomized to one of the study treatment groups (tirzepatide MTD or placebo) for the duration of the treatment period. Assignment to treatment groups will be determined by a computer-generated random sequence using an IWRS. Participants will be randomized in a 1:1 ratio to receive tirzepatide MTD or placebo.

The randomization will be stratified by country, sex (female, male), and percent weight loss at the end of lead-in (<10% versus ≥10%).

Investigators, site staff, clinical monitors and participants will remain blinded to the treatment assignments until the study is complete.

Emergency unblinding for AEs may be performed through the IWRS. This option may be used ONLY if the participant's well-being requires knowledge of the participant's treatment assignment. All emergency unblinding events are recorded and reported by the IWRS.

If an investigator, site personnel performing assessments, or participant is unblinded, the participant must be discontinued from the study. In cases where there are ethical reasons to have the participant remain in the study the investigator must obtain specific approval from a Lilly clinical research physician (CRP) for the participant to continue in the study.

In case of an emergency, the investigator has the sole responsibility for determining if unblinding of a participant's treatment assignment is warranted for medical management of the event. The participant's safety must always be the first consideration in making such a determination. If a participant's treatment assignment is unblinded, Lilly must be notified immediately. If the investigator decides that unblinding is warranted, it is the responsibility of the investigator to promptly document the decision and rationale and notify Lilly as soon as possible.

6.4. Study Intervention Compliance

Participant compliance with study drug intervention will be assessed at each visit (after randomization). Study drug compliance will be determined by the following:

- Study drug administration data will be recorded by the participant and reviewed by the investigator at each study visit.
- The participants will be instructed to return any unused study drug and/or empty cartons at the next office visit to the study site for the purpose of performing drug accountability.

Treatment compliance for each visit interval is defined as taking at least 75% of the required doses of study drug. Similarly, a participant will be considered significantly noncompliant if he or she is judged by the investigator to have intentionally or repeatedly taken more than the prescribed amount of medication (more than 125%).

In addition to the assessment of a participant's compliance with the study drug administration, other aspects of compliance with the study treatments will be assessed at each visit based on the participant's adherence to the visit schedule, completion of study diaries, and any other parameters the investigator considers necessary.

Participants considered to be poorly compliant with their study drug and/or the study procedures will receive additional training and instruction, as required, and will be reminded of the importance of complying with the protocol.

6.5. Concomitant Therapy

Participants will be permitted to use concomitant medications that they require during the study, except certain medications (for example, other medications for weight management, see Section 5.2) that may interfere with the assessment of efficacy and safety characteristics of the study treatments.

Participants who develop diabetes during the study may initiate medication for glucose control, with the exception of dipeptidyl peptidase 4 (DPP-4) inhibitors or GLP-1R agonists. Initiation of metformin for the treatment of diabetes is permitted, but metformin should not be initiated during the study for the treatment of other metabolic conditions (for example, PCOS, diabetes prevention). Management of incident diabetes should be performed by participants' usual care providers.

Investigative site staff will inform participants that they must consult with the investigator or a designated site staff member upon being prescribed any new medications during the study. This may not be possible when initiated for treatment of medical emergencies, in which case, the participant will inform the investigator or a designated site staff member as soon as possible.

Non-study medications taken by participants who are screened but not enrolled will not be reported to Lilly unless an SAE or AE occurs that the investigator believes may have been caused by a study procedure.

6.6. Dose Modification

6.6.1. Tirzepatide

Tirzepatide is given QW by SC injection. There are no restrictions on the time of day each weekly dose of tirzepatide is given, but it is advisable to administer the SC injections on the same day of the week and similar time each week. If a dose of tirzepatide is missed, the participant should take it as soon as possible unless it is within 72 hours of the next dose, in which case, that dose should be skipped and the next dose should be taken at the appropriate time (see Section 6.1).

Study drug dose modification is not permitted, except for management of intolerable GI symptoms from Week 0 to Week 24 (see Section 6.6.2).

6.6.2. Management of Participants with Gastrointestinal Symptoms

Participants who experience intolerable GI symptoms (for example, nausea, vomiting, or diarrhea) at any time during the study, should first be counseled on dietary behaviors that may help mitigate nausea and vomiting (for example, eating smaller meals, splitting 3 daily meals into 4 or more smaller ones, and stopping eating when they feel full) (see Section 10.9). If symptoms persist, the participant should be prescribed, at the investigator's discretion, symptomatic medication (for example, antiemetic or antidiarrheal medication). A temporary interruption of study drug for 1 dose is permitted, provided the participant has taken the last 3 weekly doses. Study treatment should be resumed immediately, either alone or in combination with symptomatic medication, which can also be utilized to manage symptoms. Management of study drug after interruptions >1 dose is discussed in Section 7.1.1.

During the first 24 weeks of the treatment period (20-week dose-escalation plus 4 weeks), if intolerable GI symptoms (for example, nausea, vomiting, or diarrhea) persist despite the above measures, the investigator should contact Lilly to consider a dose de-escalation step with subsequent re-escalation (Section 4.1.1.2.3). During the remaining treatment period (Week 24 to Week 72), dose de-escalation with subsequent re-escalation, or dose de-escalation to a lower maintenance dose, is not permitted. Those with intolerable GI symptoms that develop after 24 weeks and persist despite the above measures (for example, dietary counseling, symptomatic medication, and interruption of study drug for one dose) should discontinue study drug but remain in the study for continued follow-up.

6.7. Intervention after the End of the Study

Tirzepatide will not be made available to participants after conclusion of the study.

7. Discontinuation of Study Intervention and Participant Discontinuation/Withdrawal

7.1. Discontinuation of Study Intervention

In rare instances, it may be necessary for a participant to permanently discontinue (definitive discontinuation) study drug intervention. If intervention is definitively discontinued, the participant will remain in the study to be evaluated for any trial endpoint at the end of the study. See the SoA for data to be collected at the time of discontinuation of study drug intervention and follow-up and for any further evaluations that need to be completed.

In some instances, it may be necessary for a participant to permanently discontinue the intensive lifestyle intervention during the 12-week lead-in phase. If the intensive lifestyle intervention is discontinued during the lead-in period, the participant will be permanently discontinued from the study.

Possible reasons leading to permanent discontinuation of intensive lifestyle intervention during lead-in period or study drug intervention during treatment period:

- **Participant Decision**
  - The participant requests to discontinue intensive lifestyle intervention during the lead-in period or study drug intervention during treatment period.

- **Clinical Considerations**
  - Initiation of open-label GLP-1R agonist or DPP-4 inhibitor, if participants will not or cannot discontinue them
  - BMI ≤18.5 kg/m^2 is reached at any time

    Note: The investigator should contact the sponsor CRP to discuss whether it is medically appropriate for the participant to continue study treatment
  - Diagnosis of T1DM
  - Diagnosis of MTC or MEN syndrome type 2
  - Significant elevation of serum calcitonin (Section 8.3.2.4)
  - Diagnosis of acute or chronic pancreatitis
  - Diagnosis of an active or untreated malignancy (other than basal or squamous cell skin cancer, in situ carcinomas of the cervix, or in situ prostate cancer)
  - Onset of pregnancy in a female participant
  - Occurrence of any other treatment-emergent AE (TEAE), SAE, or clinically significant finding for which the investigator believes that permanent discontinuation is the appropriate measure to be taken
  - Inadvertent enrollment if continued intensive lifestyle intervention or treatment with study drug would not be medically appropriate or not allowed per local regulations
  - PHQ-9 score ≥15.

    - Participants should be referred to a Mental Health Professional (MHP) to assist in deciding whether the participant should be discontinued. If a participant's psychiatric disorder can be adequately treated with psycho- and/or pharmacotherapy, then the participant, at the discretion of the investigator (in agreement with the MHP), may be continued in the trial.
  - Participants with C-SSRS responses **at any visit** indicative of suicidal ideation or behavior should have intensive lifestyle or study drug interventions discontinued and be referred to an MHP. Both interventions may be restarted, if the MHP and the investigator agree it is appropriate for the participant. Responses that should prompt mental health referral and cessation of intensive lifestyle intervention or study drug intervention are
    - a "yes" answer to Question 4 (Active Suicidal Ideation with Some Intent to Act, Without Specific Plan) on the "Suicidal Ideation" portion of the C-SSRS

      or

    - a "yes" answer to Question 5 (Active Suicidal Ideation with Specific Plan and Intent) on the "Suicidal Ideation" portion of the C-SSRS

      or

    - a "yes" answer to any of the suicide-related behaviors (actual attempt, interrupted attempt, aborted attempt, preparatory act or behavior) on the "Suicidal Behavior" portion of the C-SSRS

  Note: A psychiatrist or appropriately trained professional may assist in the decision to discontinue the participant.

- **Discontinuation due to a hepatic event or liver test abnormality.**
  - Participants who are discontinued from intensive lifestyle intervention or study drug intervention due to a hepatic event or liver test abnormality should have additional hepatic safety data collected via eCRF.
  - Discontinuation of intensive lifestyle intervention or study drug intervention for abnormal liver tests **should be** considered by the investigator when a participant meets one of the following conditions after consultation with the Lilly designated medical monitor:
    - ALT or aspartate aminotransferase (AST) >8X ULN
    - ALT >2X baseline value or ≥300 U/L, whichever occurs first, if baseline ALT ≥2X ULN
    - ALT or AST >5X ULN for more than 2 weeks
    - ALT or AST >3X ULN and TBL >2X ULN or international normalized ratio (INR) >1.5
    - ALT or AST >3X ULN with the appearance of fatigue, nausea, vomiting, right upper-quadrant pain or tenderness, fever, rash, and/or eosinophilia (>5%)

- ALP >3X ULN
- ALP >2.5X ULN and TBL >2X ULN
- ALP >2.5X ULN with the appearance of fatigue, nausea, vomiting, right upper-quadrant pain or tenderness, fever, rash, and/or eosinophilia (>5%)

Note: In this study baseline may refer to lead-in enrollment visits or randomization visit, whichever is the most recent point of reference.

The following are also applicable if developing/occurring during intensive lifestyle lead-in period:

- Ketoacidosis or hyperosmolar state/coma
- Uncontrolled hypertension (systolic blood pressure above or equal to 160 mmHg and/or diastolic blood pressure above or equal to 100 mmHg)
- Diagnosis of any of the following cardiovascular conditions:
  - Acute myocardial infarction
  - Cerebrovascular accident (stroke)
  - Unstable angina
  - Hospitalization due to CHF, or
  - NYHA Functional Classification Class IV CHF
- Chronic (>2 weeks or >14 days) systemic glucocorticoid therapy (excluding topical, intra-ocular, intranasal, intra-articular, or inhaled preparations) or evidence of a significant, active autoimmune abnormality (for example, lupus or rheumatoid arthritis) that has required or is likely to require, in the opinion of the investigator, concurrent treatment with systemic glucocorticoids (excluding the ones mentioned above)
- Surgical treatment or endoscopic and/or device-based therapy for obesity
- Initiation of treatment with medications that may significantly impact body weight (please refer to Section 5.2. for details)
- Initiation of implantable or injectable contraceptives (such as Depo Provera®)

The following are also applicable during study drug intervention period (after randomization):

- Intolerable GI symptoms despite management as described in Section 6.6.2
- If the investigator, after consultation with the sponsor-designated medical monitor, determines that a systemic hypersensitivity reaction has occurred related to study drug administration, the participant should be permanently discontinued from the investigational drug.

Participants who stop intensive lifestyle intervention permanently should perform an early discontinuation (ED) visit and discontinue from the study. Participants who stop the study drug permanently should perform an ED visit and continue to attend all scheduled study visits to collect all planned efficacy and safety measurements. Randomized participants who are unwilling to attend all scheduled visits and stop the study treatment prior to 72 weeks, should

return for a final weight measurement (Visit 99). If participants are unwilling to attend Visit 99, their refusal to attend should be documented in the patient medical record.

See the SoA for data to be collected at the time of intervention discontinuation and follow-up and for any further evaluations that need to be completed.

7.1.1. Temporary Discontinuation

In certain situations, after randomization, the investigator may need to temporarily interrupt study drug. Every effort should be made by the investigator to maintain participants on study drug and to restart study drug after any temporary interruption, as soon as it is safe to do so. Distribution of study drug at the correct dose will be per IWRS instructions.

If study drug interruption is…	then…
2 consecutive doses or less	participant restarts study drug at last administered dose, as per escalation schedule.
3 consecutive doses or more	participant restarts study drug at 5 mg (managed by IWRS) and repeats dose escalation scheme.
due to an AE	the event is to be documented and followed according to the procedures in Section 8.3 of this protocol.
due to intolerable persistent GI AE	participants should be treated as suggested in Section 6.6.2.

Investigators should inform the sponsor that study drug has been temporarily interrupted. The data related to temporary interruption of study treatment will be documented in source documents and entered on the eCRF.

7.2. Participant Discontinuation/Withdrawal from the Study

To minimize the amount of missing data and to enable assessment of study objectives as planned in the study protocol, every attempt will be made to keep participants in the study irrespective of the following:

- adherence to or discontinuation from study drug
- adherence to visit schedule
- missing assessments
- study drug discontinuation due to AE
- development of comorbidities
- development of clinical outcomes

The circumstances listed above are ***not*** valid reasons for discontinuation from the study.

A participant may withdraw from the study:

- at any time at his/her own request
- at the request of his/her designee (for example, parents or legal guardian)
- at the discretion of the investigator for safety, behavioral, compliance, or administrative reasons
- if enrolled in any other clinical study involving an IP or enrolled in any other type of medical research judged not to be scientifically or medically compatible with this study
- if the participant, for any reason, requires treatment (for example, pharmacological, device-based or surgical) that has been demonstrated to be effective for treatment of the study indication, discontinuation from the study occurs prior to introduction of the new treatment

Female participants will be withdrawn from the study if the participant becomes pregnant.

Participation in the study can be stopped for medical, safety, regulatory, or other reasons consistent with applicable laws, regulations, and Good Clinical Practice (GCP). Participants who agree to provide information relevant to any trial endpoint at the end of the study are not considered to have discontinued from the study.

At the time of discontinuing from the study, if possible, an ED visit should be conducted, as shown in the SoA. See the SoA for data to be collected at the time of study discontinuation and follow-up and for any further evaluations that need to be completed. The participant will be permanently discontinued from either the intensive lifestyle intervention or study drug intervention, and from the study at that time.

If the participant withdraws consent for disclosure of future information, the sponsor may retain and continue to use any data collected before such a withdrawal of consent. If a participant withdraws from the study, he/she may request destruction of any samples taken and not tested, and the investigator must document this in the site study records.

7.2.1. Participant Disposition and Timing of Safety Follow-Up

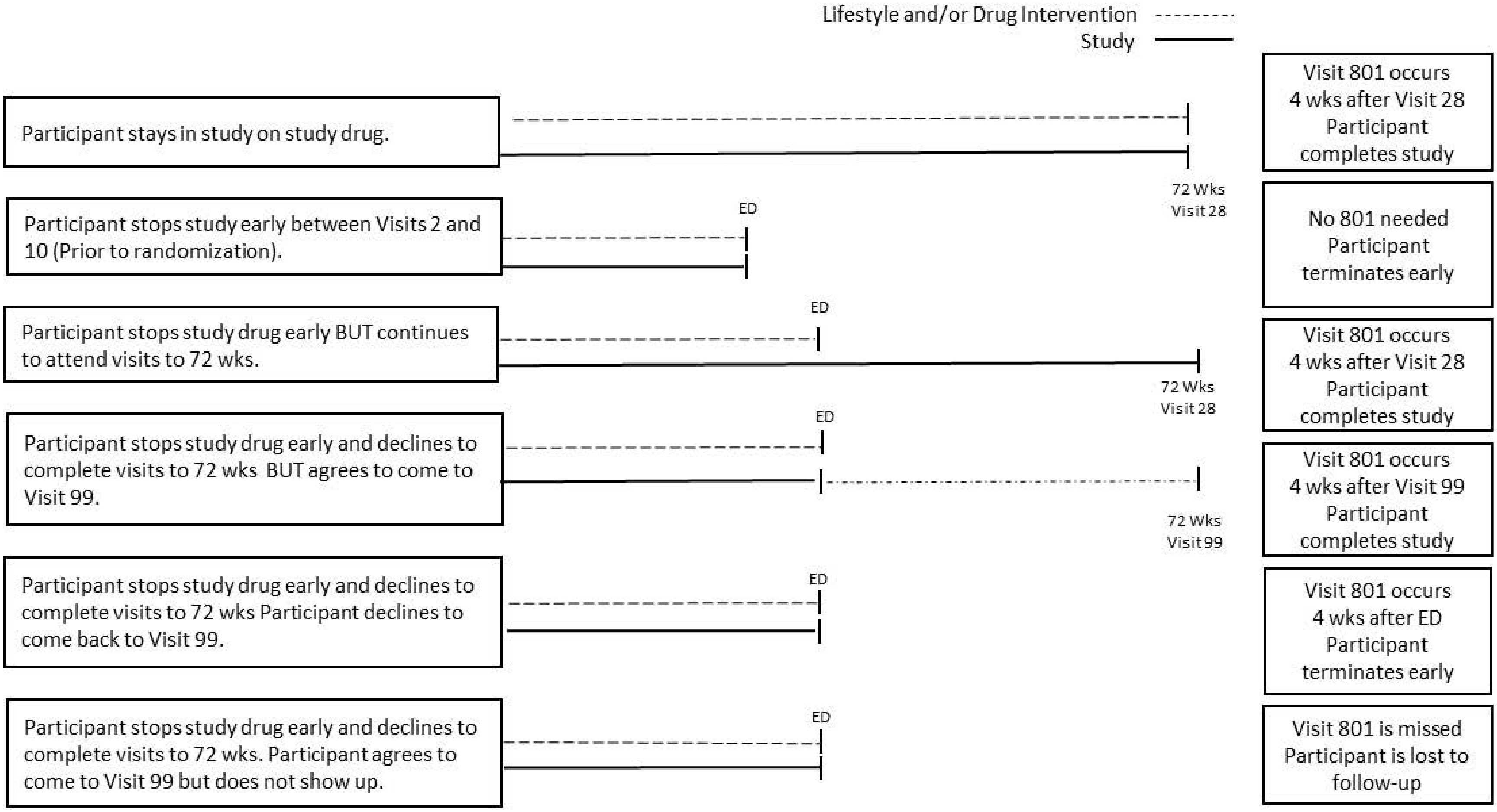

Abbreviations: ED = early discontinuation; wk(s) = week(s).

7.2.2. Discontinuation of Inadvertently Enrolled Participants

If the sponsor or investigator identifies a participant who did not meet enrollment criteria and was inadvertently enrolled, the investigator and the sponsor CRP must agree whether continuing the study drug intervention is medically appropriate. Participants identified as being inadvertently enrolled during lead-in period should be discontinued. Continuation of inadvertently enrolled participants in the study, with or without study drug treatment, requires documented approval from the sponsor CRP. Safety follow up should be performed as outlined in Section 1.3 (SoA), Section 8.3 (Adverse Events, Serious Adverse Events, and Product Complaints), and Section 8.2 (Safety Assessments) of the protocol.

7.3. Lost to Follow up

A participant will be considered lost to follow-up if he or she repeatedly fails to return for scheduled visits and is unable to be contacted by the study site. Site personnel or designee are expected to make diligent attempts to contact participants who fail to return for a scheduled visit or were otherwise unable to be followed-up by the site.

Site personnel, or an independent third party, will attempt to collect the vital status of the participant within legal and ethical boundaries for all participants enrolled, including those who did not get IP. Public sources may be searched for vital status information. If vital status is determined to be deceased, this will be documented, and the participant will not be considered lost to follow-up.

Lilly personnel will not be involved in any attempts to collect vital status information.

Discontinuation of specific sites or of the study as a whole are handled as part of Section 10.1 (Appendix 1).

8. Study Assessments and Procedures

- Study procedures and their timing are summarized in the SoA.
- Immediate safety concerns should be discussed with the sponsor immediately upon occurrence or awareness to determine if the participant should continue or discontinue intensive lifestyle intervention or study drug intervention
- Adherence to the study design requirements, including those specified in the SoA, is essential and required for study conduct.
- All screening evaluations must be completed and reviewed to confirm that potential participants meet all eligibility criteria. The investigator will maintain a screening log to record details of all participants screened and to confirm eligibility or record reasons for screening failure, as applicable

8.1. Efficacy Assessments

8.1.1. Primary Efficacy Assessments

The primary efficacy measurement in this study is body weight. Body weight measurements will be collected at specific clinic visits as summarized in the SoA. Methods for measuring body weight are described in Section 10.7.

8.1.2. Secondary Efficacy Assessments

The following secondary efficacy measures will be collected at the times shown in the SoA (Section 1.3)

- BMI (derived using body weight in kilograms divided by the square of height in meters)
- Waist circumference (measuring method is described in Section 10.7)
- Fasting insulin (measured through central lab)
- HbA1c (measured through central lab)
- Fasting glucose (measured through central lab)
- Blood pressure (measuring method is described in Section 10.7)
- Lipids (measured through central lab)

8.1.3. Patient-Reported Outcomes Assessments

The self-administered questionnaires will be translated into the native language of the region, linguistically validated and administered according to the SoA (Section 1.3). At these visits, the questionnaires should be completed before the participant has discussed their medical condition or progress in the study with the investigator and/or site staff, if the participant is not adversely affected by their fasting condition.

8.1.3.1. Short-Form 36 Version 2 Health Survey, Acute Form, 1-Week Recall Version

The SF-36v2 acute form, 1-week recall version is a 36-item generic, participant-administered measure designed to assess the following 8 domains

- Physical functioning
- Role-physical
- Bodily pain
- General health
- Vitality
- Social functioning
- Role-emotional
- Mental health

The Physical Functioning domain assesses limitations due to health "now" while the remaining domains assess functioning "in the past week." Each domain is scored individually and information from these 8 domains are further aggregated into 2 health-component summary scores: Physical Component Summary and Mental Component Summary. Items are answered on Likert scales of varying lengths (3-, 5- or 6- point scales). Scoring of each domain and both summary scores are norm-based and presented in the form of T-scores, with a mean of 50 and standard deviation (SD) of 10; higher scores indicate better levels of function and/or better health (Maruish 2011).

8.1.3.2. Impact of Weight on Quality of Life-Lite Clinical Trials Version

The IWQOL-Lite-CT is a 20-item, obesity-specific PRO instrument developed for use in obesity clinical trials. It assesses 2 primary domains of obesity-related health-related quality of life (HRQoL): Physical (7 items), and Psychosocial (13 items). A 5-item subset of the Physical domain – the Physical Function composite is also supported. Items in the Physical Function composite describe physical impacts related to general and specific physical activities. All items are rated on either a 5-point frequency ("never" to "always") scale or a 5-point truth ("not at all true" to "completely true") scale (Kolotkin et al. 2017, 2019).

8.1.3.3. EQ-5D-5L

Generic HRQoL will be assessed using the EQ-5D-5L (EuroQoL Research Foundation 2019). The EQ-5D-5L is a standardized 5-item instrument for use as a measure of health outcome. It provides a simple descriptive profile and a single index value for health status that can be used in the clinical and economic evaluation of health care as well as population health surveys. The EQ-5D-5L comprises 5 dimensions of health (mobility, self-care, usual activities, pain/discomfort, and anxiety/depression). The 5L version, introduced in 2005, scores each dimension at 5 levels (no problems, slight problems, moderate problems, severe problems, unable to perform/extreme problems), for a total of 3125 possible health states. In addition to the health profile, a single health state index value can be derived based on a formula that attaches weights to each of the levels in each dimension. This index value ranges between less than 0 (where 0 is a health state equivalent to death; negative values are valued as worse than dead) to 1 (perfect health) (Dolan 1997). In addition, the EQ Visual Analog Scale records the respondent's self-rated health status

on a vertical graduated (0 to 100) visual analog scale. In conjunction with the health state data, it provides a composite picture of the respondent's health status.

The EQ-5D-5L is used worldwide and is available in more than 170 different languages. Details on the instrument, scoring, organizing, and presenting the data collected can be found in the EQ-5D-5L User Guide (EuroQoL Research Foundation 2019).

8.1.3.4. Patient Global Impression of Status for Physical Activity

Study participants will be asked to complete a PGIs item specifically developed for this study. This is a participant-rated assessment of current limitation on physical activity due to health and is rated on a 5-point scale ranging from "1- not at all limited" to "5- extremely limited".

8.2. Safety Assessments

Planned time points for all safety assessments are provided in the SoA.

8.2.1. Physical Examinations

- A complete physical examination will include, at a minimum, assessments of the cardiovascular, respiratory, GI and neurological systems, as well as thyroid exam. Height, weight, and waist circumference will also be measured and recorded, per Section 10.7.
- Investigators should pay special attention to clinical signs related to previous serious illnesses.

8.2.2. Vital Signs

- For each participant, vital signs measurements should be conducted according to the SoA (Section 1.3) and following the study-specific recommendations included in Section 10.7 (Appendix 7).
- Any clinically significant findings from vital signs measurement that result in a diagnosis should be reported to Lilly or its designee as an AE via the eCRF.

8.2.3. Electrocardiograms

For each participant, single-tracing 12-lead electrocardiograms (ECGs) should be collected according to the SoA.

Electrocardiograms will initially be interpreted by a qualified physician (the investigator or qualified designee) at the site as soon after the time of ECG collection as possible, and ideally while the participant is still present, for immediate participant management, should any clinically relevant findings be identified. Any clinically significant findings from ECGs that result in a diagnosis should be reported to Lilly or its designee as an AE via the eCRF.

All digital ECGs will be obtained using centrally provided ECG machines and will be electronically transmitted to a designated central ECG laboratory. The central ECG laboratory will perform a basic quality control check (for example, demographics and study details) and then store the ECGs in a database. At a future time, the stored ECG data may be overread by a cardiologist at the central ECG laboratory for further evaluation of machine-read measurements or to meet regulatory requirements. The machine-read ECG intervals and heart rate may be used for

data analysis and report-writing purposes, unless a cardiology overreading of the ECGs is conducted prior to completion of the final study report (in which case, the overread data would be used).

8.2.4. Clinical Safety Laboratory Assessments

- See Section 10.2, Appendix 2: “Clinical Laboratory Tests” for the list of clinical laboratory tests to be performed and to the SoA for the timing and frequency.

  Note: see Section 10.2 Appendix 2: “Clinical Laboratory Tests” for clinical laboratory tests reported or not reported to the investigator

- The investigator must review the laboratory results, document this review, and report any clinically relevant changes occurring during the study as an AE. The laboratory results must be retained with source documents unless a Source Document Agreement or comparable document cites an electronic location that accommodates the expected retention duration. Clinically significant abnormal laboratory findings are those which are not associated with the underlying disease, unless judged by the investigator to be more severe than expected for the participant's condition.

- All laboratory tests with values considered clinically significantly abnormal during participation in the study or within 4 weeks after the last dose of study intervention should be repeated until the values return to normal or most recent baseline or are no longer considered clinically significant by the investigator or medical monitor.

  - If such values do not return to normal/most recent baseline within a period of time judged reasonable by the investigator, the etiology should be identified and the sponsor notified.

  - All protocol-required laboratory assessments, as defined in Section 10.2, must be conducted in accordance with the SoA, standard collection requirements, and laboratory manual.

- If laboratory values from non-protocol specified laboratory assessments performed at an investigator-designated local laboratory require a change in participant management or are considered clinically significant by the investigator (for example, SAE, AE, or dose modification), then report the information as an AE.

Repeat or unscheduled samples may be taken for safety reasons or for technical issues with the samples. Unless otherwise stated in the subsections below, all samples collected for specified laboratory tests will be destroyed within 60 days of receipt of confirmed test results. Certain samples may be retained for a longer period, if necessary, to comply with applicable laws, regulations, or laboratory certification standards.

8.2.5. Safety Monitoring

Lilly will periodically review evolving aggregate safety data within the study by appropriate methods. The study team will review safety reports in a blinded fashion according to the schedule provided in the Trial-Level Safety Review plan. Lilly will also review SAEs within time frames mandated by company procedures. The Lilly CRP will, as appropriate, consult with the functionally independent Global Patient Safety therapeutic area physician or clinical scientist.

8.2.5.1. Hepatic Safety Monitoring

Close Hepatic Monitoring

Laboratory tests (Section 10.6, Appendix 6), including ALT, AST, ALP, TBL, direct bilirubin, gamma-glutamyl transferase, and creatine kinase, should be repeated within 48 to 72 hours to confirm the abnormality and to determine if it is increasing or decreasing, if 1 or more of these conditions occur:

If a participant with baseline results of…	develops the following elevations:
ALT or AST <1.5X ULN	ALT or AST ≥3X ULN
ALP <1.5X ULN	ALP ≥2X ULN
TBL <1.5X ULN	TBL ≥2X ULN (except for patients with Gilbert's syndrome)
ALT or AST ≥1.5X ULN	ALT or AST ≥2X baseline
ALP ≥1.5X ULN	ALP ≥2X baseline
TBL ≥1.5X ULN	TBL ≥1.5X baseline (except for patients with Gilbert's syndrome)

Abbreviations: ALP = alkaline phosphatase; ALT = alanine aminotransferase; AST = aspartate aminotransferase; TBL = total bilirubin level; ULN = upper limit of normal.

Note: In this study baseline may refer to lead-in enrollment visits or randomization visit, whichever is the most recent point of reference.

If the abnormality persists or worsens, clinical and laboratory monitoring, and evaluation for possible causes of abnormal liver tests should be initiated by the investigator in consultation with the Lilly-designated medical monitor. At a minimum, this evaluation should include physical examination and a thorough medical history, including symptoms, recent illnesses (for example, heart failure, systemic infection, hypotension, or seizures), recent travel, history of concomitant medications (including over-the-counter), herbal and dietary supplements, and history of alcohol drinking and other substance abuse.

Initially, monitoring of symptoms and hepatic biochemical tests should be done at a frequency of 1 to 3 times weekly, based on the participant's clinical condition and hepatic biochemical tests.

Subsequently, the frequency of monitoring may be lowered to once every 1 to 2 weeks, if the participant's clinical condition and lab results stabilize. Monitoring of ALT, AST, ALP, and TBL should continue until levels normalize or return to approximate baseline levels.

Comprehensive Hepatic Evaluation

A comprehensive evaluation should be performed to search for possible causes of liver injury if one or more of these conditions occur:

If a participant with baseline results of…	develops the following elevations:
ALT or AST <1.5X ULN	ALT or AST ≥3X ULN with hepatic signs/symptoms*, or ALT or AST ≥5X ULN
ALP <1.5X ULN	ALP ≥3X ULN
TBL <1.5X ULN	TBL ≥2X ULN (except for patients with Gilbert's syndrome)
ALT or AST ≥1.5X ULN	ALT or AST ≥2X baseline with hepatic signs/symptoms*, or ALT or AST ≥3X baseline
ALP ≥1.5X ULN	ALP ≥2X baseline
TBL ≥1.5X ULN	TBL ≥2X baseline (except for patients with Gilbert's syndrome)

Abbreviations: ALP = alkaline phosphatase; ALT = alanine aminotransferase; AST = aspartate aminotransferase; TBL = total bilirubin level; ULN = upper limit of normal.

* Hepatic signs/symptoms are severe fatigue, nausea, vomiting, jaundice, right upper quadrant abdominal pain, fever, rash, and/or eosinophilia >5%.

Note: In this study baseline may refer to lead-in enrollment visits or randomization visit, whichever is the most recent point of reference.

At a minimum, this evaluation should include physical examination and a thorough medical history, as outlined above, as well as tests for prothrombin-time-INR; tests for viral hepatitis A, B, C, or E; tests for autoimmune hepatitis; and an abdominal imaging study (for example, ultrasound or computed tomography (CT) scan).

Based on the participant's history and initial results, further testing should be considered in consultation with the Lilly-designated medical monitor, including tests for hepatitis D virus, cytomegalovirus, Epstein-Barr virus, acetaminophen levels, acetaminophen protein adducts, urine toxicology screen, Wilson's disease, blood alcohol levels, urinary ethyl glucuronide, and blood phosphatidylethanol. Based on the circumstances and the investigator's assessment of the participant's clinical condition, the investigator should consider referring the participant for a hepatologist or gastroenterologist consultation, magnetic resonance cholangiopancreatography, endoscopic retrograde cholangiopancreatography, cardiac echocardiogram, or a liver biopsy.

Additional Hepatic Data Collection (Hepatic Safety CRF) in Study Participants Who Have Abnormal Liver Tests During the Study

Additional hepatic safety data collection in hepatic safety case report forms (CRFs) should be performed in study participants who meet 1 or more of the following 5 conditions:

1. Elevation of serum ALT to ≥5X ULN on 2 or more consecutive blood tests (if baseline ALT <1.5X ULN)
    - In participants with baseline ALT ≥1.5X ULN, the threshold is ALT ≥3X baseline on 2 or more consecutive tests
2. Elevated TBL to ≥2X ULN (if baseline TBL <1.5X ULN) (except for cases of known Gilbert's syndrome)
    - In participants with baseline TBL ≥1.5X ULN, the threshold should be TBL ≥2X baseline
3. Elevation of serum ALP to ≥2X ULN on 2 or more consecutive blood tests (if baseline ALP <1.5X ULN)
    - In participants with baseline ALP ≥1.5X ULN, the threshold is ALP ≥2X baseline on 2 or more consecutive blood tests
4. Hepatic event considered to be an SAE
5. Discontinuation of intensive lifestyle intervention or study drug intervention due to a hepatic event

Note: the interval between the 2 consecutive blood tests should be at least 2 days.

Note: In this study baseline may refer to lead-in enrollment visits or randomization visit, whichever is the most recent point of reference.

8.2.6. Depression, Suicidal Ideation and Behavior Risk Monitoring

Patients who have obesity or are overweight are at increased risk for depression (Luppino et al. 2010). Depression can increase the risk for suicidal ideation and behavior. Therefore, study participants will be screened at trial entry and monitored during the study for depression, suicidal ideation and behavior.

Participants should be monitored appropriately and observed closely for suicidal ideation and behavior or any other unusual changes in behavior, especially at the beginning and end of the course of treatment, or at the time of dose changes, either increases or decreases. Consideration should be given to discontinuing intensive lifestyle intervention or the study drug intervention in participants who experience signs of suicidal ideation or behavior, following a risk assessment (Section 7.1).

Baseline and treatment emergent assessment of depression, suicidal ideation and behavior will be monitored during the study using the C-SSRS and PHQ-9 (Section 8.3.2.13).

8.3. Adverse Events, Serious Adverse Events, and Product Complaints

The definitions of the following events can be found in Section 10.4 (Appendix 4):

- Adverse events (AEs)

- Serious adverse events (SAEs)
- Product complaints (PCs)

These events will be reported by the participant (or, when appropriate, by a caregiver, surrogate, or the participant's legally authorized representative).

The investigator and any qualified designees are responsible for detecting, documenting, and recording events that meet these definitions and remain responsible for following up events that are serious, considered related to the study drug intervention, or study procedures (for example, intensive lifestyle intervention), or that caused the participant to discontinue the study intervention (see Section 7).

Care will be taken not to introduce bias when detecting events. Open-ended and non-leading verbal questioning of the participant is the preferred method to inquire about event occurrences.

After the initial report, the investigator is required to proactively follow each participant at subsequent visits/contacts. All SAEs, will be followed until resolution, stabilization, the event is otherwise explained, or the participant is lost to follow-up (as defined in Section 7.3). For product complaints, the investigator is responsible for ensuring that follow-up includes any supplemental investigations as indicated to elucidate the nature and/or causality. Further information on follow-up procedures is provided in Section 10.4 (Appendix 4).

8.3.1. Timing and Mechanism for Collecting Events

This table describes the timing, deadlines, and mechanism for collecting events.

**Event**	**Collection Start**	**Collection Stop**	**Timing for Reporting to Sponsor or Designee**	**Mechanism for Reporting**	**Back-up Method of Reporting**
**Adverse Event**					
AE	signing of the informed consent form (ICF)	the safety follow-up visit OR participation in study has ended	As soon as possible upon site awareness	AE eCRF	N/A
**Serious Adverse Event**					
SAE and SAE updates – prior to start of study drug intervention **and** deemed reasonably possibly related with study procedures	signing of the informed consent form (ICF)	start of study drug intervention	Within 24 hours of awareness	SAE eCRF	SAE paper form

Event	Collection Start	Collection Stop	Timing for Reporting to Sponsor or Designee	Mechanism for Reporting	Back-up Method of Reporting
SAE and SAE updates – after start of study drug intervention	start of study drug intervention	The safety follow-up visit OR participation in study has ended	Within 24 hours of awareness	SAE eCRF	SAE paper form
SAE – after participant's study participation has ended **and** the investigator becomes aware	After participant's study participation has ended	N/A	Promptly	SAE paper form	N/A
**Pregnancy**					
Pregnancy in female participants and female partners of male participants	After the start of study intervention	Four months after the last injection for female partners of male participants and 2 months after the last injection for female participants	Within 24 hours of learning of the pregnancy	eCRF	SAE paper form
**Product Complaints**					
PC associated with an SAE or might have led to an SAE	Start of study drug intervention	End of study drug intervention	Within 24 hours of awareness	Product Complaint form	N/A
PC not associated with an SAE	Start of study drug intervention	End of study drug intervention	Within 1 business day of awareness	Product Complaint form	N/A

Event	Collection Start	Collection Stop	Timing for Reporting to Sponsor or Designee	Mechanism for Reporting	Back-up Method of Reporting
Updated PC information	—	—	As soon as possible upon site awareness	Originally completed Product Complaint form with all changes signed and dated by the investigator	N/A
PC (after participant's study participation has ended **and** the investigator becomes aware)	After participant's study participation has ended	N/A	Promptly	Product Complaint form	N/A

Abbreviations: AE = adverse event; eCRF = electronic case report form; N/A = not applicable; PC = product complaint; SAE = serious adverse event.

8.3.2. Special Safety Topics

8.3.2.1. Hypoglycemia

Upon ICF signing, all participants will be educated about signs and symptoms of hypoglycemia, how to treat hypoglycemia, and how to collect appropriate information for each episode of hypoglycemia.

Hypoglycemia may be identified by spontaneous reporting of symptoms from participants (whether confirmed or unconfirmed by simultaneous glucose values) or by blood glucose (BG) samples collected during study visits.

All participants who develop diabetes during the study will be provided with glucometers. Participants without diabetes may be given glucometers to assist in the evaluation of reported symptoms consistent with hypoglycemia at the investigator's discretion. Participants receiving glucometers will be provided a diary to record relevant information (for example, glucose values, symptoms).

All hypoglycemic episodes will be recorded on a specific eCRF (Hypoglycemic Events eCRF) and should not be recorded on the AE eCRF, unless the event meets serious criteria. If a hypoglycemic event meets severe criteria (see definition below), it should be recorded as serious on the AE and SAE eCRFs, and reported to Lilly as an SAE. All hypoglycemic events collected on the hypoglycemic eCRF will be considered as treatment-emergent (TE) and included in the analysis of incidence and rate of hypoglycemic events.

Investigators should use the following definitions and criteria when diagnosing and categorizing an episode considered to be related to hypoglycemia (the BG values in this section refer to values determined by a laboratory or International Federation of Clinical Chemistry and Laboratory Medicine blood-equivalent glucose meters and strips) in accordance with the 2020 American Diabetes Association position statement on glycemic targets (ADA 2020):

Glucose Alert Value (Level 1):

- **Documented symptomatic hypoglycemia** is defined as any time a patient feels that he or she is experiencing symptoms and/or signs associated with hypoglycemia and has a BG level of <70 mg/dL (<3.9 mmol/L).
- **Documented asymptomatic hypoglycemia** is defined as any event not accompanied by typical symptoms of hypoglycemia, but with a measured BG <70 mg/dL (<3.9 mmol/L).
- **Documented unspecified hypoglycemia** is defined as any event with no information about symptoms of hypoglycemia available, but with a measured BG <70 mg/dL (<3.9 mmol/L).

Clinically Significant Hypoglycemia (Level 2):

- **Documented symptomatic hypoglycemia** is defined as any time a participant feels that he or she is experiencing symptoms and/or signs associated with hypoglycemia and has a BG level of <54 mg/dL (<3.0 mmol/L).
- **Documented asymptomatic hypoglycemia** is defined as any event not accompanied by typical symptoms of hypoglycemia but with a measured BG <54 mg/dL (<3.0 mmol/L).
- **Documented unspecified hypoglycemia** is defined as any event with no information about symptoms of hypoglycemia available but with a measured BG <54 mg/dL (<3.0 mmol/L).

Severe hypoglycemia (Level 3):

- **Severe hypoglycemia** is defined as an episode with severe cognitive impairment requiring the assistance of another person to actively administer carbohydrate, glucagon, or other resuscitative actions. These episodes may be associated with sufficient neuroglycopenia to induce seizure or coma. Blood glucose measurements may not be available during such an event, but neurological recovery attributable to the restoration of BG to normal is considered sufficient evidence that the event was induced by a low BG concentration.

To avoid duplicate reporting, all consecutive BG values <70 mg/dL (<3.9 mmol/L) occurring within a 1-hour period may be considered to be a single hypoglycemic event (Weinberg et al. 2010; Danne et al. 2013).

8.3.2.2. Pancreatitis

Diagnosis of Acute Pancreatitis

Acute pancreatitis is an AE of interest in all studies with tirzepatide, including this study. The diagnosis of acute pancreatitis requires 2 of the following 3 features (Banks et al. 2006; Koizumi 2006):

- abdominal pain, characteristic of acute pancreatitis (that is, epigastric pain radiating to the back, often associated with nausea and vomiting)
- serum amylase (total, pancreatic, or both) and/or lipase ≥3X ULN
- characteristic findings of acute pancreatitis on CT scan or magnetic resonance imaging (MRI)

If acute pancreatitis is suspected, the investigator should

- obtain appropriate laboratory tests, including pancreatic amylase (p-amylase) and lipase
- perform imaging studies, such as abdominal CT scan with or without contrast, abdominal MRI, or gallbladder ultrasound

Note: Abdominal ultrasound may be used as an alternative method only if CT and MRI cannot be performed.

- evaluate for possible causes of acute pancreatitis, including alcohol use, gallstone/gallbladder disease, hypertriglyceridemia, and concomitant medications.

Discontinuation for Acute Pancreatitis

If acute pancreatitis is diagnosed after randomization, the participant must discontinue use of the IP, but will continue in the study.

Case Adjudication and Data Entry

An independent clinical endpoint committee (CEC) will adjudicate all suspected cases of acute pancreatitis. In addition, AEs of severe or serious abdominal pain of unknown etiology will also be submitted to the adjudication committee to assess for possible pancreatitis or other pancreatic disease.

Asymptomatic Elevation of Pancreatic Amylase and/or Lipase

Serial measures of pancreatic enzymes have limited clinical value for predicting episodes of acute pancreatitis in asymptomatic patients (Nauck et al. 2017; Steinberg et al. 2017a, 2017b). Therefore, further diagnostic follow-up of cases of asymptomatic elevation of pancreatic enzymes (lipase and/or p-amylase ≥3X ULN) is not mandated but may be performed based on the investigator’s clinical judgment and assessment of the participant’s overall clinical condition.

8.3.2.3. Thyroid Malignancies and C-Cell Hyperplasia

Individuals with personal or family history of MTC and/or MEN syndrome type 2 will be excluded from the study. Participants who are diagnosed with MTC and/or MEN syndrome type 2 during the study will have intensive lifestyle intervention or study drug intervention stopped and should continue follow-up with an endocrinologist.

The assessment of thyroid safety during the trial will include reporting of any case of thyroid malignancy (including MTC, papillary carcinoma and others) and measurements of calcitonin. These data will be captured in specific eCRFs. The purpose of calcitonin measurements is to assess the potential of tirzepatide to affect thyroid C-cell function, which may indicate development of C-cell hyperplasia and neoplasms.

8.3.2.4. Calcitonin Measurements

If an increased calcitonin value (see definitions below) is observed in a participant who has been administered a medication that is known to increase serum calcitonin, then this medication should be stopped, and calcitonin levels should be measured after an appropriate washout period.

For participants who require additional endocrine assessment because of increased calcitonin concentration as defined in this section, data from the follow-up assessment will be collected in the specific section of the eCRF.

Calcitonin Measurements in Participants with eGFR ≥60 mL/min/1.73 m^2

A significant increase in calcitonin for participants with eGFR ≥60 mL/min/1.73 m^2 is defined below. If a participant's laboratory results meet these criteria, these clinically significant laboratory results should be recorded as an AE.

- *Serum calcitonin value ≥20 ng/L and <35 ng/L AND ≥50% increase from the baseline value.* These participants will be asked to repeat the measurement within 1 month. If this repeat value is increasing (≥10% increase), the study drug should be stopped, and the participant encouraged to undergo additional endocrine assessment and longer term, follow-up by an endocrinologist to exclude any serious adverse effect on the thyroid.
- *Serum calcitonin value ≥35 ng/L AND ≥50% over the baseline value*. In these participants, study drug should be stopped, and the participant recommended to immediately undergo additional endocrine assessments and longer term, follow-up by an endocrinologist.

Calcitonin Measurement in Participants with eGFR <60 mL/min/1.73 m^2

A significant increase in calcitonin for participants with eGFR <60 mL/min/1.73 m^2 is defined as a *serum calcitonin value ≥35 ng/L AND ≥50% over the baseline value.* If a participant's labs meet these criteria, these clinically significant labs should be recorded as an AE.

In these participants, study drug should be discontinued (after first confirming the value) and the participant recommended to immediately undergo additional endocrine assessments and longer term, follow-up by an endocrinologist to exclude any serious adverse effect on the thyroid.

Note: In this study baseline may refer to lead-in enrollment visits or randomization visit, whichever is the most recent point of reference.

8.3.2.5. Major Adverse Cardiovascular Events

Deaths and nonfatal cardiovascular AEs will be adjudicated by a committee of physicians external to Lilly with cardiology expertise. This committee will be blinded to treatment assignment. The nonfatal cardiovascular AEs to be adjudicated include

- myocardial infarction
- hospitalization for unstable angina
- hospitalization for heart failure
- coronary interventions (such as coronary artery bypass graft or percutaneous coronary intervention), and
- cerebrovascular events, including cerebrovascular accident (stroke) and transient ischemic attack.

8.3.2.6. Supraventricular Arrhythmias and Cardiac Conduction Disorders

Treatment-emergent cardiac conduction disorders will be further evaluated. Participants who develop any event from these groups of disorders should undergo an ECG, which should be submitted to the central reading center. Additional diagnostic tests to determine exact diagnosis should be performed, as needed. The specific diagnosis will be recorded as an AE. Events that meet criteria for serious conditions as described in Section 10.4 must be reported as SAEs.

8.3.2.7. Hypersensitivity Reactions

Many drugs, but particularly biologic agents, carry the risk of systemic hypersensitivity reactions. If such a reaction occurs, additional data describing each symptom should be provided to the sponsor in the eCRF.

Sites should have appropriately trained medical staff and appropriate medical equipment available when study participants are receiving study drug. It is recommended that participants who experience a systemic hypersensitivity reaction be treated per national and international guidelines.

In the case of generalized urticaria or anaphylaxis, additional blood and urine samples should be collected as described in Section 10.3, Appendix 3, “Laboratory Testing for Hypersensitivity Events”. Laboratory results are provided to the sponsor via the central laboratory.

8.3.2.8. Injection-Site Reactions

Symptoms of a local injection-site reaction may include erythema, induration, pain, pruritus, and edema. If an injection site event is reported, the AE will be recorded, and additional data will be provided to the sponsor in the eCRF.

Injection site reactions will be collected on the eCRF separate from the hypersensitivity reaction eCRF. At the time of AE occurrence, samples will be collected for measurement of tirzepatide antidrug antibodies (ADA) and tirzepatide concentration.

8.3.2.9. Antidrug Antibodies

The occurrence of ADA formation will be assessed as outlined in Section 8.9.

8.3.2.10. Hepatobiliary Disorders

All events of TE biliary colic, cholecystitis, or other suspected events related to gallbladder disease should be evaluated and additional diagnostic tests performed, as needed. In cases of elevated liver markers, hepatic monitoring should be initiated as outlined in Section 8.2.5.1.

8.3.2.11. Severe Gastrointestinal Adverse Events

Tirzepatide may cause severe GI AEs, such as nausea, vomiting, and diarrhea. Information about severe GI AEs as well as antiemetic/antidiarrheal use will be collected in the eCRF/AE form. For detailed information concerning the management of GI AEs, please refer to Section 6.6.2.

8.3.2.12. Acute Renal Events

Renal safety will be assessed based on repeated renal function assessment as well as assessment of AEs suggestive of acute or worsening of chronic renal failure. Gastrointestinal AEs have been reported with tirzepatide, including nausea, diarrhea, and vomiting. This is consistent with other

GLP-1R agonists (Aroda and Ratner 2011). The events may lead to dehydration, which could cause a deterioration in renal function, including acute renal failure. Participants should be advised to notify investigators in case of severe nausea, frequent vomiting, or symptoms of dehydration.

8.3.2.13. Depression, Suicidal Ideation or Behavior Monitoring

Participants will be monitored for depression and suicidal ideation or behavior through AE collection and by using the C-SSRS and the PHQ-9. Participants will be referred to an MHP if in the opinion of the investigator it is necessary for the safety of the participant or if the participant had any of the following:

- a PHQ-9 score ≥15
- C-SSRS responses of
  - A “yes” answer to Question 4 (Active Suicidal Ideation with Some Intent to Act, Without Specific Plan) on the “Suicidal Ideation” portion of the C-SSRS
    or
  - A “yes” answer to Question 5 (Active Suicidal Ideation with Specific Plan and Intent) on the “Suicidal Ideation” portion of the C-SSRS
    or
  - A “yes” answer to any of the suicide-related behaviors (actual attempt, interrupted attempt, aborted attempt, preparatory act, or behavior) on the “Suicidal Behavior” portion of the C-SSRS

8.4. Treatment of Overdose

Study drug overdose (more than the specified number of injections) will be reported as an AE. In the event of overdose, refer to the IB for tirzepatide.

8.5. Pharmacokinetics

Not applicable.

8.6. Pharmacodynamics

Samples to assess the PD properties of tirzepatide are included in the efficacy measures and not applicable in this section.

8.7. Genetics

A whole blood sample will be collected for pharmacogenetic analysis as specified in the SoA (Section 1.3) where local regulations allow.

Samples will not be used to conduct unspecified disease or population genetic research either now or in the future. Samples will be used to investigate variable response to tirzepatide and to investigate genetic variants thought to play a role in obesity. Assessment of variable response may include evaluation of AEs or differences in efficacy.

All samples will be coded with the participant number. These samples and any data generated can be linked back to the participant only by the investigator site personnel.

Samples will be retained at a facility selected by Lilly or its designee for a maximum of 15 years after the last participant visit for the study, or for a shorter period if local regulations and/or Ethical Review Boards (ERBs)/ Institutional Review Boards (IRBs) impose shorter time limits. This retention period enables use of new technologies, response to regulatory questions, and investigation of variable response that may not be observed until later in the development of tirzepatide or after tirzepatide become(s) commercially available.

Molecular technologies are expected to improve during the 15-year storage period and therefore cannot be specifically named. However, existing approaches include whole genome or exome sequencing, genome wide association studies, and candidate gene studies. Regardless of technology utilized, genotyping data generated will be used only for the specific research scope described in this section.

8.8. Biomarkers

Biomarker research is performed to address questions of relevance to drug disposition, target engagement, PD, mechanism of action, variability of participant response (including safety), and clinical outcome. Sample collection is incorporated into clinical studies to enable examination of these questions through measurement of biomolecules including deoxyribonucleic acid (DNA), proteins, lipids, and other cellular elements.

Serum and plasma samples for biomarker research (non-pharmacogenetic stored sample) will be collected at the times specified in the SoA (Section 1.3) where local regulations allow.

Samples will be used for research on the drug target, disease process, variable response to tirzepatide, pathways associated with obesity, mechanism of action of tirzepatide, and/or research method or in validating diagnostic tools or assay(s) related to obesity.

All samples will be coded with the participant number. These samples and any data generated can be linked back to the participant only by the investigator site personnel.

Samples will be retained at a facility selected by Lilly or its designee for a maximum 15 years after the last participant visit for the study, or for a shorter period if local regulations and ERBs impose shorter time limits. This retention period enables use of new technologies, response to regulatory questions, and investigation of variable response that may not be observed until later in the development of tirzepatide or after tirzepatide become(s) commercially available.

8.9. Immunogenicity Assessments

Where local regulations and ERBs allow, blood samples for immunogenicity testing will be collected to determine antibody production against tirzepatide as specified in the SoA (Section 1.3).

For immunogenicity testing, venous blood samples will be collected from each participant according to the SoA (Section 1.3) to determine antibody production against tirzepatide. To interpret the results of immunogenicity, a PK sample will be collected at the same time points as the immunogenicity sample. All samples for immunogenicity should be taken predose when applicable and possible.

In the event of systemic drug hypersensitivity reactions (immediate or nonimmediate), additional unscheduled samples will be collected as detailed in Section 8.3.2.7 (Hypersensitivity Reactions).

Instructions for the collection and handling of blood samples will be provided by the sponsor. The actual date and time (24-hour clock time) of each sampling will be recorded. Samples collected at Visit 801 will assess immunogenicity at washout of tirzepatide (5 half-lives post end of treatment).

Immunogenicity will be assessed by a validated assay designed to detect ADAs in the presence of tirzepatide at a laboratory approved by the sponsor. Antibodies may be further characterized for their ability to neutralize the activity of tirzepatide on GIP and GLP-1 receptors. Positive tirzepatide ADA samples will be tested for cross-reactivity with native GIP and GLP-1, and, if positive, may then be tested for neutralizing antibodies against native GIP and/or GLP-1. In vivo laboratory indicators for effect on weight loss and PK will be utilized to detect potential neutralizing effect of ADA against tirzepatide.

Treatment-emergent ADAs are defined in Section 9.4.6.

Samples will be retained for a maximum of 15 years after the last participant visit, or for a shorter period if local regulations and ERBs allow, at a facility selected by the sponsor. The duration allows the sponsor to respond to future regulatory requests related to tirzepatide. Any samples remaining after 15 years will be destroyed.

Concentrations of tirzepatide will be assayed using a validated liquid chromatography mass spectrometry method. Bioanalytical samples collected to measure tirzepatide concentrations will be retained for a maximum of 1 year following last subject visit for the study. During this time, samples remaining after the bioanalyses may be used for exploratory analyses such as metabolism work, protein binding, and/or bioanalytical method cross-validation.

8.10. Health Economics

Health Economics parameters are not evaluated in this study.

9. Statistical Considerations

9.1. Statistical Hypotheses

The alternative hypothesis for the primary objective is the following:

H_1: QW tirzepatide MTD is superior to placebo for mean percent change in body weight AND proportion of participants who achieve ≥5% body weight reduction from randomization to 72 weeks.

The above hypothesis will be tested at 2-sided significance level of 0.05.

The alternative hypotheses for the key secondary objectives controlling for type 1 error rate are the following:

H_2: QW tirzepatide MTD is superior to placebo for percentage of participants who maintain ≥80% of the body weight lost during the 12-week lead-in period.

H_3: QW tirzepatide MTD is superior to placebo for proportion of participants who achieve ≥10% body weight reduction from randomization to 72 weeks.

H_4: QW tirzepatide MTD is superior to placebo for proportion of participants who achieve ≥15% body weight reduction from randomization to 72 weeks.

H_5: QW tirzepatide MTD is superior to placebo for mean change in waist circumference (cm) from randomization to 72 weeks.

9.2. Sample Size Determination

Approximately 1100 participants will be screened and 800 participants enrolled into the 12-week lead-in period in order to achieve approximately 600 participants randomly assigned to study drug intervention (300 participants per treatment group).

The sample size determination assumes that evaluation of superiority of tirzepatide MTD (10 mg or 15 mg) to placebo will be conducted at a 2-sided significance level of 0.05 using a 2-sample t-test. Additionally, a difference of at least 12% mean body weight percentage reduction from randomization at 72 weeks for tirzepatide MTD compared to placebo, a common SD of 10%, and a dropout rate of 25% are assumed for statistical power calculations. Under the assumptions above, randomizing 600 participants in a 1:1 ratio to MTD (300 participants) and placebo (300 participants) provides more than 90% power to demonstrate superiority of tirzepatide MTD to placebo.

The chosen sample size and randomization ratio also provides >90% power to establish superiority of tirzepatide MTD to placebo in terms of proportion of participants achieving at least 5% body weight reduction from randomization at 72 weeks, based on a Chi-square test at a 2-sided significance level of 0.05, assuming 20% placebo treated participants and 46% tirzepatide treated participants achieve the goal and a dropout rate of 25%.

9.3. Populations for Analyses

For purposes of analysis, the following populations are defined:

Population	Description
Entered	All participants who sign informed consent
Enrolled	All participants who are enrolled in 12-week lead-in period
Randomized	All participants who are randomly assigned a study drug.
Modified Intent-to-Treat (mITT)	All randomly assigned participants who are exposed to at least 1 dose of study drug. Participants will be included in the treatment group to which they were randomized.
Efficacy Analysis Set (EAS)	Data obtained during the double-blind treatment period from the mITT population, excluding data after discontinuation of study drug (last dose date + 7 days)
Full Analysis Set (FAS)	Data obtained during the treatment period from mITT population, regardless of adherence to study drug.
Safety Analysis Set (SS)	Data obtained during the treatment plus follow up period from the mITT population, regardless of adherence to study drug.

9.4. Statistical Analyses

9.4.1. General Considerations

Statistical analysis of this study will be the responsibility of Lilly or its designee. Any change to the data analysis methods described in the protocol will require an amendment ONLY if it changes a principal feature of the protocol. Any other change to the data analysis methods described in the protocol, and the justification for making the change, will be described in the statistical analysis plan (SAP) or the clinical study report (CSR). Additional exploratory data analyses may be conducted as deemed appropriate.

Unless otherwise noted, all tests of treatment effects will be conducted at a 2-sided alpha level of 0.05, and the confidence interval (CI) will be calculated at 95%, 2-sided. In statistical summaries and analyses, data will be analyzed as randomized.

Unless specified otherwise, efficacy analyses will be conducted using efficacy analysis set (EAS) and safety analyses will be conducted using safety analysis set (SS).

Unless otherwise specified, for analyses in the double-blind period, baseline is defined as the last non-missing data collected prior to or at randomization (prior to first dosing of study drug); for analyses in the lead-in period, baseline is defined as the last non-missing data collected prior to or at study entry (Visit 1 and Visit 2).

Summary statistics for continuous measures will include sample size, mean, SD, median, minimum, and maximum. The analysis model to make comparisons among treatment groups relative to continuous measurements assessed over time will be a mixed model for repeated measures (MMRM), with terms of treatment, visit, treatment-by-visit interaction, stratification factors and baseline measurement as a covariate. An unstructured covariance structure will model relationship of within-patient errors.

Kaplan-Meier method will be used for estimation of cumulative event-free survival rates over time, and cox proportional hazards regression analysis will be used to compare hazard rates among treatments.

Summary statistics for categorical measures (including categorized continuous measures) will include sample size, frequency, and percentages. Logistic regression will be used to examine the treatment difference in binary efficacy outcomes if there is a need to adjust for covariates. Otherwise, Fisher's exact test will be used to examine the treatment difference in categorical outcomes.

Summary statistics for discrete count measures will include sample size, mean, SD, median, minimum, and maximum. The negative binomial regression model will be used for the treatment comparison of discrete count measures.

Other statistical methods may be used, as appropriate, and details will be described in the SAP.

9.4.2. Treatment Group Comparability

9.4.2.1. Participant Disposition

A detailed description of participant disposition will be provided at the end of the study.

Frequency counts and percentages of all participants screened, enrolled, randomized, and receiving at least 1 dose of study drug will be summarized by treatment group. Of the randomized population, frequency counts and percentages of participants who completed the study, prematurely discontinued the study (and/or study drug), including reason for premature discontinuation, will be summarized by treatment group.

A Kaplan-Meier analysis of time from randomization to premature discontinuation from study and/or study treatment by treatment group will be provided.

9.4.2.2. Participant Characteristics

Demographics will be summarized for all enrolled participants.

Demographics will also be summarized by treatment group for all randomized participants.

9.4.2.3. Concomitant Therapy

Concomitant medication, including previous therapy, will be summarized by treatment group for all randomized participants.

9.4.2.4. Treatment Compliance

Frequency counts and percentages of participants compliant to study drug will be summarized by treatment arms and visits for FAS.

9.4.3. Efficacy Analyses

9.4.3.1. Primary Analyses

There will be 2 estimands of interest in comparing efficacy of tirzepatide MTD with placebo. For the FDA, the primary efficacy analysis will be guided by the "hybrid" estimand and will be conducted using the FAS. This assessment will analyze percent change in body weight obtained at the 72-week visit using an analysis of covariance (ANCOVA) and the percentage of participants achieving at least 5% body weight reduction obtained at the 72-week visit using a logistic regression model. Both models (ANCOVA and logistic regression model) will include terms of treatment, stratification factors, and baseline body weight as a covariate. Missing data solely due to a pandemic or natural disaster (after other reasons for missing data are ruled out) will be considered missing at random, and missing data will be imputed using all non-missing data of the primary outcome measurement from the same treatment arm. Missing data of the primary outcome measurement due to all other Intercurrent Events (ICEs) will be imputed based on retrieved dropouts in the same treatment arm, defined as observed primary outcome measurements from participants in the same treatment group who had their efficacy assessed after early discontinuation of study drug. In cases where there are not enough retrieved dropouts to provide a reliable imputation model (for example, the model implemented by the SAS program does not converge), an alternative multiple imputation method with reference to the placebo group (that is, placebo multiple imputation) will be used. Analysis will be conducted with multiple imputations.

For all other purposes, the primary efficacy analyses will be guided by the "efficacy" estimand, which represents efficacy prior to discontinuation of study drug, and conducted using the EAS. The primary analysis model will be an MMRM for body weight percentage change over time and a longitudinal logistic regression for proportion of participants achieving at least 5% body weight reduction over time. The response variable of the MMRM will be the percent change in body weight from baseline values obtained at each scheduled post baseline visit. The response variable of the longitudinal logistic regression will be the proportion of participants achieving at least 5% body weight reduction at each scheduled post randomization visit. The Kenward-Roger approximation will be used to estimate denominator degrees of freedom.

Since the mean percent change in body weight and proportion of participants with ≥5% body weight reduction needs to be achieved at the same time, no multiplicity adjustment is planned for these 2 tests.

9.4.3.2. Key Secondary Efficacy Analyses

- Superiority of tirzepatide MTD to placebo for the percentage of participants who maintain ≥80% of the body weight lost during the 12-week lead-in period
- Superiority of tirzepatide MTD to placebo for the proportion of participants who achieve ≥10% body weight reduction from randomization to 72 weeks
- Superiority of tirzepatide MTD to placebo for the proportion of participants who achieve ≥15% body weight reduction from randomization to 72 weeks
- Superiority of tirzepatide MTD to placebo for the mean change in waist circumference (cm) from randomization to 72 weeks

Additional details, including analysis methods for key secondary endpoints and the strategy for controlling the overall Type 1 error rate at a 2-sided alpha of 0.05 of primary and key secondary endpoint evaluation, will be provided in the SAP.

9.4.3.3. Other Secondary Efficacy Analyses

All other efficacy analyses will be guided by “efficacy” estimand and will be conducted using EAS. Details will be provided in the SAP.

9.4.4. Safety Analyses

Unless specified otherwise, safety assessments will be guided by an estimand comparing the safety of tirzepatide MTD with placebo irrespective of adherence to study drug. Thus, safety analyses will be conducted using SS.

9.4.4.1. Study Drug Exposure

Study drug exposure will be calculated for each participant and summarized by treatment group.

9.4.4.2. Adverse Events

Adverse events will be coded from the actual term using the Medical Dictionary for Regulatory Activities (MedDRA) and reported with preferred terms and system organ class. Counts and proportions of participants experiencing events will be reported for each treatment group, and Fisher’s exact test will be used to compare the treatment groups.

The proportion of participants experiencing TEAE, SAE, and discontinuation due to AE (DCAE) will be summarized by treatment group.

9.4.4.3. Special Safety Topics

This section includes areas of interest whether due to observed safety findings, potential findings based on drug class, or agreed upon consultation with regulatory agencies for the reasons previously mentioned.

The following AEs are AEs of special interest (AESI) for this study:

- Severe hypoglycemia
- Major adverse cardiovascular events (adjudicated). Includes, but not limited to cardiovascular death, nonfatal myocardial infarction, nonfatal stroke, and hospitalization for heart failure
- Treatment-emergent supraventricular arrhythmias and cardiac conduction disorders
- Hepatobiliary disorders. Includes biliary colic, cholecystitis, and other gallbladder disease
- Severe GI events
- Acute renal events
- MDD/suicidal behavior and ideation
- Pancreatitis (adjudicated)
- C-cell hyperplasia and thyroid malignancies
- Allergic/hypersensitivity reactions. Includes injection site reactions and ADA formation

Summaries and analyses for the incidence of AESIs will be provided by treatment. The details of analysis of AESI will be provided in the SAP.

9.4.4.4. Other Adverse Event Assessments

9.4.4.4.1. Gastrointestinal Events

Summaries and analyses for incidence and severity of nausea, vomiting, and diarrhea will be provided by each treatment.

9.4.4.4.2. Events Related to Potential Abuse Liability

Summaries and analyses for incidence of potential abuse liability TEAEs will be provided by treatment. The details will be provided in the SAP.

9.4.4.4.3. Depression, Suicidal Ideation and Behavior

In addition to the summary of TEAEs, suicidal ideation and behavior will be assessed by C-SSRS, and depression related symptoms will be assessed using the PHQ-9.

The analysis details will be provided in the SAP.

9.4.4.4.4. Central Laboratory Measures, Vital Signs, and Electrocardiograms

Actual values and change from randomization to post randomization values of central laboratory measures, vital signs, and selected ECG parameters will be summarized at each scheduled visit. Change from randomization to post randomization value will be summarized for participants who have both a randomization and at least 1 post randomization result.

The percentages of participants with TE abnormal, high, or low measures (including laboratory, vital, and ECG parameters) at any time will be summarized and compared between treatment groups using Fisher's exact test.

The analysis details will be provided in the SAP.

9.4.5. Pharmacokinetic/Pharmacodynamic Analyses

No PK/PD analyses are planned for this study.

9.4.6. Evaluation of Immunogenicity

The frequency and percentage of participants with preexisting ADA and with TE ADA+ to tirzepatide will be tabulated. Treatment-emergent ADAs are defined as those with a titer 2-fold (1 dilution) greater than the minimum required dilution (1:10) of the ADA assay if no ADAs were detected at baseline (treatment-induced ADA) or those with a 4-fold (2 dilutions) increase in titer compared to baseline if ADAs were detected at baseline (treatment-boosted ADA). For the TE ADA+ participants, the distribution of maximum titers will be described. The frequency of neutralizing antibodies, if assessed, and cross reactivity to native GIP and GLP-1 may also be tabulated in TE ADA+ participants.

The relationship between the presence of antibodies and the PK parameters and PD response, including safety and efficacy to tirzepatide, may be assessed.

9.4.7. Other Analyses

9.4.7.1. Health Outcomes

Analyses of actual and change from randomization in the domains and/or summary scores of patient-reported outcomes questionnaires will be conducted using the EAS. The details on questionnaire-specific analyses will be provided in the SAP.

9.4.7.2. Subgroup Analyses for Primary Analysis

Details of the subgroup analyses will be provided in the SAP.

The following subgroup variables will be considered (but not limited to):

- age (<65 years and ≥65 years)
- sex (female and male)
- race
- ethnicity
- BMI at study entry (<30, ≥30 and <35, ≥35 and <40, ≥40 kg/m^2)
- percent body weight loss at the end of lead-in (<10% and ≥10%)

The outcome measures for the subgroup analyses will include:

- percent change in body weight from randomization to 72 weeks
- proportion of participants achieving at least 5% body weight reduction from randomization to 72 weeks.

9.5. Interim Analyses

No interim analyses are planned for this study. If an unplanned interim analysis is deemed necessary for reasons other than a safety concern, the protocol must be amended.

9.6. Data Monitoring Committee (DMC)

Not applicable.

10. Supporting Documentation and Operational Considerations

10.1. Appendix 1: Regulatory, Ethical, and Study Oversight Considerations

10.1.1. Regulatory and Ethical Considerations

- This study will be conducted in accordance with the protocol and with the following:
    - Consensus ethical principles derived from international guidelines including the Declaration of Helsinki and Council for International Organizations of Medical Sciences (CIOMS) International Ethical Guidelines
    - Applicable ICH Good Clinical Practice (GCP) Guidelines
    - Applicable laws and regulations
- The protocol, protocol amendments, ICF, IB, and other relevant documents (for example, advertisements) must be submitted to an IRB/IEC by the investigator and reviewed and approved by the IRB/IEC before the study is initiated.
- Any amendments to the protocol will require IRB/IEC approval before implementation of changes made to the study design, except for changes necessary to eliminate an immediate hazard to study participants.
- Protocols and any substantial amendments to the protocol will require health authority approval prior to initiation except for changes necessary to eliminate an immediate hazard to study participants.
- The investigator will be responsible for the following:
    - Providing written summaries of the status of the study to the IRB/IEC annually or more frequently in accordance with the requirements, policies, and procedures established by the IRB/IEC
    - Notifying the IRB/IEC of SAEs or other significant safety findings as required by IRB/IEC procedures
    - Providing oversight of study conduct for participants under their responsibility and adherence to requirements of 21 Code of Federal Regulations (CFR), ICH guidelines, the IRB/IEC, European regulation 536/2014 for clinical studies (if applicable), and all other applicable local regulations
- Investigator sites are compensated for participation in the study as detailed in the clinical trial agreement (CTA).

10.1.2. Informed Consent Process

- The investigator or his/her representative will explain the nature of the study, including the risks and benefits, to the participant or his/her legally authorized representative and answer all questions regarding the study.

- Study participants must be informed that their participation is voluntary. Study participants or their legally authorized representative will be required to sign a statement of informed consent that meets the requirements of 21 CFR 50, local regulations, ICH guidelines, Health Insurance Portability and Accountability Act (HIPAA) requirements, where applicable, and the IRB/IEC or study center.
- The medical record must include a statement that written informed consent was obtained before the participant was entered in the study and the date the written consent was obtained. The authorized person obtaining the informed consent must also sign the ICF.
- Participants must be re-consented to the most current version of the ICF(s) during their participation in the study.
- A copy of the ICF(s) must be provided to the participant or the participant's legally authorized representative and is kept on file.

10.1.3. Data Protection

- Participants will be assigned a unique identifier by the sponsor. Any participant records, datasets or tissue samples that are transferred to the sponsor will contain the identifier only; participant names or any information which would make the participant identifiable will not be transferred.
- The participant must be informed that his/her personal study-related data will be used by the sponsor in accordance with local data protection law. The level of disclosure must also be explained to the participant who will be required to give consent for his/her data to be used as described in the informed consent.
- The participant must be informed that his/her medical records may be examined by Clinical Quality Assurance auditors or other authorized personnel appointed by the sponsor, by appropriate IRB/IEC members, and by inspectors from regulatory authorities.
- The sponsor has processes in place to ensure data protection, information security and data integrity. These processes include appropriate contingency plan(s) for appropriate and timely response in the event of a data security breach.

10.1.4. Committees Structure

Prospective adjudication of major adverse cardiovascular events and pancreatic AEs will be performed for this study. Sections 8.3.2.2, and 8.3.2.5 outline additional information on pancreatic and cardiovascular adjudication committees.

10.1.5. Dissemination of Clinical Study Data

Required clinical trial registries (for example, ClinicalTrials.gov) will be updated with the results from registered clinical trials regardless of the research outcome in accordance with local laws and regulations.

All CSRs, amendments and addenda will be submitted to external regulatory authorities, external partners (as applicable) and sites.

The publication policy for Study GPHM is outlined in Section 10.1.9 and further described in the CTA.

10.1.6. Data Quality Assurance

- All participant data relating to the study will be recorded on printed or electronic CRF unless transmitted to the sponsor or designee electronically (for example, laboratory data). The investigator is responsible for verifying that data entries are accurate and correct by physically or electronically signing the CRF.
- The investigator must maintain accurate documentation (source data) that supports the information entered in the CRF.
- The investigator must permit study-related monitoring, audits, IRB/IEC review, and regulatory agency inspections and provide direct access to source data documents.
- Monitoring details describing strategy (for example, risk-based initiatives in operations and quality such as risk management, mitigation strategies, and analytical risk-based monitoring), methods, responsibilities and requirements, including handling of noncompliance issues and monitoring techniques are provided in the Monitoring Plan.
- The sponsor or designee is responsible for the data management of this study including quality checking of the data.
- The sponsor assumes accountability for actions delegated to other individuals (for example, contract research organizations).
- Study monitors will perform ongoing source data verification to confirm that data entered into the CRF by authorized site personnel are accurate, complete, and verifiable from source documents; that the safety and rights of participants are being protected; and that the study is being conducted in accordance with the currently approved protocol and any other study agreements, ICH GCP, and all applicable regulatory requirements.
- Records and documents, including signed ICFs, pertaining to the conduct of this study must be retained by the investigator for the time period outlined in the CTA unless local regulations or institutional policies require a longer retention period. No records may be destroyed during the retention period without the written approval of the sponsor. No records may be transferred to another location or party without written notification to the sponsor.
- In addition, sponsor or its representatives will periodically check a sample of the participant data recorded against source documents at the study site. The study may be audited by sponsor or its representatives, and/or regulatory agencies at any time. Investigators will be given notice before an audit occurs.

Data Capture System

The investigator is responsible for ensuring the accuracy, completeness, legibility, and timeliness of the data reported to the sponsor.

An electronic data capture system (EDC) will be used in this study for the collection of CRF data. The investigator maintains a separate source for the data entered by the investigator or designee into the sponsor-provided EDC system. The investigator is responsible for the identification of any data to be considered source and for the confirmation that data reported are accurate and complete by signing the CRF.

Additionally, clinical outcome assessment (COA) data (participant-focused outcome instrument) and other data (for example, diary), will be collected by the participant, caregiver, or authorized study personnel, via a paper source document and will be transcribed by the authorized study personnel into the EDC system.

Data collected via the sponsor-provided data capture system(s) will be stored at third parties. The investigator will have continuous access to the data during the study and until decommissioning of the data capture system(s). Prior to decommissioning, the investigator will receive an archival copy of pertinent data for retention.

Data managed by a central vendor, such as laboratory test data, will be stored electronically in the central vendor’s database. Data will subsequently be transferred from the central vendor to the sponsor data warehouse.

Data from complaint forms submitted to sponsor will be encoded and stored in the global product complaint management system.

10.1.7. Source Documents

- Source documents provide evidence for the existence of the participant and substantiate the integrity of the data collected. Source documents are filed at the investigator’s site.
- Data reported on the CRF or entered in the eCRF that are transcribed from source documents must be consistent with the source documents or the discrepancies must be explained. The investigator may need to request previous medical records or transfer records, depending on the study. Also, current medical records must be available.
- Definition of what constitutes source data can be found in study training material.

10.1.8. Study and Site Start and Closure

The study start date is the date on which the clinical study will be open for recruitment of participants.

The sponsor or designee reserves the right to close the study site or terminate the study at any time for any reason at the sole discretion of the sponsor. Study sites will be closed upon study completion. A study site is considered closed when all required documents and study supplies have been collected and a study-site closure visit has been performed.

The investigator may initiate study-site closure at any time, provided there is reasonable cause and sufficient notice is given in advance of the intended termination.

Reasons for the early closure of a study site by the sponsor or investigator may include but are not limited to:

- Failure of the investigator to comply with the protocol, the requirements of the IRB/IEC or local health authorities, the sponsor's procedures, or GCP guidelines
- Inadequate recruitment of participants by the investigator
- Discontinuation of further study intervention development

If the study is prematurely terminated or suspended, the sponsor shall promptly inform the investigators, the IECs/IRBs, the regulatory authorities, and any contract research organization(s) used in the study of the reason for termination or suspension, as specified by the applicable regulatory requirements. The investigator shall promptly inform the participant and assure appropriate participant therapy and/or follow-up.

10.1.9. Publication Policy

In accordance with the sponsor’s publication policy, the results of this study will be submitted for publication by a peer-reviewed journal.

- The sponsor will comply with the requirements for publication of study results.
- Authorship will be determined by mutual agreement and in line with International Committee of Medical Journal Editors authorship requirements.
- The publication policy for Study GPHM is described in the CTA.

10.2. Appendix 2: Clinical Laboratory Tests

- The tests detailed below will be performed by a central lab unless designated as local in the SoA and in the table below.
- In circumstances where the sponsor approves local laboratory testing in lieu of central laboratory testing (in the table below), the local laboratory must be qualified in accordance with applicable local regulations.
- Protocol-specific requirements for inclusion or exclusion of participants are detailed in Section 5 of the protocol.
- Additional tests may be performed at any time during the study as determined necessary by the investigator or required by local regulations.
- Pregnancy testing (Refer to Section 5.1 Inclusion Criteria for screening pregnancy criteria).
- Investigators must document their review of each laboratory safety report.
- Laboratory results that could unblind the study will not be reported to investigative sites or other blinded personnel and are denoted in the table below.

Clinical Laboratory Tests

**Hematology**[a]	**Clinical Chemistry**[a]
Hemoglobin	Bicarbonate
Hematocrit	Sodium
Erythrocyte count (RBC)	Potassium
Mean cell volume	Total bilirubin
Mean cell hemoglobin concentration	Direct bilirubin
Leukocytes (WBC)	Alkaline phosphatase
Neutrophils, segmented	Alanine aminotransferase (ALT)
Lymphocytes	Aspartate aminotransferase (AST)
Monocytes	Blood urea nitrogen (BUN)
Eosinophils	Creatinine
Basophils	Uric acid
Platelets	Calcium
	Glucose
**Urine Chemistries**[a]	Albumin
Albumin	Creatine kinase (CK)
Creatinine	
**Cystatin-C**[a]	**Hormones (females)**
	Pregnancy Test serum [a] and/or urine (local)
	Follicle-stimulating hormone (FSH)[a]
**HbA1c**[a]	
**Endocrine**[a]	
Calcitonin	**Pancreas (exocrine)**[a]
Thyroid-stimulating hormone (TSH)	Pancreatic amylase
	Lipase
**Nonpharmacogenetic Stored Samples**[a,b]	**Immunogenicity**[a,b]
Serum	Anti-tirzepatide antibodies
EDTA plasma	Anti-tirzepatide antibody neutralization
P800 plasma	Pharmacokinetic Sample for Immunogenicity
	**Lipid Panel**[a]
	Triglycerides
**Pharmacogenetics Sample**[a,b]	VLDL-C
Whole blood (EDTA)	HDL-C
	LDL-C[c]
	Total cholesterol
**Free Fatty Acids**[a]	**Calculations** [a]
**Insulin**[a]	eGFR (calculated by CKD-EPI equation)
**C-peptide**[a]	UACR

Abbreviations: CKD-EPI = Chronic Kidney Disease-Epidemiology; eGFR = estimated glomerular filtration rate; EDTA = ethylenediaminetetraacetic acid; HbA1c = hemoglobin A1c; HDL-C = high-density lipoprotein cholesterol; LDL-C = low-density lipoprotein cholesterol; RBC = red blood cells; UACR = urine albumin to creatinine ratio; VLDL-C = very low-density lipoprotein cholesterol; WBC = white blood cells.

[a] All tests will be performed by a Lilly-designated central laboratory, unless otherwise noted.

[b] Results will not be provided to the investigative sites.

[c] This value will be calculated. If triglycerides are >400 mg/dL, the direct LDL will be assayed.

10.3. Appendix 3: Laboratory Assessments for Hypersensitivity Events

- Laboratory assessments should be performed if the participant experiences generalized urticaria or if anaphylaxis is suspected.
- Collect sample after the participant has been stabilized, and within 1 to 2 hours of the event; however, samples may be obtained as late as 12 hours after the event as analytes can remain altered for an extended period of time. Record the time at which the sample was collected.
- Obtain a follow-up sample at the next regularly scheduled visit or after 4 weeks, whichever is later.

The table below summarizes the laboratory parameters that will be evaluated. These laboratory tests are bundled in the hypersensitivity laboratory testing kit.

Clinical Lab Tests for Hypersensitivity Events

Hypersensitivity Tests	Notes<br>Selected test may be obtained in the event of anaphylaxis or systemic allergic/hypersensitivity reactions.
Tirzepatide anti-drug antibodies (immunogenicity/ADA)	Assayed by Lilly-designated laboratory. Results will not be provided to the investigative sites.
Tirzepatide concentrations (PK)	Assayed by Lilly-designated laboratory. Results will not be provided to the investigative sites.
Tryptase	Assayed by Lilly-designated laboratory. Results will not be provided to the investigative sites.<br>**Note:** If a tryptase sample is obtained more than 2 hours after the event (that is, within 2 to 12 hours), or is not obtained because more than 12 hours have lapsed since the event, obtain urine sample for N-methylhistamine testing. Note that for tryptase serum samples obtained within 2 to 12 hours of the event, urine N-methylhistamine testing is performed in addition to tryptase testing. Collect the first void urine sample following the event. Obtain a follow-up urine sample for N-methylhistamine testing at the next regularly scheduled visit or after 4 weeks, whichever is later.
N-methylhistamine	Assayed by Lilly-designated laboratory. Results will not be provided to the investigative sites.
Drug-specific IgE	Will be performed if a validated assay is available.<br>Assayed by Lilly-designated laboratory. Results will not be provided to the investigative sites.
Basophil activation test	Will be performed if a validated assay is available.<br>Assayed by Lilly-designated laboratory. Results will not be provided to the investigative sites.<br>***Note:*** The basophil activation test is an in vitro cell based assay that only requires a serum sample. It is a surrogate assay for drug specific IgE, but is not specific for IgE.
Complement (C3, C3a, and C5a)	Assayed by Lilly-designated laboratory. Results will not be provided to the investigative sites.
Cytokine panel (IL-6, IL-1β, IL-10)	Assayed by Lilly-designated laboratory. Results will not be provided to the investigative sites.

Abbreviations: ADA = anti-drug antibody; IgE = immunoglobulin E; IL = interleukin; PK = pharmacokinetic.

10.4. Appendix 4: Adverse Events: Definitions and Procedures for Recording, Evaluating, Follow-up, and Reporting

10.4.1. Definition of AE

**AE Definition**
• An AE is any untoward medical occurrence in a participant administered a pharmaceutical product and which does not necessarily have a causal relationship with the study intervention. An AE can therefore be any unfavourable and unintended sign (including an abnormal laboratory finding), symptom, or disease (new or exacerbated) temporally associated with the use of a medicinal (investigational) product, whether or not related to the medicinal (investigational) product.

**Events Meeting the AE Definition**
• Any abnormal laboratory test results (hematology, clinical chemistry, or urinalysis) or other safety assessments (for example, ECG, radiological scans, vital signs measurements), including those that worsen from baseline, considered clinically significant in the medical and scientific judgment of the investigator (that is, not related to progression of underlying disease).<br>• Exacerbation of a chronic or intermittent pre-existing condition including either an increase in frequency and/or intensity of the condition.<br>• New conditions detected or diagnosed after intensive lifestyle intervention or study drug intervention administration even though they may have been present before the start of the study.<br>• Signs, symptoms, or the clinical sequelae of a suspected drug-drug interaction.<br>• Signs, symptoms, or the clinical sequelae of a suspected overdose of either study drug intervention or a concomitant medication.<br>• “Lack of efficacy” or “failure of expected pharmacological action” per se will not be reported as an AE or SAE. Such instances will be captured in the efficacy assessments. However, the signs, symptoms, and/or clinical sequelae resulting from lack of efficacy will be reported as an AE or SAE if they fulfill the definition of an AE or SAE.

**Events NOT Meeting the AE Definition**
• Any clinically significant abnormal laboratory findings or other abnormal safety assessments which are associated with the underlying disease, unless judged by the investigator to be more severe than expected for the participant’s condition.<br>• The disease/disorder being studied or expected progression, signs, or symptoms of the disease/disorder being studied, unless more severe than expected for the participant’s condition.<br>• Medical or surgical procedure (for example, endoscopy, appendectomy): the condition

that leads to the procedure is the AE.<br>• Situations in which an untoward medical occurrence did not occur (social and/or convenience admission to a hospital).<br>• Anticipated day-to-day fluctuations of pre-existing disease(s) or condition(s) present or detected at the start of the study that do not worsen.

10.4.2. Definition of SAE

If an event is not an AE per definition above, then it cannot be an SAE even if serious conditions are met (for example, hospitalization for signs/symptoms of the disease under study, death due to progression of disease).

**SAE is defined as any untoward medical occurrence that, at any dose:**
**a. Results in death**
**b. Is life-threatening**<br>The term 'life-threatening' in the definition of 'serious' refers to an event in which the participant was at risk of death at the time of the event. It does not refer to an event, which hypothetically might have caused death, if it were more severe.
**c. Requires inpatient hospitalization or prolongation of existing hospitalization**<br>• In general, hospitalization signifies that the participant has been admitted to hospital for observation and/or treatment that would not have been appropriate in the physician's office or outpatient setting. Complications that occur during hospitalization are AEs. If a complication prolongs hospitalization or fulfills any other serious criteria, the event is serious. When in doubt as to whether "hospitalization" occurred or was necessary, the AE should be considered serious.<br>• Hospitalization for elective treatment of a pre-existing condition that did not worsen from baseline is not considered an AE.
**d. Results in persistent disability/incapacity**<br>• The term disability means a substantial disruption of a person's ability to conduct normal life functions.<br>• This definition is not intended to include experiences of relatively minor medical significance such as uncomplicated headache, nausea, vomiting, diarrhea, influenza, and accidental trauma (for example, sprained ankle) which may interfere with or prevent everyday life functions but do not constitute a substantial disruption.
**e. Is a congenital anomaly/birth defect**<br>• Abnormal pregnancy outcomes (for example, spontaneous abortion, fetal death, stillbirth, congenital anomalies, ectopic pregnancy) are considered SAEs.

**f.**	**Other situations:**<br>• Medical or scientific judgment should be exercised in deciding whether SAE reporting is appropriate in other situations such as important medical events that may not be immediately life-threatening or result in death or hospitalization but may jeopardize the participant or may require medical or surgical intervention to prevent one of the other outcomes listed in the above definition. These events should usually be considered serious.<br>• Examples of such events include invasive or malignant cancers, intensive treatment in an emergency room or at home for allergic bronchospasm, blood dyscrasias or convulsions that do not result in hospitalization, or development of drug dependency or drug abuse.
**g.**	Resulted in medical or surgical intervention to prevent life-threatening illness or injury or permanent impairment to a body structure or a body function.

10.4.3. Definition of Product Complaint

**Product Complaint**
• A product complaint is any written, electronic, or oral communication that alleges deficiencies related to the identity, quality, durability, reliability, safety, effectiveness or performance of a study intervention. When the ability to use the study intervention safely is impacted, the following are also product complaints:<br>o Deficiencies in labeling information, and<br>o Use errors for device or drug-device combination products due to ergonomic design elements of the product.<br>• Product complaints related to study interventions used in clinical trials are collected in order to ensure the safety of participants, monitor quality, and to facilitate process and product improvements.<br>• Investigators will instruct participants to contact the site as soon as possible if he or she has a product complaint or problem with the study intervention so that the situation can be assessed.<br>• An event may meet the definition of both a product complaint and an AE/SAE. In such cases, it should be reported as both a product complaint and as an AE/SAE.

10.4.4. Recording and Follow-Up of AE and/or SAE and Product Complaints

**AE, SAE, and Product Complaint Recording**
• When an AE/SAE/product complaint occurs, it is the responsibility of the investigator to review all documentation (for example, hospital progress notes, laboratory reports, and diagnostics reports) related to the event.<br>• The investigator will then record all relevant AE/SAE/product complaint information in the participant's medical records, in accordance with the investigator's normal clinical practice. Adverse event/SAE information is reported on the appropriate (e)CRF page and

product complaint information is reported on the Product Complaint form.

Note: An event may meet the definition of both a product complaint and an AE/SAE. In such cases, it should be reported as both a product complaint and as an AE/SAE.

- It is **not** acceptable for the investigator to send photocopies of the participant's medical records to the sponsor or designee in lieu of completion of the (e)CRF page for AE/SAE and the Product Complaint form for product complaints.
- There may be instances when copies of medical records for certain cases are requested by sponsor or designee. In this case, all participant identifiers, with the exception of the participant number, will be redacted on the copies of the medical records before submission to the sponsor or designee.
- The investigator will attempt to establish a diagnosis of the event based on signs, symptoms, and/or other clinical information. Whenever possible, the diagnosis (not the individual signs/symptoms) will be documented as the AE/SAE.

Assessment of Intensity

The investigator will make an assessment of intensity for each AE and SAE reported during the study and assign it to 1 of the following categories:

- Mild: A type of AE that is usually transient and may require only minimal treatment or therapeutic intervention. The event does not generally interfere with usual activities of daily living.
- Moderate: A type of AE that is usually alleviated with additional specific therapeutic intervention. The event interferes with usual activities of daily living, causing discomfort but poses no significant or permanent risk of harm to the research participant.
- Severe: A type of AE that interrupts usual activities of daily living, significantly affects clinical status, or may require intensive therapeutic intervention. An AE that is assessed as severe should not be confused with an SAE. Severe is a category utilized for rating the intensity of an event, and both AEs and SAEs can be assessed as severe.

An event is defined as 'serious' when it meets at least 1 of the predefined outcomes as described in the definition of an SAE, NOT when it is rated as severe.

Assessment of Causality

- The investigator is obligated to assess the relationship between intensive lifestyle intervention or study drug intervention and each occurrence of each AE/SAE.
- A "reasonable possibility" of a relationship conveys that there are facts, evidence, and/or arguments to suggest a causal relationship, rather than a relationship cannot be ruled out.
- The investigator will use clinical judgment to determine the relationship.
- Alternative causes, such as underlying disease(s), concomitant therapy, and other risk factors, as well as the temporal relationship of the event to intensive lifestyle intervention or study drug intervention administration will be considered and investigated.

- The investigator will also consult the IB and/or Product Information, for marketed products, in his/her assessment.
- For each AE/SAE, the investigator **must** document in the medical notes that he/she has reviewed the AE/SAE and has provided an assessment of causality.
- There may be situations in which an SAE has occurred and the investigator has minimal information to include in the initial report to the sponsor or designee. However, it is very important that the investigator always make an assessment of causality for every event before the initial transmission of the SAE data to the sponsor or designee.
- The investigator may change his/her opinion of causality in light of follow-up information and send an SAE follow-up report with the updated causality assessment.
- The causality assessment is one of the criteria used when determining regulatory reporting requirements.

**Follow-up of AEs and SAEs**
• The investigator is obligated to perform or arrange for the conduct of supplemental measurements and/or evaluations as medically indicated or as requested by the sponsor or designee to elucidate the nature and/or causality of the AE or SAE as fully as possible. This may include additional laboratory tests or investigations, histopathological examinations, or consultation with other health care professionals.<br>• If a participant dies during participation in the study or during a recognized follow-up period, the investigator will provide the sponsor or designee with a copy of any post-mortem findings, including histopathology.

10.4.5. Reporting of SAEs

**SAE Reporting via an Electronic Data Collection Tool**
• The primary mechanism for reporting an SAE will be the electronic data collection tool.<br>• If the electronic system is unavailable, then the site will use the paper SAE data collection tool (see next section) in order to report the event within 24 hours.<br>• The site will enter the SAE data into the electronic system as soon as it becomes available.<br>• After the study is completed at a given site, the electronic data collection tool will be taken off-line to prevent the entry of new data or changes to existing data.<br>• If a site receives a report of a new SAE from a study participant or receives updated data on a previously reported SAE after the electronic data collection tool has been taken off-line, then the site can report this information on a paper SAE form (see next section) or to the sponsor contact for SAE reporting by telephone.<br>• Contacts for SAE reporting can be found in study training material.

**SAE Reporting via Paper CRF**
• Facsimile transmission of the SAE paper CRF is the preferred method to transmit this

information to the sponsor contacts for SAE reporting.<br>• Initial notification via telephone does not replace the need for the investigator to complete and sign the SAE CRF pages within the designated reporting time frames.<br>• Contacts for SAE reporting can be found in study training material.

10.4.6. Regulatory Reporting Requirements

SAE Regulatory Reporting
• Prompt notification by the investigator to the sponsor of an SAE is essential so that legal obligations and ethical responsibilities towards the safety of participants and the safety of a study intervention under clinical investigation are met.<br>• The sponsor has a legal responsibility to notify both the local regulatory authority and other regulatory agencies about the safety of a study intervention under clinical investigation. The sponsor will comply with country-specific regulatory requirements relating to safety reporting to the regulatory authority, IRB/IEC, and investigators.<br>• An investigator who receives an investigator safety report describing an SAE or other specific safety information (for example, summary or listing of SAEs) from the sponsor will review and then file it along with the IB and will notify the IRB/IEC, if appropriate, according to local requirements.

10.5. Appendix 5: Contraceptive Guidance and Collection of Pregnancy Information

Definitions:

Woman of Childbearing Potential (WOCBP)

A woman is considered fertile following menarche and until becoming post-menopausal unless permanently sterile (see below).

If fertility is unclear (for example, amenorrhea in adolescents or athletes) and a menstrual cycle cannot be confirmed before first dose of study intervention, additional evaluation should be considered.

Women in the following categories are not considered women of childbearing potential

Article I. Premenarchal

Article II. Premenopausal female with 1 of the following:

- Documented hysterectomy
- Documented bilateral salpingectomy
- Documented bilateral oophorectomy

For individuals with permanent infertility due to an alternate medical cause other than the above, (for example, Mullerian agenesis, androgen insensitivity), investigator discretion should be applied to determining study entry.

Note: Determination can come from the site personnel's review of the participant's medical records, medical examination, or medical history interview.

Article III. Postmenopausal female

- A postmenopausal state is defined as either
  - A woman at least 40 years of age with an intact uterus, not on hormone therapy, who has cessation of menses for at least 1 year without an alternative medical cause, AND an FSH ≥40 mIU/mL; or
  - A woman 55 or older not on hormone therapy, who has had at least 12 months of spontaneous amenorrhea; or
  - A woman at least 55 years of age with a diagnosis of menopause prior to starting hormone replacement therapy (HRT).
- Females on HRT and whose menopausal status is in doubt will be required to use one of the nonestrogen hormonal highly effective contraception methods if they wish to continue their HRT during the study. Otherwise, they must discontinue HRT to allow confirmation of postmenopausal status before study enrollment.

Contraception Guidance:

Contraceptive use by men and women should be consistent with local regulations regarding the methods of contraception for those participating in clinical studies.

Two forms of effective contraception, where at least 1 form is highly effective, will be used. Effective contraception may be used as the second therapy. Barrier protection methods without concomitant use of a spermicide are not a reliable or acceptable method. The use of male and female condoms as a double barrier method is not considered acceptable due to the high failure rate when these methods are combined.

Highly Effective Methods of Contraception:

- Combined oral contraceptive pill and mini pill
- NuvaRing®
- Implantable contraceptives
- Injectable contraceptives (such as Depo-Provera®)
- Intrauterine device (such as Mirena® and ParaGard®)
- Contraceptive patch – ONLY women <198 pounds or 90 kg
- Total abstinence (if this is their preferred and usual lifestyle) or in a same-sex relationship with no sexual relationship with males (as part of their preferred and usual lifestyle), and agrees to maintain this status throughout trial follow-up

Note: periodic abstinence (for example, calendar, ovulation, symptothermal, and postovulation methods), declaration of abstinence just for the duration of a trial, and withdrawal are not acceptable methods of contraception

- Vasectomy – for men in clinical studies

Note: Implantable contraceptives and injectable contraceptives (such as Depo Provera) are only permitted if started more than 18 months prior to screening. Participants should not start these methods of contraception after being enrolled in the study.

Effective Methods of Contraception (must use combination of 2 methods):

- Male condom with spermicide
- Female condom with spermicide
- Diaphragm with spermicide
- Cervical sponge
- Cervical cap with spermicide

Men, regardless of their fertility status, with nonpregnant women of childbearing potential partners must agree to either remain abstinent (if this is their preferred and usual lifestyle) or use condoms plus 1 additional highly effective (less than 1% failure rate) method of contraception (such as combination oral contraceptives, implanted contraceptives, or intrauterine device) or effective method of contraception (such as diaphragms with spermicide or cervical sponge) for the duration of the study and for 5 half-lives of study drug plus 90 days, which is approximately 4 months after the last injection. Periodic abstinence (for example, calendar, ovulation,

symptothermal, and postovulation methods), declaration of abstinence just for the duration of a trial, and withdrawal are not acceptable methods of contraception.

Men with pregnant partners should use condoms during intercourse for the duration of the study and until the end of estimated relevant potential exposure in women of childbearing potential.

Men who are abstinent (if this is complete abstinence, as their preferred and usual lifestyle) or in a same-sex relationship (as part of their preferred and usual lifestyle) must agree to either remain abstinent or stay in a same-sex relationship without sexual relationships with females. In these situations, men are not required to use contraception.

Men should refrain from sperm donation for the duration of the study and for 5 half-lives of study drug plus 90 days after the last dose of study drug, corresponding to 4 months after the last injection.

Collection of Pregnancy Information

Male participants with partners who become pregnant

- The investigator will attempt to collect pregnancy information on any male participant’s female partner who becomes pregnant while the male participant is in this study.
- After obtaining the necessary signed informed consent from the pregnant female partner directly, the investigator will record pregnancy information on the appropriate form and submit it to the sponsor within 24 hours of learning of the partner’s pregnancy. If pregnancy occurs during study drug intervention period, the female partner will also be followed to determine the outcome of the pregnancy. Information on the status of the mother and child will be forwarded to the sponsor. Generally, the follow-up will be no longer than 6 to 8 weeks following the estimated delivery date. Any termination of the pregnancy will be reported regardless of fetal status (presence or absence of anomalies) or indication for the procedure.

Female Participants who become pregnant

- The investigator will collect pregnancy information on any female participant who becomes pregnant while participating in this study. The initial information will be recorded on the appropriate form and submitted to the sponsor within 24 hours of learning of a participant's pregnancy.
- The participants who become pregnant during the study drug intervention period will be followed to determine the outcome of the pregnancy. The investigator will collect follow-up information on the participant and the neonate and the information will be forwarded to the sponsor. Generally, follow-up will not be required for longer than 6 to 8 weeks beyond the estimated delivery date. Any termination of pregnancy will be reported, regardless of fetal status (presence or absence of anomalies) or indication for the procedure.

- While pregnancy itself is not considered to be an AE or SAE, any pregnancy complication or elective termination of a pregnancy for medical reasons will be reported as an AE or SAE.
- A spontaneous abortion (occurring at <20 weeks gestational age) or still birth (occurring at >20 weeks gestational age) is always considered to be an SAE and will be reported as such.
- Any post-study pregnancy related SAE considered reasonably related to the study intervention by the investigator will be reported to the sponsor as described in protocol. While the investigator is not obligated to actively seek this information in former study participants, he or she may learn of an SAE through spontaneous reporting.
- Any female participant who becomes pregnant while participating in the study will discontinue intensive lifestyle intervention or study drug intervention. If the participant is discontinued from the study intervention, follow the standard discontinuation process for each study period.

10.6. Appendix 6: Liver Safety: Suggested Actions and Follow-up Assessments

- For testing selected, analysis is required to be completed by the Lilly designated central laboratory, except for microbiology.
- Local testing may be performed in addition to central testing when required for immediate participant management.
- Results will be reported if a validated test or calculation is available.

Hepatic Evaluation Labs

**Hematology**	**Clinical Chemistry**
Hemoglobin	Total bilirubin
Hematocrit	Direct bilirubin
Erythrocytes (RBCs - red blood cells)	Alkaline phosphatase (ALP)
Leukocytes (WBCs - white blood cells)	Alanine aminotransferase (ALT)
Differential:	Aspartate aminotransferase (AST)
Neutrophils, segmented	Gamma-glutamyl transferase (GGT)
Lymphocytes	Creatine kinase (CK)
Monocytes	**Other Chemistry**
Basophils	Acetaminophen
Eosinophils	Acetaminophen protein adducts
Platelets	Alkaline phosphatase isoenzymes
Cell morphology (RBC and WBC)	Ceruloplasmin
**Coagulation**	Copper
Prothrombin Time, INR (PT-INR)	Ethyl Alcohol
**Serology**	Haptoglobin
Hepatitis A Virus (HAV) testing:	IgA (Quantitative)
HAV total antibody	IgG (Quantitative)
HAV IgM antibody	IgM (Quantitative)
Hepatitis B Virus (HBV) Testing:	Phosphatidylethanol (PEth)
Hepatitis B surface antigen (HBsAg)	**Urine Chemistry**
Hepatitis B surface antibody (Anti-HBs)	Drug Screen
Hepatitis B core total antibody (Anti-HBc)	Ethyl glucuronide (EtG)
Hepatitis B core IgM antibody	**Other Serology**
Hepatitis B core IgG antibody	Anti-nuclear antibody (ANA)

**Hematology**	**Clinical Chemistry**
HBV DNA[a]	Anti-smooth muscle antibody (ASMA) [b]
Hepatis C Virus (HCV) Testing:	Anti-actin antibody[c]
HCV antibody	Epstein-Barr Virus (EBV) Testing:
HCV RNA[a]	EBV antibody
Hepatitis D Virus (HDV) Testing:	EBV DNA[a]
HDV antibody	Cytomegalovirus (CMV) Testing:
Hepatitis E Virus (HEV) Testing:	CMV antibody
HEV IgG antibody	CMV DNA[a]
HEV IgM antibody	Herpes Simplex Virus (HSV) Testing:
HEV RNA[a]	HSV (Type 1 and 2) antibody
**Microbiology**[d]	HSV (Type 1 and 2) DNA[a]
Culture:	Liver Kidney Microsomal Type 1 (LKM-1) Antibody
Blood	
Urine	

Abbreviations: Ig = immunoglobulin; INR = international normalized ratio.

[a] Reflex/confirmation dependent on regulatory requirements, testing availability, or both.

[b] This is not required if anti-actin antibody is tested.

[c] This is not required if anti-smooth muscle antibody is tested.

[d] Assayed by investigator-designated local laboratory ONLY. No central testing available.

10.7. Appendix 7: Protocol GPHM Standardized Protocols for the Measurement of Height, Weight, Waist Circumference, Vital Signs and Electrocardiogram

The following information has been adapted from standardized physical measurement protocols for the World Health Organization's STEPwise approach to Surveillance (STEPS) (WHO 2017)

Measuring Height

Step 1. Ask the participant to remove their footwear and any headgear (light headgear worn for religious reasons can remain, but this should be worn by the participant at every clinic visit when their height is measured).

Step 2. Ask the participant to stand on the calibrated height measuring board (stadiometer) or against a wall with their feet together and their knees straight with their heels against the backboard, the stadiometer or the wall.

Step 3. Ask the participant to look straight ahead without tilting their head up.

Step 4. Ask the participant to breathe in and stand tall. Measure and record the participant's height in centimeters (cm) to one decimal place.

Measuring Weight

- Body weight measurements should be done in a consistent manner using a calibrated electronic scale capable of measuring weight in kilograms (kg) to one decimal place.
- All weights for a given participant should be measured using the same scale, whenever possible, at approximately the same time in the morning after evacuation of bladder contents.
- Body weight must be measured in fasting state. If the participant is not fasting, the participant should be called in for a new visit within the visit window to have the fasting body weight measured.

Step 1. Ask the participant to empty their pockets, remove their footwear, outerwear (coat, jacket, etc.), and any headgear (light headgear worn for religious reasons can remain, but this should be worn by the participant at every clinic visit when weight is measured).

Step 2. Make sure the scale is placed on a firm, flat, even surface (not on carpet, on a sloping surface, or a rough, uneven surface).

Step 3. Ask the participant to step onto the scale with 1 foot on each side of the scale.

Step 4. Ask the participant to stand still with arms by sides and then record weight in kilograms (kg) to the nearest one-tenth kilogram.

Measuring Waist Circumference

- Waist circumference should be measured in the horizontal plane and at the midpoint between the lower margin of the last palpable rib and the top of the iliac crest.
- Measurements should be taken at the end of a normal expiration using a non-stretchable measuring tape. The tape should lie flat against the skin without compressing the soft tissue.

- The waist circumference should be measured twice, rounded to the nearest 0.5 cm. The measuring tape should be removed between the 2 measurements. Both measurements will be recorded in the eCRF. If the difference between the 2 measurements exceeds 1 cm, this set of measurements should be discarded and the 2 measurements repeated.

Step 1: Ask the participant to wear little clothing (if available, patient gowns could also be used).

Step 2: Ask the participant to stand with their feet close together, arms at their side, body weight evenly distributed.

Step 3: Ask the participant to relax and measure the participant's waist circumference.

Vital Sign Measurements

- Vital sign measurements (blood pressure and heart rate, measured by pulse) should be taken before obtaining an ECG tracing and before collection of blood samples for laboratory testing
- The participant should sit quietly for 5 minutes before vital signs measurements are taken
- For each parameter, 2 measurements will be taken using the same arm, preferably the nondominant arm
- The recordings should be taken at least 1 minute apart. Each measurement of sitting pulse and BP needs to be recorded in the eCRF
- Blood pressure must be taken with an automated blood pressure instrument.
- If blood pressure and pulse measurements are taken separately, pulse should be taken prior to blood pressure.

Note: In the event pulse measurement cannot be taken via an automated blood pressure instrument, the preferred location for measurement of pulse is the radial artery.

Electrocardiogram

- All digital ECGs will be obtained using centrally provided ECG machines and will be electronically transmitted to a designated central ECG laboratory.
- 12-lead ECGs should be obtained after the subject has rested in a supine position for at least 10 minutes.
- Electrocardiograms should be collected at least 30 minutes prior to collection of blood samples for laboratory testing, including PK samples (collected with immunogenicity testing).

10.8. Appendix 8: Suggested Visit Structure

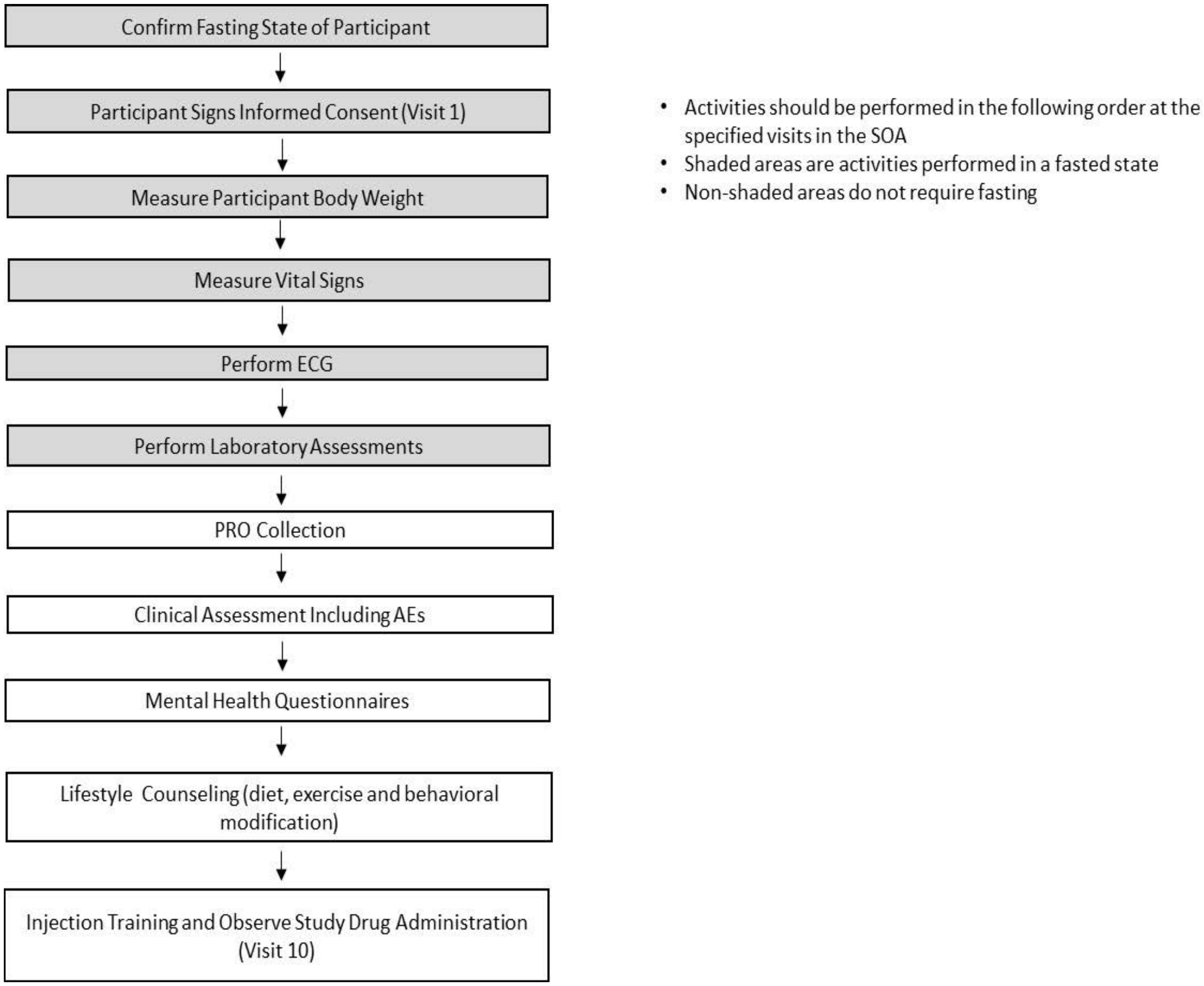

Abbreviations: AE=adverse event; ECG=electrocardiogram; PRO=patient reported outcomes; SoA=Schedule of Activities

10.9. Appendix 9: Management of Gastrointestinal Symptoms

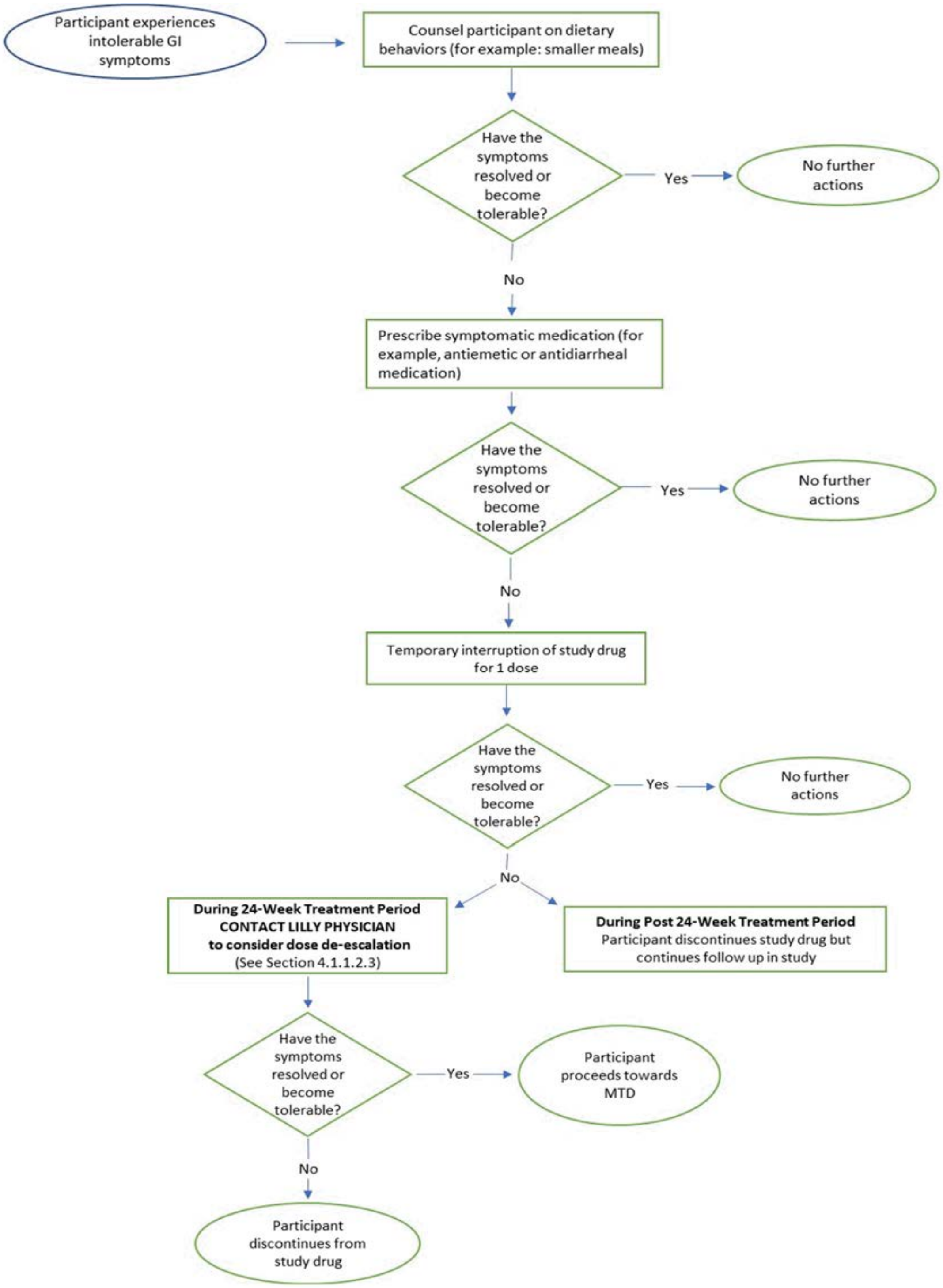

Abbreviation: GI= gastrointestinal

10.10. Appendix 10: Provisions for Changes in Study Conduct During Exceptional Circumstances

Implementation of this appendix

The changes to procedures described in this appendix are temporary measures intended to be used only during specific time periods as directed by the sponsor in partnership with the investigator.

Exceptional circumstances

Exceptional circumstances are rare events that may cause disruptions to the conduct of the study. Examples include pandemics or natural disasters. These disruptions may limit the ability of the investigators, participants, or both to attend on-site visits or to conduct planned study procedures.

Implementing changes under exceptional circumstances

In an exceptional circumstance, after receiving the sponsor's written approval, sites may implement changes if permitted by local regulations.

After approval by local ERBs, regulatory bodies and any other relevant local authorities, implementation of these exceptional circumstance changes will not typically require additional notification to these groups, unless they have specific conditions in which notification is required. To protect the safety of study participants, urgent changes may be implemented before approval but need to be reported as soon as possible. All approvals must be retained in the study records.

If the sponsor grants written approval for changes in study conduct, the sponsor will also provide additional written guidance, if needed.

Considerations for making a change

The prevailing consideration for making a change is ensuring the safety of study participants. Additional important considerations for making a change are compliance with GCP, enabling participants to continue safely in the study and maintaining the integrity of the study.

Informed consent

Additional consent from the participant will be obtained, if required, for:

- participation in remote visits, as defined in Section "Remote visits,"
- dispensation of additional study intervention during an extended treatment period,
- alternate delivery of study intervention and ancillary supplies, and
- provision of their personal or medical information required prior to implementation of these activities.

Changes in study conduct during exceptional circumstances

Changes in study conduct not described in this appendix, or not consistent with applicable local regulations, are not allowed.

The following changes in study conduct will not be considered protocol deviations.

1. Remote visits

In source documents and the CRF, the study site should capture the visit method, with a specific explanation for the reason of conducting a remote visit instead of an onsite visit and for any data missing because of missed in-person site visits.

Telemedicine:
Telephone or technology-assisted virtual visits, or both, are acceptable to complete appropriate assessments. Assessments to be completed in this manner include, but are not limited to, concomitant medications, review of study participant diary (including study drug compliance), review diet and exercise goals, lifestyle counseling, C-SSRS (Since Last Visit Version), Self-Harm Supplement Form, Self-Harm Follow-up Form (if applicable), and PHQ-9.

Mobile healthcare:

Healthcare visits may be performed by a mobile healthcare provider at locations other than the study site when participants cannot travel to the site due to an exceptional circumstance if written approval is provided by the sponsor. Procedures performed at such visits include, but are not limited to, weight and waist measurements, physical assessments, vital signs, ECG, PRO questionnaires administration, collection of blood samples and health information.

Other alternative locations:
Laboratory draws may be done at an alternate location in exceptional circumstances.

Regardless of the type of remote visits implemented, the protocol requirements regarding the reporting of AEs, SAEs, and product complaints remain unchanged. Furthermore, every effort should be made to enable participants to return to on-site visits as soon as reasonably possible, while ensuring the safety of both the participants and the site staff.

2. Local laboratory testing option

Local laboratory testing may be conducted in lieu of central laboratory testing. The local laboratory must be qualified in accordance with applicable local regulations.

3. Study intervention and ancillary supplies (including participant diaries)

When a participant is unable to go to the site to receive study supplies during normal on-site visits, the site should work with the sponsor to determine appropriate actions. These actions may include:

- asking the participant to go to the site and receive study supplies from site staff without completion of a full study visit,
- asking the participant's designee to go to the site and receive study supplies on a participant's behalf, and
- arranging delivery of study supplies.

These requirements must be met before action is taken:

- Alternate delivery of study intervention should be performed in a manner that does not compromise treatment blinding and ensures product integrity. The existing protocol requirements for product accountability remain unchanged, including verification of participant's receipt of study supplies.
- When delivering supplies to a location other than the study site (for example, participant's home), the investigator, sponsor, or both should ensure oversight of the shipping process to ensure accountability and product quality (that is, storage conditions maintained and intact packaging upon receipt).
- Instructions may be provided to the participant or designee on the final disposition of any unused or completed study supplies.

4. Screening period guidance

To ensure safety of study participants, laboratory values and other eligibility assessments taken at or prior to Visit 2 are valid for a maximum of 90 days. The following rules will be applied for active, not yet enrolled participants whose participation in the study must be paused due to exceptional circumstances:

- If paused for less than 90 days from Visit 1 to Visit 2: the participant will proceed to the next study visit per the usual Schedule of Activities, provided that Visit 2 must be conducted within 90 days from Visit 1.
  - The site should conduct the next visit if the participant's eligibility criteria are confirmed, and the site should document the reason for delay in the CRF.
  - Due to the pause in screening, sites should also reconfirm the impacted participant's consent and document this confirmation in the source documentation.
- If paused for more than 90 days from Visit 1 to Visit 2: The participant must be discontinued because of screening interruption due to an exceptional circumstance. This is documented as a screen failure in the CRF. The participant can reconsent and be rescreened as a new participant. The screening procedures per the usual SoA should be followed, starting at Visit 1 to ensure participant eligibility by Visit 2.

5. Adjustments to visit windows

Whenever possible and safe to do so, as determined by the investigator's discretion, participants should complete the usual SoA. To maximize the possibility that these visits can be conducted as on-site visits, the windows for visits may be adjusted, upon further guidance from the sponsor. This minimizes missing data and preserves the intended conduct of the study.

Randomization visit (Visit 10) should be completed as per original schedule whenever possible and safe to do so. However, the visit may be brought forward no sooner than 7 days or extended up to 14 days relative to the target visit date.

Primary endpoint visit (Visit 28 or 99) should be completed as per original schedule whenever possible and safe to do so. However, the visit may be brought forward no sooner than 14 days or extended up to 28 days relative to the target visit date.

For participants whose visits have extended windows, additional study intervention may need to be provided to avoid interruption and maintain overall integrity of the study.

Documentation

Changes to study conduct will be documented:

- Sites will identify and document the details of how participants, visit types, and conducted activities were affected by exceptional circumstances. Dispensing/shipment records of study intervention and relevant communications, including delegation, should be filed with site study records.
- Source documents generated at a location other than the study site should be part of the investigator's source documentation and should be transferred to the site in a secure and timely manner.

10.11. Appendix 11: Abbreviations

Term	Definition
**ADA**	antidrug antibody
**AE**	adverse event: Any untoward medical occurrence in a patient or clinical investigation subject administered a pharmaceutical product that does not necessarily have a causal relationship with this treatment. An adverse event can therefore be any unfavorable and unintended sign (including an abnormal laboratory finding), symptom, or disease temporally associated with the use of a medicinal (investigational) product, whether or not related to the medicinal (investigational) product.
**AESI**	adverse event of special interest
**ALP**	alkaline phosphatase
**ALT**	alanine aminotransferase
**AMA**	American Medical Association
**ANCOVA**	analysis of covariance
**AST**	aspartate aminotransferase
**baseline**	A fixed point of reference that is used for comparison purposes. In this study baseline may refer to lead-in enrollment visits (Visit 1 and Visit 2) or randomization visit.
**BG**	blood glucose
**BMI**	body mass index
**BMR**	basal metabolic rate
**CEC**	clinical endpoint committee
**CFR**	Code of Federal Regulations
**CHF**	congestive heart failure
**CIOMS**	Council for International Organizations of Medical Sciences
**CK**	creatine kinase
**CKD-EPI**	Chronic Kidney Disease-Epidemiology
**complaint**	A complaint is any written, electronic, or oral communication that alleges deficiencies related to the identity, quality, purity, durability, reliability, safety or effectiveness, or performance of a drug or drug delivery system.
**compliance**	Adherence to all study-related, GCP, and applicable regulatory requirements.
**CONSORT**	Consolidated Standards of Reporting Trials

**CRP**	clinical research physician: Individual responsible for the medical conduct of the study. Responsibilities of the CRP may be performed by a physician, clinical research scientist, global safety physician or other medical officer.
**CSR**	clinical study report
**C-SSRS**	Columbia-Suicide Severity Rating Scale
**CT**	computed tomography
**CTA**	Clinical Trail Agreement
**DMC**	Data monitoring committee
**DNA**	deoxyribonucleic acid
**DPP-4**	dipeptidyl-peptidase-4
**EAS**	efficacy analysis set
**ECG**	electrocardiogram
**eCRF**	electronic case report form
**ED**	early discontinuation
**EDC**	electronic data capture
**eGFR**	estimated glomerular filtration rate
**EMA**	European Medicines Agency
**enroll**	In this study enroll refers to all participants who enter the 12-week lead-in period at Visit 2.
**enter**	Participants entered into a study are those who sign the informed consent form directly or through their legally acceptable representatives.
**ERB**	Ethical Review Board
**FAS**	full analysis set
**FDA**	Food and Drug Administration
**FSH**	follicle-stimulating hormone
**GCP**	Good Clinical Practice
**GI**	gastrointestinal
**GIP**	glucose-dependent insulinotropic polypeptide
**GIPR**	glucose-dependent insulinotropic polypeptide receptor

**GLP-1**	glucagon-like peptide-1
**GLP-1R**	glucagon-like peptide-1 receptor
**HbA1c**	hemoglobin A1c
**HDL**	high-density lipoprotein
**HIPAA**	Health Insurance Portability and Accountability Act
**HRQoL**	health-related quality of life
**IB**	Investigator’s Brochure
**ICF**	informed consent form
**ICH**	The International Council for Harmonization of Technical Requirements for Pharmaceuticals for Human Use
**informed consent**	A process by which a patient voluntarily confirms his or her willingness to participate in a particular study, after having been informed of all aspects of the study that are relevant to the patient’s decision to participate. Informed consent is documented by means of a written, signed and dated informed consent form.
**INR**	international normalization ratio
**interim analysis**	An interim analysis is an analysis of clinical study data, separated into treatment groups, that is conducted before the final reporting database is created/locked.
**IP**	Investigational product; a pharmaceutical form of an active ingredient or placebo being tested or used as a reference in a clinical trial, including products already on the market when used or assembled (formulated or packaged) in a way different from the authorized form, or marketed products used for an unauthorized indication, or marketed products used to gain further information about the authorized form.
**IRB/IEC**	Institutional Review Boards /Independent Ethics Committees
**IWQOL-Lite-CT**	Impact of Weight on Quality of Life-Lite-Clinical Trials Version
**IWRS**	interactive web-response system
**LCD**	low calorie diet
**LDL**	low-density lipoprotein
**MAD**	multiple ascending dose
**MDD**	Major Depressive Disorder
**MedDRA**	Medical Dictionary for Regulatory Activities
**MEN**	multiple endocrine neoplasia
**MHP**	mental health professional

**mITT**	modified intention-to-treat
**MMRM**	mixed model for repeated measures
**MRI**	magnetic resonance imaging
**MTC**	medullary thyroid carcinoma
**MTD**	maximum tolerated dose
**NAFLD**	nonalcoholic fatty liver disease
**NYHA**	New York Heart Association
**p-amylase**	pancreatic amylase
**PAL**	physical activity level
**PCOS**	polycystic ovarian syndrome
**PGIs**	Patient Global Impression of status
**PHQ-9**	Patient Health Questionnaire-9
**PK/PD**	pharmacokinetics/pharmacodynamics
**PROs**	patient-reported outcomes
**QW**	once weekly
**randomized**	All participants who are indiscriminately assigned a study drug.
**SAD**	single ascending dose
**SAE**	serious adverse event
**SAP**	statistical analysis plan
**SC**	subcutaneous
**screen**	The act of determining if an individual meets minimum requirements to become part of a pool of potential candidates for participation in a clinical study.
**SD**	standard deviation
**SF-36v2**	Short Form-36 Health Survey (SF-36), version 2 acute form
**SoA**	Schedule of Activities
**SS**	Safety analysis set
**T1DM**	Type 1 diabetes mellitus
**T2DM**	Type 2 diabetes mellitus

**TBL**	total bilirubin level
**TE**	Treatment-emergent
**TE ADA+**	treatment-emergent antidrug antibody positive
**TEAE**	treatment-emergent adverse event: An untoward medical occurrence that emerges during a defined treatment period, having been absent pretreatment, or worsens relative to the pretreatment state, and does not necessarily have to have a causal relationship with this treatment.
**TEE**	total energy expenditure
**THC**	tetrahydrocannabinol
**TSH**	thyroid-stimulating hormone
**ULN**	upper limit of normal
**VLCD**	very low calorie diet
**WHO**	World Health Organization

11. References

[ADA]. Glycemic targets: standards of medical care in diabetes- 2020. *Diabetes Care* 2020(suppl 1):S66–S76. https://doi.org/10.2337/dc20-S006

[AMA]. American Medical Association House of Delegates resolution: 420 (A-13): recognition of obesity as a disease. May 2013. Accessed May 21, 2020. https://media.npr.org/documents/2013/jun/ama-resolution-obesity.pdf

Aroda VR, Ratner R. The safety and tolerability of GLP-1 receptor agonists in the treatment of type 2 diabetes: a review. *Diabetes Metab Res Rev*. 2011;27(6):528-542. https://doi.org/10.1002/dmrr.1202

Baggio LL, Drucker DJ. Biology of incretins: GLP-1 and GIP. *Gastroenterology*. 2007;132(6):2131-2157. https://doi.org/10.1053/j.gastro.2007.03.054

Banks PA, Freeman ML. Practice Parameters Committee of the American College of Gastroenterology. Practice guidelines in acute pancreatitis. *Am J Gastroenterol.* 2006;101(10):2379-2400. https://doi.org/10.1111/j.1572-0241.2006.00856.x

Coskun T, Sloop KW, Loghin C, et al. LY3298176, a novel dual GIP and GLP-1 receptor agonist for the treatment of type 2 diabetes mellitus: from discovery to clinical proof of concept. *Mol Metab*. 2018;18:3-14. https://doi.org/10.1016/j.molmet.2018.09.009

Council on Science and Public Health. Report of the Council on Science and Public Health: is obesity a disease? (Resolution 115-A-12): Report number: 3-A-13. 2013. Accessed May 21, 2020. https://www.ama-assn.org/sites/ama-assn.org/files/corp/media-browser/public/about-ama/councils/Council%20Reports/council-on-science-public-health/a13csaph3.pdf.

Danne T, Philotheou A, Goldman D, et al. A randomized trial comparing the rate of hypoglycemia – assessed using continuous glucose monitoring – in 125 preschool children with type 1 diabetes treated with insulin glargine or NPH insulin (the PRESCHOOL study). *Pediatr Diabetes*. 2013;14(8):593-601. https://doi/10.1111/pedi.12051

Dolan P. Modeling valuations for EuroQol health states. *Medical Care.* 1997;35(11):1095-1108. https://doi/10.1097/00005650-199711000-00002

Doucet É, McInis K, Mahmoodianfard S. Compensation in response to energy deficits induced by exercise or diet. *Obesity Rev*. 2018;19(suppl1):36-46. https://doi.org/10.1111/obr.12783

[EMA]. Guideline on clinical evaluation of medicinal products used in weight management. EMA/CHMP/311805/2014. Published June 23, 2016. Accessed May 22, 2020https://www.ema.europa.eu/en/documents/scientific-guideline/guideline-clinical-evaluation-medicinal-products-used-weight-management-revision-1_en.pdf. Published 23 June 2016.

EuroQol Research Foundation. EQ-5D-5L User Guide, Version 3.0. Updated September 2019. Accessed March 13, 2020. https://euroqol.org/publications/user-guides.

[FAO/WHO/UNU] Food and Agriculture Organization of the United Nations/World Health Organization/United Nations University. Human energy requirements: report of a joint

FAO/WHO/UNU expert consultation. Published October 2004 Accessed May 21, 2020.http://www.fao.org/3/y5686e/y5686e00.htm.

[FDA]. Guidance for industry: developing products for weight management. Published February 2007. Accessed May 22, 2020. https://www.fda.gov/media/71252/download.

[FDA]. FDA requests the withdrawal of the weight-loss drug Belviq, Belviq XR (lorcaserin) from the market: potential risk of cancer outweighs the benefits. Published 14 January 2020. Accessed June 5, 2020. https://www.fda.gov/media/135189/download.

Frias JP, Nauck MA, Van J, et al. Efficacy and safety of LY3298176, a novel dual GIP and GLP-1 receptor agonist, in patients with type 2 diabetes: a randomized, placebo-controlled and active comparator-controlled phase 2 trial. *Lancet.* 2018;392(10160):2180-2193. https://doi.org/10.1016/S0140-6736(18)32260-8

Garvey WT, Mechanick JI, Brett EM, et al. American Association of Clinical Endocrinologists and American College of Endocrinology comprehensive clinical practice guidelines for medical care of patients with obesity. *Endocr Pract*. 2016;22(suppl 3):1-203. https://doi.org/10.4158/EP161365.GL

Gerstein HC, Colhoun HM, Dagenais GR, et al. Dulaglutide and cardiovascular outcomes in type 2 diabetes (REWIND): a double-blind, randomized placebo-controlled trial. *Lancet.* 2019;394(10193):121-130. https://doi.org/10.1016/S0140-6736(19)31149-3

Goldstein DJ. Beneficial health effects of modest weight loss. *Int J Obes Relat Metab Disord*. 1992;16(6): 397-41

Heymsfield SB, van Mierlo CA, van der Knaap HC, Heo M, Frier HI. Weight management using a meal replacement strategy: meta and pooling analysis from six studies. *Int J Obes Relat Metab Disord.* 2003 May;27(5):537-49. https://doi: 10.1038/sj.ijo.0802258. PMID: 12704397.

Jensen MD, Ryan DH, Donato KA, et al. Executive summary: guidelines (2013) for the management of overweight and obesity in adults: a report of the American College of Cardiology/American Heart Association Task Force on Practice Guidelines and the Obesity Society published by the Obesity Society and American College of Cardiology/American Heart Association Task Force on Practice Guidelines. based on a systematic review from the Obesity Expert Panel, 2013. *Obesity.* 2014;22(suppl 2):S5-S39. https://doi.org/10.1002/oby.20821

Knowler WC, Barrett-Connor E, Fowler SE, et al. Reduction in the incidence of type 2 diabetes with lifestyle intervention or metformin. *N Engl J Med.* 2002;346(6):393-403. https://doi.org/10.1056/NEJMoa012512

Koizumi M, Takada T, Kawarada Y, et al. JPN Guidelines for the management of acute pancreatitis: diagnostic criteria for acute pancreatitis. *J Hepatobiliary Pancreat Surg*. 2006;13(1):25-32. https://doi.org/10.1007/s00534-005-1048-2

Kolotkin RL, Andersen JR. A systematic review of reviews: exploring the relationship between obesity, weight loss and health-related quality of life. *Clin Obes*. 2017;7(5):273-289. https://doi.org/10.1111/cob.12203

Kolotkin RL, Ervin CM, Meincke HH, et al. Development of a clinical trials version of the Impact of Weight on Quality of Life-Lite questionnaire (IWQoL-Lite Clinical Trials Version):

results from two qualitative studies. *Clin Obes.* 2017;7(5):290-299. https://doi.org/10.1111/cob.12197

Kolotkin RL, Williams VSL, Ervin CM, et al. Validation of a new measure of quality of life in obesity trials: Impact of Weight on Quality of Life – Lite Clinical Trials Version. *Clin Obes.* 2019;9(3):e12310. https://doi.org/10.1111/cob.12310

Lauby-Secretan, B, Scoccianti C, Loomis D, et al.; International Agency for Research on Cancer Handbook Working Group. Body fatness and cancer-viewpoint of the IARC Working Group. *N Engl J Med.* 2016;375(8):794-798. https://doi.org/10.1056/NEJMsr1606602

Leibel RL, Rosenbaum M, Hirsch J. Changes in energy expenditure resulting from altered body weight. *N Engl J Med*. 1995;332(10):621-628. https://doi.org/10.1056/NEJM199503093321001

Luppino FS, de Wit LM, Bouvy PF, et al. Overweight, obesity, and depression: a systematic review and meta-analysis of longitudinal studies. *Arch Gen Psychiatry.* 2010;67(3):220-229. https://doi.org/10.1001/archgenpsychiatry.2010.2

Marso SP, Bain SC, Consoli A, et al. Semaglutide and cardiovascular outcomes in patients with type 2 diabetes. *N Engl J Med.* 2016a;375(19):1834-1844. https://doi.org/10.1056/NEJMoa1607141

Marso SP, Daniels GH, Brown-Frandsen K, et al. Liraglutide and cardiovascular outcomes in type 2 diabetes. *N Engl J Med.* 2016b;375(4):311-322. https://doi.org/10.1056/NEJMoa1603827

Maruish ME, ed. *User's Manual for the SF-36v2 Health Survey*. 3rd ed. Lincoln, RI: Quality Metric Incorporated; 2011.

Mertens IL, Van Gaal LF. Overweight, obesity, and blood pressure: the effects of modest weight reduction. *Obes Res*. 2000;8(3):270-278. https://doi.org/ 10.1038/oby.2000.32

Müller TD, Clemmensen C, Finan B, et al. Anti-obesity therapy: from rainbow pills to polyagonists. *Pharmacol Rev*. 2018;70(4):712-746. https://doi.org/10.1124/pr.117.014803

Nauck MA, Meier JJ, Schmidt WE. Incretin-based glucose-lowering medications and the risk of acurte pancreatitis and/or pancreatic cancer: reassuring data from cardio-vascular outcome trials. *Diabetes Obes Metab*. 2017;19(9):1327-1328. https://doi.org/10.1111/dom.12981

[NHLBI]. Managing overweight and obesity in adults: systematic evidence review from the Obesity Expert Panel, 2013. Available at: https://www.nhlbi.nih.gov/sites/default/files/media/docs/obesity-evidence-review.pdf. Published 2013. Accessed June 12, 2020.

[NHLBI]. The practical guide identification, evaluation, and treatment of overweight and obesity in adults. Available at: https://www.nhlbi.nih.gov/files/docs/guidelines/prctgd_c.pdf. Published October 2000. Accessed June 12, 2020.

Ryan DH, Yockey SR. Weight loss and improvement in comorbidity: differences at 5%, 10%, 15%, and over. *Curr Obes Rep.* 2017;6(2):187-194. https://doi.org/10.1007/s13679-017-0262-y

SAXENDA [summary of product characteristics]. Bagsværd, Denmark: Novo Nordisk, 2015.

SAXENDA [package insert]. Bagsværd, Denmark: Novo Nordisk; 2014.

Skow MA, Bergmann NC, Knop FK. Diabetes and obesity treatment based on dual incretin receptor activation: "twincretins". *Diabetes Obe Metab.* 2016;18(9):847-854. https://doi.org/10.1111/dom.12685

Srivastava G, Apovian CM. Current pharmacotherapy for obesity. *Nat Rev Endocrinol.* 2018;14(1):12-24. https://doi.org/10.1038/nrendo.2017.122

Steinberg WM, Buse JB, Ghorbani MLM, et al. LEADER Steering Committee; LEADER Trial Investigators. Amylase, lipase, and acute pancreatitis in people with type 2 diabetes treated with liraglutide: results from the LEADER randomized trial. *Diabetes Care.* 2017a;40(7):966-972. https://doi.org/10.2337/dc16-2747

Steinberg WM, Rosenstock J, Wadden TA, et al. Impact of liraglutide on amylase, lipase, and acute pancreatitis in participants with overweight/obesity and normoglycemia, prediabetes, or type 2 diabetes: secondary analyses of pooled data from the SCALE clinical development program. *Diabetes Care.* 2017b;40(7):839-848. https://doi.org/10.2337/dc16-2684

Sumithran P, Prendergast LA, Delbridge E, et al. Long-term persistence of hormonal adaptations to weight loss. *N Engl J Med*. 2011;365(17):1597-1604. https://doi.org/10.1056/NEJMoa1105816

Tomlinson B, Hu M, Zhang Y, et al. An overview of new GLP-1 receptor agonists for type 2 diabetes. *Expert Opin Investig Drugs*. 2016;25(2):145-158. https://doi.org/10.1517/13543784.2016.1123249

van Bloemendaal L, Ten Kulve JS, la Fleur SE, et al. Effects of glucagon-like peptide 1 on appetite and body weight: focus on the CNS. *J Endocrinol*. 2014;221(1):T1-T16. https://doi.org/10.1530/JOE-13-0414

Wadden TA, Foster GD, Letizia KA. One-year behavioral treatment of obesity: comparison of moderate and severe caloric restriction and the effects of weight maintenance therapy. *J Consult Clin Psychol*. 1994;62(1):165-171. https://doi.org/10.1037//0022-006x.62.1.165

Weinberg ME, Bacchetti P, Rushakoff RJ. Frequently repeated glucose measurements overestimate the incidence of inpatient hypoglycemia and severe hyperglycemia. *J Diabetes Sci Technol*. 2010;4(3):577-582. https://doi.org/10.1177/193229681000400311

[WHO]. WHO STEPS surveillance manual: the WHO STEPwise approach to noncommunicable disease risk factor surveillance. Updated 26 January 2017. Accessed May 4, 2020. https://www.who.int/ncds/surveillance/steps/STEPS_Manual.pdf.

Wing RR, Lang W, Wadden TA, et al. ; Look AHEAD Research Group. Benefits of modest weight loss in improving cardiovascular risk factors in overweight and obese individuals with type 2 diabetes. *Diabetes Care*. 2011; 34(7):1481-1486. https://doi.org/ 10.2337/dc10-2415

Leo Document ID = a1f37e94-98cb-4d3e-b822-c86293aa4b40

Approver: PPD

Approval Date & Time: 19-Nov-2020 21:02:18 GMT

Signature meaning: Approved

Approver: PPD

Approval Date & Time: 19-Nov-2020 21:05:48 GMT

Signature meaning: Approved
